# Pharmaceutical industry use of key opinion leaders to market prescription opioids: A review of internal industry documents

**DOI:** 10.1016/j.rcsop.2024.100543

**Published:** 2024-11-19

**Authors:** Brian Gac, Kgosi Tavares, Hanna Yakubi, Hannah Khan, Dorie E. Apollonio, Eric Crosbie

**Affiliations:** a Department of Clinical Pharmacy, University of California, San Francisco, San Francisco, CA, United States of America; b School of Public Health, University of Nevada Reno, Reno, NV, United States of America; c Department of Pharmacy, University of California, Davis Health System, Sacramento, CA, United States of America; d University of Nevada, Reno School of Medicine, Reno, NV, United States of America; e Ozmen Institute for Global Studies, University of Nevada Reno, Reno, NV, United States of America

**Keywords:** Analgesics, Opioid, Conflict of interest, Consultants, Inappropriate prescribing, Marketing

## Abstract

**Objective:**

Prescription opioid-related deaths increased by 200 % from 2000 to 2014. There has been limited research regarding channels used by pharmaceutical companies to market prescription opioids. In this study, we investigated pharmaceutical industry use of key opinion leaders (KOLs).

**Methods:**

We conducted a retrospective qualitative review of the first 503 opioid industry documents publicly released, which are held at the University of California, San Francisco Opioid Industry Document Archive (OIDA). We reviewed documents including legal rulings, correspondences, witness statements, clinical studies, and corporate communications for relevance and coded them by themes.

**Results:**

Between 2001 and 2019, pharmaceutical companies including Janssen, Purdue Pharma, and Cephalon identified, recruited and developed individuals they referred to as “Key Opinion Leaders,” (KOLs) that they recognized could reach strategic audiences to influence prescriber behaviors. Pharmaceutical companies identified KOLs through a variety of sources ranging from partnerships with PR firms to social media analysis and congressional and regulatory sources. Companies recruited KOLs through various methods including surveys to identify common names identified by physicians, internal rankings based on friendliness, and opioid prescribing behaviors. Companies employed KOLs as speakers at conferences for branded opioid products, authors of research articles in support of prescription opioids, and consultants regarding marketing strategies.

**Conclusions:**

KOLs were employed by the pharmaceutical industry to leverage their reputations in the service of encouraging healthcare providers to prescribe more opioids. It is critical to ensure that researchers and leaders in the medical field are aware and critical of pharmaceutical corporate profit-led biases and are free from conflicts of interest to avoid inappropriate prescribing and minimize adverse outcomes for patients.

## 1 Introduction

A key opinion leader (KOL) is “an influential physician or researcher who is held in high esteem by their colleagues”.^36^ In the 1940s, communications research found that most people's beliefs are driven by the influence of trusted figures within their social networks, referred to as “opinion leaders”.^22^ The use of KOLs has been particularly common the pharmaceutical industry and was first identified in the research literature in 1966.^9^ Although KOLs express belief in the products they promote and their audiences of healthcare providers trust that they can identify bias, both are strongly influenced by the companies that provide preferred evidence in support of commercial interests.^36^

Pharmaceutical companies incorporated multiple tactics to market prescription opioids and increase sales, a key factor in the opioid epidemic. For example, Purdue Pharma advertised “off-label” (not FDA-approved) uses of opioids and minimized risks of addiction, overdose, and death from OxyContin to encourage physician prescribing.^12^ Spending on direct-to-consumer advertising of drugs increased from $2.1 billion in 1997 to $9.6 billion in 2016. However, the majority of marketing targeted healthcare professionals, with spending rising to $20.3 billion in 2016.^35^ Traditionally, pharmaceutical companies relied on sales representatives to market products to physicians.^42^ Later, they recruited and supported “key opinion leaders” (KOLs), who are physicians that influence the practice of medicine and prescribing behavior for their peers,^30^ and who are recruited by and work with pharmaceutical companies to advise on the marketing of medications.^25^ Specifically, we sought to assess how the pharmaceutical industry identified and relied on opinion influencers to disseminate their marketing messages to increase opioid prescribing. Understanding these methods is critical to developing regulations that reduce future overprescribing of medications with the potential for abuse and dependence and protect public health.

Opioids are a class of drugs used for pain management when legally prescribed, and can also be in the form of illegal drugs, such as heroin or illicitly manufactured fentanyl.^6^ The overprescribing of opioid medications began in the 1990s when state medical boards loosened prescription regulations on opioids for non-cancer chronic pain following advocacy efforts by organizations such as the American Pain Society.^7^ At the same time, the pharmaceutical industry began funding aggressive marketing campaigns that contributed to the opioid overdose epidemic.^7^ Overdose deaths involving opioids continued to rise in the U.S. into the 2020s.^43^ Although opioid overdose deaths had begun increasing by the year 2000, the effects were only widely reported beginning in 2012.^29^ By 2013, illicit synthetic opioids had begun contributing substantially to opioid deaths.^10^ Illicit drug manufacturing in the U.S. can involve mixing synthetic opioids with other drugs, and “pressing synthetic opioids into counterfeit tablets that look like commonly misused prescription opioids,”.^10^ The overprescribing of prescription opioids (e.g. OxyContin and Percocet) contributed to the first wave of the opioid epidemic, and people who first became addicted to prescription opioids demonstrated an increased risk to transition to the use of illicit opioids.^6^

Multiple civil lawsuits have been filed against pharmaceutical companies regarding the opioid epidemic. In 2017, the state of Oklahoma filed a lawsuit against Purdue Pharma regarding a marketing campaign that increased opioid prescribing by providing misinformation.^16^ As a result of this litigation, 503 previously secret internal industry documents produced by over a dozen pharmaceutical companies were released to the public in January 2020. These documents detail industry marketing tactics used to increase the sales of prescription opioids.^44^ Comparable litigation has since expanded to include distributors such as McKesson, retail pharmacies like Walgreens and CVS, management consultants, and pharmacy benefit managers.^16^ Past research has investigated pharmaceutical industry efforts to increase sales of opioids before and during the opioid crisis.^7^^,^^31^^,^^32^

Similar to other industry document research,^4^ access to internal pharmaceutical industry documents allows researchers to analyze the motivations, development, and recruitment strategies these companies employed to utilize opinion influencers to promote their products. Before the release of previously secret industry documents, research studies surrounding pharmaceutical industry marketing primarily focused on how the industry marketed towards physicians using sales representatives, video advertising, and branded promotional items such as pens, mugs, or clothing.^42^ More recent research has considered how pharmaceutical companies have marketed to physicians through writing industry-funded research articles and promoting favorable continuing medical education modules.^13^^,^^17^ A 2024 study documented opioid industry efforts to increase opioid prescribing by oncologists that relied, in part, on the use of KOLs.^23^ This study expands on this work by considering the channels through which materials promoting opioids were disseminated.

## 2 Methods

This study relied on a retrospective qualitative review of industry documents released in *State of Oklahoma v. Purdue Pharma, L.P.* et al. Since 2020, documents made public in litigation against pharmaceutical companies have been available through the Opioid Industry Document Archive (OIDA) at the University of California San Francisco.^40^ On January 24, 2020, OIDA posted the first 503 documents in the collection, totaling over 62,000 pages and released as part of the Oklahoma litigation. These documents included clinical studies, witness declarations, internal corporate communications, and marketing strategies regarding opioids, and served as the primary data source for the study.^27^

The review we conducted of all 503 documents in the Oklahoma collection, as well as the coding strategy, are described in detail elsewhere.^44^ The complete coding instrument and summaries of each document and its codes are provided in Supplement 1 (also available at the Open Science Foundation, https://doi.org/10.17605/OSF.IO/GCKMB). Briefly, two authors (BG, HY) conducted an initial review of all documents in the collection, excluding those documents that did not contain relevant information due either to legal redaction or because they could not be coded (e.g., lists of calls to unnamed providers). Key concepts were identified inductively by one of these two authors, and the coding was reviewed jointly with a third author (DA) while the team re-read each document until a consensus was reached. Key concepts relevant to this manuscript were identified by one author (BG). From the final list of documents relevant to the key concepts identified in this study, this author selected 15 documents that most completely described the efforts made to recruit and leverage KOLs, which included materials referring to the design and implementation of marketing campaigns, recruitment and payment of KOLs, and reports describing subsequent changes in prescribing patterns. The 15 documents comprised legal rulings, correspondence, witness statements, clinical studies, and corporate communications. These documents were discussed by three authors (BG, HY, DA) and then reviewed again by two independent authors (KT, EC) to confirm the interpretation. The detailed descriptions of the 15 documents used in this manuscript have been provided separately in Supplement 2.

## 3 Results

The following sections present an overview of how the pharmaceutical companies, including Cephalon, Purdue Pharma, and most notably, Janssen, first recognized the importance of KOLs and then proceeded to identify and recruit them to market prescription opioids. The sections below cover documents over a two-decade period (2001–2019) and detail how pharmaceutical companies invested in the development of KOLs they identified by providing research funding, educating them about abuse, and training KOLs to deliver more consistent and comprehensive messages to leverage support to increase opioid prescribing.

### 3.1 Recognizing the importance of KOLs

Multiple pharmaceutical companies identified KOLs as critical to increase opioid sales. An email from Janssen medical science liaison (MSL), a healthcare professional who works with the medical community to educate them about a company's products and advances, Heather Thomson to several co-workers in 2002 listed several ways that KOLs were useful in advocating for the company's opioid product Duragesic.^39^ This included instructing advocates that they “can present well-balanced completely fair material, but also Alert MSL to upcoming publications of interest, Keep MSL abreast of developments within institutions and organizations, Position Duragesic appropriately and enthusiastically in chronic pain, Dispel myths about opioids and Duragesic in their teaching and talks, Create, and/or review drafts of algorithms and guidelines with an eye to suitable mention of Duragesic, Say, ‘Use non-invasive measures first like patches or orals’ when giving the WHO pain lecture, Introduce MSL to other practitioners and researchers of interest, Are considered ‘good’ speakers when funded by Janssen either as live or teletopics presenters,”.^39^

**Fig. 1:**
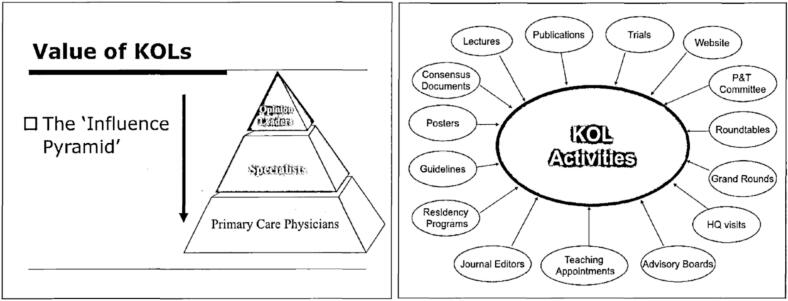
Select slides from a draft internal Cephalon sales strategy presentation regarding the scope of key opinion leader influence.

A draft internal Cephalon sales strategy presentation slide deck from February 2005, defined what the industry perceived as KOLs, highlighting the importance of targeting select individuals who could influence prescribing practices on a much broader scale than pharmaceutical sales representatives through a variety of activities (see Fig. 1).^26^ The presentation emphasized the value of KOLs, stating, “the #1 reason a MD [Medical Doctor] changes prescribing behavior is due to **peers**, healthcare professionals learn through an apprenticeship model…..throughout their careers, [and] in spite of their importance…most pharma companies do not currently have an effective system in place to identify, manage or develop Ols [opinion leaders, short for KOLs],”.^26^ It continued, “[Opinion leaders] help to shape: Clinical drug development, Product positioning, Brand development/life cycle management, Prescribing practices, ….. i.e. $$$.”^26^ Additionally, the presentation emphasized that a “Small number of KOLs (<100) influence hundreds of prescribers,” and, “A small number of prescribers (<500) generate large dollar volume,”.^26^

Purdue Pharma likewise recognized the value of KOLs in a 2011 Corporate Reputation and Visibility Strategic Plan,^33^ which they continued to pursue even after pleading guilty and paying fines totaling $600 million for “misbranding OxyContin…with the intent to defraud or mislead,” in 2007.^21^ The plan highlighted maximizing existing external relationships and creating and building new ones by stating, “Key opinion leaders (KOLs) and professional associations can support or interfere with the company's efforts to reach key audiences. KOLs can influence healthcare professionals' prescribing practices. The emergence of new competition [federal, state and local policies], the 2005 corporate downsizing, and the WDVA [Western District of Virginia] settlement agreement has had a negative impact on Purdue's relationship with KOLs. The company has an opportunity to recapture these positive relationships through coordinated liaison and communications efforts,”.^33^

### 3.2 Identifying supportive KOLs

Pharmaceutical companies employed multiple means to identify KOLs. Janssen developed categories of KOLs ranking them in order of “friendliness” by how strongly they might be relied upon to advocate for a particular product or for opioid use in general, ranging from “Advocates” to “Neutrals” to “Opponents.” In a February 2002 email discussion to other employees on how to classify KOLs, Janssen MSL Heather Thomson described KOL characteristics that classified KOLs as more or less friendly.^39^ Thomson described “Opponents” as “actively hurting the cause and can qualify by ‘Personally [blocking] access to the department for the sales representative,” or, “Get hysterical, as opposed to intelligently concerned, about the abuse and diversion of opioids,”.^39^ Thomson described “Neutrals” who “probably [would] consider themselves good friends of Janssen but Never mention opioids or Duragesic in their talks,” or “Inadvertently pigeon-hole Duragesic for NPO, bowel-obstructed, etc. patients,”.^39^ Finally, Thomson described “Advocates” as those that “can present well-balanced completely fair material and “can dispel myths about opioids and Duragesic in their teaching and talks,”.^39^ In the email, Thomson went on further to state, “Are you guys OK with the concept that just because a KOL is obnoxious, difficult or a pain it doesn't mean he or she is an opponent? Conversely, that a sweetie who just isn't putting out for you shouldn't qualify as an advocate,”.^39^

Janssen also identified which aspects of KOLs' scopes of influence had the largest effect on prescribers. An email from Janssen Pain Product Director Stephen E. Cornwell to several co-workers in July 2006 detailed a mapping analysis of KOLs that resulted in creating a master list of KOLs.^11^ The mapping analysis found that prescribers who valued the “Influence of Opinion Leaders” derived that value more from the number of KOL events they attended than from reading journal articles the KOL had published. In addition, prescribers “tend to read (or remember reading) articles by the same authors that they remember hearing as speakers,”.^11^

**Fig. 2:**
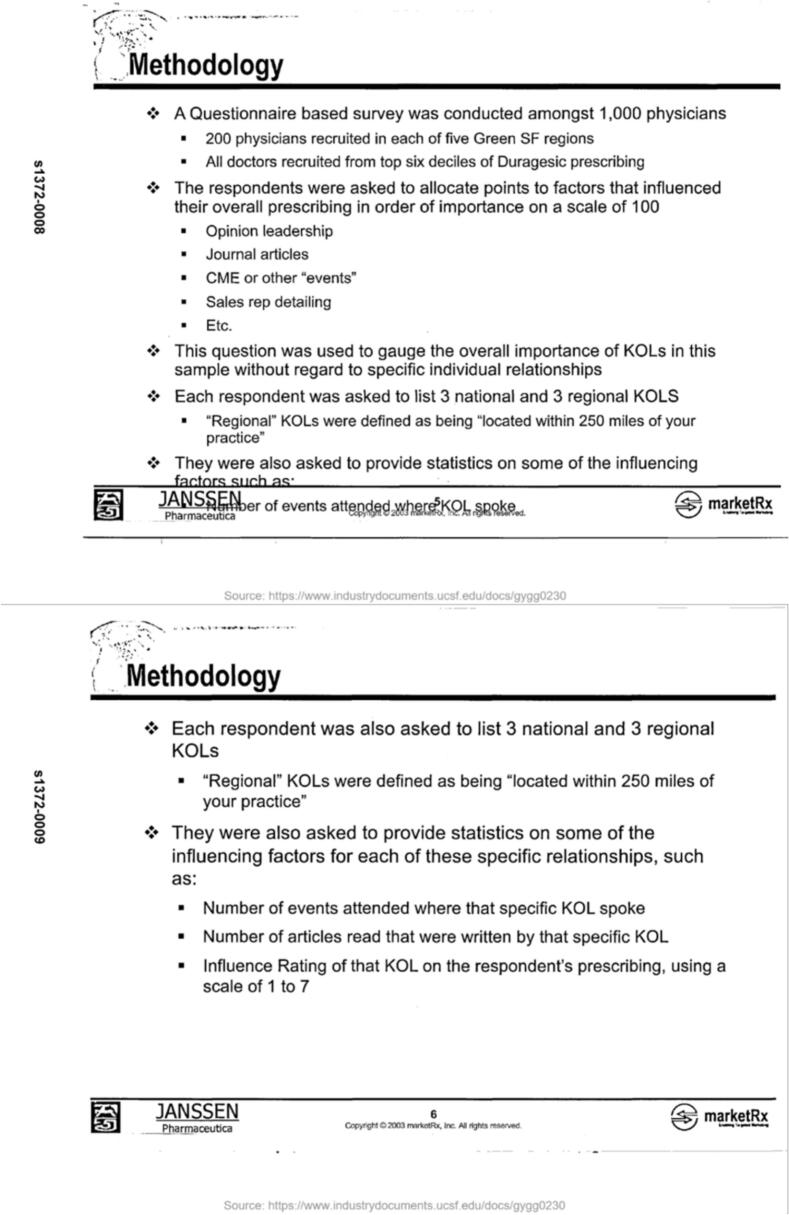
Methodology for a Janssen survey to physicians for the purpose of identifying key opinion leaders in pain management.

In 2004, Janssen, in partnership with life sciences market analytics firm marketRx, conducted a “Questionnaire based survey” that recruited 1000 physicians to identify the KOLs with the most influence over their prescribing practices^11^ (Fig. 2). The survey targeted physicians that prescribed the most Duragesic, a fentanyl patch manufactured by Janssen, including primary care providers, rheumatologists, neurologists, surgeons, and pain specialists.^11^ Ultimately, “886 National KOLs were identified” by the survey, but there were, “A relatively small number of doctors with multiple mentions,”.^11^

**Fig. 3:**
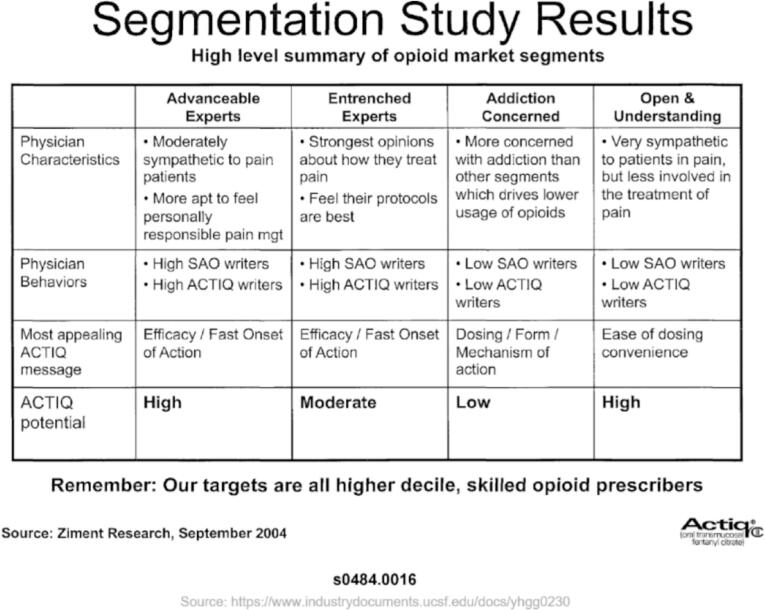
Classification of Key Opinion Leaders based on conversations with KOLs that had been preliminarily characterized as friendly (“good eggs”) or unfriendly (“bad eggs”) to opioids and Actiq specifically.

Cephalon/Teva, manufacturer of Actiq (an oral transmucosal fentanyl product) used a variety of sources to identify KOLs, ranging from partnerships with PR firms to “social media analysis” and “Congressional and regulatory sources,”.^38^ A 2004 slide deck for a Director's Meeting presentation about Actiq had similar categories to those from Janssen, classifying KOLs as “good eggs” and “bad eggs,”.^1^ The company's conversations with these prescribers led to their categorization by “ACTIQ potential,” based on attitudes that ranged from moderately to very sympathetic and behaviors such as high or low prescribing levels, shown in Fig. 3.^1^

### 3.3 Development of KOLs

Pharmaceutical companies also invested in the development of KOLs they identified. In their 2004 Business plan for Duragesic (fentanyl transdermal patch), Janssen sought to, “Leverage functionality positioning to differentiate DURAGESIC® from the competition,” as a key strategy by “Continu[ing] to leverage KOL relationship,” in medical affairs, the National Pain Education Council, and the National Pain Summit.^19^ They also sought to, “Build on DURAGESIC's current success to ensure rapid Equity transfer to AP-48 [new generation of fentanyl transdermal system],” by, “Enhanc[ing] KOL, Pharmacist & key customer relationships within managed market segments,”.^19^ This plan allocated $360,000 for 2004 to, “Develop a market influence map, database and communications program to formalize and better manage our KOL relationships as we transition to AP-48.”^19^ These opioid-friendly KOLs also helped the company develop its marketing strategies; Janssen dedicated an additional $1.3 million to build, “programs to solicit advice [from Advocate KOLs]… on issues which may impact the chronic pain market,”.^19^

### 3.4 Janssen messaging to KOLs: research funding

Janssen also examined the prospects of funding research projects that could benefit the drugs they manufacture and educate clinicians about drug abuse and addiction. At a Board meeting in November 2001, Janssen Director of Medical Development “Gary V[orsanger, internal medical specialist] urged physicians to bring all ideas to their MSLs and allow them to determine if a project was feasible and where funding might be found. Bill Whyte [Janssen Product Director, Analgesics] recommended that projects be clearly defined when shared with MSLs, by which he meant not only the scope, timeframe and costs, but also the anticipated end results. He also pointed out the financial limitations on funding research and the rigorous measure of return on investment when larger grants are awarded,”.^8^ One KOL, Dr. Russell Portenoy MD, later made a statement during the 2019 lawsuit against Johnson & Johnson that, “I believe that drug company research grants to researchers working in academic centers or health care facilities after a drug is approved for marketing almost always align with the company's interest in demonstrating the benefits of the drugs they manufacture, with the intention of publishing results that could yield higher sales in the future,”.^37^ Dr. Portenoy went on further, “[T]he amount of funding provided by drug companies for the purpose of education clinicians about drug abuse and addiction, and for the purpose of clinical research into the risk of abuse and addiction, was very limited between the 1980s and 2000s,”.^37^

### 3.5 Advice from KOLs: limit discussion of abuse potential

Janssen created a “Chronic Pain Scientific Advisory Board” that held events to formally develop relationships between these KOLs and the company.^8^ A confidential agenda for a Chronic Pain Scientific Advisory Board meeting from November 2001 revealed several key messages from Janssen to their KOLs and the opinions KOLs were providing to Janssen as experts in their field. The executive summary stated that a review of market trends in long-acting opioid prescribing led to a discussion about the negative effects of OxyContin abuse stories in the media,”^8^ which included stating, “Opiophobia has increased as a result, with short- and long-acting opioid prescribing going down and patients suffering as well as prescribers… The FDA perception of opioids is affected. We need an educational policy and strategies to educate physicians AND the FDA and their physician-advisors about the proper use of opioids,”.^8^ When asked, “Should the abuse potential of Duragesic be discussed?” KOLs responded, “’NO’ – resounding and unanimous. It is bad for the LAO class and bad for patients and prescribers,”.^8^

A “key message” to be delivered to physicians in a July 2009 Ortho McNeil Janssen internal training presentation read, “Although many physicians are reluctant to prescribe controlled substances, the risks (for both patient addiction/misuse and physician disciplinary action) are much smaller than commonly believed,”.^28^ Additionally, associates are instructed to, “Avoid the Addiction Ditch,” and that, “Many HCPs will find the 2.6% incidence of addiction to be extremely low,”.^28^

### 3.6 Attempts to enhance KOL influence

Rather than relying on a KOL's established expertise, a Janssen 2004 business plan stated, “Trained speakers deliver a more consistent and comprehensive message,”.^19^ Pharmaceutical companies conducted significant speaker training and clearly defined speaker agreements but sometimes these guidance documents were at odds with one another. For example, a contract between Cephalon and KOL Charles Argoff delineated, “Cephalon may engage Healthcare Provider [Argoff] to participate as a speaker in company-sponsored programs regarding its marketed products,” but, “Healthcare Provider is not obligated or expected to provide any particular promotional message or to otherwise induce, encourage, or recommend prescribing activity or use of any Cephalon product,”.^3^ However, a speaker training slide deck for Cephalon's Nucynta [tapentadol and tapentadol extended-release tablets] pointed out, “We want physicians to make NUCYNTA ER **their** LAO choice for the appropriate patient with moderate to severe chronic pain,”.^20^ The speaker training also specified, “Promotional speakers MUST: Remain on label,…Not solicit off-label inquiries,” but then grants a fair amount of latitude in handling off-label questions.^20^ Speakers may, “Provide an answer from [their] scientific knowledge that is within the scope of the solicited request, factual, objective, non-promotional, and fairly balanced;” so long as they, “Disclose that the response reflects [their] own views and does not represent the Company's position;” before they, “Transition back to the Company approved slide deck,”.^20^

Speakers were well compensated for their efforts. In a document outlining Janssen's payments to KOLs in preparation for a 2019 deposition, they reported paying Dr. Argoff upwards of $37,288.15 in one year, 2014.^18^ OpenPaymentsData.CMS.gov, a Center for Medicare & Medicaid Services reporting industry payments to providers since 2013, reported Argoff actually received $49,408.72 from Janssen in 2014.^2^ He received $240,170.05 from pharmaceutical companies overall that year and took in over $300,000 the following year.^2^

### 3.7 Utilization of KOLs

Once relationships were established, the influence of KOLs was leveraged to support increased opioid prescribing. One of the most common ways KOLs were used to shape medical consensus was through the authorship of articles, both in clinical research and less formally in unbranded efforts that were favorable to opioids. One doctor recruited by Janssen, Keith Candiotti, authored an article titled “Use of Opioid Analgesics in Pain Management” in 2015 that provided extremely broad usage recommendations for opioids as a class.^5^ It claimed, “Opioid analgesics, for example, have no true ‘ceiling dose’ for analgesia and do not cause direct organ damage … With the exception of constipation, many patients often develop tolerance to most of the opioid analgesic-related side effects,”.^5^ Discussion of addiction referred to it as a “nonmedical issue” and the article concluded, “While [concerns about addiction] are not without some merit, it would appear that they are often overestimated. According to clinical opinion polls, true addiction occurs only in a small percentage of patients with chronic pain who receive chronic opioid analgesics analgesic therapy,”.^5^ Candiotti's article claimed that he, “received compensation from Janssen Pharmaceuticals, Inc. for [his] contributions to PrescribeResponsibly.com,” which was an unbranded website funded by Janssen promoting the use of opioids to physicians.^5^

## 4 Discussion

This study investigated how pharmaceutical companies leveraged KOLs to market prescription opioids. KOLs were identified and recruited by pharmaceutical companies, who observed their influence over the prescribing practices of other healthcare professionals and leveraged that influence. KOLs were identified by how often they prescribed opioids, their willingness to advocate for opioids, and the extent to which they could influence healthcare professionals. Relationships with these leaders, once identified, were developed using programs that solicited their advice in marketing opioids, paid them to speak at events educating healthcare providers about opioids, and recruited them as authors of articles promoting opioid use.

Existing research has detailed the pharmaceutical industry's reliance on KOLs to promote its products.^9^^,^^36^ This research expands on that work with a specific focus on opioids, detailing how companies provided funding for research to support claims that opioids were not addictive, sought to reduce the concerns of providers about potential abuse, and trained KOLs to increase opioid prescribing. These activities are particularly concerning when directed to addictive medications that can harm health. Previous work on pharmaceutical marketing has highlighted how companies used professional organizations (e.g. the American Pain Society) and sales representatives,^31^ describing KOLs as part of the industry's sales force.^24^^,^^25^ The level of resources and effort dedicated to the identification and classification of KOLs for opioids mirrors that of the tobacco, alcohol, cannabis, and food industries.^14^^,^^15^^,^^34^

This study also highlights the importance of litigation in helping influence public discourse on opioids in medicine. Litigation involving pharmaceutical companies contained a variety of allegations including aggressive marketing to physicians and downplaying the risks of opioid use.^41^ There has been no direct litigation specifically focusing on pharmaceutical companies leveraging KOLs to influence public discourse centered around opioids in medicine.^37^ As a result, there is a risk of prescribers and prescribers in training being influenced by KOLs to practice medicine in ways that would benefit pharmaceutical companies financially and may ultimately present harm to patients.

### 4.1 Limitations

Industry document research is incomplete and may not be representative of all documents created by pharmaceutical companies to further market and sell prescription opioids. While these documents were considered noteworthy during the legal discovery process, they may not include other applicable information, particularly if it was never in writing form. Some documents were visual aids such as presentations that may not have been used. Furthermore, since the selection of available and related documents used in this study are scattered over a two-decade long time-period there is important contextual information that is missing to fully understand what transpired. Despite these limitations, our findings provide detailed information on how pharmaceutical companies identified, recruited, and used KOLs to market prescription opioids.

## 5 Conclusion

Our findings suggest that stronger conflict-of-interest policies are needed to limit the influence of the pharmaceutical industry in medicine. Beginning in at least the 2000s, pharmaceutical companies recruited and employed KOLs to leverage their reputations in ways that would encourage other healthcare providers to inappropriately prescribe more opioids than they otherwise would have, significantly contributing to the overdose epidemic. As a result, healthcare providers should be aware and critical of pharmaceutical company members and their practices to identify corporate profit-led biases and to weight unbiased evidence more favorably. The public health harms resulting from this behavior demonstrate the critical need to ensure that researchers and leaders in the medical field, particularly those involved in prescribing decisions related to medications with the potential for abuse and dependence, are free from conflicts of interest.

## Funding

This work was supported by the University of Nevada, Reno (UNR) (Crosbie) and US National Institutes of Health (NIH) grants R01 DA058687 (Apollonio) and R01 CA268491 (Apollonio). Neither UNR nor NIH played a role in the research or the preparation of this article.

## Ethics approval

Not applicable.

## CRediT authorship contribution statement

**Brian Gac:** Writing – review & editing, Writing – original draft, Visualization, Validation, Methodology, Investigation, Data curation, Conceptualization. **Kgosi Tavares:** Writing – review & editing, Writing – original draft, Validation, Investigation. **Hanna Yakubi:** Writing – review & editing, Validation, Methodology, Investigation, Data curation, Conceptualization. **Hannah Khan:** Writing – review & editing, Writing – original draft. **Dorie E. Apollonio:** Writing – review & editing, Validation, Supervision, Project administration, Methodology, Investigation, Conceptualization. **Eric Crosbie:** Writing – review & editing, Validation, Supervision, Project administration.

## Declaration of competing interest

No conflicts of interest to declare.

## Data Availability

The data underlying this article are publicly available as cited in the references.

## Appendix A Supplementary data

Supplementary material 1: Detailed notes from the Opioid Industry Documents Image 1

Supplementary material 2 Image 2

## References

[1] ACTIQ (2004). Directors Meeting ACTIQ 2 December. https://www.industrydocuments.ucsf.edu/docs/#id=yhgg0230.

[2] Argoff C. (June 2022). Open Payments Data. https://openpaymentsdata.cms.gov/physician/93628.

[3] Brookes L., Argoff C. (9 April 2006). Speaker Agreement. https://industrydocuments.ucsf.edu/docs/#id=gsgg0230.

[4] Caleb Alexander G., Mix L.A., Choudhury S. (2022). The opioid industry documents archive: a living digital repository. Am J Public Health.

[5] Candiotti K. (25 January 2014). Use of opioid analgesics in pain management. https://www.industrydocuments.ucsf.edu/docs/#id=llgg0230.

[6] Center for Disease Control and Prevention (5 April 2024). Understanding the Opioid Overdose Epidemic. https://www.cdc.gov/overdose-prevention/about/understanding-the-opioid-overdose-epidemic.html?CDC_AAref_Val=https://www.cdc.gov/opioids/basics/epidemic.html.

[7] Chisholm-Burns M.A., Spivey C.A., Sherwin E., Wheeler J., Hohmeier K. (2019). The opioid crisis: origins, trends, policies, and the roles of pharmacists. Am J Health-Syst Pharm.

[8] Chronic Pain Scientific Advisory Board (30 November 2001). Agenda for the Chronic Pain Scientific Advisory Board. https://www.industrydocuments.ucsf.edu/docs/#id=fggg0230.

[9] Coleman J., Katz E., Menzel H. (1966).

[10] Compton W.M., Jones C.M. (2019). Epidemiology of the U.S. opioid crisis: the importance of the vector. Ann N Y Acad Sci.

[11] Cornwell S. (15 January 2007). Email from Stephen Cornwell to Dennis Fitzgerald regarding the Master KOL listing. https://www.industrydocuments.ucsf.edu/docs/#id=gygg0230.

[12] Egilman D.S., Collins G., Falender J., Shembo N., Keegan C., Tohan S. (2019). The marketing of OxyContin(R): a cautionary tale. Indian J Med Ethics.

[13] Gac B., Yakubi H., Apollonio D.E. (22 June 2023). Issues arising from the study design, conduct, and promotion of clinical trials funded by opioid manufacturers: a review of internal pharmaceutical industry documents. https://escholarship.org/uc/item/2t17c5tx.

[14] Gardner M.N., Brandt A.M. (2006). “the doctors’ choice is America’s choice”: the physician in US cigarette advertisements, 1930-1953. Am J Public Health.

[15] Grundy Q., Imahori D., Mahajan S. (2023). Cannabis companies and the sponsorship of scientific research: a cross-sectional Canadian case study. PloS One.

[16] Hodge J.G., Gostin L.O. (2019). Guiding industry settlements of opioid litigation. Am J Drug Alcohol Abuse.

[17] Infeld M., Bell A., Marlin C., Waterhouse S., Uliassi N., Fugh-Berman A. (2019). Continuing medical education and the Marketing of Fentanyl for breakthrough pain: marketing messages in an industry-funded CME module on breakthrough pain. World Med Health Policy.

[18] Janssen Pharmaceutica (25 January 2019). Payment history by J and J or Janssen to specified invidiuals - based upon deposition or Kimberly Deem-Eshleman. https://www.industrydocuments.ucsf.edu/docs/#id=rngg0230.

[19] Janssen Pharmaceutica (2004). https://www.industrydocuments.ucsf.edu/docs/#id=fxgg0230.

[20] Janssen Pharmaceutical Inc (July 2012). Nucynta Speaker Training. https://www.industrydocuments.ucsf.edu/docs/#id=yrgg0230.

[21] Jones J. (21 August 2020). United States of America v. the Purdue Frederick Company, Inc, et al (The Western District of Virginia Abingdon Division 2007). http://www.vawd.uscourts.gov/OPINIONS/JONES/107CR00029.PDF.

[22] Lazarsfeld P., Berelson B., Gaudet H. (1948).

[23] Lee C., Tsui A., Xu S., Apollonio D.E. (2024). Evaluation of the strategies opioid manufacturers used to recruit health professionals and encourage overprescribing: an analysis of industry documents. BMC Public Health.

[24] Meffert J.J. (2009). Key opinion leaders: where they come from and how that affects the drugs you prescribe. Dermatol Ther.

[25] Moynihan R. (2008). Key opinion leaders: independent experts or drug representatives in disguise?. BMJ.

[26] Neumann C., Toscani M. (4 February 2005). Key Opinion Leader (KOL) Development Plan for Cephalon Pain Franchise. https://www.industrydocuments.ucsf.edu/docs/#id=hygg0230.

[27] Oklahoma Opioid Litigation Documents (10 March 2020). Collections - Drug Industry Documents. https://www.industrydocuments.ucsf.edu/drug/collections/oklahoma-opioid-litigation-documents/.

[28] Ortho McNell Janssen Pharmaceuticals Inc (25 July 2008). NEO pathways: Heading in new directions in pain management. https://www.industrydocuments.ucsf.edu/docs/#id=zngg0230.

[29] Ostling P.S., Davidson K.S., Anyama B.O., Helander E.M., Wyche M.Q., Kaye A.D. (2018). America’s opioid epidemic: a comprehensive review and look into the rising crisis. Curr Pain Headache Rep.

[30] Pharma Marketing Network (April 2024). The Pharma Marketing Glossary: Key Opinion Leader (KOL). https://web.archive.org/web/20240224070820/https://www.pharma-mkting.com/glossary/?dir=2&name_directory_startswith=K.

[31] Podolsky S.H., Herzberg D., Greene J.A. (2019). Preying on prescribers (and their patients) - pharmaceutical marketing, iatrogenic epidemics, and the Sackler legacy. N Engl J Med.

[32] Psaty B.M., Merrill J.O. (2017). Addressing the opioid epidemic - opportunities in the Postmarketing setting. N Engl J Med.

[33] Purdue Pharma L.P. (21 January 2011). Corporate Reputation & Visibility Strategic Plan. https://www.industrydocuments.ucsf.edu/docs/#id=rkgg0230.

[34] Scher J.U., Schett G. (2021). Key opinion leaders - a critical perspective. Nat Rev Rheumatol.

[35] Schwartz L.M., Woloshin S. (2019). Medical Marketing in the United States, 1997-2016. JAMA.

[36] Sismondo S. (2015). How to make opinion leaders and influence people. CMAJ.

[37] Spencer A. Email, from S. (14 December 2018). Amy Spencer to Linda Singer with Attached Copy of Declaration of Russell K. Portenoy, M.D. https://www.industrydocuments.ucsf.edu/opioids/docs/#id=gjpw0232.

[38] Teva (28 March 2013). Teva Advocacy Mapping: Identifying Advocacy Partners to Enhance Patient Care. https://www.industrydocuments.ucsf.edu/docs/#id=lngg0230.

[39] Thomson H. (18 February 2002). Email from Healther Thomson to Michele Cole regarding KOL categorization. https://www.industrydocuments.ucsf.edu/docs/#id=lhgg0230.

[40] United States Department of Justice (13 May 2004). Warner-Lambert to pay $430 million to resolve criminal & civil health care liability relating to off-label promotion. https://www.justice.gov/archive/opa/pr/2004/May/04_civ_322.htm.

[41] University of California San Francisco (UCSF) (January 2024). Opioid Industry Doucments: National Prescription Opiate Litigation Documents. https://www.industrydocuments.ucsf.edu/opioids/collections/national-prescription-opiate-litigation-documents/.

[42] Van Zee A. (2009). The promotion and marketing of oxycontin: commercial triumph, public health tragedy. Am J Public Health.

[43] Wilcock M. (2020). Pharmaceutical marketing-greater than the sum of its parts?. Drug Ther Bull.

[44] Yakubi H., Gac B., Apollonio D.E. (2022). Industry strategies to market opioids to children and women in the USA: a content analysis of internal industry documents from 1999 to 2017 released in state of Oklahoma v. Purdue Pharma, L.P. et al. BMJ Open.

