# Supplementary material for: Pharmaceutical industry use of key opinion leaders to market prescription opioids: A review of internal industry documents
Source: Explor Res Clin Soc Pharm. 2024 Nov 19;16:100543. doi: 10.1016/j.rcsop.2024.100543 (PMC11647219; doi:10.1016/j.rcsop.2024.100543)
Supplement: Supplementary file 1 — Supplementary material 1: Detailed notes from the Opioid Industry Documents [file mmc1.docx]

**Topics**

- **Legal filings [S1.0]**
- **Vulnerable populations**
  - **Children**
  - **Military**
  - **Elderly**
- **Academic Malfeasance**
  - **Ghostwriting**
  - **Enriched Enrollment**
  - **Research Laundering**
  - **Other**
- **Other relevant topics**
  - **Advocacy Groups**
  - **Key Opinion Leaders**
  - **Continuing Education**

**LEGAL FILINGS**

1. Original Petition in State of Oklahoma vs Purdue Pharma, et al.
   - [https://www.industrydocuments.ucsf.edu/docs/mshg0230](https://www.industrydocuments.ucsf.edu/drug/docs/#id=mshg0230)
   - Author : District Court of Cleveland County, State of Oklahoma
   - Document Date : 2017 June 30
   - Type : legal
   - ID : mshg0230 ( TID : wyf71j00 )
   - ARK : ark:/88122/mshg0230
   - Collection : Oklahoma Opioid Litigation Documents; Opioid Documents Collection
   - Key Points:
     1. INTRODUCTION
        1. “To convince medical professionals to prescribe more opioids to a broader range of patients, Defendants elected to falsely downplay the risk of opioid addiction and overstate the efficacy of opioids for more wide-ranging conditions, including chronic non-cancer pain.”
        2. “To encourage physicians to prescribe more opioids, Defendants event went so far as to tell prescribers that classic signs of addiction should actually be treated with ***more*** opioid use because they were signs of "pseudoaddiction" which meant the patient was supposedly experiencing undertreated pain.”
     2. FACTUAL ALLEGATIONS
        1. Defendants Falsely and Deceptively Marketed Their Opioids in Oklahoma
           1. “Defendants caused catastrophic damage to the State of Oklahoma by dramatically altering the perception of opioids by doctors and patients alike. Prior to Defendants' deceptive marketing campaign, the medical community and consumers primarily relied on opioids for limited purposes, such as surgery recovery, cancer treatment, and end-of-life palliative care. This was largely due to the risk of addiction and abuse posed by these powerful drugs. Defendants sought to change that perception in two key ways. First, Defendants misrepresented the risks of addiction and abuse from opioids. Defendants falsely represented that the risks of addiction were overstated and that scientific studies supported a low risk of addiction associated with their drugs. Second, Defendants touted unsubstantiated benefits of opioid treatment, including its effectiveness in treating chronic non-cancer related pain. Defendants repeated these misrepresentations to physicians and consumers throughout the country, including directly to physicians and consumers in Oklahoma. At times, Defendants specifically targeted vulnerable patient populations. Each Defendant employed massive and unprecedented marketing campaigns premised on these two key misrepresentations.”
           2. “Defendants could not work this scheme without providing some "scientific" support for their statements. Defendants did this by operating through "Key Opinion Leaders" or "KOLs" and third-party groups to further spread their misrepresentations about opioids.”
           3. “KOLs are doctors who act as consultants or advisors to Defendants and through whom Defendants tout their misrepresentations regarding the risk of addiction and benefits of opioids. Defendants paid KOLs to give speeches, talks, and speak at continuing medical education seminars (CMEs) about opioids, advocating that they could be used effectively to treat things like chronic pain and downplaying the risks of addiction and abuse. By operating through KOLs, Defendants added perceived legitimacy and/or impartiality to their misrepresentations regarding opioids.”
           4. “Defendants operated through many of the same KOLs including Dr. Russell Portenoy and Dr. Lynn Webster.”
           5. “Defendants Funded Seemingly Third-Party Groups to Spread Their False Marketing Even Further and Give Their Statements False Credibility”
           6. “In addition to KOLs, Defendants relied on seemingly unaffiliated and impartial organizations to promote opioid use. Defendants utilized and funded these organizations to spread their misrepresentations by downplaying the risks of addiction of opioids and the benefits of use for conditions like chronic pain. Defendants funded, directed, and controlled several such organizations, and certain of Defendants' KOLs also served in various roles for these organizations, including as board members and officers.”
           7. “For example, the American Pain Foundation (the "APF") was one of the more prominent "pain advocacy" organizations Defendants utilized to spread their misrepresentations. While APF purported to be an independent organization, it obtained much of its funding from pharmaceutical companies such as Defendants. In 2010, the APF reportedly obtained almost 90% of its $5 million funding from drug and medical device companies including certain Defendants such as Purdue. Defendants, through the APF, created treatment guides and other materials for patients and others that downplayed the addiction risks of opioids and exaggerated their benefits. Defendants, through the APF, also specifically promoted opioid use among veterans. APF made these materials available nationwide, including in Oklahoma. These guides were funded by Defendants to spread their misrepresentations further and add perceived legitimacy and impartiality.”
           8. “Another supposedly unaffiliated and impartial group Defendants utilized was the American Academy of Pain Medicine ("AAPM"). The AAPM claimed addiction risk of opioid treatment was low when used to treat people in pain.”
           9. “The list of groups Defendants funded and utilized to spread their misrepresentations is long. Indeed, Defendants have been tied to at least the following groups that distributed pro-opioid messages for Defendants with the same misrepresentations regarding the risk of addiction and benefits: the American Pain Society; American Geriatrics Society, American Chronic Pain Association, American Society of Pain Education, National Pain Foundation, and Pain & Policy Studies Group. Defendants used groups like those listed above to spread their misrepresentations about the risk of addiction of opioids and their benefits.”
           10. “The nature of Defendants' marketing scheme required Defendants to conceal the truth for it to be effective. Thus, Defendants operated from behind the scenes, spreading their deceptive misrepresentations through KOLs and third-party groups to conceal their own involvement. Defendants also concealed the falsity of their misrepresentations regarding addiction risk and the benefits of long-term opioid treatment. As such, while the opioid epidemic spread, Defendants' role and responsibility remained concealed. The State could not have acquired such knowledge through the exercise of reasonable diligence.”
2. The Drug Addiction and Opioid Crisis
   - [https://www.industrydocuments.ucsf.edu/docs/npgg0230](https://www.industrydocuments.ucsf.edu/drug/docs/#id=npgg0230)
   - Author : Christie, Chris; Kennedy, Patrick J; Madras, Bertha; Bondi, Pam; Cooper, Roy; Baum, Richard J
   - Document Date : 2017 November 01
   - Type : letter; report
   - ID : npgg0230 ( TID : fnf71j00 )
   - ARK : ark:/88122/npgg0230
   - Collection : Oklahoma Opioid Litigation Documents; Opioid Documents Collection
   - Key Points:
     1. 2017 Report from multiple governors on the status of the opioid epidemic
3. CDC Guideline for Prescribing Opioids for Chronic Pain – United States, 2016
   - [https://www.industrydocuments.ucsf.edu/docs/zkfg0230](https://www.industrydocuments.ucsf.edu/drug/docs/#id=zkfg0230)
   - Author : Center for Disease Control and Prevention; US Department of Health and Human Services
   - Document Date : 2016 March 18
   - Type : report, scientific
   - ID : zkfg0230 ( TID : oaf71j00 )
   - ARK : ark:/88122/zkfg0230
   - Collection : Oklahoma Opioid Litigation Documents; Opioid Documents Collection
   - Key Points:
     1. “This guideline provides recommendations for primary care clinicians who are prescribing opioids for chronic pain outside of active cancer treatment, palliative care, and end-of-life care. The guideline addresses 1) when to initiate or continue opioids for chronic pain; 2) opioid selection, dosage, duration, follow-up, and discontinuation; and 3) assessing risk and addressing harms of opioid use. CDC developed the guideline using the Grading of Recommendations Assessment, Development, and Evaluation (GRADE) framework, and recommendations are made on the basis of a systematic review of the scientific evidence while considering benefits and harms, values and preferences, and resource allocation. CDC obtained input from experts, stakeholders, the public, peer reviewers, and a federally chartered advisory committee. It is important that patients receive appropriate pain treatment with careful consideration of the benefits and risks of treatment options. This guideline is intended to improve communication between clinicians and patients about the risks and benefits of opioid therapy for chronic pain, improve the safety and effectiveness of pain treatment, and reduce the risks associated with long-term opioid therapy, including opioid use disorder, overdose, and death. CDC has provided a checklist for prescribing opioids for chronic pain (http://stacks.cdc.gov/view/cdc/38025) as well as a website (http://www.cdc.gov/drugoverdose/prescribingresources.html) with additional tools to guide clinicians in implementing the recommendations.”
4. Increase in Unintentional Medication Overdose Deaths
   - [https://www.industrydocuments.ucsf.edu/docs/lpgg0230](https://www.industrydocuments.ucsf.edu/drug/docs/#id=lpgg0230)
   - Author : Piercefield, Emily; Archer, Pam; Kemp, Philip; Mallonee, Sue
   - Document Date : Unknown
   - Type : article; graph; report; report, scientific; table
   - ID : lpgg0230 ( TID : dnf71j00 )
   - ARK : ark:/88122/lpgg0230
   - Collection : Oklahoma Opioid Litigation Documents; Opioid Documents Collection
   - Key Points:
     1. Funded by the Oklahoma State Department of Health and the Oklahoma Office of the Chief Medical Examiner
5. The Prescription Drug Overdose Epidemic
   - [https://www.industrydocuments.ucsf.edu/docs/mxgg0230](https://www.industrydocuments.ucsf.edu/drug/docs/#id=mxgg0230)
   - Author : Paulozzi, Len; National Center for Injury Prevention and Control; Centers for Disease Control and Prevention; USA Department of Health and Human Services
   - Document Date : Unknown
   - Type : graph; report, scientific
   - ID : mxgg0230 ( TID : wif71j00 )
   - ARK : ark:/88122/mxgg0230
   - Collection : Oklahoma Opioid Litigation Documents; Opioid Documents Collection
   - Key Points:
     1. “The Prescription Drug Overdose Epidemic and the Role of PDMPs in Stopping It”
     2. “Early surveillance data failed to detect a problem: DAWN 1990-1996, (Joranson, 2000)
     3. “Perhaps because some data was left out: DAWN 1990-1998 data available when Joranson et al (2000) published”
6. Checklist for Prescribing Opioids for Chronic Pain
   - [https://www.industrydocuments.ucsf.edu/docs/skfg0230](https://www.industrydocuments.ucsf.edu/drug/docs/#id=skfg0230)
   - Author : US Department of Health and Human Services; Centers for Disease Control and Prevention
   - Document Date : Unknown
   - Type : list
   - ID : skfg0230 ( TID : maf71j00 )
   - ARK : ark:/88122/skfg0230
   - Collection : Oklahoma Opioid Litigation Documents; Opioid Documents Collection
7. Opioid Overdose Fast Facts
   - [https://www.industrydocuments.ucsf.edu/docs/ytgg0230](https://www.industrydocuments.ucsf.edu/drug/docs/#id=ytgg0230)
   - Author : Oklahoma State Department of Health
   - Document Date : Unknown
   - Type : newsletter
   - ID : ytgg0230 ( TID : spf71j00 )
   - ARK : ark:/88122/ytgg0230
   - Collection : Oklahoma Opioid Litigation Documents; Opioid Documents Collection
8. Reducing Prescription Drug Abuse in Oklahoma
   - [https://www.industrydocuments.ucsf.edu/docs/zghg0230](https://www.industrydocuments.ucsf.edu/drug/docs/#id=zghg0230)
   - Author : Fallin, Mary; Oklahoma Department of Mental Health and Substance Abuse Services
   - Document Date : Unknown
   - Type : graph; photograph; publication
   - ID : zghg0230 ( TID : urf71j00 )
   - ARK : ark:/88122/zghg0230
   - Collection : Oklahoma Opioid Litigation Documents; Opioid Documents Collection
9. Oklahoma Opioid Prescribing Guidelines
   - [https://www.industrydocuments.ucsf.edu/docs/zjfg0230](https://www.industrydocuments.ucsf.edu/drug/docs/#id=zjfg0230)
   - Author : Oklahoma State Department of Health; Injury Prevention Service
   - Document Date : Unknown
   - Type : report, scientific
   - ID : zjfg0230 ( TID : yze71j00 )
   - ARK : ark:/88122/zjfg0230
   - Collection : Oklahoma Opioid Litigation Documents; Opioid Documents Collection
10. OxyContin Abuse and Diversion and Efforts to Address the Problem
    - [https://www.industrydocuments.ucsf.edu/docs/tlgg0230](https://www.industrydocuments.ucsf.edu/drug/docs/#id=tlgg0230)
    - Author : United States General Accounting Office; Department of Health and Human Services; Food and Drug Administration; McClellan, Mark B; Guevara, Rogelio
    - Document Date : 2003 November 05
    - Type : graph; letter; publication; report; table
    - ID : tlgg0230 ( TID : zkf71j00 )
    - ARK : ark:/88122/tlgg0230
    - Collection : Oklahoma Opioid Litigation Documents; Opioid Documents Collection

**CHILDREN**

81) 2013 National Advocacy Business Planning

Document Data

- Author : Johnson and Johnson; Janssen Pharmaceutical
- Document Date : 2012 June 29
- Type : proposal
- ID : zggg0230 ( TID : yhf71j00 )
- ARK : ark:/88122/zggg0230

Document Notes

- Business planning from Janssen (PAIN FRANCHISE) June 29, 2012
- Key Questions = How to leverage sales of Nucynta??
- 2013 PAIN Advocacy Strategy
  - Engage partners to embrace the IOM report-national/state implications advocate for and act collectively to actualize the recommendations
  - **Influence agencies that impact policy and quality to maintain or improve access**
- Barriers to care
  - FOCUS: **Engage with advocacy partners** at the national level for greater impact and alignment. Support models for collaboration and synergies that can then be applied at a regional level
  - Chronic pain can and should be thought of as a disease in and of itself.
  - Public health and community-based approaches are required to **address the under-treatment of chronic pain**
- Advocacy/Policy Focus
  - Provide state and federal legislators and other regulatory groups with access to objective materials to assist them in making public policy.
  - Collaborate with the **Pain Care Forum (PCF)** on policy issues and common strategies with key decision makers; such as HHS, Surgeon General's Office, CDC, state and federal legislators and regulators. •
  - Collaborate with State Pain Policy Action Coalition (SPPAC), a newly formed organization made up of pain focused organizations that will pro-actively inform and influence state policy . •
  - **Sponsor Public Awareness campaigns targeted at preventing chronic pain and misuse of prescription pain medications. •**
  - **Sponsor disease awareness to promote balanced and effective pain management. •**
  - Support collaboration between Medicine and Law Enforcement to prevent the "chilling effect".
    - [Chilling effect](https://oxfordmedicine.com/view/10.1093/med/9780190659721.001.0001/med-9780190659721-chapter-10) ([link](https://oxfordmedicine.com/view/10.1093/med/9780190659721.001.0001/med-9780190659721-chapter-10)) = An overzealous Drug Enforcement Administration is sometimes prosecuting the wrong physicians, thus creating a chilling effect in the medical community with regard to opioid prescription and making it harder for people in pain to get the help they need
  - Support effective Prescription Monitoring Programs (PMPs) that provide prescribing healthcare professionals with "real time access" and improve patient care.

91) Email from Kohn Robyn to Penney Cowan Concerning Growing Pains

Document Data

- **Author :** Kohn, Robyn
- **Document Date :** 2011 December 12
- **Type :** email
- **ID :** nmgg0230 ( TID : jlf71j00 )
- **ARK :** ark:/88122/nmgg0230
- **Collection :** Oklahoma Opioid Litigation Documents; Opioid Documents Collection

Document Notes

- Penney Cowan = Founder & Executive director of American Chronic Pain Association
- **Growing Pains (ACPA program)** = <https://www.theacpa.org/conditions-treatments/conditions-a-z/growing-pains/> (link accessed 4/17/2020 at 6pm)
  - The American Chronic Pain Association (ACPA) is pleased to announce the launch of [Growing Pains](http://growingpains.org/), a new social networking site for young people with pain. Growing Pains is here to help young people connect with their peers who understand the effect pain has on their lives. Users can create journals, join discussions, and learn ways to manage pain
  - Growing Pains is a support group for chronically ill youth. The members communicate through email and snail mail. Through communication, kids will develop a greater self-awareness, a sense of connection with their peers whose experiences are similar, and the ability to express how they are feeling. As a teenager, it is essential to love yourself and be proud of who you are.
  - Adults are not the only ones afflicted with chronic pain. There are many young people struggling to balance the obstacles of growing up with the ache of chronic illness. It is time for teenagers in pain to realize how many of their peers understand. Communication and education are the key to accepting the obstacles life presents, and the beginning of a happy life
- This represents excellent work and a significant contribution to the understanding of pain among the child and adolescent populations. **I would like to respond and suggest a change to our new legal entity Janssen Pharmaceuticals. Inc.** We can certainly forward the logo as appropriate. Many thanks for your continued support and passion for our children. Warmest regards, Robyn

92) Letter from Robyn Kohn to Tricia Haertlein enclosing Meeting 2

Document Data

- **Author :** Kohn, Robyn
- **Document Date :** 2011 November 01
- **Type :** email; presentation; slides; report
- **ID : mmgg0230**

Document Notes

- Meeting of **Imagine the Possibilities: Pain Coalition**
- 3 subteams charged with creating message
  - MEDIA OUTREACH – Targets = **YOUTH, VETERANS**, PUBLIC
  - POLICY/ADVOCACY
    - Peer-reviewed publication in health policy journal
- Messaging: **Chronic pain as public health problem, chronic pain is multi-contextual**
- Advocacy + Pain Policy Sub-Team Platform
  - **Chronic pain is the #1 public health problem**
  - **Epidemic of Pain v Epidemic of Addiction**
  - **Wide dissemination of plan**
    - Advocacy magazines – AAPM Currents, AAFP Live, AAN Neurology Today
    - General public – Op-eds, popular media, retail chains (**Starbucks pain message of the day????)**
- Education Sub-Team Platform
  - Major needs
    - Placard for HCP so they keep pain conversation front of mind (should fit in lab coat)
    - Quick Guide geared toward community-based pharmacists
    - Medical school curriculum development grant programs
- MEdia Outreach Initiatives
  - Reaching out to: **YOUTH**
    - Reach early – elementary school level – via respoected channels (coaches)
    - Delivery a practical message: **pain is your body telling you something important**
  - Reaching out to: **RETURNING VETERANS**
    - Where do they get their info about pain?
    - Change the paradaign: **the positive side of pain management**
  - Reaching out to: **MEDIA**
    - Capture venues that the media want to cover: art cretaed by people in pain
    - Emphazie the problem of **poorly managed pain is often lost to the topic of addiction fear**; even though it [poorly managed pain] is an issue of many magniutes greater concern [than addiction]
- Teams and Members
  - **MEDIA OUTREACH = “destigmatize pain”**
    - **Jack Henningfield**
    - **Patricia Cosler**
    - **Apriler Vallerand**
    - **Pam Galassini**
    - ^^^**These are all authors of the Military & Chronic Pain paper!!**
  - **PUBLIC POLICY = “change the conversation about pain”**
    - **Bob Twillman**
    - Robyn Kohn
  - **EDUCATION = “deliver the education”**
    - Art Caplan
    - Myra Glajchen
    - Gary Baker
    - Richard Payne
    - Penny Cowan

104) Pain Brief Advocacy & Policy Monthly 2011

Document Data

- **Author :** Kohn, Robyn
- **Document Date :** 2011 July 07
- **Type :** email; report
- **ID :** tygg0230 ( TID : vmf71j00 )
- **ARK :** ark:/88122/tygg0230
- **Collection :** Oklahoma Opioid Litigation Documents; Opioid Documents Collection

Document Notes

- SEE # 103 – this is an example of the Policy Monthly Briefing proposed by Kohn
- Primary External Partners
  - GO TO PARTNER = **American Pain Foundation**
  - American Academy of Pain Medicine
  - American Academy of Pain Management
  - American Chronic Pain Association
- Secondary External Partners
  - American Academy of Hospice & Palliative Care Medicine
  - American Academy of Family Physicians
  - American Geriatric Society
  - American Society of Consultant Pharmacists
  - American Academy of Physician Assistants
  - American Academy of Nurse Practitioners
- **KEY ADVOCACY THEMES**
  - Marketplace
    - **Significant unmet needs in moderate-severe chronic pain market**
    - Advocacy on the local market level gaining momentum among partner org
  - Pain Tools
    - **Prescribe Responsibly**-external HCP community
    - **Smart Moves, Smart Choices**-Teen RX Abuse
    - **Let's Talk Pain**-Provider/Patient Communications
- **NEW INITIATIVES**
  - **Imagine the Possibilities – Pain Coalition**
    - Internal cross-functional members of the pain teams and external members of the pain communities
    - Goal: align and address issues in pain management with emphasis on abuse and diversion
  - **New Partnership: State Pain Policy Action Coalition (SPPAC)-AAPM, APF, ASPMN**
    - Goal: Formed out of need to respond and influence positive state-based public policies is crucial to assuring access to effective care for persons experiencing pain.
    - **National pain management organizations have attempted to influence** many of these issues, but often have done so in an ineffective piecemeal and hit-or-miss fashion, failing to take advantage of opportunities to **speak with one voice and achieve desired outcomes.** SPPAC provides the opportunity to create a synergistic voice to insure the ability of our members to provide and receive optimal care for pain.
    - While three organizations are initially organizing the coalition, other organizations will be invited to join once the structure is developed.
- IOM REPORT (6/29/11): **Reliving Pain in America**
  - What is the IOM report: The report, released on June 30, 2011 — Relieving Pain in America: A Blueprint for Transforming Prevention, Care, Education, and Research — calls for a cultural transformation of attitudes toward pain and its prevention and management.
  - Noteworthy findings in IOM
    - Chronic pain affects an estimated **116 million** American adults
    - Pain costs the nation up to **$635 billion each year** in medical treatments and lost productivity.
    - Chronic pain negatively affects socioeconomic status.
    - Federal and state drug abuse prevention laws, regulations, and enforcement practices have been considered impediments to effective pain management...." Among other barriers, they say "**Twentynine percent of primary care physicians and 16 percent of pain specialists report they prescribe opioids less often than they think appropriate because of concerns about regulatory repercussions."**
  - Partner Commentary
    - **Pain Care Task Force** is being formed to develop a communication and outreach plan for dissemination of key messaging across national/local markets. The activities can serve as an advocacy tool for our partner organizations.
    - The committee calls for government agencies, healthcare providers, and public and private funders of health care to adopt a comprehensive, strategic approach to reduce or eliminate the barriers to pain care.

105) Prescriptive Authority Slide

Document Data

- **Author :** Janssen; Johnson and Johnson; Kohn, Robyn; Health Care Systems, Inc
- **Document Date :** 2011 July 05
- **Type :** email; report
- **ID :** gpgg0230 ( TID : ymf71j00 )
- **ARK :** ark:/88122/gpgg0230
- **Collection :** Oklahoma Opioid Litigation Documents; Opioid Documents Collection

Document Notes

- Email from Robyn Kohn (National Advocacy Director) -- **POWERPOINT: “2011-2012 Advocacy Launch Plan”**
- Critical Launch Success Factors
  - Continue to drive national and regional access
  - Understand & influence policy and legislative events to ensure appropriate patient access
  - **Collaborate with key patient advocacy organizations to advance awareness of under -treatment and under-management of pain**
- National Advocacy: Key Launch Components
  - Pain Patient Foundations: **APF, ACPA**
  - Provider Organizations: **APS, AAPM (medicine), AAPM (management)**
- National Advocacy: Local Advocacy
  - Patient Foundation: **APF Action Network, ASPI**
  - Provider Org: **APS, AAPM**
  - Individal KAL: **COEs, Medical/Professional Pain & Family Practice organizations**
- Recognizing pain as own disease state
  - Stigma of Pain
  - Validate, diagnosis, treatment, management – **UNDER TREATMENT OF PAIN**
- **Big problem = LIMITED ACCESS TO PRESCRIPTIVE AUTHORITY**
- Advocacy Support: National & Local needed for balanced pain policy position statements
  - Goals for Advocacy Support
    - **Remove barriers to access for pain treatment**
    - Educate partners and stakeholders on appropriate opioid use
  - **“We support unrestricted access to scheduled pain medications that are being used appropriately for the treatment of moderate to severe pain”**
  - **“We support intervention by the appropriate authorities to address the misuse, abuse, and diversion of scheduled pain medications”**
- Tier 1 Pain Organization: **American Pain Society, American Academy of Pain Medicine, American Pain Foundation/Action Network, COE**
- Goal = Coordinate/Collaborated network prepared to mobilize when issues on the state level need Advocacy Support from our:
  - **Pain Organizations**
  - **Professional Organizations**
  - **Patient Organizations**
  - **Medical & Health Care Organization**
- Pain state issues
  - States are moving quickly to address RX drug abuse – emphasis on opiates
  - Growing importance of role of national state organizations
  - Concerns about punitive policy that discourages appropriate pain management
- **CASE STUDIES – Examples of Successes**
  - PhRMA supports/actively lobbies PMP for first time (3/11) in Georgia
    - Law enacted w/ many of our desired provisions
    - **Defeated 60 unit per RX limited**
    - **Defeated wholesaler reporting requirement**
    - **Includes balanced pain management language**
  - FL Pill Mill Enacted with PMP
    - Secured inclusion of PMP
    - Favorable amendment added: wholesaler 5000 monthly pill limit removed
  - Defeated ND Worker’s Comp Restrictive Opiate Protoco
    - Bill would have made it extremely difficult to prescribe opiates for pain
    - Close collaboration between GA&P (Government affairs & policy) and SCG/Advocacy resulted in success
- Landmark Advocacy reports Released by Obama Admin
  - NIH/IOM Report: State of pain – *validation of unmet need*
- **UNBRANDED INITIATIVES** -- a term I am seeing a lot in the documents
  - Unbranded Initiatives commonly mentioned in Jansen internal memos ([link](https://www.multivu.com/assets/51908/documents/51164-Janssen-Responsibility-Fact-Sheet-original.pdf))
    - **Let’s Talk Pain** -- TOOL FOR PATIENTS
      - Let’s Talk Pain is a coalition that encourages people with pain and their healthcare professionals to talk more about pain, to listen actively, and to act in ways that improve care for people with acute or chronic pain. The Let’s Talk Pain Coalition is a collaboration between the American Pain Foundation, the American Academy of Pain Management, the American Society for Pain Management Nursing, and Janssen Pharmaceuticals, Inc., which also sponsors the Coalition. LetsTalkPain.org features resources and interactive tools to help encourage an open dialogue about pain. These include tips to help patients talk with their healthcare professional, tips to help healthcare professionals talk to their patients, and several videos providing patient and healthcare professional perspectives, including two online video series, the “Let’s Talk Pain Show” and a medication safety series.
    - **Prescriber Responsibly** – TOOL FOR PHYSICIANS
      - Prescribe Responsibly is a website for healthcare professionals about the appropriate and responsible prescribing of opioid pain therapies for patients with acute and chronic pain. Physicians cite a number of concerns when prescribing opioid analgesics to people with pain, including risks of diversion and misuse.1 These concerns can present barriers to appropriate pain management, and may lead to the under-treatment of pain.2 Prescribe Responsibly addresses concerns physicians may have when prescribing opioid analgesics to patients with pain. PrescribeResposibly.com covers a range of topics related to the appropriate prescribing of opioid analgesics and offers case studies to help healthcare professionals assess various scenarios they may encounter when caring for patients with pain. Prescribe Responsibly also provides links to valuable tools that can help healthcare professionals assess pain levels as well as the risks for aberrant drug-related behavior
    - **Smart Moves, Smart Choices** – TOOL FOR TEENS
      - The National Association of School Nurses and Janssen Pharmaceuticals teamed up to create Smart Moves, Smart Choices, a national awareness program that informs parents, teens and educators about teen prescription drug abuse and its serious risks. SmartMovesSmartChoices.org features a wealth of resources, including myth-busting facts, tips for parents, an interactive quiz for teens, lesson plans and a school assembly tool kit for educators, and videos featuring addiction medicine specialist and TV host Dr. Drew Pinsky in conversations with teens, parents and educators.
  - “UI” appears in every email by Kohn (with the same 3 programs listed)
  - **More info on** [**UI**](https://www.pm360online.com/the-boldness-of-an-unbranded-campaign/)
    - when an unbranded campaign vigorously hones in on a specific unmet need that can ultimately direct HCPs to your brand—it is essential. It is worth its weight in gold, and it will indelibly illuminate the reasons to prescribe your product.
    - How can we be sure that an unbranded campaign will ultimately translate into greater prescription volume when we never even mention the product’s name?
    - Which is why an unbranded campaign can deliver to HCPs the type of information that will challenge them to overcome clinical inertia, reconsider their accepted protocols, and deviate from the status quo
    - Above all, an unbranded campaign must highlight a crisply defined unmet need that your product will in some way address
    - Goals of unbranded campaign
      - 1) Drive diagnosis through Disease Awareness
      - 2) Highlight the Pathophysiology & Burden of a Specific Disease
      - 3) Enhance Corporate Reputation


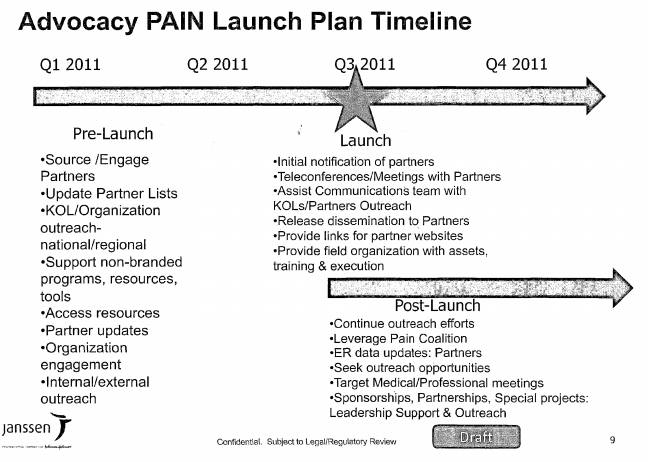


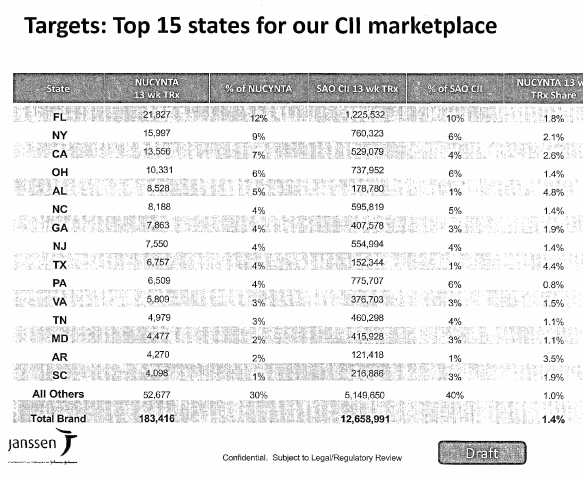


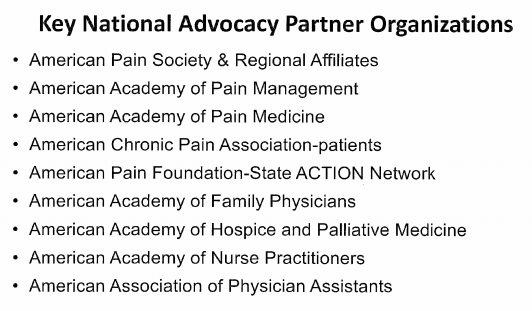


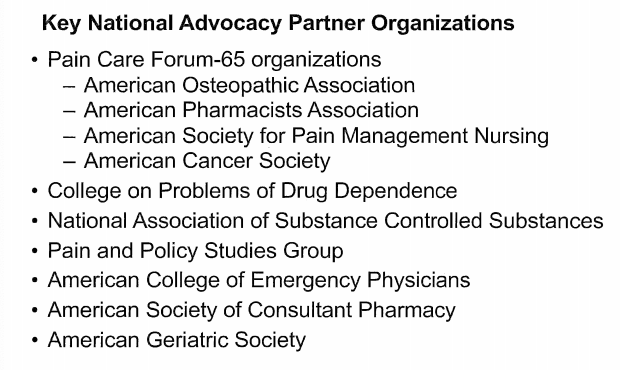


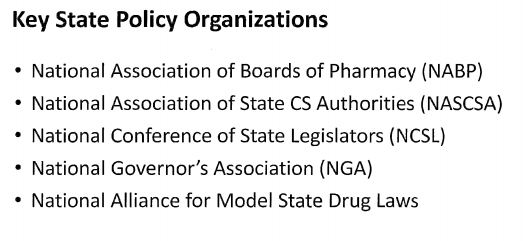


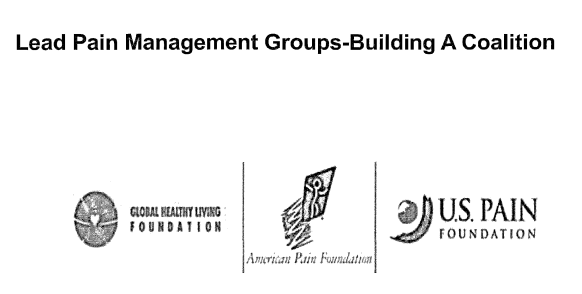


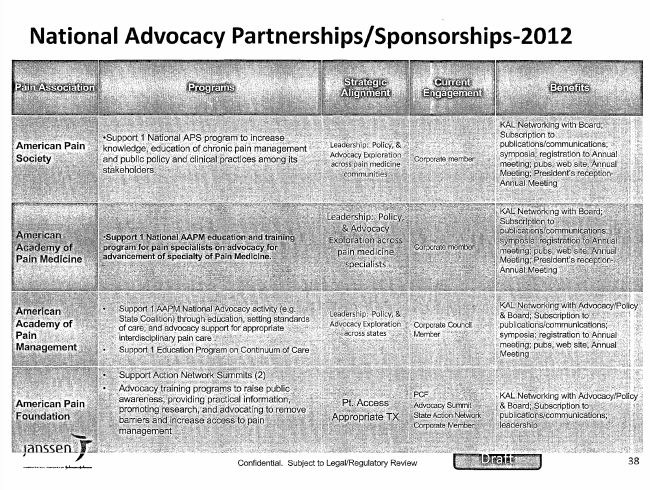


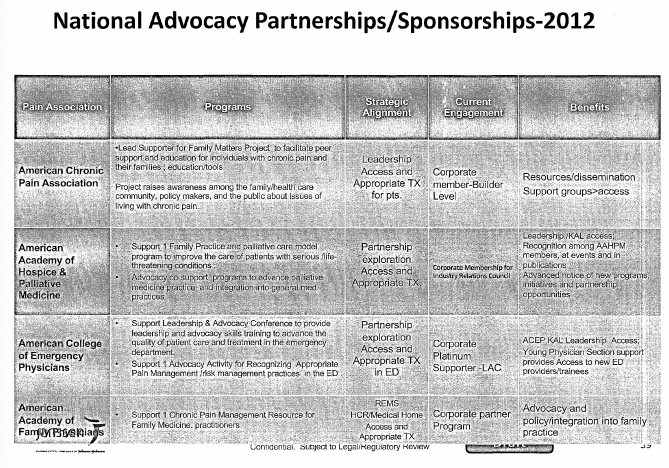


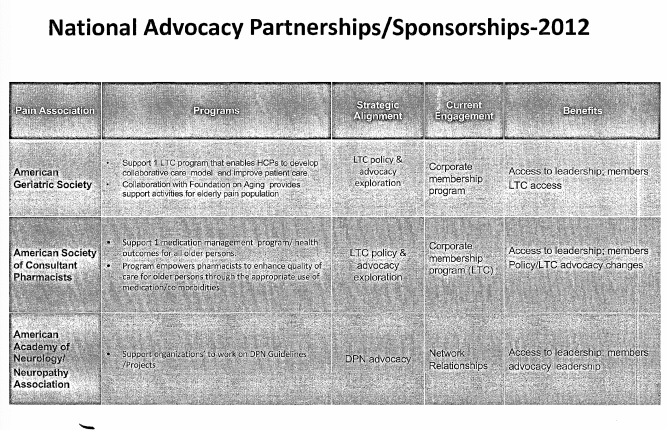


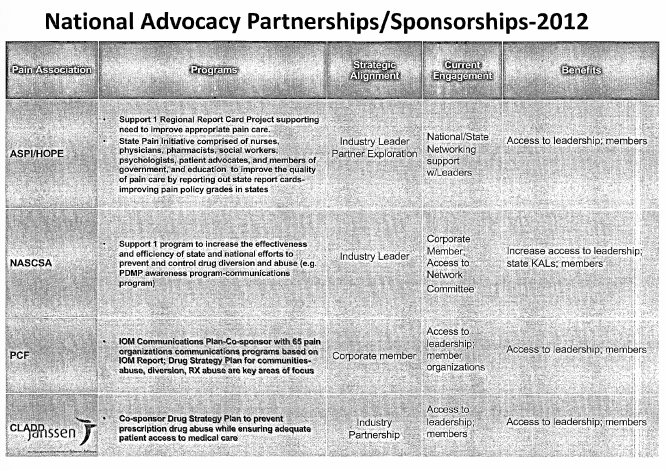


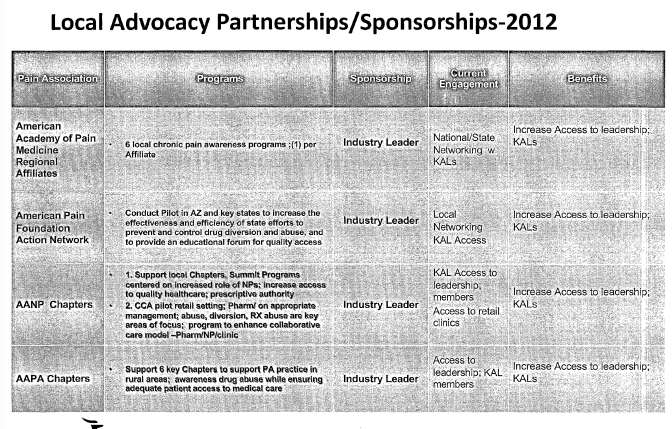


1. The Epidemic of Pain in America
   - [https://www.industrydocuments.ucsf.edu/docs/yqgg0230](https://www.industrydocuments.ucsf.edu/drug/docs/#id=yqgg0230)
   - Author : American Pain Foundation, The; Rogers, Mike
   - Document Date : 2006 June 13
   - Type : report
   - ID : yqgg0230 ( TID : wnf71j00 )
   - ARK : ark:/88122/yqgg0230
   - Collection : Oklahoma Opioid Litigation Documents; Opioid Documents Collection
   - Key Points:
     1. Extensive report (133 pages) supporting the concept of undertreatment of pain
2. Smart Moves Smart Choices School Toolkit
   - [https://www.industrydocuments.ucsf.edu/docs/gfgg0230](https://www.industrydocuments.ucsf.edu/drug/docs/#id=gfgg0230)
   - Author : Unknown
   - Document Date : Unknown
   - Type : form
   - ID : gfgg0230 ( TID : ugf71j00 )
   - ARK : ark:/88122/gfgg0230
   - Collection : Oklahoma Opioid Litigation Documents; Opioid Documents Collection
   - Key Points:
     1. Copy Review Submission Cover Sheet
     2. Brand: Unbranded pain

180) Oklahoma Kids and Adults go Far to Feed their Addiction

Document Data

- Author : Charlie, Price; Woodward, Mark; Oklahoma Bureau of Narcotics; Mathes, Patty
- Document Date : 2008 September 29
- Type : chart; diagram; photograph
- ID : knfg0230 ( TID : acf71j00 )
- ARK : ark:/88122/knfg0230
- Collection : Oklahoma Opioid Litigation Documents; Opioid Documents Collection

Document Notes

- In the attached Diversion report are some very alarming stats from our Prescription Monitoring Program showing the number of Oklahomans going to 5, 10 or more doctors. Some seeing as many as 66 docs to get meds for an addiction. It also shows we dispense nearly 104 million doses of Hydrocodone per month—that's about 30 pills for every man, woman and child in Oklahoma each month.

Tapentadol PR Initiatives

- - [https://www.industrydocuments.ucsf.edu/docs/kmgg0230](https://www.industrydocuments.ucsf.edu/drug/docs/#id=kmgg0230)
  - Author : Lynn, Leonard; OMPUS; Thomas Jefferson University
  - Document Date : Unknown
  - Type : diagram; memo
  - ID : kmgg0230 ( TID : glf71j00 )
  - ARK : ark:/88122/kmgg0230
  - Key Points:
    - “Market Dynamics – Barriers
      - Fear of prescribing
      - Fear of addiction”
    - “Stepping Stones to Success
      - Build Medical Need
      - Support Patient Education
      - Foster Patient – Physician Communication
      - Publicize the Science”
    - “Public Relations Strategic Approach
      - Redirect dialogue from *drug control* to *controlling pain*
    - “’Let’s Talk Pain’
      - Advocacy Coalition
      - Objective: Focus on providers & patients:
        - Improving effective dialogue between physicians & patients
        - Understanding the true impact on SEs on patients
      - Coalition
        - APF: American Pain Foundation (patients)
        - APPM: American Academy of Pain Management (physicians)
        - ASPMN: American Society of Pain Management Nursing (nurses)
    - “’Smart Moves, Smart Choices’
      - Fighting Teen Rx Drug Abuse”
    - “Media Event
      - Objective: A one-day forum to educate the media by reviewing the latest research, patient perspectives, public policy proposal and law enforcement perspectives on pain management
      - Cross-functional KOL speakers
        - Physicians, patients, policy & law enforcement
      - Participants include: media, policy experts, physicians & patients”
    - 2008 Internal Communications Plan
      - E-newsletter: Unbranded efforts
      - Lunch and learn: Pain Awareness Month
    - “Tapentadol Media at APS
      - Three press releases on pivotal Phase 3 data and summary release announcing tapentadol IR presentations
      - Press releases will be issued after embargo: Thursday, May 8
      - Web briefing on Phase 3 data will be pre-recorded and made available for download by media
      - Headlines:
        - Seven clinical presentations on tapentadol immediate release for acute pain to be featured at 2008 American Pain Society Annual Meeting
        - Results of Phase 3 study show tapentadol IR relieves acute pain and offers favorable gastrointestinal tolerability profile
        - New data from Phase 3 study suggests tapentadol immediate release effective for acute pain from common foot surgery
        - New study suggests tapentadol IR for acute pain is associated with improved gastrointestinal tolerability compared to oxycodone IR”
    - “Overcoming ‘Opiophobia’ (CII)”
    - “Initiatives to Overcome ‘Opiophobia’”
      - Opioid Risk Management Tool
      - Pain Policy Resource Center
      - Pain Policy Speaker Initiative
      - Government Affairs Collaboration”
    - “APS Sponsorship Activities
      - Maximize high-impact sponsorship activities to raise awareness of the unbranded campaign launching the NEO Pathways program
        - Commercial Support: Diamond Level

Branded signage, logo, recognition in program materials and brochure, attendee registration list

- - - - - Event Support: Cyber Central

Two locations (by registration area, outside the convention hall and on the convention floor), 6-8 computer terminals with branded screen saver and mouse pad

- - - - - Promotional Activities

Pre-mailer Postcard APS event announcements sent to all convention attendees

Full-page program brochure advertisement (adjacent to the Schedule-of-Events page)

20 x 20 NEO Pathways booth with interactive detail activity, website terminals & professional detailing

- - - - - KOL Engagement: KOL Advisory Board Meeting

Half-day meeting prior to APS with 12-15 KOLs

Dinner reception where KOLs can meet to discuss category data and the market”

- - -
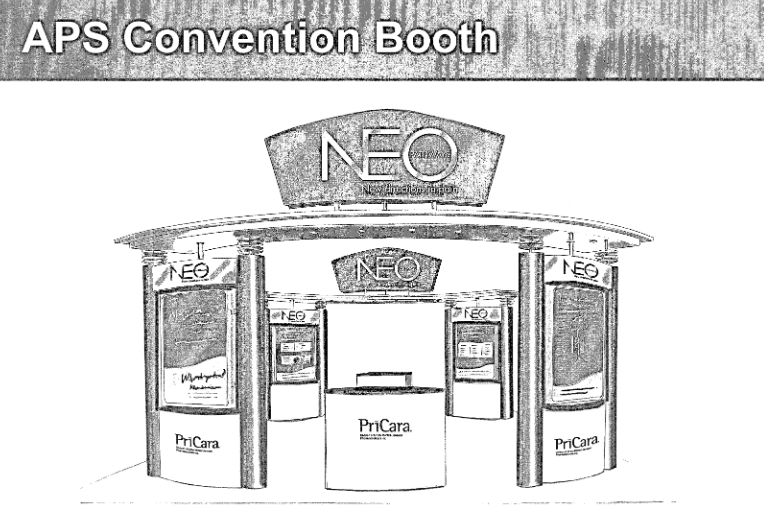


182) Non Branded Round 2 – Final Report Review

Document Data

- Author : Jones, Susan T
- Document Date : 2008 April 10
- Type : email; notes
- ID : lmgg0230 ( TID : hlf71j00 )
- ARK : ark:/88122/lmgg0230
- Collection : Oklahoma Opioid Litigation Documents; Opioid Documents Collection

Document Notes

- **Pain Non-Branded Campaign Market Research – Round 2**
  - The Campaign = Non-Branded Message regarding the under treatment of pain, why so and what can lead to
  - The P3 Program = **Physicians Partnering Against Pain**
  - The Product = Tapentadol IR (NUCYNTA)
- **Market Research Objectives =** Test the non-branded campaign's ability to "impact" the market place behaviors/thinking relative to the treatment &. management of acute pain
  - To Explore physicians’ reactions to four messages presented by a sales representative
  - To understand the impact of the message regarding the undertreatment of acute pain and its implications
  - To understand the connection of educational campaign to the Tapentadol TPP (Triplicate prescription program???)
- **THE MESSAGES – RECAP (see screenshots)**
  - Message = Undertreatment of Pain
    - Behavioral Change = will attempt better assessment of acute pain
  - Message = Consequences
    - Behavioral Change = **More aggressive approach to treating (stronger dosing and meds);** desire to spread the message to other physicians
  - Message = Concerns that Hinder Effective Pain Mgmt
    - Behavioral change = Low abuse numbers
    - About half believe the addiction percentage (For the most part, the Rep was successful with refocusing them from addiction to side effect concerns)
  - Message = Multi-Pathways
    - NEW AGENTS – we have a new solution
    - Behavioral Change = **Increased opioid use (PCP), increase use of poly pharmacy until Product Y becomes available**
- **Impact of Campaign Overall =** PCPs state that they will be more aggressive in their treatment and use more opioids
- Physicians offer the following advice and suggestions on how to better present the educational campaign regarding acute pain management
  - Include thought leader presentations and peer-to-peer discussions in the campaign
- Reactions to Tapentadol IR TPP
  - Other benefits include efficacy similar to oxycodone, low withdrawal potential, **and some physicians on their own made a leap that it may result in lower addiction potential**
- Physicians report many patient types as undertreatment. These include: **the elderly**, **younger patients**, post-op and **post-trauma patients**
  - RECOMMENDATION = Include these patients in the undertreatment message

184) National Association of School Nurses Launches Educational Program to Address Surge of Prescription Drug Abuse by America’s Youth

Document Data

- Author : National Association of School Nurses; NASN; Murphy, Jaime; Derden, Michelle
- Document Date : 2008 April 06
- Type : press release
- ID : ksfg0230 ( TID : cff71j00 )
- ARK : ark:/88122/ksfg0230
- Collection : Oklahoma Opioid Litigation Documents; Opioid Documents Collection

Document Notes

- The National Association of School Nurses (NASN) announced today **"Smart Moves, Smart Choices,"** a program that responds to the growing rate of prescription drug abuse among middle and high school students nationwide.
- "Smart Moves, Smart Choices" educates teens about the serious risks of abusing prescription medicines, such as those used legitimately to treat pain.

81) 2013 National Advocacy Business Planning

Document Data

- Author : Johnson and Johnson; Janssen Pharmaceutical
- Document Date : 2012 June 29
- Type : proposal
- ID : zggg0230 ( TID : yhf71j00 )
- ARK : ark:/88122/zggg0230

Document Notes

- Business planning from Janssen (PAIN FRANCHISE) June 29, 2012
- Key Questions = How to leverage sales of Nucynta??
- 2013 PAIN Advocacy Strategy
  - Engage partners to embrace the IOM report-national/state implications advocate for and act collectively to actualize the recommendations
  - **Influence agencies that impact policy and quality to maintain or improve access**
- Barriers to care
  - FOCUS: **Engage with advocacy partners** at the national level for greater impact and alignment. Support models for collaboration and synergies that can then be applied at a regional level
  - Chronic pain can and should be thought of as a disease in and of itself.
  - Public health and community-based approaches are required to **address the under-treatment of chronic pain**
  - **Smart Moves, Smart Choices are underutilized!**
- Advocacy/Policy Focus
  - Provide state and federal legislators and other regulatory groups with access to objective materials to assist them in making public policy.
  - Collaborate with the **Pain Care Forum (PCF)** on policy issues and common strategies with key decision makers; such as HHS, Surgeon General's Office, CDC, state and federal legislators and regulators. •
  - Collaborate with State Pain Policy Action Coalition (SPPAC), a newly formed organization made up of pain focused organizations that will pro-actively inform and influence state policy . •
  - **Sponsor Public Awareness campaigns targeted at preventing chronic pain and misuse of prescription pain medications. •**
  - **Sponsor disease awareness to promote balanced and effective pain management. •**
  - Support collaboration between Medicine and Law Enforcement to prevent the "chilling effect".
    - [Chilling effect](https://oxfordmedicine.com/view/10.1093/med/9780190659721.001.0001/med-9780190659721-chapter-10) ([link](https://oxfordmedicine.com/view/10.1093/med/9780190659721.001.0001/med-9780190659721-chapter-10)) = An overzealous Drug Enforcement Administration is sometimes prosecuting the wrong physicians, thus creating a chilling effect in the medical community with regard to opioid prescription and making it harder for people in pain to get the help they need
  - Support effective Prescription Monitoring Programs (PMPs) that provide prescribing healthcare professionals with "real time access" and improve patient care.
- Addressing the Growth of Prescription Drug Abuse Among Teens
  - [https://www.industrydocuments.ucsf.edu/docs/ptfg0230](https://www.industrydocuments.ucsf.edu/drug/docs/#id=ptfg0230)
  - Author : NASN; PriCara; National Association of School of Nurses; Partnership for a Drug-Free America
  - Document Date : Unknown
  - Type : article; photograph; report
  - ID : ptfg0230 ( TID : xff71j00 )
  - ARK : ark:/88122/ptfg0230
  - Collection : Oklahoma Opioid Litigation Documents; Opioid Documents Collection
  - Key Points:
    - “Smart Moves, Smart Choices”
    - December 21, 2009
    - “Conducted media tour featuring addiction expert Dr. Drew Pinsky”
    - “Media Impact
      - Dr. Drew Pinsky interviewed by 41 media outlets, including top-tier media in markets most impacted by prescription drug abuse, reaching an audience of more than 9 million
      - Matte release and radio news release resulted in over 600 placements
    - Educational Videos and Curricula
      - More than 100 requests for DVDs from school nurses, teachers, community substance abuse educators and counselors
      - Distributed 90,000 post cards to physicians offices via sales force
      - Distributed 500 DVDs to school nurses at NASN Annual Meeting and 1,500 DVDs and post cards during Pain Week
    - School Assemblies / Assembly Tool Kit
      - More than 30 requests from school nurses to host a local assembly
      - Three school assemblies held in markets impacted by prescription drug abuse; with key community members, parents and media outlets in attendance
      - School assembly tool kit posted on the NASN.org viewed more than 1,600 times”

1. The Epidemic of Pain in America
   - [https://www.industrydocuments.ucsf.edu/docs/yqgg0230](https://www.industrydocuments.ucsf.edu/drug/docs/#id=yqgg0230)
   - Author : American Pain Foundation, The; Rogers, Mike
   - Document Date : 2006 June 13
   - Type : report
   - ID : yqgg0230 ( TID : wnf71j00 )
   - ARK : ark:/88122/yqgg0230
   - Collection : Oklahoma Opioid Litigation Documents; Opioid Documents Collection
   - Key Points:
     1. Extensive report (133 pages) supporting the concept of undertreatment of pain
     2. AUTHORS
        1. Howard Heit – paid consultant for Cephalon and J&J ([link](https://projects.propublica.org/d4d-archive/search?utf8=%E2%9C%93&term=howard+heit&state%5Bid%5D=47&company%5Bid%5D=&period%5B%5D=&services%5B%5D=))
        2. PAIN CARE FORUM + Representative Mike Rogers (R-MI)
        3. Presented at the Cannon House Office Building, Washington DC
     3. Interesting Tidbits
        1. Pain affects people at all stages of life - including infants, children, young adults, and the elderly.
        2. For one third of sufferers, their chronic pain was so severe and debilitating, they felt they couldn't function as normal people and sometimes felt so bad they wanted to die.
        3. 2/3 of older Americans who take pain medications said pain still prevents them from performing routine tasks, engaging in hobbies, or doing things they enjoy. Of these, 44% said they had difficulty Walking .
     4. Personal Stories of Pain
        1. Alexandra, sixteen years old (patient w/RSD)
           1. At this moment I am trying to start a program at the local hospital for kids facing chronic pain problems and giving them support and just a friend.
        2. Jim, US Military Veteran
           1. For years my pain was ignored. I was labeled as a drug seeker. The Veterans Hospitals would never address the real cause
           2. They [VA] didn't know how to treat what they created
           3. This disease has no cure but to take pain medication.
           4. I have had to fight the Veterans Heath Care System for years
     5. The pain gap – minorities in America are considered “under treated for their pain from medical conditions”
        1. Donald Dillard counted himself lucky to survive when a loaded dump truck rolled over his body, crushing his pelvis and thighs and shredding a football-size chunk of flesh in his torso. But the 15 years since have been a nightmare of agonizing days and nights made worse by the lack of potent pain medications at pharmacies near his home, struggles to get proper care and the red tape involved in workers' compensation coverage
        2. Hispanics and African Americans are consistently under-treated for pain across a range of conditions, from cancer to chest pain to broken bones
        3. "There are many people not getting treatment for their chronic pain, and race is part of the equation."
        4. Just as important, Staats added, is that pain management is not fully recognized as a board specialty by the American Board of Medical Specialties, "But there are a group of like-minded doctors who recognize that there is disease of chronic pain," said Staats, who serves on the board of directors of the American Academy of Pain Medicine. "Chronic pain affects a person in so many different ways — physical, psychological, emotional
     6. Professional Education – Fast Facts
        1. Most front-line family practitioners and specialists have very little training or education in identifying and treating the various distinct kinds of pain conditions.
        2. Despite the fact that pain is the number one condition that brings people to the doctor, pain education is barely present in most medical school curricula.
        3. **There are millions of people living with pain who are told by healthcare professionals that opioid medicines, one of the most powerful and effective class of medicines, are addictive and shouldn't he used.**
        4. Each year there are millions of people near death and in severe pain when there are effective pain medicines and treatments available to relieve them of their pain.
        5. **Health professionals and the public are unaware:** • Physical dependence on a medication is not the same as addiction. • Appropriate use of opioid medications (like oxycodone) is safe and effective and unlikely to cause addiction in people who are under the care of a doctor and who have no history of substance abuse. • Opioid medications are sometimes the only effective treatment for some types of pain.
        6. The typical healthcare practitioner is both time-challenged and **clueless** with regard to the treatment of pain,
        7. Americans come to their healthcare professionals expecting to be cared for, to have their pain relieved, but due to practitioner ignorance about the significance of untreated or undertreated pain, and the possibility of prosecution, they opt to leave patients in pain as the "safest course."
     7. Balancing the Need to Curb Diversion of Pain Medicines and the Need to Protect Access to Pain Medicines
        1. In most communities it is difficult for pain patients to find physicians willing to prescribe opioid medicines for pain.
        2. There is a need to reform overly restrictive regulatory polices which impede pain relief.
     8. PITCHING RELIEF – Firsthand account from Dr. Heit (paid consultant)
        1. Heit, 61, doesn't use prescription opioids for his own pain now, but he does prescribe them in high doses to many of his patients, and he's seen the drugs (in conjunction with proper monitoring) provide remarkable relief ~ the kind he still wishes he had had available back when he really needed it. As the showdown between pain doctors and prosecutors stiffened several years ago, he felt obliged to get more actively involved in defense of opioid treatment despite the potential risk to his practice
        2. Shugol, 54, wheeled herself into Heit's Arlington Boulevard office two weeks ago for a monthly appointment, smiling broadly and filled with an energy seldom seen in people who don't carry her many physical burdens. The first order of business was, as always, to hand Heit her vials of drugs, so he could see exactly how many pills she had used since the last visit. Heit took out a pill counter and went to work, first on the OxyContin, and then the Dilaudid. He was puzzled to find more than 100 extra pills. "Have you been taking everything you need?" he asked. "Yep, but I think you made a mistake last time," she replied. Rather than writing a prescription for 230 pills, Heit had written one for 330 pills, and that's what the pharmacist filled. As Shugol continued to talk of the active life she can now have because of the opioids and her care by Heit, the doctor went through the detailed paperwork he keeps on all patients.
        3. Shugol had followed Kathryn Brock of Reston — another woman in a wheelchair with an easy smile and a strong desire to remain active - into Heit's office. Brock sufferers from rheumatoid arthritis in virtually every joint in her body, and she, too, is subject to constant pain. She says that her regimen of six OxyContin and eight Dilaudid pills a day has kept her marriage going, and gives her the ability to continue painting, which she does regularly
        4. **The Gift of a Baby** A fourth patient seeing Heit earlier this month was a young woman who had fallen down a flight of stairs at a nightclub and mptured two disks in her neck. Another jock accustomed to playing with pain, she tried to gut her way through it. Doctors recommended surgery, but she resisted. Her boyfriend recommended Heit, and she began treatment. She improved markedly, married the boyfriend and, while still on OxyContin, got pregnant. She knew she could never get through the pregnancy without the medication, but she was concerned that the child could be in withdrawal at birth. When the baby was bom in 2004, Heit was at the hospital to examine the newborn for signs of opioid trouble, and was relieved to find none. Heit is the kind of person who likes to connect quickly and deeply to others, and so it wasn't entirely unexpected that he would be in the hospital. But there was another reason for his presence. The new mother, whom Heit had received permission from Virginia medical authorities to treat, was Jamie Heit ~ his son David's wife. And the healthy child, bom to a woman who wasn't sure she could ever carry a baby after her fall, was his first granddaughter, Lilly.
   - Empowerment – WOMEN
     1. Create patient demand for proper pain diagnosis and treatment; encourage patients to advocate for their rights
     2. Consider vital tactics/word of mouth education – let women educate women in their natural setting **(Tupperware parties, garden groups, e-cards)**
     3. Conduct media roundtable w/ women’s books; could work with additional partner with a female focus (like the National Women’s Health Resource Center)
     4. Consider using applicable information from the “Savvy Woman Patient” chapter on women & pain
   - CHRONIC PAIN HITS WOMEN HARDER – Jennifer Wider, Society for Women’s Health Research
   - Provide visuals of multiple patients (woman, man, child, elderly person) to help them find their fit
     1. Each visual could contain descriptions to help patients identify
   - Flyer from Research!America = **Facts about Pain – Investment in research saves lives and money**
     1. Pain Today
        1. As many as 20% of children experience chronic pain
        2. Inadequate pain management is more severe problem for ethnic and racial minorities than for other population groups
     2. The Bottom Line
        1. In a recent Research!America public opinion survey, 57% of Americans reported experiencing chronic or recurrent pain. Sixty-six percent of those in pain expect to live with it for the rest of their lives. Americans want and expect a sustained investment in research to alleviate the burdens caused by chronic pain.
   - **When it comes to severe pain, doctors still have much to learn**
     1. A Painful lesson
        1. I called the surgeon's office weekly and reported my minimal progress in pain control, but at no point was an increase in pain medication suggested, nor was I referred to a pain management specialist on the hospital staff
        2. When, at seven weeks after surgery, I spoke to Dr. Schneider, a Tucson-based specialist in pain management and addiction medicine, she chastised me for not being more insistent about getting adequate pain relief. The trouble is, when you're experiencing intense pain, it's hard to be proactive about anything
        3. Many doctors are afraid to prescribe narcotic drugs like oxycodone, fearing they will create addiction problems. But that in fact rarely happens to chronic pain patients who don't have a history of addiction. When a pain patient needs increasing doses of a narcotic, it's nearly always because the pain worsens, as often happens in patients with advanced cancer. Patients do become tolerant to side effects, like grogginess, but rarely to the pain-relieving properties of these drugs.
     2. When the Nerves Respond
        1. When I read this,! realized I was on the wrong track, taking too little of the long-acting drug and too much of the short-acting one
        2. As it turned out, my internist knew far more than my surgeon about treating pain. He has many elderiy patients with chronic pain and knows very well how to treat it. I realize now I should have sought his help from the beginning. Or I should have asked to be referred to a pain management specialist at the hospital where I had my surgery
     3. Let’s Fix What’s Broken
        1. First and foremost, patients need to be proactive and insist on the help they need. If patients are not able to do this for themselves, an advocate should do it for them. Second, every person with prolonged or chronic pain should become educated about the huge range of medications, therapies and complementary remedies available to treat pain.
   - **Painful Choices: Physicians challenged by quest to end suffering**
     1. Nursing home patients have historically been some of the most undertreated for pain. The complaints of fragile elderly are sometimes dismissed as "normal aging," It's even worse, says Community Nursing Services hospice director Dr. Anna Beck, for those who are too weak or confused to communicate
     2. When it comes to severe pain, it's called "pain management'' for a reason, because some pain is so awful that it can't entirely be erased. Marcia Solum is thrilled to now have pain she can live with, Before, when her pain was unbearable, she would sometimes take eight Advil every four hours. "I was trying to be able to have a life, and I just couldn't," she says, her voice breaking. Now on a morphine pump, she can get out of bed each day, teach sewing classes, have 25 people over for Thanksgiving dinner.
   - **PREVALENCE: Fast Facts**
     1. For American military, the current way in Iraq is resulting in a significant number of body and limb injuries which often lead to life-long chronic pain conditions


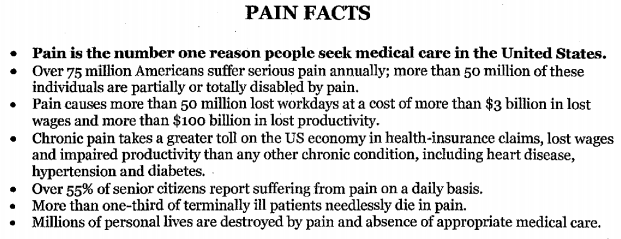


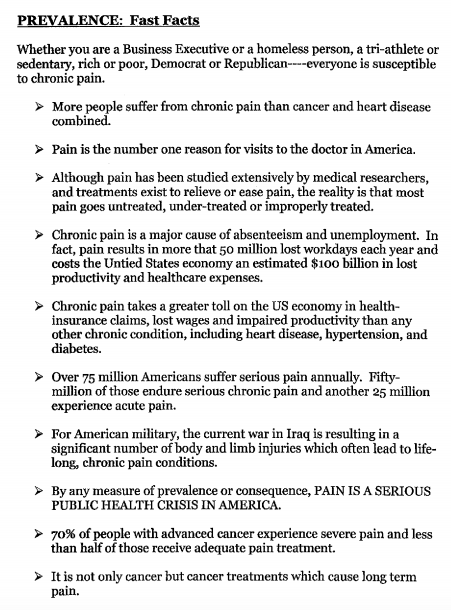


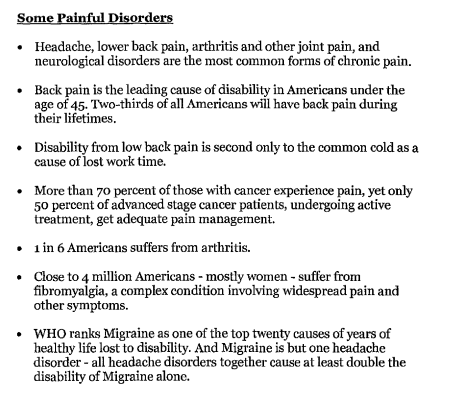


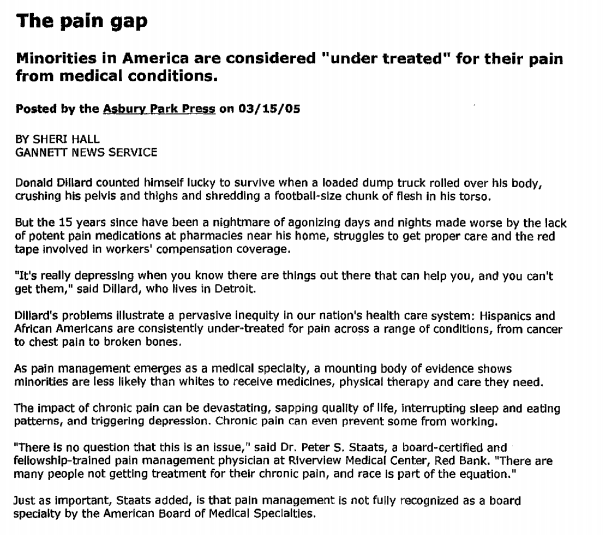


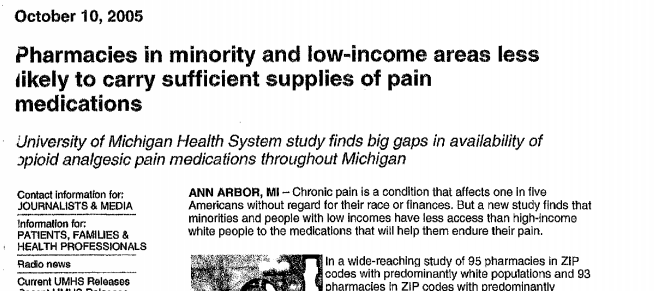


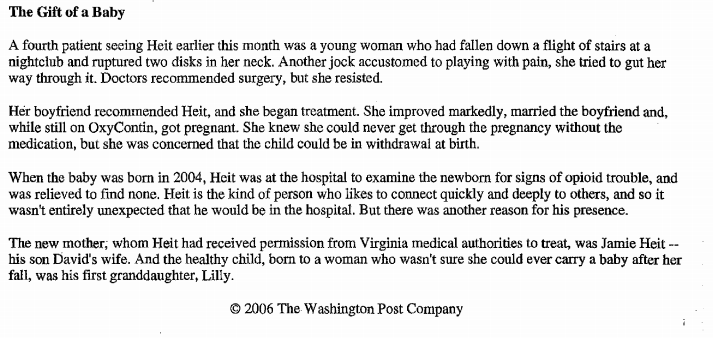


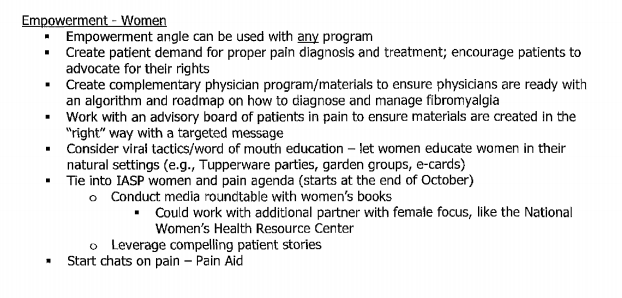


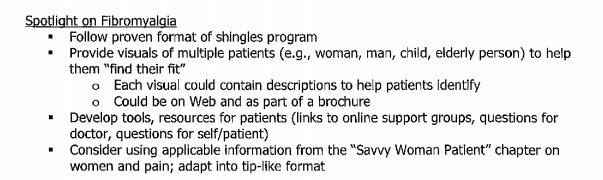


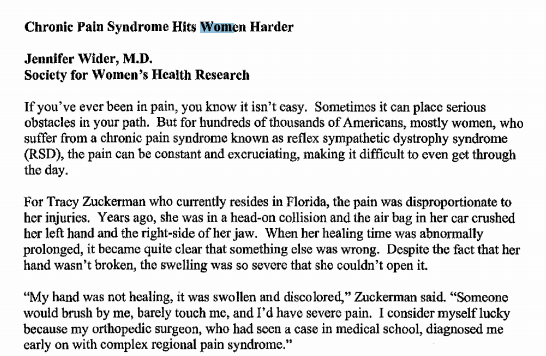


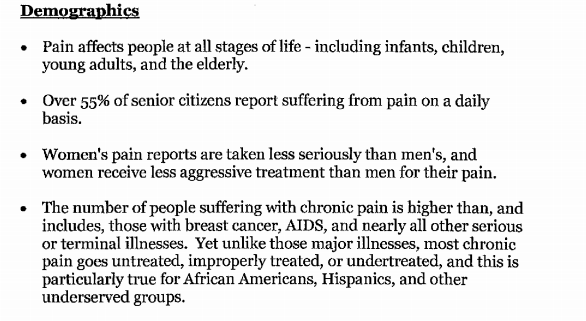


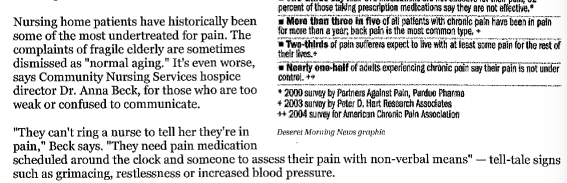


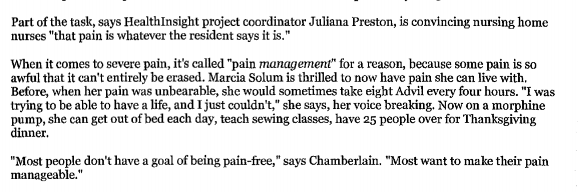


183) Stepping Stones to Success

Document Data

- Author : Leonard, Lynn
- Document Date : 2008 April 10
- Type : email; notes; report
- ID : nygg0230 ( TID : pmf71j00 )
- ARK : ark:/88122/nygg0230
- Collection : Oklahoma Opioid Litigation Documents; Opioid Documents Collection

Document Notes

- Title = **Tapendtadol PR Initiatives**
- Public Relations Strategic Approach = **Redirect dialogue from drug CONTROL to CONTROLLING PAIN**
  - 1) Emphasize the need – Advocacy Development
  - 2) Make the case – Reimbursement Support
  - 3) Do the right thing – Fighting Teen RX Drug Abuse


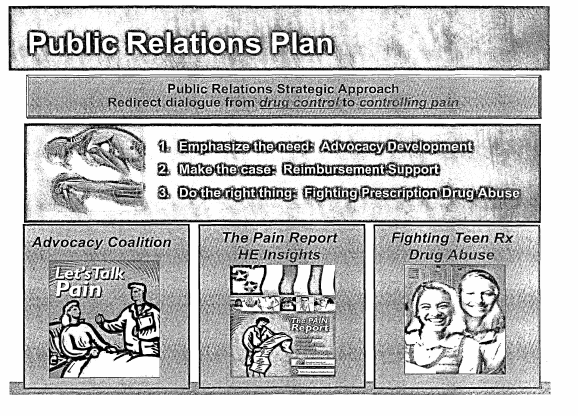


1. Pain Brief Advocacy and Policy Monthly 2011
   - [https://www.industrydocuments.ucsf.edu/docs/ymgg0230](https://www.industrydocuments.ucsf.edu/drug/docs/#id=ymgg0230)
   - Author : Unknown
   - Document Date : Unknown
   - Type : report; report, scientific
   - ID : ymgg0230 ( TID : klf71j00 )
   - ARK : ark:/88122/ymgg0230
   - Collection : Oklahoma Opioid Litigation Documents; Opioid Documents Collection
   - Key Points:
     1. Janssen internal confidential publication
     2. “Primary external partners:”
        1. “American Pain Foundation/State Action Network, ‘Go to Partner’”
        2. “American Pain Society-regional affiliates”
        3. “American Academy of Pain Medicine-regional societies”
        4. “American Academy of Pain Management”
        5. “American Chronic Pain Association”
     3. “Pain Tools Disseminated:
        1. Unbranded Programs-
           1. Prescribe Responsibly-external HCP community
           2. Smart Moves, Smart Choices-Teen Rx Abuse
           3. Let’s Talk Pain-Provider/Patient Communications
        2. Prescribe Responsibly
        3. HPAD Partner Resource Kits
        4. Burden of Pain Deck”
     4. “Note: Pain Care Task Force is being formed to develop a communication and outreach plan for dissemination of key messaging across national/local markets. The activities can serve as an advocacy tool for our partner organizations.”

204) Non-Branded Promotion

Document Data

- Author : Leonard, Lynn
- Document Date : 2007 June 29
- Type : diagram; email; report
- ID : fngg0230 ( TID : rlf71j00 )
- ARK : ark:/88122/fngg0230
- Collection : Oklahoma Opioid Litigation Documents; Opioid Documents Collection

Document Notes

- Email from Lynn Leonard
  - here are some slides on the unbranded efforts for the tactical presentation
  - I have included the "banana" analysis on one of the slides - which gets us to 60% of physician targets and gives us 60% of the business (targeted business).
- Preliminary Message Points
  - 1) Pain is mismanaged and under treated
  - 2) The consequences are serious and significant
  - 3) Appropriate treatment requires effective pain control
- Capturing and Maintaing Interest
  - Supported through lively stories – Illustrate patient/MD gap and highlight elderly patients

Slide Title: **PriCara Non-Branded Promotion**

- Disrupting a Complacent Marketplace
  - Web Strategy: Professional Promotional Platform + Physician-Patient Education/PR Strategy
- Disrupt the Marketplace
  - Heighten awareness of the under-treatment of pain and its consequences
  - Establish reps as pain experts
  - Profile targeted HCPs in preparation of launch
- Unbranded Message Continuum – Paving the way for tapentadol
  - 1) Pain is mismanaged and under-treated
  - 2) There are serious consequences of mismanaging & under-treating pain
  - 3) Appropriate treatment requires EFFECTIVE pain relief
  - 4) New pathways in pain management coming soon
- Pain Management: **At the Threshold of NEO Thinking**
- Gaining Endorsements for Credibility
  - Establish Instant Credibility
  - Develop Good Will
  - Alignment of Long Term Goals
  - Alleviate Regulatory Anxiety


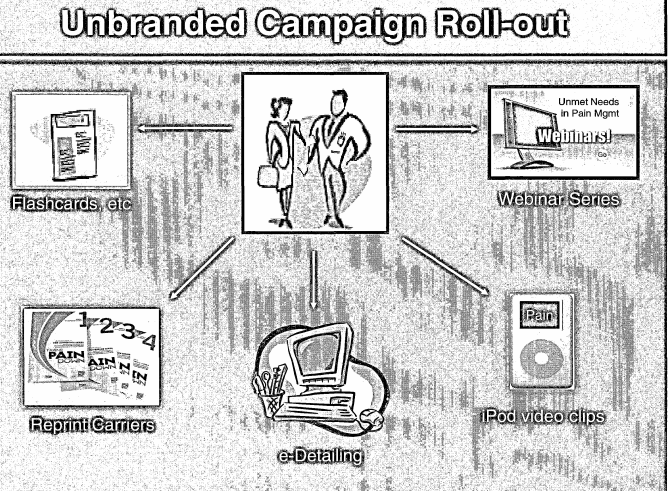


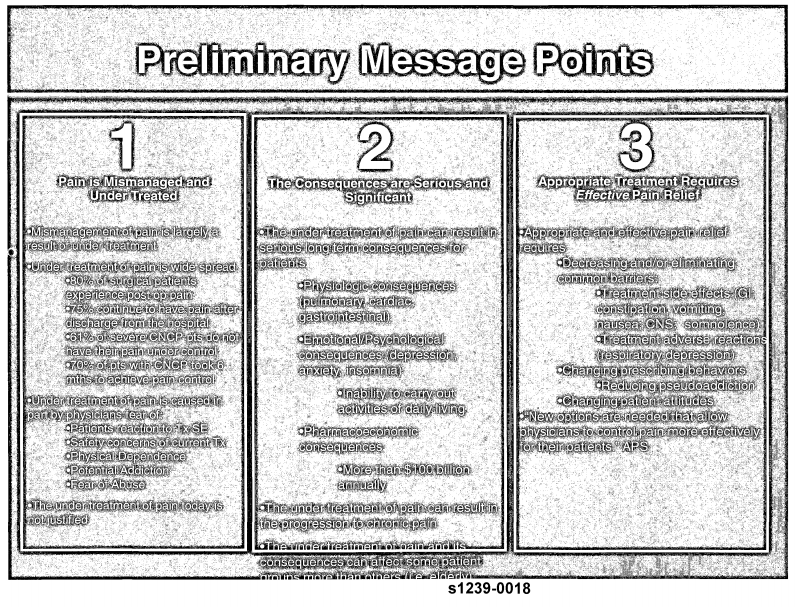


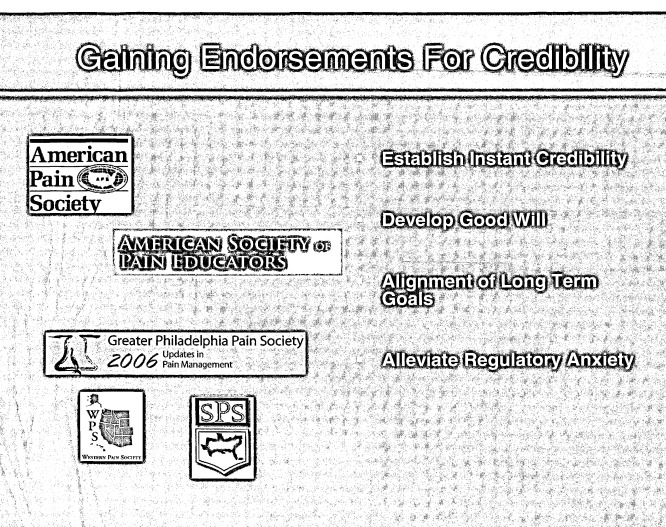


262) Strategic Customer Group 2003 Business Plan

Document Data

- Author : Unknown
- Document Date : 2003
- Type : report, scientific; table
- ID : frgg0230 ( TID : dof71j00 )
- ARK : ark:/88122/frgg0230
- Collection : Oklahoma Opioid Litigation Documents; Opioid Documents Collection

Document Notes

- **J&J Long Term Care Group & Janssen Elder Care – Composites and Demographics**
- The SCG vision is to "ensure maximum access, reimbursement, profitability and compliance within all strategic customer segments while improving customer satisfaction"
- Long Term Care Segment
  - The LTC market is currently valued at $10 billion and is growing at a robust 12% annual rate
  - Much of the recent growth in LTC has been driven by demographic trends. The elderly (65+) represent the fastest growing segment of the population, increasing at three times the rate of the overall population.
  - This market potential has been universally recognized and is now a major focus within the pharmaceutical industry. Evidence includes the recent creation of specialty senior care sales forces, as well as new product launches in areas of high elderly potential (i.e., Alzheimer's, APS, chronic pain).
  - Much of the future growth in this segment will be derived from the rapid expansion of assisted living facilities (ALFs)
  - Share has increased for other Janssen and J&J products, including **DURAGESIC**®, REMINYL®, LEVAQUIN®, **ULTRACET**®, and DITROPAN XL®. This market segment represents a significant opportunity for the Johnson & Johnson LTC Group, especially since the J&J Pharmaceutical Group covers almost all of the high spend therapeutic areas for these elderly patient
  - LTC Goals is to deliver $970MM in Janssen sales
  - Developing relationships with Geriatric Nurse Practitioners and business opportunities in the emerging assisted living sector are expected to be key critical success factors.
- J&J Long Term Care Group – Situation Diagnostics
  - The consultant pharmacists are an important influence in the long-term care segment and play a critical role in encouraging appropriate use of pharmaceuticals. **If leveraged appropriately, they can act as an extension of our sales force.**
- ULTRACET and DURAGESIC also offer growth opportunities in the LTC Market. Pain is seriously under-treated in the elderly.
  - Consequently, the LTC Group's efforts in 2003 will be directed towards accelerating the growth of ULTRACET, which is the latest addition in the pain market and continuing the market share gains experienced by DURAGESIC

300) Smart Moves Smart Choices School Toolkit

Document Data

- Author : Unknown
- Document Date : Unknown
- Type : form
- ID : gfgg0230 ( TID : ugf71j00 )
- ARK : ark:/88122/gfgg0230
- Collection : Oklahoma Opioid Litigation Documents; Opioid Documents Collection

Document Notes

- Nothing new here; SMSC is an unbranded initaitive from PriCara


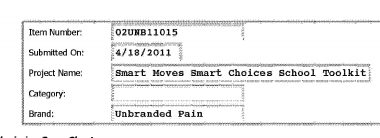


**MILITARY**

70) Email from Felice Sweeney to Patricia Cosler Regarding the Chronic Pain Management Strategies and Lessons from the Military

Document Data

- **Author :** Sweeney, Felice
- **Document Date :** 2013 May 24
- **Type :** email
- **ID :** jqgg0230 ( TID : rnf71j00 )
- **ARK :** ark:/88122/jqgg0230
- **Collection :** Oklahoma Opioid Litigation Documents; Opioid Documents Collection

Document Notes

- “Chronic Pain Management Strategies and Lessons From the Military: A Narrative Review” has been successfully submitted to the *Annals of Internal Medicine*
- Email from Felice Sweeney --> **employee at Medergy Scientific, Inc**
  - [https://www.dnb.com/business-directory/company-profiles.medergy_scientific_inc.332a3021c3bf8b1f999e7b9f01cdbfad.html﷟HYPERLINK "https://www.dnb.com/business-directory/company-profiles.medergy_scientific_inc.332a3021c3bf8b1f999e7b9f01cdbfad.html"](https://www.dnb.com/business-directory/company-profiles.medergy_scientific_inc.332a3021c3bf8b1f999e7b9f01cdbfad.html%22%EF%BF%BDHYPERLINK%20%22https://www.dnb.com/business-directory/company-profiles.medergy_scientific_inc.332a3021c3bf8b1f999e7b9f01cdbfad.html)
    - **Medergy Scientific, Inc.** is located in Yardley, PA, United States and is part of the Advertising & Marketing Services Industry. Medergy Scientific, Inc. has 40 total employees across all of its locations and generates 0 million in sales (USD).
  - <https://www.linkedin.com/company/medergy-healthgroup>
- Email sent to the Authors of the paper + “Gary Baker”
  - Can’t find who exactly Gary Baker is??
  - Authors listed on the paper: April Hazard Vallerand, Patricia Cosler, Jack E Henningfield, Pam Galassini
- Paper: “Chronic Pain Management Strategies and Lesson from the Military: A Narrative Review”
  - **ABSTRACT: The application of US military pain management guidelines has been shown to improve pain monitoring, education and relief. In addition, the US military has instituted the development of programs and guidelines to ensure proper use and discourage aberrant behaviours with regard to opioid use, because opioids are regarded as a critical part of acute and chronic pain management schemes. Inadequate pain management, particularly inadequate chronic pain management, remains a major problem for the general population in the US. Application of military strategies for pain management to the general US population may lead to more effective pain management and improved long-term patient outcomes**
  - The paper heavily repeats the major talking points from Johnson and Johnson
    - Opioids are needed in CHRONIC pain management
    - Abuse potential for opioids are limited and can be avoided
    - The REAL problem is inadequate pain management
  - **Acknowledgements**: Editorial support for manuscript preparation was provided by Megan Knagge, PhD, of MedErgy, and was funded by Janssen Scientific Affairs, LLC. The authors retained full editorial control over the content of the article. The authors acknowledge the members of the **Imagine the Possibilities Pain Coalition (Coalition started and funded by Jansenn!!!! See #82)** for their helpful discussions and critical review of this manuscript: Gary Baker, Jeff Buel, Arthur Caplan, Penney Cowen, Myra Glajchen, Phyllis Grauer, Myoung Kim, Robyn Kohn, Richard Payne, Jennifer Pluim, Frank Sapeinza, Scott Taylor, Robert Twillman, Jon Ukropec, Gary Vorsanger, Behin Yektashenas.
  - **Disclosures**: P Cosler was an employee of Janssen Scientific Affairs, LLC, at the time of the writing of the manuscript and declares no current conflicts of interest. AH Vallerand and P Galassini declare no conflicts of interest. JE Henningfield provides consulting services through Pinney Associates to pharmaceutical developers and marketers of central nervous system-acting drugs, including analgesics, to help assess the abuse liability of the drugs and develop appropriate recommendations for labelling and risk management to minimize the risks of abuse, overdose and other unintended effects.

Document Relevance = YES

81) 2013 National Advocacy Business Planning

Document Data

- Author : Johnson and Johnson; Janssen Pharmaceutical
- Document Date : 2012 June 29
- Type : proposal
- ID : zggg0230 ( TID : yhf71j00 )
- ARK : ark:/88122/zggg0230

Document Notes

- Business planning from Janssen (PAIN FRANCHISE) June 29, 2012
- Key Questions = How to leverage sales of Nucynta??
- 2013 PAIN Advocacy Strategy
  - Engage partners to embrace the IOM report-national/state implications advocate for and act collectively to actualize the recommendations
  - **Influence agencies that impact policy and quality to maintain or improve access**
- Barriers to care
  - FOCUS: **Engage with advocacy partners** at the national level for greater impact and alignment. Support models for collaboration and synergies that can then be applied at a regional level
  - Chronic pain can and should be thought of as a disease in and of itself.
  - Public health and community-based approaches are required to **address the under-treatment of chronic pain**
  - **Smart Moves, Smart Choices are underutilized!**
- Advocacy/Policy Focus
  - Provide state and federal legislators and other regulatory groups with access to objective materials to assist them in making public policy.
  - Collaborate with the **Pain Care Forum (PCF)** on policy issues and common strategies with key decision makers; such as HHS, Surgeon General's Office, CDC, state and federal legislators and regulators. •
  - Collaborate with State Pain Policy Action Coalition (SPPAC), a newly formed organization made up of pain focused organizations that will pro-actively inform and influence state policy . •
  - **Sponsor Public Awareness campaigns targeted at preventing chronic pain and misuse of prescription pain medications. •**
  - **Sponsor disease awareness to promote balanced and effective pain management. •**
  - Support collaboration between Medicine and Law Enforcement to prevent the "chilling effect".
    - [Chilling effect](https://oxfordmedicine.com/view/10.1093/med/9780190659721.001.0001/med-9780190659721-chapter-10) ([link](https://oxfordmedicine.com/view/10.1093/med/9780190659721.001.0001/med-9780190659721-chapter-10)) = An overzealous Drug Enforcement Administration is sometimes prosecuting the wrong physicians, thus creating a chilling effect in the medical community with regard to opioid prescription and making it harder for people in pain to get the help they need
  - Support effective Prescription Monitoring Programs (PMPs) that provide prescribing healthcare professionals with "real time access" and improve patient care.

86) Pain Franchise 2012 PR Program

Document Data

- **Author :** *Unknown*
- **Document Date :** 2012 February 10
- **Type :** notes; report; report, scientific
- **ID :** nrgg0230 ( TID : lof71j00 )
- **ARK :** ark:/88122/nrgg0230
- **Collection :** Oklahoma Opioid Litigation Documents; Opioid Documents Collection

Document Notes

- Long Term PR Goal: **Build out narrative and deliver data points that establish NUCYNTA as the right choice for patients and a potential solution to a broader societal problem**
  - Establish NUCYNTA as new standard in moderate-severe pain
  - Demonstrate industry leadership in advocacy for HCP & patient access
- IOM Study: Relieving Pain in America ([LINK](https://healthland.time.com/2011/06/29/report-chronic-undertreated-pain-affects-116-million-americans/))
  - **New England Journal of Medicine editorial (Jan. 2012) from Dean of Stanford Med School** quotes IOM study More than 116 million Americans have chronic pain Financial costs ranging from $560 to $635 billion per year
- Seizing Media Opportunities
  - Two **RADAR** posters
    - Non-med use of Tapentadol
    - Non-med use of opioids/tapentadol among college students
  - Focus on **MILITARY** -- "The long road home"-lraq/Afghanistan troops the next gen of chronic pain patients
    - News Hook: Major troop withdrawals, return to civilian life with chronic pain
    - News Hook: Those who have served, need to be served
- Educate/Influence to Maintain Physician & Patient Access – Public affairs/policy support
  - Laser focus on states where access is threatened
- “Tell the true story of unmet medical need in chronic pain patients”

92) Letter from Robyn Kohn to Tricia Haertlein enclosing Meeting 2

Document Data

- **Author :** Kohn, Robyn
- **Document Date :** 2011 November 01
- **Type :** email; presentation; slides; report
- **ID : mmgg0230**

Document Notes

- Meeting of **Imagine the Possibilities: Pain Coalition**
- 3 subteams charged with creating message
  - MEDIA OUTREACH – Targets = **YOUTH, VETERANS**, PUBLIC
  - POLICY/ADVOCACY
    - Peer-reviewed publication in health policy journal
- Messaging: **Chronic pain as public health problem, chronic pain is multi-contextual**
- Advocacy + Pain Policy Sub-Team Platform
  - **Chronic pain is the #1 public health problem**
  - **Epidemic of Pain v Epidemic of Addiction**
  - **Wide dissemination of plan**
    - Advocacy magazines – AAPM Currents, AAFP Live, AAN Neurology Today
    - General public – Op-eds, popular media, retail chains (**Starbucks pain message of the day????)**
- Education Sub-Team Platform
  - Major needs
    - Placard for HCP so they keep pain conversation front of mind (should fit in lab coat)
    - Quick Guide geared toward community-based pharmacists
    - Medical school curriculum development grant programs
- MEdia Outreach Initiatives
  - Reaching out to: **YOUTH**
    - Reach early – elementary school level – via respoected channels (coaches)
    - Delivery a practical message: **pain is your body telling you something important**
  - Reaching out to: **RETURNING VETERANS**
    - Where do they get their info about pain?
    - Change the paradaign: **the positive side of pain management**
  - Reaching out to: **MEDIA**
    - Capture venues that the media want to cover: art cretaed by people in pain
    - Emphazie the problem of **poorly managed pain is often lost to the topic of addiction fear**; even though it [poorly managed pain] is an issue of many magniutes greater concern [than addiction]
- Teams and Members
  - **MEDIA OUTREACH = “destigmatize pain”**
    - **Jack Henningfield**
    - **Patricia Cosler**
    - **Apriler Vallerand**
    - **Pam Galassini**
    - ^^^**These are all authors of the Military & Chronic Pain paper!!**
  - **PUBLIC POLICY = “change the conversation about pain”**
    - **Bob Twillman**
    - Robyn Kohn
  - **EDUCATION = “deliver the education”**
    - Art Caplan
    - Myra Glajchen
    - Gary Baker
    - Richard Payne
    - Penny Cowan

104) Pain Brief Advocacy & Policy Monthly 2011

Document Data

- **Author :** Kohn, Robyn
- **Document Date :** 2011 July 07
- **Type :** email; report
- **ID :** tygg0230 ( TID : vmf71j00 )
- **ARK :** ark:/88122/tygg0230
- **Collection :** Oklahoma Opioid Litigation Documents; Opioid Documents Collection

Document Notes

- SEE # 103 – this is an example of the Policy Monthly Briefing proposed by Kohn
- Primary External Partners
  - GO TO PARTNER = **American Pain Foundation**
  - American Academy of Pain Medicine
  - American Academy of Pain Management
  - American Chronic Pain Association
- Secondary External Partners
  - American Academy of Hospice & Palliative Care Medicine
  - American Academy of Family Physicians
  - American Geriatric Society
  - American Society of Consultant Pharmacists
  - American Academy of Physician Assistants
  - American Academy of Nurse Practitioners
- **KEY ADVOCACY THEMES**
  - Marketplace
    - **Significant unmet needs in moderate-severe chronic pain market**
    - Advocacy on the local market level gaining momentum among partner org
  - Pain Tools
    - **Prescribe Responsibly**-external HCP community
    - **Smart Moves, Smart Choices**-Teen RX Abuse
    - **Let's Talk Pain**-Provider/Patient Communications
- **NEW INITIATIVES**
  - **Imagine the Possibilities – Pain Coalition**
    - Internal cross-functional members of the pain teams and external members of the pain communities
    - Goal: align and address issues in pain management with emphasis on abuse and diversion
  - **New Partnership: State Pain Policy Action Coalition (SPPAC)-AAPM, APF, ASPMN**
    - Goal: Formed out of need to respond and influence positive state-based public policies is crucial to assuring access to effective care for persons experiencing pain.
    - **National pain management organizations have attempted to influence** many of these issues, but often have done so in an ineffective piecemeal and hit-or-miss fashion, failing to take advantage of opportunities to **speak with one voice and achieve desired outcomes.** SPPAC provides the opportunity to create a synergistic voice to insure the ability of our members to provide and receive optimal care for pain.
    - While three organizations are initially organizing the coalition, other organizations will be invited to join once the structure is developed.
- IOM REPORT (6/29/11): **Reliving Pain in America**
  - What is the IOM report: The report, released on June 30, 2011 — Relieving Pain in America: A Blueprint for Transforming Prevention, Care, Education, and Research — calls for a cultural transformation of attitudes toward pain and its prevention and management.
  - Noteworthy findings in IOM
    - Chronic pain affects an estimated **116 million** American adults
    - Pain costs the nation up to **$635 billion each year** in medical treatments and lost productivity.
    - Chronic pain negatively affects socioeconomic status.
    - Federal and state drug abuse prevention laws, regulations, and enforcement practices have been considered impediments to effective pain management...." Among other barriers, they say "**Twentynine percent of primary care physicians and 16 percent of pain specialists report they prescribe opioids less often than they think appropriate because of concerns about regulatory repercussions."**
  - Partner Commentary
    - **Pain Care Task Force** is being formed to develop a communication and outreach plan for dissemination of key messaging across national/local markets. The activities can serve as an advocacy tool for our partner organizations.
    - The committee calls for government agencies, healthcare providers, and public and private funders of health care to adopt a comprehensive, strategic approach to reduce or eliminate the barriers to pain care.

110) Speaker Agreement

Document Data

- **Author :** Cephalon, Inc; Repella, Robert
- **Document Date :** 2011 May 06
- **Type :** agreement
- **ID :** lfhg0230 ( TID : vqf71j00 )
- **ARK :** ark:/88122/lfhg0230
- **Collection :** Oklahoma Opioid Litigation Documents; Opioid Documents Collection

Document Notes

- APRIL VALLERAND = **Contracted speaker with Cephalon**
  - Also one of the authors of Chronic Pain Management Strategies and Lessons from the Military
- Contract between April Vallerand and Cephalon
  - Cephalon = FENTORA (fentanyl)
- Services
  - a. Cephalon may engage Speaker to conduct presentations at Cephalon CSP (Cephalon Speaker Programs)
- Compensation
  - Cephalon shall pay Speaker a fee in accordance with honoraria guidelines established by Cephalon and described in Exhibit C (Compensation and Training).
  - The parties agree that the compensation provided hereunder has been established pursuant to aims length negotiations between the parties and is consistent with the fair market value of the services provided by Speaker under this Agreement
- Standards
  - Speaker will have access to a Cephalon-approved slide kit. Speaker must present this slide kit. Speaker must never use their own slides or other presentation materials for any purpose during the course of a CSP
  - Product-Specific Questions
    - In response to an "on-Iabel" question in the Q&A session. Speaker may answer the question verbally and/or use the Cephalon approved promotional slides
    - In response to an "off-label" question in the Q&A session, Speaker must not display or distribute any slides or other materials, but rather, if Speaker chooses, may provide a verbal answer to the question
    - Speaker's answer to questions must present the information in an accurate, fair and balanced, and objective manner, and must disclose the basis for answering the question (e.g. personal clinical experience, or data from a clinical study).
- Confidentiality. Speaker agrees to maintain in confidence, beyond the termination of this Agreement, and to not disclose to any third partly the terms of this Agreement, any information provided to Speaker by Cephalon under 'litis Agreement, as well as any password(s) that can be used to access any Cephalon Speaker Bureau web site. Speaker also agrees not to provide copies of the Cephalon slide kits to any third party, including any other pharmaceutical ( company.
- The Epidemic of Pain in America
  - [https://www.industrydocuments.ucsf.edu/docs/yqgg0230](https://www.industrydocuments.ucsf.edu/drug/docs/#id=yqgg0230)
  - Author : American Pain Foundation, The; Rogers, Mike
  - Document Date : 2006 June 13
  - Type : report
  - ID : yqgg0230 ( TID : wnf71j00 )
  - ARK : ark:/88122/yqgg0230
  - Collection : Oklahoma Opioid Litigation Documents; Opioid Documents Collection
  - Key Points:
    - Extensive report (133 pages) supporting the concept of undertreatment of pain
- Duragesic – 2003 Business Plan Summary
  - [https://www.industrydocuments.ucsf.edu/docs/sngg0230](https://www.industrydocuments.ucsf.edu/drug/docs/#id=sngg0230)
  - Author : Janssen
  - Document Date : Unknown
  - Type : report
  - ID : sngg0230 ( TID : emf71j00 )
  - ARK : ark:/88122/sngg0230
  - Collection : Oklahoma Opioid Litigation Documents; Opioid Documents Collection
  - Key Points:
    - VA/DOD – strategic customer segment
- Chronic Pain Management Strategies and Lessons from the Military
  - [https://www.industrydocuments.ucsf.edu/docs/kqgg0230](https://www.industrydocuments.ucsf.edu/drug/docs/#id=kqgg0230)
  - Author : Wayne State University; Vallerand, April Hazard; Cosler, Patricia; Henningfield, Jack E; Galassin, Pam
  - Document Date : Unknown
  - Type : bibliography; flow chart; graph; report
  - ID : kqgg0230 ( TID : snf71j00 )
  - ARK : ark:/88122/kqgg0230
  - Collection : Oklahoma Opioid Litigation Documents; Opioid Documents Collection
  - Key Points:
    - Confidential
    - “Editorial support for the writing of this article was provided by Megan Knagge, PhD, of MedErgy, and was funded by Janssen Scientific Affairs, LLC. The authors retained full editorial control over the content of the article.”
    - “P. Cosler was an employee of Janssen Scientific Affairs, LLC, at the rime of the writing of this manuscript and declares no current conflicts of interest.”
    - J. E. Henningfield provides consulting services through Pinney Associates to pharmaceutical developers and marketers of CNS acting drugs, including analgesics, to help assess the abuse liability of the drugs and develop appropriate recommendations for labeling and risk management to minimize the risks of abuse, overdose, and other unintended effects.”

182) Non Branded Round 2 – Final Report Review

Document Data

- Author : Jones, Susan T
- Document Date : 2008 April 10
- Type : email; notes
- ID : lmgg0230 ( TID : hlf71j00 )
- ARK : ark:/88122/lmgg0230
- Collection : Oklahoma Opioid Litigation Documents; Opioid Documents Collection

Document Notes

- **Pain Non-Branded Campaign Market Research – Round 2**
  - The Campaign = Non-Branded Message regarding the under treatment of pain, why so and what can lead to
  - The P3 Program = **Physicians Partnering Against Pain**
  - The Product = Tapentadol IR (NUCYNTA)
- **Market Research Objectives =** Test the non-branded campaign's ability to "impact" the market place behaviors/thinking relative to the treatment &. management of acute pain
  - To Explore physicians’ reactions to four messages presented by a sales representative
  - To understand the impact of the message regarding the undertreatment of acute pain and its implications
  - To understand the connection of educational campaign to the Tapentadol TPP (Triplicate prescription program???)
- **THE MESSAGES – RECAP (see screenshots)**
  - Message = Undertreatment of Pain
    - Behavioral Change = will attempt better assessment of acute pain
  - Message = Consequences
    - Behavioral Change = **More aggressive approach to treating (stronger dosing and meds);** desire to spread the message to other physicians
  - Message = Concerns that Hinder Effective Pain Mgmt
    - Behavioral change = Low abuse numbers
    - About half believe the addiction percentage (For the most part, the Rep was successful with refocusing them from addiction to side effect concerns)
  - Message = Multi-Pathways
    - NEW AGENTS – we have a new solution
    - Behavioral Change = **Increased opioid use (PCP), increase use of poly pharmacy until Product Y becomes available**
- **Impact of Campaign Overall =** PCPs state that they will be more aggressive in their treatment and use more opioids
- Physicians offer the following advice and suggestions on how to better present the educational campaign regarding acute pain management
  - Include thought leader presentations and peer-to-peer discussions in the campaign
- Reactions to Tapentadol IR TPP
  - Other benefits include efficacy similar to oxycodone, low withdrawal potential, **and some physicians on their own made a leap that it may result in lower addiction potential**
- Physicians report many patient types as undertreatment. These include: **the elderly**, **younger patients**, post-op and **post-trauma patients**
  - RECOMMENDATION = Include these patients in the undertreatment message

1. The Epidemic of Pain in America
   - [https://www.industrydocuments.ucsf.edu/docs/yqgg0230](https://www.industrydocuments.ucsf.edu/drug/docs/#id=yqgg0230)
   - Author : American Pain Foundation, The; Rogers, Mike
   - Document Date : 2006 June 13
   - Type : report
   - ID : yqgg0230 ( TID : wnf71j00 )
   - ARK : ark:/88122/yqgg0230
   - Collection : Oklahoma Opioid Litigation Documents; Opioid Documents Collection
   - Key Points:
     1. Extensive report (133 pages) supporting the concept of undertreatment of pain
     2. AUTHORS
        1. Howard Heit – paid consultant for Cephalon and J&J ([link](https://projects.propublica.org/d4d-archive/search?utf8=%E2%9C%93&term=howard+heit&state%5Bid%5D=47&company%5Bid%5D=&period%5B%5D=&services%5B%5D=))
        2. PAIN CARE FORUM + Representative Mike Rogers (R-MI)
        3. Presented at the Cannon House Office Building, Washington DC
     3. Interesting Tidbits
        1. Pain affects people at all stages of life - including infants, children, young adults, and the elderly.
        2. For one third of sufferers, their chronic pain was so severe and debilitating, they felt they couldn't function as normal people and sometimes felt so bad they wanted to die.
        3. 2/3 of older Americans who take pain medications said pain still prevents them from performing routine tasks, engaging in hobbies, or doing things they enjoy. Of these, 44% said they had difficulty Walking .
     4. Personal Stories of Pain
        1. Alexandra, sixteen years old (patient w/RSD)
           1. At this moment I am trying to start a program at the local hospital for kids facing chronic pain problems and giving them support and just a friend.
        2. Jim, US Military Veteran
           1. For years my pain was ignored. I was labeled as a drug seeker. The Veterans Hospitals would never address the real cause
           2. They [VA] didn't know how to treat what they created
           3. This disease has no cure but to take pain medication.
           4. I have had to fight the Veterans Heath Care System for years
     5. The pain gap – minorities in America are considered “under treated for their pain from medical conditions”
        1. Donald Dillard counted himself lucky to survive when a loaded dump truck rolled over his body, crushing his pelvis and thighs and shredding a football-size chunk of flesh in his torso. But the 15 years since have been a nightmare of agonizing days and nights made worse by the lack of potent pain medications at pharmacies near his home, struggles to get proper care and the red tape involved in workers' compensation coverage
        2. Hispanics and African Americans are consistently under-treated for pain across a range of conditions, from cancer to chest pain to broken bones
        3. "There are many people not getting treatment for their chronic pain, and race is part of the equation."
        4. Just as important, Staats added, is that pain management is not fully recognized as a board specialty by the American Board of Medical Specialties, "But there are a group of like-minded doctors who recognize that there is disease of chronic pain," said Staats, who serves on the board of directors of the American Academy of Pain Medicine. "Chronic pain affects a person in so many different ways — physical, psychological, emotional
     6. Professional Education – Fast Facts
        1. Most front-line family practitioners and specialists have very little training or education in identifying and treating the various distinct kinds of pain conditions.
        2. Despite the fact that pain is the number one condition that brings people to the doctor, pain education is barely present in most medical school curricula.
        3. **There are millions of people living with pain who are told by healthcare professionals that opioid medicines, one of the most powerful and effective class of medicines, are addictive and shouldn't he used.**
        4. Each year there are millions of people near death and in severe pain when there are effective pain medicines and treatments available to relieve them of their pain.
        5. **Health professionals and the public are unaware:** • Physical dependence on a medication is not the same as addiction. • Appropriate use of opioid medications (like oxycodone) is safe and effective and unlikely to cause addiction in people who are under the care of a doctor and who have no history of substance abuse. • Opioid medications are sometimes the only effective treatment for some types of pain.
        6. The typical healthcare practitioner is both time-challenged and **clueless** with regard to the treatment of pain,
        7. Americans come to their healthcare professionals expecting to be cared for, to have their pain relieved, but due to practitioner ignorance about the significance of untreated or undertreated pain, and the possibility of prosecution, they opt to leave patients in pain as the "safest course."
     7. Balancing the Need to Curb Diversion of Pain Medicines and the Need to Protect Access to Pain Medicines
        1. In most communities it is difficult for pain patients to find physicians willing to prescribe opioid medicines for pain.
        2. There is a need to reform overly restrictive regulatory polices which impede pain relief.
     8. PITCHING RELIEF – Firsthand account from Dr. Heit (paid consultant)
        1. Heit, 61, doesn't use prescription opioids for his own pain now, but he does prescribe them in high doses to many of his patients, and he's seen the drugs (in conjunction with proper monitoring) provide remarkable relief ~ the kind he still wishes he had had available back when he really needed it. As the showdown between pain doctors and prosecutors stiffened several years ago, he felt obliged to get more actively involved in defense of opioid treatment despite the potential risk to his practice
        2. Shugol, 54, wheeled herself into Heit's Arlington Boulevard office two weeks ago for a monthly appointment, smiling broadly and filled with an energy seldom seen in people who don't carry her many physical burdens. The first order of business was, as always, to hand Heit her vials of drugs, so he could see exactly how many pills she had used since the last visit. Heit took out a pill counter and went to work, first on the OxyContin, and then the Dilaudid. He was puzzled to find more than 100 extra pills. "Have you been taking everything you need?" he asked. "Yep, but I think you made a mistake last time," she replied. Rather than writing a prescription for 230 pills, Heit had written one for 330 pills, and that's what the pharmacist filled. As Shugol continued to talk of the active life she can now have because of the opioids and her care by Heit, the doctor went through the detailed paperwork he keeps on all patients.
        3. Shugol had followed Kathryn Brock of Reston — another woman in a wheelchair with an easy smile and a strong desire to remain active - into Heit's office. Brock sufferers from rheumatoid arthritis in virtually every joint in her body, and she, too, is subject to constant pain. She says that her regimen of six OxyContin and eight Dilaudid pills a day has kept her marriage going, and gives her the ability to continue painting, which she does regularly
        4. **The Gift of a Baby** A fourth patient seeing Heit earlier this month was a young woman who had fallen down a flight of stairs at a nightclub and mptured two disks in her neck. Another jock accustomed to playing with pain, she tried to gut her way through it. Doctors recommended surgery, but she resisted. Her boyfriend recommended Heit, and she began treatment. She improved markedly, married the boyfriend and, while still on OxyContin, got pregnant. She knew she could never get through the pregnancy without the medication, but she was concerned that the child could be in withdrawal at birth. When the baby was bom in 2004, Heit was at the hospital to examine the newborn for signs of opioid trouble, and was relieved to find none. Heit is the kind of person who likes to connect quickly and deeply to others, and so it wasn't entirely unexpected that he would be in the hospital. But there was another reason for his presence. The new mother, whom Heit had received permission from Virginia medical authorities to treat, was Jamie Heit ~ his son David's wife. And the healthy child, bom to a woman who wasn't sure she could ever carry a baby after her fall, was his first granddaughter, Lilly.
   - Empowerment – WOMEN
     1. Create patient demand for proper pain diagnosis and treatment; encourage patients to advocate for their rights
     2. Consider vital tactics/word of mouth education – let women educate women in their natural setting **(Tupperware parties, garden groups, e-cards)**
     3. Conduct media roundtable w/ women’s books; could work with additional partner with a female focus (like the National Women’s Health Resource Center)
     4. Consider using applicable information from the “Savvy Woman Patient” chapter on women & pain
   - CHRONIC PAIN HITS WOMEN HARDER – Jennifer Wider, Society for Women’s Health Research
   - Provide visuals of multiple patients (woman, man, child, elderly person) to help them find their fit
     1. Each visual could contain descriptions to help patients identify
   - Flyer from Research!America = **Facts about Pain – Investment in research saves lives and money**
     1. Pain Today
        1. As many as 20% of children experience chronic pain
        2. Inadequate pain management is more severe problem for ethnic and racial minorities than for other population groups
     2. The Bottom Line
        1. In a recent Research!America public opinion survey, 57% of Americans reported experiencing chronic or recurrent pain. Sixty-six percent of those in pain expect to live with it for the rest of their lives. Americans want and expect a sustained investment in research to alleviate the burdens caused by chronic pain.
   - **When it comes to severe pain, doctors still have much to learn**
     1. A Painful lesson
        1. I called the surgeon's office weekly and reported my minimal progress in pain control, but at no point was an increase in pain medication suggested, nor was I referred to a pain management specialist on the hospital staff
        2. When, at seven weeks after surgery, I spoke to Dr. Schneider, a Tucson-based specialist in pain management and addiction medicine, she chastised me for not being more insistent about getting adequate pain relief. The trouble is, when you're experiencing intense pain, it's hard to be proactive about anything
        3. Many doctors are afraid to prescribe narcotic drugs like oxycodone, fearing they will create addiction problems. But that in fact rarely happens to chronic pain patients who don't have a history of addiction. When a pain patient needs increasing doses of a narcotic, it's nearly always because the pain worsens, as often happens in patients with advanced cancer. Patients do become tolerant to side effects, like grogginess, but rarely to the pain-relieving properties of these drugs.
     2. When the Nerves Respond
        1. When I read this,! realized I was on the wrong track, taking too little of the long-acting drug and too much of the short-acting one
        2. As it turned out, my internist knew far more than my surgeon about treating pain. He has many elderiy patients with chronic pain and knows very well how to treat it. I realize now I should have sought his help from the beginning. Or I should have asked to be referred to a pain management specialist at the hospital where I had my surgery
     3. Let’s Fix What’s Broken
        1. First and foremost, patients need to be proactive and insist on the help they need. If patients are not able to do this for themselves, an advocate should do it for them. Second, every person with prolonged or chronic pain should become educated about the huge range of medications, therapies and complementary remedies available to treat pain.
   - **Painful Choices: Physicians challenged by quest to end suffering**
     1. Nursing home patients have historically been some of the most undertreated for pain. The complaints of fragile elderly are sometimes dismissed as "normal aging," It's even worse, says Community Nursing Services hospice director Dr. Anna Beck, for those who are too weak or confused to communicate
     2. When it comes to severe pain, it's called "pain management'' for a reason, because some pain is so awful that it can't entirely be erased. Marcia Solum is thrilled to now have pain she can live with, Before, when her pain was unbearable, she would sometimes take eight Advil every four hours. "I was trying to be able to have a life, and I just couldn't," she says, her voice breaking. Now on a morphine pump, she can get out of bed each day, teach sewing classes, have 25 people over for Thanksgiving dinner.
   - **PREVALENCE: Fast Facts**
     1. For American military, the current way in Iraq is resulting in a significant number of body and limb injuries which often lead to life-long chronic pain conditions


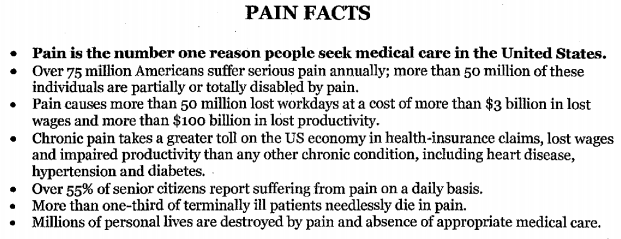


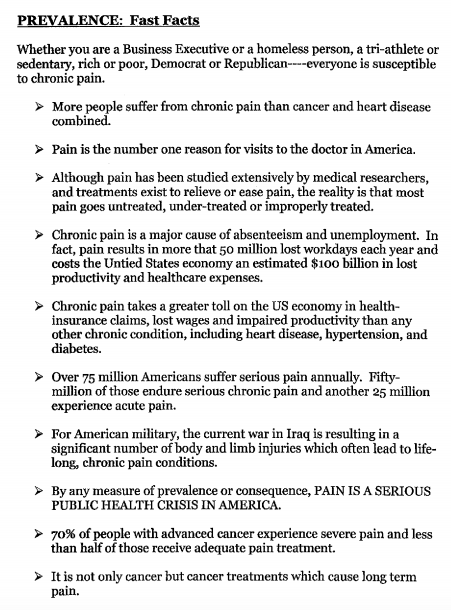


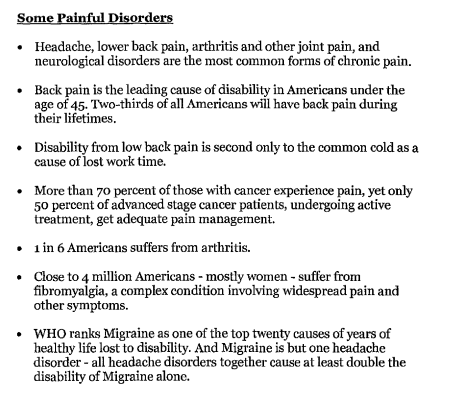


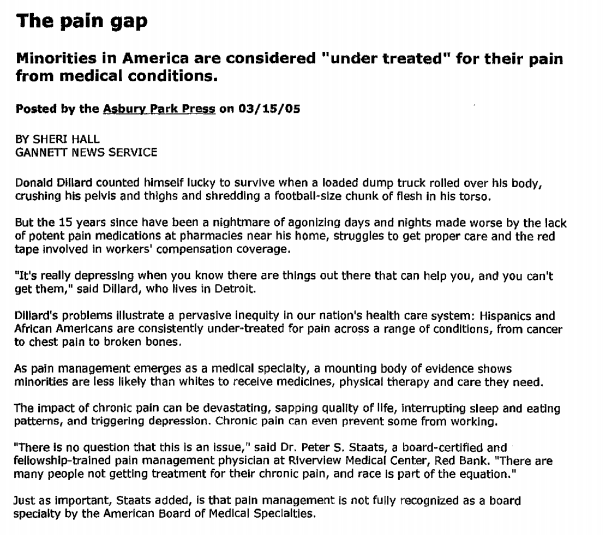


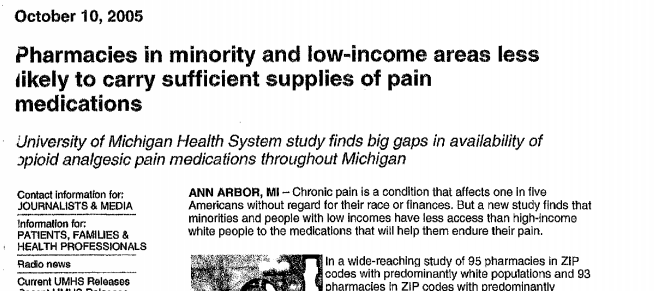


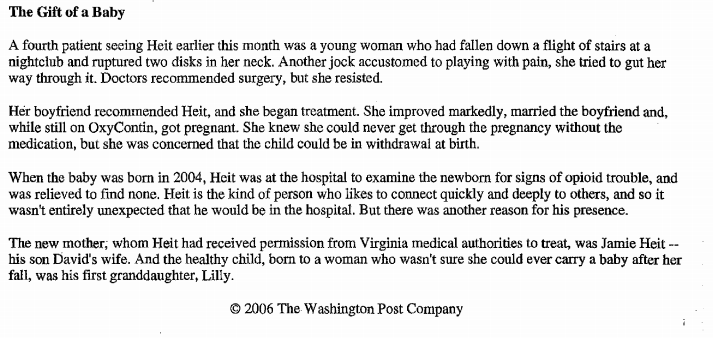


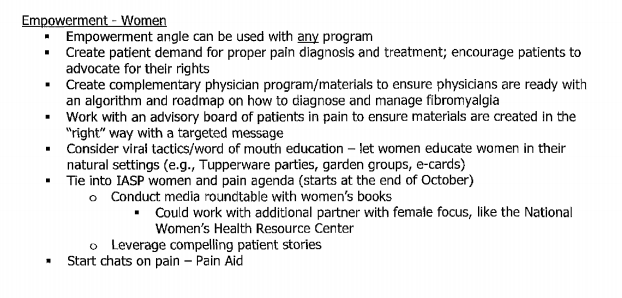


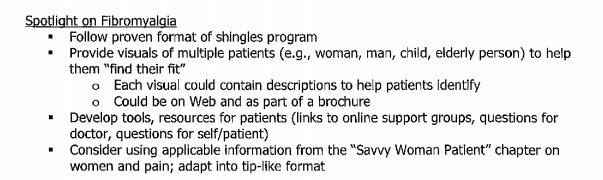


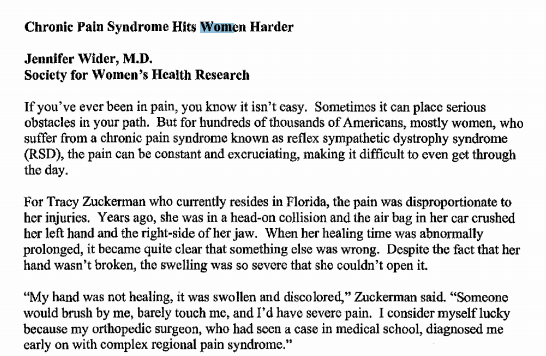


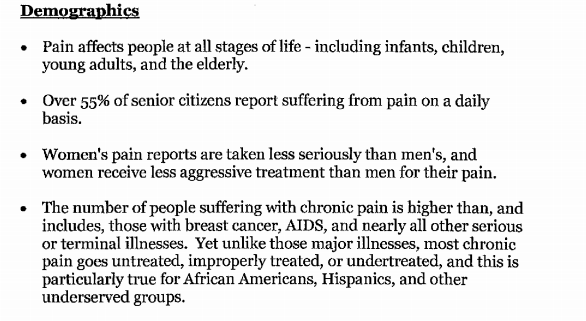


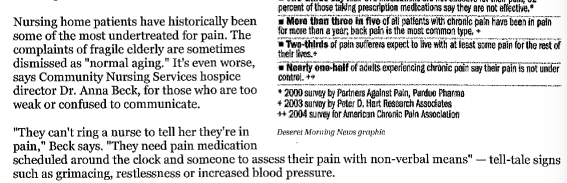


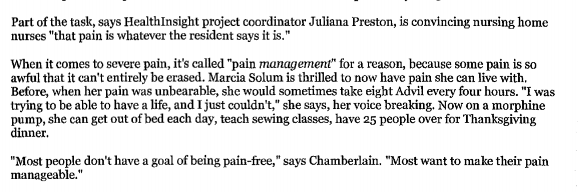


187) Email re: The Pain Care Forum

Document Data

- Author : Udell, Howard; Purdue Pharma
- Document Date : 2008 March 14
- Type : email
- ID : rygg0230 ( TID : tmf71j00 )
- ARK : ark:/88122/rygg0230
- Collection : Oklahoma Opioid Litigation Documents; Opioid Documents Collection

Document Notes

- Congressman Loebsack met with members of the Pain Care Forum regarding the Military Pain Care Act
  - PCF is working with Loebsack to get cosponsors for the Military Pain Care Act
  - PCF is having other pain organizations send letters of support to Ranking Members Hunger and Skelton (Armed Services Committee)
- Howard Udell, Executive Vice President, Chief Legal Officer @ Purdue
  - **“That’s just great. The PCF has become a ‘force’ to be courted by members of congress”**
- Hospital Sales Force Cycle III Presentation
  - [https://www.industrydocuments.ucsf.edu/docs/gtgg0230](https://www.industrydocuments.ucsf.edu/drug/docs/#id=gtgg0230)
  - Author : Unknown
  - Document Date : 2002 September 17
  - Type : chart; graph; photograph; publication
  - ID : gtgg0230 ( TID : kpf71j00 )
  - ARK : ark:/88122/gtgg0230
  - Collection : Oklahoma Opioid Litigation Documents; Opioid Documents Collection

**ELDERLY**

13) Director of Stakeholders – Business Plan

- Document Data
  - **Author :** *Unknown*
  - **Document Date :** 2019 January 24
  - **Type :** presentation; slides; report
  - **ID :** nlgg0230 ( TID : tkf71j00 )
  - **ARK :** ark:/88122/nlgg0230
  - **Collection :** Oklahoma Opioid Litigation Documents; Opioid Documents Collection
- Document Notes
  - Challenge = fear of opioids --> Strategy = create awareness of under treated pain
  - Challenge = Media perception of opioids as highly addictive --> **Leverage advocacy groups and KOLs to help the media beter understand, the nature of addiciton, health risk of untreated pain**
  - TACTIC = ADVOCACY GROUPS
    - Find which advocacy groups serve high-risk patients (high risk = suffering from effects of under treated pain)
    - Develop targeted relationships with advocacy groups to ensure that key pain message is embraced **AND REFLECED IN GUIDELINES**
  - TACTIC = POLICY
    - Partner with internal government affairs group and the Alliance of State Pain Initiatives to map the regulatory environment as it relates to pain management
    - Partner with government affairs to create advocacy plan
- Document Relevance? Yes

81) 2013 National Advocacy Business Planning

Document Data

- Author : Johnson and Johnson; Janssen Pharmaceutical
- Document Date : 2012 June 29
- Type : proposal
- ID : zggg0230 ( TID : yhf71j00 )
- ARK : ark:/88122/zggg0230

Document Notes

- Business planning from Janssen (PAIN FRANCHISE) June 29, 2012
- Key Questions = How to leverage sales of Nucynta??
- 2013 PAIN Advocacy Strategy
  - Engage partners to embrace the IOM report-national/state implications advocate for and act collectively to actualize the recommendations
  - **Influence agencies that impact policy and quality to maintain or improve access**
- Barriers to care
  - FOCUS: **Engage with advocacy partners** at the national level for greater impact and alignment. Support models for collaboration and synergies that can then be applied at a regional level
  - Chronic pain can and should be thought of as a disease in and of itself.
  - Public health and community-based approaches are required to **address the under-treatment of chronic pain**
- Advocacy/Policy Focus
  - Provide state and federal legislators and other regulatory groups with access to objective materials to assist them in making public policy.
  - Collaborate with the **Pain Care Forum (PCF)** on policy issues and common strategies with key decision makers; such as HHS, Surgeon General's Office, CDC, state and federal legislators and regulators. •
  - Collaborate with State Pain Policy Action Coalition (SPPAC), a newly formed organization made up of pain focused organizations that will pro-actively inform and influence state policy . •
  - **Sponsor Public Awareness campaigns targeted at preventing chronic pain and misuse of prescription pain medications. •**
  - **Sponsor disease awareness to promote balanced and effective pain management. •**
  - Support collaboration between Medicine and Law Enforcement to prevent the "chilling effect".
    - [Chilling effect](https://oxfordmedicine.com/view/10.1093/med/9780190659721.001.0001/med-9780190659721-chapter-10) ([link](https://oxfordmedicine.com/view/10.1093/med/9780190659721.001.0001/med-9780190659721-chapter-10)) = An overzealous Drug Enforcement Administration is sometimes prosecuting the wrong physicians, thus creating a chilling effect in the medical community with regard to opioid prescription and making it harder for people in pain to get the help they need
  - Support effective Prescription Monitoring Programs (PMPs) that provide prescribing healthcare professionals with "real time access" and improve patient care.

120) Prescribe Responsibility Copy & Insert: Pain Resource Guide

Document Data

Document Notes

- REMINDER: **Prescribe Responsibility (PR) is one of three unbranded initiative programs started by Janssen regarding pain; PR is aimed at prescribers (**[**LINK**](https://www.multivu.com/assets/51908/documents/51164-Janssen-Responsibility-Fact-Sheet-original.pdf)**)**
- Email from Laura Flannery to Kimberely Deem-Eshleman
  - Laura Flannery = Project Manager @ Decile.Ten (Communication/Marketing/Ad company for pharma)
  - Kimberely Deem-Eshleman = Regional Business Direction @ Jansen
- QUICK SUMMARY --- Decile.Ten created a resource guide to be placed in Prescribe Responsibility binder (unbranded initiative for Jansenn); the resource guide is named Pain Resource Guide: Getting the Help You Need, and it is guide given out by the (**American Pain Foundation**)
  - It contains useful and important information about pain and advice to help patients get the quality pain care they deserve. This guide will help answer patients' questions and point them in the right direction. It will provide an understanding on:
  - • **Why pain management is important • How to find effective pain care and pain specialists** • How pain is measured and diagnosed • Goals of treatment and available treatment options • Questions to ask your healthcare provider • Steps you can take to reclaim your life
- American Pain Foundation – Pain Resource Guide: Getting the Help you Need
  - How to use this guide: his booklet is designed to help you take charge of your pain care. It provides important information about pain and tips to help you get the quality pain care you deserve. Whether you've just started experiencing pain or have lived with it for years, you are bound to have lots of questions. We hope this booklet will help answer many of these questions, and point you in the right direction.
  - IF you’re not satisfied with your pain care, don’t give up
    - Does your healthcare provider listen to you7 Is he or 'she ab'e to assess and treat your pain7 If after a reasonable time the answer is "''no," "ask for a referral to a pain specialist oi other healthcare provider Finding the right provider is one of the most important decisions you can make
  - Setting the record straight on addiction
    - Many people living with pain -- and even some healthcare providers – falsely believe opioids are universally addictive.
    - Studies have shown that the risk of addiction is small...
    - Opioids provide needed relief for many patients ..
    - Physical dependence – which is not addiction – may occur and cause withdrawal symptoms if you stop taking these medications suddenly
    - Unless you have a past or current history of substance abuse, the chance of addiction is low
    - Still, those who suffer with chronic pain and a substance abuse problem deserve the same quality of pain treatment as others
- The Epidemic of Pain in America
  - [https://www.industrydocuments.ucsf.edu/docs/yqgg0230](https://www.industrydocuments.ucsf.edu/drug/docs/#id=yqgg0230)
  - Author : American Pain Foundation, The; Rogers, Mike
  - Document Date : 2006 June 13
  - Type : report
  - ID : yqgg0230 ( TID : wnf71j00 )
  - ARK : ark:/88122/yqgg0230
  - Collection : Oklahoma Opioid Litigation Documents; Opioid Documents Collection
  - Key Points:
    - Extensive report (133 pages) supporting the concept of undertreatment of pain
- The Prescription Opioid and Heroin Crisis
  - [https://www.industrydocuments.ucsf.edu/docs/jtgg0230](https://www.industrydocuments.ucsf.edu/drug/docs/#id=jtgg0230)
  - Author : Kolodny, Andrew; Courtwright, David T; Hwang, Catherine S; Kreiner, Peter; Eadie, John L; Clark, Thomas W; Alexander, G Caleb
  - Document Date : Unknown
  - Type : article; report
  - ID : jtgg0230 ( TID : npf71j00 )
  - ARK : ark:/88122/jtgg0230
  - Collection : Oklahoma Opioid Litigation Documents; Opioid Documents Collection
- Duragesic – 2003 Business Plan Summary
  - [https://www.industrydocuments.ucsf.edu/docs/sngg0230](https://www.industrydocuments.ucsf.edu/drug/docs/#id=sngg0230)
  - Author : Janssen
  - Document Date : Unknown
  - Type : report
  - ID : sngg0230 ( TID : emf71j00 )
  - ARK : ark:/88122/sngg0230
  - Collection : Oklahoma Opioid Litigation Documents; Opioid Documents Collection
  - Key Points:
    - Long term care/elderly patients – strategic customer segment

132) Permission to Reproduce APF Materials

Document Data

- **Author :** Weissfeld, Bonnie; American Pain Foundation
- **Document Date :** 2010 December 06
- **Type :** email; report
- **ID :** xngg0230 ( TID : ulf71j00 )
- **ARK :** ark:/88122/xngg0230
- **Collection :** Oklahoma Opioid Litigation Documents; Opioid Documents Collection

Document Notes

- Email from Bonnie Weissfeld (Assistant to the CEO, American Pain Foundation)
  - Dear David, Per your request to reproduce the publications noted below, permission is granted.
    - Target Chronic Pain Notebook
    - Target Chronic Pain Provider Card
    - Top 10 Tips: Making the Most of Your Medical Visits (tip sheet)
- Brochure: **Chronic Pain – Intermittent, Persistent, Breakthrough**
  - Information about Opioids
    - Treating patients with opioids – **when prescribed by a HCP and taken as directed, opioids are safe, effective, and rarely lead to addiction**
  - Responsible Opioid Prescribing: A Physician’s Guide – written by Scott Fishman, provides strategies to reduce the risk of addiction, abuse, and diversion
    - SEE #6 – book is heavily funded by AFP, other advocacy orgs, pharma
- Brochure: **Target Chronic Pain**
  - Suggestions for reaching target goals
    - Accept your patient’s reports of pain; assess chronic pain as part of each visit; look for underlying causes of pain;

177) Finding Relief: Pain Management for Older Adults – Final Video Script

Document Data

- Author : Johnson and Johnson
- Document Date : 2008 December 29
- Type : transcript; notes
- ID : hjgg0230 ( TID : hjf71j00 )
- ARK : ark:/88122/hjgg0230
- Collection : Oklahoma Opioid Litigation Documents; Opioid Documents Collection

Document Notes

- Link to current video: ([LINK](http://centersforpain.com/news/-Finding-Relief-Pain-Management-for-Older-Adults))
  - Click here to view the video courtesy of **American Academy of Pain Medicine.**
  - Actress = Kathy Baker
- The sponsor (**PriCara®)**, the producers (Conrad Productions and Aiari Weiss Productions), the scriptwriter (Deborah Gobble), and the partners (**American Academy of Pain Medicine, American Geriatrics Society, and American Geriatrics Society Foundation for Health in Aging**) have used reasonable efforts to include timely and accurate information in this program. Accordingly, the sponsor, producers, writer, and partners make no representations or warranties, express or implied, regarding the accuracy or completeness of the information provided herein and specifically disclaim any liability, express or implied, in connection therewith
- Pain, it's the #1 reason people go to the doctor.(1) Yet many people don't get the adequate treatment they need. (2) The pain may get worse, or even lead to other problems. That doesn't have to happen.
- Dr Bruce Ferrell: 1 think in the past, physicians oftentimes overlooked the problem of pain. In their effort to try to treat the underlying cause of the pain - they often overlooked the pain itself
- **With effective treatment, Ted has been able to have a full career and an active retirement.** But many people just Suffer with pain that is untreated, of under treated. The effects can be devastating.
- Dr Rollin Gallagher: After a long time of having chronic pain, you may not be able to socialize with your friends, do your hobbies, make love to your spouse, play with your kids or grandchildren. So it has. a universally bad effect en peoples' lives.
- Dr Rollin Gallagher: Opioid analgesics are a cornerstone of pain management. Both acute pain management and chronic pain management.
  - Medicines containing opioids have been used for centuries. They’re strong pain medicines for moderate to severe pain. Today, they come in many forms and strengths. But all opioids require RX
    - NOTE: *The video first talks poorly about APAP, Aspirin, NSAIDs then starts talking positively about opioids*
- Dr Bruce Ferrell: These drugs are actually very effective, espicially for patients with moderate to severe pain. And oftentimes, their fears of these medications are probably over-emphasized or out of proportion to the real risk involved in their use.
  - Used properly, opioid medications may make it possible for people w/ chronic pain to return to a high quality, more functional life.
- Background info on doctors involved in this video
  - Bruce Ferrell – UCLA
  - Rollin Gallagher – VA Medical Center
    - President of AAPM
  - Debra Weiner – prof of medicine at University of Pittsburgh

179) Pain District Hub Meetings

Document Data

- Author : Cho, Chris
- Document Date : 2008 October 22
- Type : memo
- ID : fygg0230 ( TID : hmf71j00 )
- ARK : ark:/88122/fygg0230
- Collection : Oklahoma Opioid Litigation Documents; Opioid Documents Collection

Document Notes

- Email from Chris Wanki Cho, Manager at Janssen
  - Neopathways Trademark by Johnson & Johnson ([LINK](https://trademarks.justia.com/775/26/neo-pathways-in-pain-77526871.html)) -- printed publication, education
- Title = **NEO Pathways Module 2: Consequence of Unresolved Acute Pain**
  - Goals
    - Underscore the urgency of achieving effective relief of moderate to severe pain as soon as possible • Help HCPs make the connection between the undertreatment of acute pain and the risk of developing chronic pain • Support the choice of appropriate analgesics for moderate to severe pain
  - Key Messages
    - The onset of chronic pain is often related to unresolved acute pain from injury or surgery
    - Change the course...**through early and aggressive intervention**
  - Pilot Learning
    - Its about Acute Pain that could develop into Chronic Pain • Do not go into depth pertaining to Neuroplasticity (???)
- Title = **NEO Pathways Module 3: Barriers to Optimal Management of Mod to Severe Pain**
  - Goals
    - Help overcome exaggerated concerns about the risk of disciplinary or legal action for physicians who are prescribing opioids appropriately in the treatment of moderate to severe pain
  - Key Messages
    - For many patients, the desire to avoid opioid side effects can be more important than pain control
    - Although many physicians are reluctant to prescribe controlled substances, the risks (for both patient addiction and physician disciplinary action) are much smaller than commonly believed
  - Execution Tips
    - **Avoid the ditch- It's not about the %, but about addiction as a barrier contributing to the undertreatment of pain**
    - • Know the difference between addiction, dependence, and tolerance

182) Non Branded Round 2 – Final Report Review

Document Data

- Author : Jones, Susan T
- Document Date : 2008 April 10
- Type : email; notes
- ID : lmgg0230 ( TID : hlf71j00 )
- ARK : ark:/88122/lmgg0230
- Collection : Oklahoma Opioid Litigation Documents; Opioid Documents Collection
  - Key Points:
    - The American Academy of Pain Medicine, American Geriatrics Society
    - Sponsored by PriCara (Janssen)
    - “This brochure and DVD are aimed specifically at older adults and what they need to know to get effective pain relief. You will learn about your options for pain management and how to talk to your doctor about finding a treatment that’s right for you. By learning more about pain and the many ways it can be treated, you are taking solid steps toward reducing the pain you or a loved one may be feeling.”
    - “Pain is the body’s alarm system.”
    - “That’s why most doctors say that ‘pain is what the patient says it is.’ In addition, sometimes a physical cause of pain cannot be found. Some people worry that a doctor will think their pain is ‘all in their head.’ Most doctors know better. They will take your pain seriously and work hard to find a solution.”
    - “You may be asked”
      - Where does it hurt?
      - Does the pain move from place to place?
      - Do you have pain in more than one place?
      - When does the pain happen?
      - How long does it last?
      - Does the pain come and go?
      - Have you had this pain before?
      - Does the pain keep you from doing all you want to do?
      - Does the pain disrupt your sleep?
      - Has your mood changed because of the pain?
      - Are your relationships being affected by the pain?
      - What do you think is causing the pain?
      - What makes the pain better?
      - What makes it worse?
      - The answers to these questions can help your doctor find possible causes.”
      - “You many also be asked to rate your pain on a scale of some kind/ Some scales use faces showing different expressions.”
      - “In most cases, additional tests, such as x-rays, are not needed. A thorough patient history and physical exam are usually used to guide effective pain treatment.
      - **But even if a specific physical cause cannot be found, your pain is real!** And even when the source is unknown, the pain can almost always be managed.”
      - Opioid medications
        - “Opioid myths

Myth: Opioid medications are always addictive.

Fact: Many studies show that opioids are *rarely* addictive when used properly for the management of chronic pain.

Myth: Opioids make it harder to function normally.

Fact: When used correctly for appropriate conditions, opioids may make it *easier* for people to live normally.

Myth: Opioid doses have to get bigger over time because the body gets used to them.

Fact: Unless the underlying cause of your pain gets worse (such as with cancer or arthritis), you will probably remain on the same dose or need only small increases over time.”

- - - Non-pharm therapy starts at page 21
    - Resources
      - American Academy of Pain
      - American Chronic Pain Association
      - American Geriatrics Society
      - AGS Foundation for Health in Aging
      - American Pain Foundation
      - Arthritis Foundation
      - The National Pain Foundation
    - Sponsor
      - PriCara
    - Partners
      - American Academy of Pain Medicine
      - American Geriatrics Society
      - AGS Foundation for Health in Aging

Document Notes

- **Pain Non-Branded Campaign Market Research – Round 2**
  - The Campaign = Non-Branded Message regarding the under treatment of pain, why so and what can lead to
  - The P3 Program = **Physicians Partnering Against Pain**
  - The Product = Tapentadol IR (NUCYNTA)
- **Market Research Objectives =** Test the non-branded campaign's ability to "impact" the market place behaviors/thinking relative to the treatment &. management of acute pain
  - To Explore physicians’ reactions to four messages presented by a sales representative
  - To understand the impact of the message regarding the undertreatment of acute pain and its implications
  - To understand the connection of educational campaign to the Tapentadol TPP (Triplicate prescription program???)
- **THE MESSAGES – RECAP (see screenshots)**
  - Message = Undertreatment of Pain
    - Behavioral Change = will attempt better assessment of acute pain
  - Message = Consequences
    - Behavioral Change = **More aggressive approach to treating (stronger dosing and meds);** desire to spread the message to other physicians
  - Message = Concerns that Hinder Effective Pain Mgmt
    - Behavioral change = Low abuse numbers
    - About half believe the addiction percentage (For the most part, the Rep was successful with refocusing them from addiction to side effect concerns)
  - Message = Multi-Pathways
    - NEW AGENTS – we have a new solution
    - Behavioral Change = **Increased opioid use (PCP), increase use of poly pharmacy until Product Y becomes available**
- **Impact of Campaign Overall =** PCPs state that they will be more aggressive in their treatment and use more opioids
- Physicians offer the following advice and suggestions on how to better present the educational campaign regarding acute pain management
  - Include thought leader presentations and peer-to-peer discussions in the campaign
- Reactions to Tapentadol IR TPP
  - Other benefits include efficacy similar to oxycodone, low withdrawal potential, **and some physicians on their own made a leap that it may result in lower addiction potential**
- Physicians report many patient types as undertreatment. These include: **the elderly**, **younger patients**, post-op and **post-trauma patients**
  - RECOMMENDATION = Include these patients in the undertreatment message

1. The Epidemic of Pain in America
   - [https://www.industrydocuments.ucsf.edu/docs/yqgg0230](https://www.industrydocuments.ucsf.edu/drug/docs/#id=yqgg0230)
   - Author : American Pain Foundation, The; Rogers, Mike
   - Document Date : 2006 June 13
   - Type : report
   - ID : yqgg0230 ( TID : wnf71j00 )
   - ARK : ark:/88122/yqgg0230
   - Collection : Oklahoma Opioid Litigation Documents; Opioid Documents Collection
   - Key Points:
     1. Extensive report (133 pages) supporting the concept of undertreatment of pain
     2. AUTHORS
        1. Howard Heit – paid consultant for Cephalon and J&J ([link](https://projects.propublica.org/d4d-archive/search?utf8=%E2%9C%93&term=howard+heit&state%5Bid%5D=47&company%5Bid%5D=&period%5B%5D=&services%5B%5D=))
        2. PAIN CARE FORUM + Representative Mike Rogers (R-MI)
        3. Presented at the Cannon House Office Building, Washington DC
     3. Interesting Tidbits
        1. Pain affects people at all stages of life - including infants, children, young adults, and the elderly.
        2. For one third of sufferers, their chronic pain was so severe and debilitating, they felt they couldn't function as normal people and sometimes felt so bad they wanted to die.
        3. 2/3 of older Americans who take pain medications said pain still prevents them from performing routine tasks, engaging in hobbies, or doing things they enjoy. Of these, 44% said they had difficulty Walking .
     4. Personal Stories of Pain
        1. Alexandra, sixteen years old (patient w/RSD)
           1. At this moment I am trying to start a program at the local hospital for kids facing chronic pain problems and giving them support and just a friend.
        2. Jim, US Military Veteran
           1. For years my pain was ignored. I was labeled as a drug seeker. The Veterans Hospitals would never address the real cause
           2. They [VA] didn't know how to treat what they created
           3. This disease has no cure but to take pain medication.
           4. I have had to fight the Veterans Heath Care System for years
     5. The pain gap – minorities in America are considered “under treated for their pain from medical conditions”
        1. Donald Dillard counted himself lucky to survive when a loaded dump truck rolled over his body, crushing his pelvis and thighs and shredding a football-size chunk of flesh in his torso. But the 15 years since have been a nightmare of agonizing days and nights made worse by the lack of potent pain medications at pharmacies near his home, struggles to get proper care and the red tape involved in workers' compensation coverage
        2. Hispanics and African Americans are consistently under-treated for pain across a range of conditions, from cancer to chest pain to broken bones
        3. "There are many people not getting treatment for their chronic pain, and race is part of the equation."
        4. Just as important, Staats added, is that pain management is not fully recognized as a board specialty by the American Board of Medical Specialties, "But there are a group of like-minded doctors who recognize that there is disease of chronic pain," said Staats, who serves on the board of directors of the American Academy of Pain Medicine. "Chronic pain affects a person in so many different ways — physical, psychological, emotional
     6. Professional Education – Fast Facts
        1. Most front-line family practitioners and specialists have very little training or education in identifying and treating the various distinct kinds of pain conditions.
        2. Despite the fact that pain is the number one condition that brings people to the doctor, pain education is barely present in most medical school curricula.
        3. **There are millions of people living with pain who are told by healthcare professionals that opioid medicines, one of the most powerful and effective class of medicines, are addictive and shouldn't he used.**
        4. Each year there are millions of people near death and in severe pain when there are effective pain medicines and treatments available to relieve them of their pain.
        5. **Health professionals and the public are unaware:** • Physical dependence on a medication is not the same as addiction. • Appropriate use of opioid medications (like oxycodone) is safe and effective and unlikely to cause addiction in people who are under the care of a doctor and who have no history of substance abuse. • Opioid medications are sometimes the only effective treatment for some types of pain.
        6. The typical healthcare practitioner is both time-challenged and **clueless** with regard to the treatment of pain,
        7. Americans come to their healthcare professionals expecting to be cared for, to have their pain relieved, but due to practitioner ignorance about the significance of untreated or undertreated pain, and the possibility of prosecution, they opt to leave patients in pain as the "safest course."
     7. Balancing the Need to Curb Diversion of Pain Medicines and the Need to Protect Access to Pain Medicines
        1. In most communities it is difficult for pain patients to find physicians willing to prescribe opioid medicines for pain.
        2. There is a need to reform overly restrictive regulatory polices which impede pain relief.
     8. PITCHING RELIEF – Firsthand account from Dr. Heit (paid consultant)
        1. Heit, 61, doesn't use prescription opioids for his own pain now, but he does prescribe them in high doses to many of his patients, and he's seen the drugs (in conjunction with proper monitoring) provide remarkable relief ~ the kind he still wishes he had had available back when he really needed it. As the showdown between pain doctors and prosecutors stiffened several years ago, he felt obliged to get more actively involved in defense of opioid treatment despite the potential risk to his practice
        2. Shugol, 54, wheeled herself into Heit's Arlington Boulevard office two weeks ago for a monthly appointment, smiling broadly and filled with an energy seldom seen in people who don't carry her many physical burdens. The first order of business was, as always, to hand Heit her vials of drugs, so he could see exactly how many pills she had used since the last visit. Heit took out a pill counter and went to work, first on the OxyContin, and then the Dilaudid. He was puzzled to find more than 100 extra pills. "Have you been taking everything you need?" he asked. "Yep, but I think you made a mistake last time," she replied. Rather than writing a prescription for 230 pills, Heit had written one for 330 pills, and that's what the pharmacist filled. As Shugol continued to talk of the active life she can now have because of the opioids and her care by Heit, the doctor went through the detailed paperwork he keeps on all patients.
        3. Shugol had followed Kathryn Brock of Reston — another woman in a wheelchair with an easy smile and a strong desire to remain active - into Heit's office. Brock sufferers from rheumatoid arthritis in virtually every joint in her body, and she, too, is subject to constant pain. She says that her regimen of six OxyContin and eight Dilaudid pills a day has kept her marriage going, and gives her the ability to continue painting, which she does regularly
        4. **The Gift of a Baby** A fourth patient seeing Heit earlier this month was a young woman who had fallen down a flight of stairs at a nightclub and mptured two disks in her neck. Another jock accustomed to playing with pain, she tried to gut her way through it. Doctors recommended surgery, but she resisted. Her boyfriend recommended Heit, and she began treatment. She improved markedly, married the boyfriend and, while still on OxyContin, got pregnant. She knew she could never get through the pregnancy without the medication, but she was concerned that the child could be in withdrawal at birth. When the baby was bom in 2004, Heit was at the hospital to examine the newborn for signs of opioid trouble, and was relieved to find none. Heit is the kind of person who likes to connect quickly and deeply to others, and so it wasn't entirely unexpected that he would be in the hospital. But there was another reason for his presence. The new mother, whom Heit had received permission from Virginia medical authorities to treat, was Jamie Heit ~ his son David's wife. And the healthy child, bom to a woman who wasn't sure she could ever carry a baby after her fall, was his first granddaughter, Lilly.
   - Empowerment – WOMEN
     1. Create patient demand for proper pain diagnosis and treatment; encourage patients to advocate for their rights
     2. Consider vital tactics/word of mouth education – let women educate women in their natural setting **(Tupperware parties, garden groups, e-cards)**
     3. Conduct media roundtable w/ women’s books; could work with additional partner with a female focus (like the National Women’s Health Resource Center)
     4. Consider using applicable information from the “Savvy Woman Patient” chapter on women & pain
   - CHRONIC PAIN HITS WOMEN HARDER – Jennifer Wider, Society for Women’s Health Research
   - Provide visuals of multiple patients (woman, man, child, elderly person) to help them find their fit
     1. Each visual could contain descriptions to help patients identify
   - Flyer from Research!America = **Facts about Pain – Investment in research saves lives and money**
     1. Pain Today
        1. As many as 20% of children experience chronic pain
        2. Inadequate pain management is more severe problem for ethnic and racial minorities than for other population groups
     2. The Bottom Line
        1. In a recent Research!America public opinion survey, 57% of Americans reported experiencing chronic or recurrent pain. Sixty-six percent of those in pain expect to live with it for the rest of their lives. Americans want and expect a sustained investment in research to alleviate the burdens caused by chronic pain.
   - **When it comes to severe pain, doctors still have much to learn**
     1. A Painful lesson
        1. I called the surgeon's office weekly and reported my minimal progress in pain control, but at no point was an increase in pain medication suggested, nor was I referred to a pain management specialist on the hospital staff
        2. When, at seven weeks after surgery, I spoke to Dr. Schneider, a Tucson-based specialist in pain management and addiction medicine, she chastised me for not being more insistent about getting adequate pain relief. The trouble is, when you're experiencing intense pain, it's hard to be proactive about anything
        3. Many doctors are afraid to prescribe narcotic drugs like oxycodone, fearing they will create addiction problems. But that in fact rarely happens to chronic pain patients who don't have a history of addiction. When a pain patient needs increasing doses of a narcotic, it's nearly always because the pain worsens, as often happens in patients with advanced cancer. Patients do become tolerant to side effects, like grogginess, but rarely to the pain-relieving properties of these drugs.
     2. When the Nerves Respond
        1. When I read this,! realized I was on the wrong track, taking too little of the long-acting drug and too much of the short-acting one
        2. As it turned out, my internist knew far more than my surgeon about treating pain. He has many elderiy patients with chronic pain and knows very well how to treat it. I realize now I should have sought his help from the beginning. Or I should have asked to be referred to a pain management specialist at the hospital where I had my surgery
     3. Let’s Fix What’s Broken
        1. First and foremost, patients need to be proactive and insist on the help they need. If patients are not able to do this for themselves, an advocate should do it for them. Second, every person with prolonged or chronic pain should become educated about the huge range of medications, therapies and complementary remedies available to treat pain.
   - **Painful Choices: Physicians challenged by quest to end suffering**
     1. Nursing home patients have historically been some of the most undertreated for pain. The complaints of fragile elderly are sometimes dismissed as "normal aging," It's even worse, says Community Nursing Services hospice director Dr. Anna Beck, for those who are too weak or confused to communicate
     2. When it comes to severe pain, it's called "pain management'' for a reason, because some pain is so awful that it can't entirely be erased. Marcia Solum is thrilled to now have pain she can live with, Before, when her pain was unbearable, she would sometimes take eight Advil every four hours. "I was trying to be able to have a life, and I just couldn't," she says, her voice breaking. Now on a morphine pump, she can get out of bed each day, teach sewing classes, have 25 people over for Thanksgiving dinner.
   - **PREVALENCE: Fast Facts**
     1. For American military, the current way in Iraq is resulting in a significant number of body and limb injuries which often lead to life-long chronic pain conditions


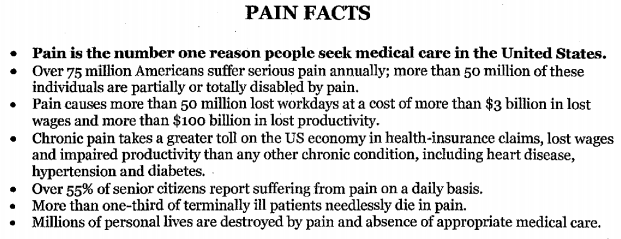


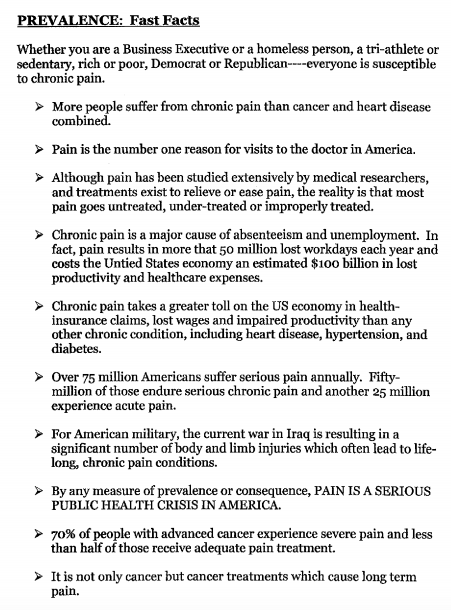


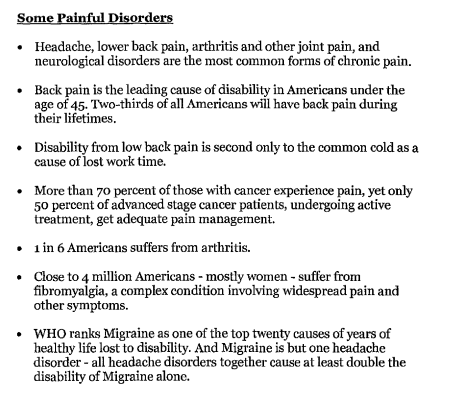


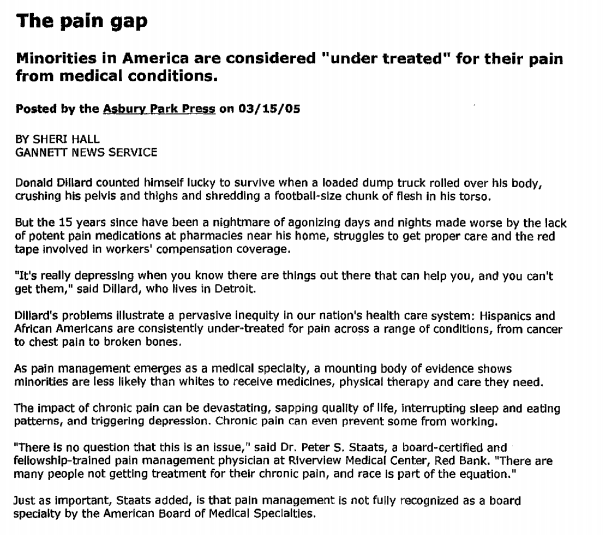


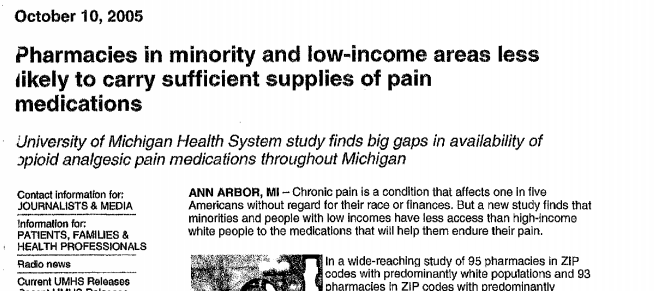


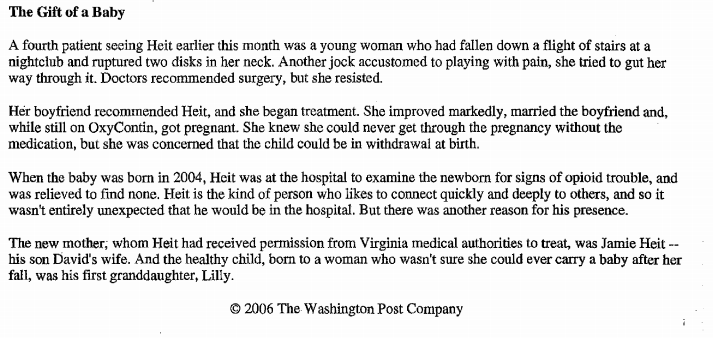


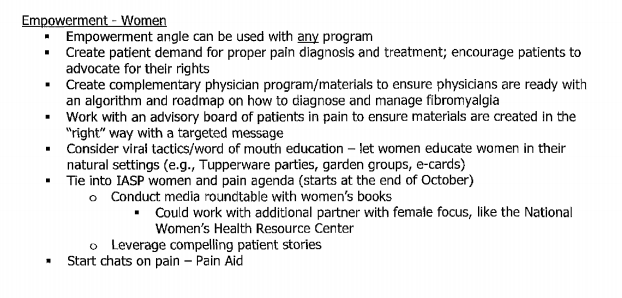


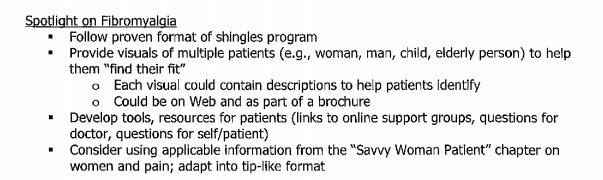


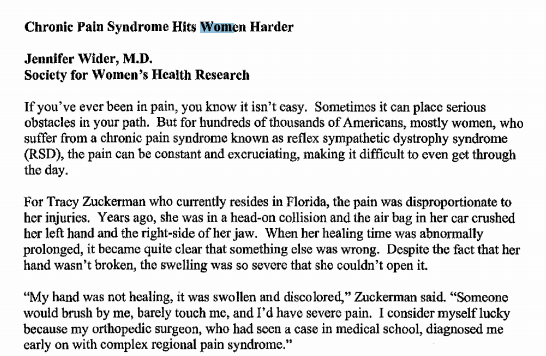


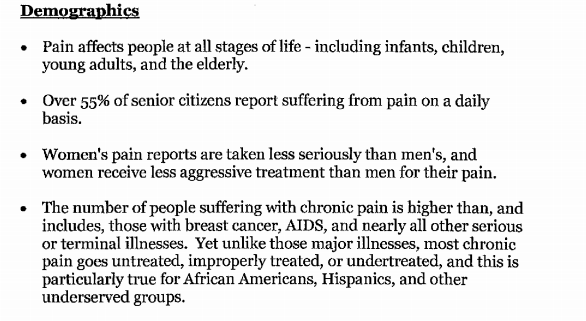


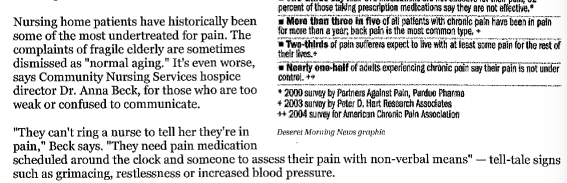


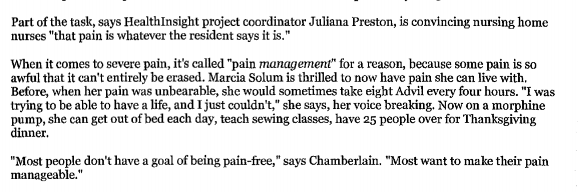


1. Pain Management for Older Adults
   - [https://www.industrydocuments.ucsf.edu/docs/hngg0230](https://www.industrydocuments.ucsf.edu/drug/docs/#id=hngg0230)
   - Author : Baker, Kathy; American Academy of Pain Medicine; Conrad and Associates, LLC
   - Document Date : Unknown
   - Type : publication
   - ID : hngg0230 ( TID : tlf71j00 )
   - ARK : ark:/88122/hngg0230
   - Collection : Oklahoma Opioid Litigation Documents; Opioid Documents Collection
   - Key Points:
     1. The American Academy of Pain Medicine, American Geriatrics Society
     2. Sponsored by PriCara (Janssen)
     3. “This brochure and DVD are aimed specifically at older adults and what they need to know to get effective pain relief. You will learn about your options for pain management and how to talk to your doctor about finding a treatment that’s right for you. By learning more about pain and the many ways it can be treated, you are taking solid steps toward reducing the pain you or a loved one may be feeling.”
     4. “Pain is the body’s alarm system.”
     5. “That’s why most doctors say that ‘pain is what the patient says it is.’ In addition, sometimes a physical cause of pain cannot be found. Some people worry that a doctor will think their pain is ‘all in their head.’ Most doctors know better. They will take your pain seriously and work hard to find a solution.”
     6. “You may be asked”
        1. Where does it hurt?
        2. Does the pain move from place to place?
        3. Do you have pain in more than one place?
        4. When does the pain happen?
        5. How long does it last?
        6. Does the pain come and go?
        7. Have you had this pain before?
        8. Does the pain keep you from doing all you want to do?
        9. Does the pain disrupt your sleep?
        10. Has your mood changed because of the pain?
        11. Are your relationships being affected by the pain?
        12. What do you think is causing the pain?
        13. What makes the pain better?
        14. What makes it worse?
        15. The answers to these questions can help your doctor find possible causes.”
        16. “You many also be asked to rate your pain on a scale of some kind/ Some scales use faces showing different expressions.”
        17. “In most cases, additional tests, such as x-rays, are not needed. A thorough patient history and physical exam are usually used to guide effective pain treatment.
        18. **But even if a specific physical cause cannot be found, your pain is real!** And even when the source is unknown, the pain can almost always be managed.”
        19. Opioid medications
            1. “Opioid myths

Myth: Opioid medications are always addictive.

Fact: Many studies show that opioids are *rarely* addictive when used properly for the management of chronic pain.

Myth: Opioids make it harder to function normally.

Fact: When used correctly for appropriate conditions, opioids may make it *easier* for people to live normally.

Myth: Opioid doses have to get bigger over time because the body gets used to them.

Fact: Unless the underlying cause of your pain gets worse (such as with cancer or arthritis), you will probably remain on the same dose or need only small increases over time.”

- - 1. Non-pharm therapy starts at page 21
    2. Resources
       1. American Academy of Pain
       2. American Chronic Pain Association
       3. American Geriatrics Society
       4. AGS Foundation for Health in Aging
       5. American Pain Foundation
       6. Arthritis Foundation
       7. The National Pain Foundation
    3. Sponsor
       1. PriCara
    4. Partners
       1. American Academy of Pain Medicine
       2. American Geriatrics Society
       3. AGS Foundation for Health in Aging

1. Pharmaceutical Industry-Sponsored Meals and Physician Prescribing Patterns for Medicare Beneficiaries
   - [https://www.industrydocuments.ucsf.edu/docs/xpgg0230](https://www.industrydocuments.ucsf.edu/drug/docs/#id=xpgg0230)
   - Author : JAMA Internal Medicine
   - Document Date : Unknown
   - Type : article; publication
   - ID : xpgg0230 ( TID : anf71j00 )
   - ARK : ark:/88122/xpgg0230
   - Collection : Oklahoma Opioid Litigation Documents; Opioid Documents Collection
   - Key Points:
     1. “OBJECTIVE To study the association between physicians’ receipt of industry-sponsored meals, which account for roughly 80% of the total number of industry payments, and rates of prescribing the promoted drug to Medicare beneficiaries.”
     2. “DESIGN, SETTING, AND PARTICIPANTS Cross-sectional analysis of industry payment data from the federal Open Payments Program for August1 through December 31, 2013, and prescribing data for individual physicians from Medicare Part D for all of 2013.”
     3. “CONCLUSIONS AND RELEVANCE Receipt of industry-sponsored meals was associated with an increased rate of prescribing the brand-name medication that was being promoted. The findings represent an association, not a cause-and-effect relationship.”

204) Non-Branded Promotion

Document Data

- Author : Leonard, Lynn
- Document Date : 2007 June 29
- Type : diagram; email; report
- ID : fngg0230 ( TID : rlf71j00 )
- ARK : ark:/88122/fngg0230
- Collection : Oklahoma Opioid Litigation Documents; Opioid Documents Collection

Document Notes

- Email from Lynn Leonard
  - here are some slides on the unbranded efforts for the tactical presentation
  - I have included the "banana" analysis on one of the slides - which gets us to 60% of physician targets and gives us 60% of the business (targeted business).
- Preliminary Message Points
  - 1) Pain is mismanaged and under treated
  - 2) The consequences are serious and significant
  - 3) Appropriate treatment requires effective pain control
- Capturing and Maintaing Interest
  - Supported through lively stories – Illustrate patient/MD gap and highlight elderly patients

Slide Title: **PriCara Non-Branded Promotion**

- Disrupting a Complacent Marketplace
  - Web Strategy: Professional Promotional Platform + Physician-Patient Education/PR Strategy
- Disrupt the Marketplace
  - Heighten awareness of the under-treatment of pain and its consequences
  - Establish reps as pain experts
  - Profile targeted HCPs in preparation of launch
- Unbranded Message Continuum – Paving the way for tapentadol
  - 1) Pain is mismanaged and under-treated
  - 2) There are serious consequences of mismanaging & under-treating pain
  - 3) Appropriate treatment requires EFFECTIVE pain relief
  - 4) New pathways in pain management coming soon
- Pain Management: **At the Threshold of NEO Thinking**
- Gaining Endorsements for Credibility
  - Establish Instant Credibility
  - Develop Good Will
  - Alignment of Long Term Goals
  - Alleviate Regulatory Anxiety


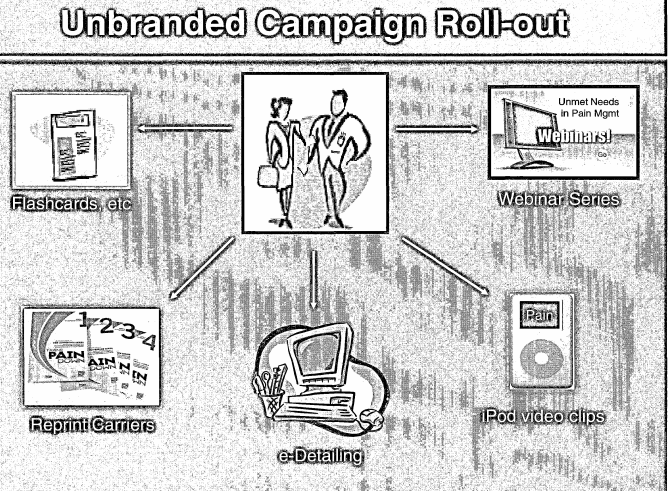


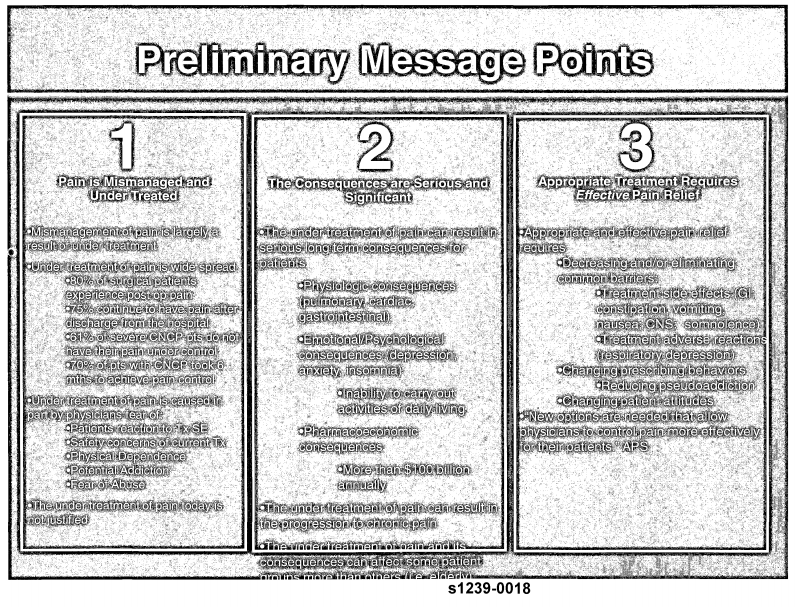


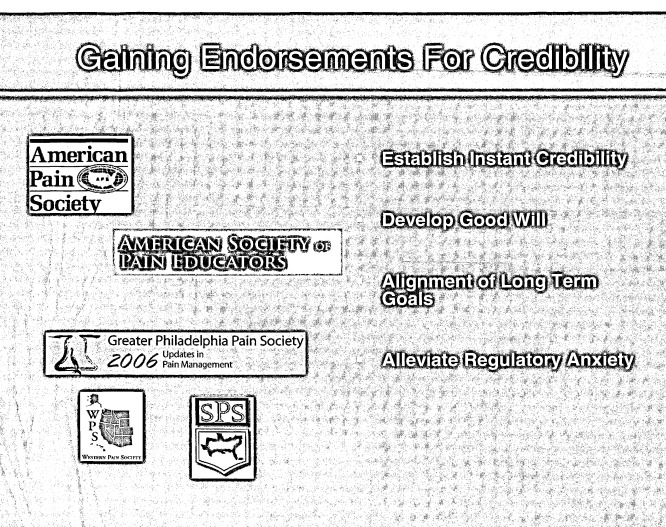


225) Duragesic Historic Sales Aids

Document Data

- Author : Deguchi, Kay
- Document Date : 2006 January 09
- Type : email
- ID : qqgg0230 ( TID : ynf71j00 )
- ARK : ark:/88122/qqgg0230
- Collection : Oklahoma Opioid Litigation Documents; Opioid Documents Collection

Document Notes

- Email from Dan Keenan (KPR Advertising) = attached are PDF files we were able to locate for the selected historical Duragesic campaigns.
- Response from Lynn Leonard (Duragesic product manager at Janssen)
  - Since we are launching non-cancer - I'm thinking the life interrupted campaign might be a good one,
  - As you know we will have a very tight budget to **launch CNCP (chronic non-cancer pain) in Australia**. I am reaching out for your assistance with the historical Durogesic sales aids to ensure that we tap into the success that Durogesic enjoyed in the US and to minimize our costs by not reinventing the wheel.
  - PS – Durogesic 12 will be launched in Australia Feb 1, 2006
- Response from Mark Porter (KPR Promotional Services)
- **PDF SLIDES WERE NOT ATTACHED TO THIS DOCUMENT 🙁**

226, 227) Duragesic Sales Force Update

Document Data

- Author: Janssen
- Document Data: 2000
- Type: report, marketing; newsletter
- ID: gngg0230, hrgg0230 (Duplicate of gngg0230)

Document Notes

- **Found via “More like this” option from #225; I don’t know if this is the “PDF” referred to in #225**
- Congratulations to the 275 Sales Force! With your leadership, DURAGESIC attained numerous **all-time highs in prescription and dollar volume in 2000**. Due to your efforts, for the first time, DURAGESTC attained $400 million in 2000. The strategic focus, sales direction, sales materials and programs for 1st cycle 2001 are designed to enhance your ability to appropriately position DURAGESIC as the 1st choice, long-acting opioid for chronic pain. Thanks to you, 2001 promises to be an exciting time for DURAGESIC -we will surpass Half-a-Billion Dollars in Sales!
- Market Update
  - Chronic pain market grows rapidly and significant growth opportunity for DURAGESIC
  - In 2000, the market grew nearly 50% versus 1999. The majority of this growth is a result of the dramatic growth in the chronic non-malignant pain market.
- Strategic Focus
  - **High Deciled Physicians**
    - The high deciled physicians continue to represent significant opportunity for DURAGESIC due to their high volume prescribing of chronic pain medications
    - greater emphasis being placed on the top 1000 who account for 20% of all the dollars in the pain market
  - **Life, Uninterrupted**
    - In 2000, we conducted extensive market research to assess the effectiveness of our new promotional campaign. Physicians overwhelmingly stated that the "Life, Uninterrupted" message was credible and compelling enough to cause them to prescribe DURAGESIC as a 1st choice for chronic pain
  - **Expand DURAGESIC Use in Non-Malignant Pain**
    - Physicians are becoming more comfortable using opioids in non-malignant pain. Our objective is to convince them that DURAGESIC is effective and safe to use in areas such as chronic back pain, degenerative joint disease, and osteoarthritis
    - It is important to remind physicians that the APS, AAPM, and AGS have all endorsed the appropriate use of opioids to manage chronic, non-malignant pain.

254) Duragesic Focused and Targeted Execution – 2004 Business Plan

Document Data

- Author : Janssen Pharmaceutica
- Document Date : 2003 August 06
- Type : presentation; slides
- ID : fxgg0230 ( TID : pif71j00 )
- ARK : ark:/88122/fxgg0230
- Collection : Oklahoma Opioid Litigation Documents; Opioid Documents Collection

Document Notes

- Title of Slides = **DURAGESIC; FENTANYL TRANSDERMAL SYSTEM; FOCUSED & TARGETED EXECUTION** (2004 business plan)
- Market Analysis – Growth Drivers
  - Recognized undertreatment of pain
    - Increased legislation
    - **Mandatory CE/CME**
    - Litigation for undertreatment
  - Acceptance of Opioids for non-cancer pain
  - **Consumers more demanding**
  - New & future competitive entries
- Market Analysis – Growth Inhibitors
  - **Perceived “risk” of opioids -- OPIOPHOBIA**
  - **Limited evidence-based scientific data**
  - Increased State/MCO restrictions
- Duragesic statement = **Life, Uninterrupted** and  **Work, Uninterrupted**
  - Duragesic positioning statement – Duragesic significantly improves physical and social functioning by providing the only chronic pain relief that is consistent and effective for 72 hours
- Non-cancer pain is the growth opportunity
- Differentiate DURAGESIC from competition
  - Focus on physician and patient end goal
  - Continue to leverage KOL relationships: Med affairs, NPEC, National Pain Summit
  - Enhance KOL, Pharmacist & key customer relationships within market
- Internal Assessment
  - Lessons Learned: functionality positioning aligns with physician and patients’ end goal of therapy 🡪 Brand messages have evolved to a more patient centric platform
- Internal Assessment: SWOT Analysis & Issue Identification
  - Weakness – Limited clinical data
  - Opportunities – Functionality message ownership
  - Threats – Opioid abuse publicity/ media & potential awareness
- **See “Medical Affairs; 2004 Key Projects” screenshots – potential evidence of seeding trials, ghost writing, general academic malfeasance, CME planning**


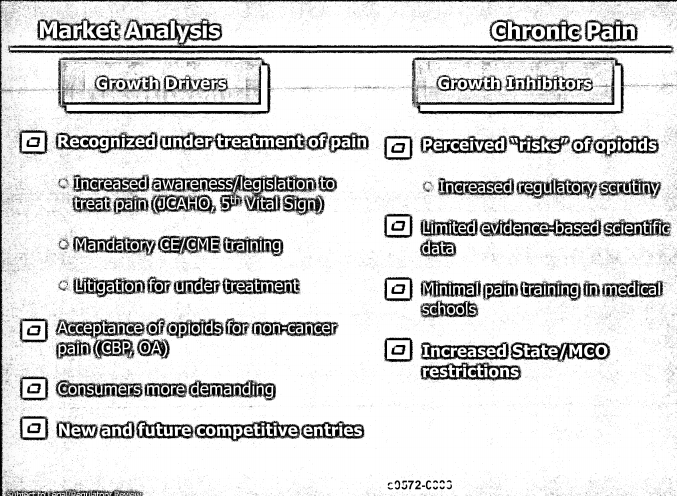


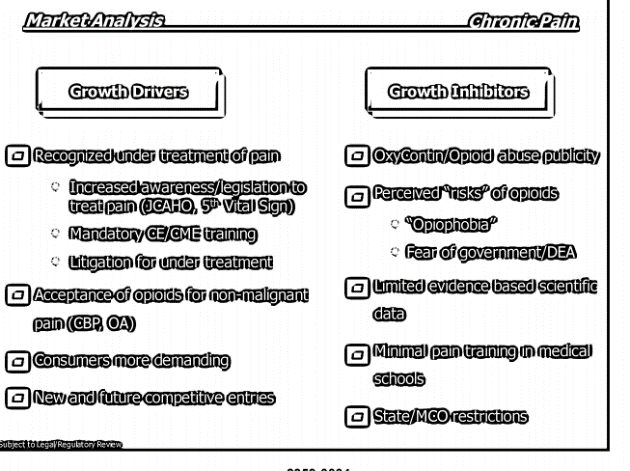


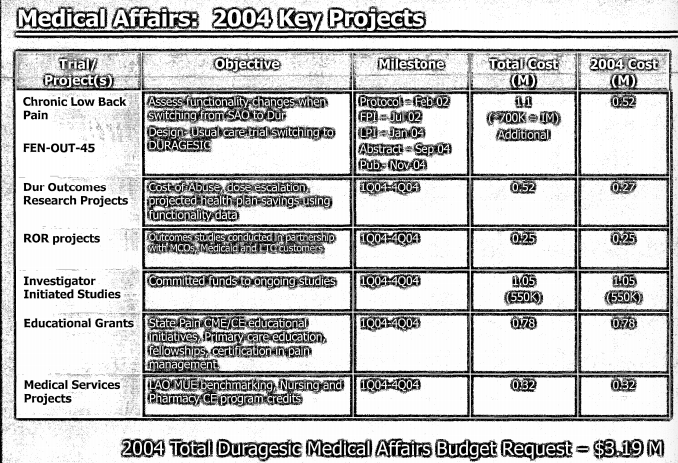


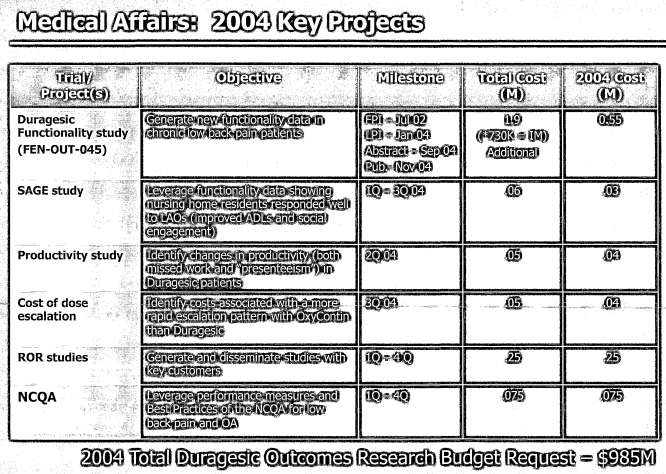


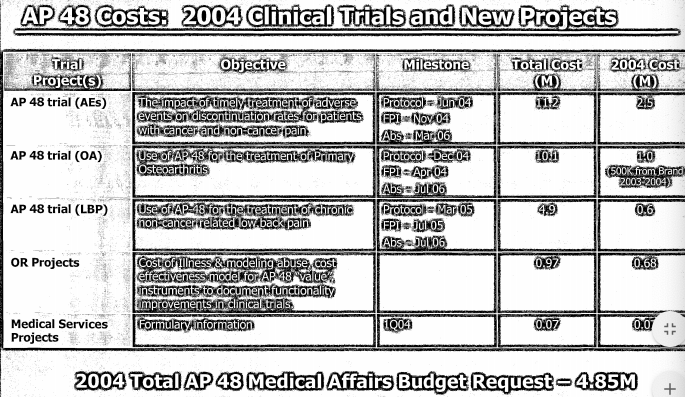


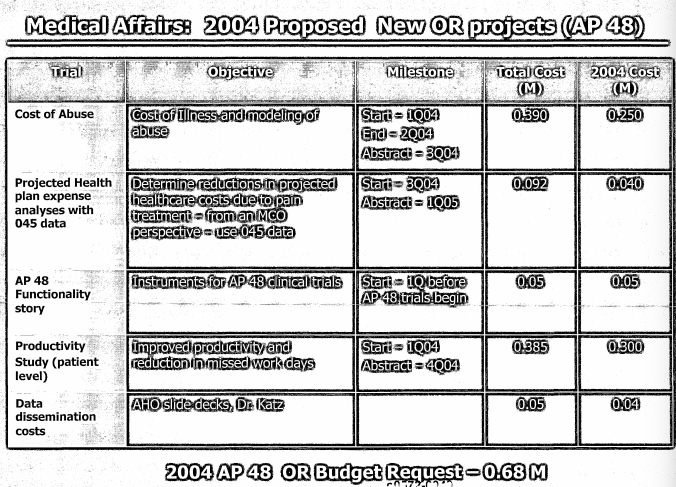


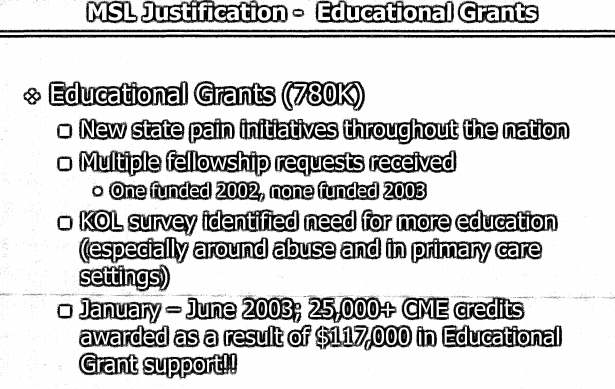


271) Duragesic Sales Force Update

Document Data

- Author : Unknown
- Document Date : 2002
- Type : report; report, marketing
- ID : qhgg0230 ( TID : kif71j00 )
- ARK : ark:/88122/qhgg0230
- Collection : Oklahoma Opioid Litigation Documents; Opioid Documents Collection

Document Notes

- Coming off a record-breaking year of $543 million in 2001, the bar has been raised for DURAGESIC® in 2002 to $692 million in sales, a 28% increase!
  - You are our primary sales force that drives nearly 75% of the business through **Pain Specialist and Primary Care physicians**
- Your commitment to sell DURAGESIC by being **ethically aggressive** will continue to position the brand for double digit sales growth throughout 2002 and beyond.
- Patient Target: **Expand DURAGESIC use in non-malignant pain**
  - Chronic, non-malignant pain states, such as lower back pain, represent considerable growth opportunities for the brand; it is imperative that we accelerate our growth in these areas.
  - Our objective is to convince physicians that DURAGESIC is effective and safe to use in moderate to severe chronic pain such as **back pain and degenerative joint disease** like osteoarthritis.
- Core Message: Life, Uninterrupted
  - Research identified the opportunity to place even greater emphasis on patient functionality as the primary goal of treatment, supported by a strong message on pain relief efficacy. Specifically, research shows: 1) **functionality is a key driver of brand selection**, 2) functionality is the end-benefit of physician treatment goals and 3) no brand currently owns functionality.
- Sales Material
  - Milligan Reprint Carrier
    - Reprint carrier for chronic non-malignant pain discussing long-term observation of DURAGESIC patients. Study supports that long-term treatment of chronic pain is effective and well tolerated. A significant improvement was shown in SF36 functionality assessment scores and patients' demonstrated preference for DURAGESIC over previous opioid medication.
  - National Pain Education Council NPEC Invitation
    - National Pain Education Council is funded by an educational grant from Janssen. Invitation to participate in a multimedia CME program for physicians and other medical professionals on the appropriate opioid pharmacotherapy for chronic pain management
  - **Dr Passik Substance Abuse Issue Teletopics**
    - CME resource on the subject of Substance Abuse Issues in Chronic Pain presented by Dr. Steven Passik. This interactive teleconference workshop identifies aberrant behaviors that may be indicative of substance abuse problems and issues with opioids in the treatment of chronic pain and impact of drug choice and use on minimizing potential abuse problems
    - CONTROL+F “PASSIK” -- lots of money from pharma!!

282) FYI Business Plan

Document Data

- Author : Roman, Ramiro
- Document Date : 2001 January 08
- Type : email; graph
- ID : tqgg0230 ( TID : bof71j00 )
- ARK : ark:/88122/tqgg0230
- Collection : Oklahoma Opioid Litigation Documents; Opioid Documents Collection

Document Notes

- Email Title = **Duragesic Business Plan; Janssen 2001 Pain Franchise Plan**
- Patient Potentials
  - Potential to earn revenue per patient – Bigger bubble = more days of treatment – **Postoperative pain, Arthritis, Low Back Pain**
  - Big groups (Arthritis and Post-Op pain) = driven by geriatric population growth and increase in day surgeries
- Pain Franchise SWOT Analysis
  - Weakness = **Limited Clinical data**
  - Opportunities
    - Large sustainable market growth
      - Recognition of undertreatment of chronic pain
      - Growing acceptance of opioid usage for non-cancer pain
    - Significant market opportunities: Chronic back pain, Arthritis
  - Threat
    - **Move toward evidence-based prescribing**
    - Risk of negative “incidents” higher in non-cancer indications


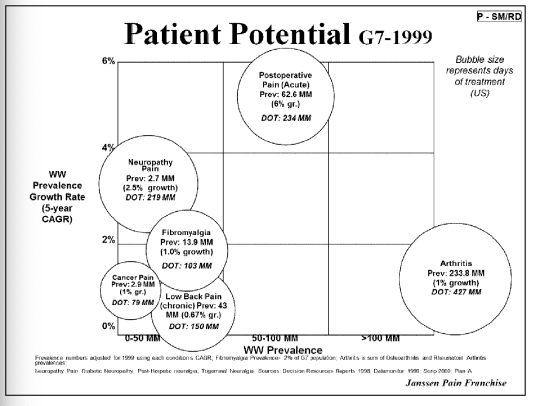


289) 2001 Business Plan August 2000

Document Data

- Author : Unknown
- Document Date : 2000
- Type : agenda; chart; diagram; report, scientific; table
- ID : sqgg0230 ( TID : aof71j00 )
- ARK : ark:/88122/sqgg0230
- Collection : Oklahoma Opioid Litigation Documents; Opioid Documents Collection

Document Notes

- Title = **DURAGESIC; 2001 Business Plan August 2000**
- Duragesic Quadrant Analysis
  - Higher scores on more important attributes
    - Efficacy, Dosing, QOL
  - Lower scores on less important attributes
    - Safety, Side effects, regulatory issues/concerns
  - This is an ideal pattern of attribute scores
- Duragesic SWOT Analysis
  - Weakness = limited clinical data
  - Threat = State medical boards, government intervention
- Expand DURAGESIC Use in non-malignant pain
  - Tactic = **MEDICAL EDUCATION** -- direct mail, dinner meetings, spin-offs, Symposia (APS, AAPM)
- Medical Development Clinical Trials
  - ACTIVE TRIALS
    - **FEN-71** = DURAGESIC vs OXYCONTIN in chronic non-malignant pain (primary endpoint = patient satisfactions; result = 1Q01)
    - **FEN-72** = DURAGESIC vs PERCOCENT in chronic low back pain (primary endpoint = patient satisfaction; result =2Q10)
  - UPCOMING TRIALS
    - **Osteoarthritis**
      - Objective = position DURAGESIC as first line opioid for chronic pain
      - Timing – initiate 2Q01, complete – 4Q02
    - **Low back pain vs OxyContin**
      - Objective = position DURAGESIC as preferred LA opioid for chronic low back pain
      - Timing – 1Q01; Complete – 2Q02
    - **25 vs 12.5 ug/hr patch, non-malignant pain**
      - Objective = establish 12.5 ug/hr patch as first line long-acting opioid
    - **Duragesic Effectiveness Trial**
      - Primary objective = Obtain a DDMAC (division of drug marketing, advertising, communication) claim for DURAGESIC demonstrating better satisfaction over OxyContin
      - Secondary objective = Pain interference on QOL
      - **Company support = $800,000** (see slide 40)
  - **Maximize lifecycle opportunities -- ELDERCARE**
- Medical Services – Expand Duragesic use in chronic non-malignant pain
  - Chronic pain guideline presentations
  - **Chronic pain CME – 2Q01, $10,000**

292) Myths about Opioids

Document Data

- Author : Purdue; Partners Against Pain
- Document Date : 1999
- Type : report; publication
- ID : ypgg0230 ( TID : gnf71j00 )
- ARK : ark:/88122/ypgg0230
- Collection : Oklahoma Opioid Litigation Documents; Opioid Documents Collection

Document Notes

- Booklet Title = **Dispelling the Myths about Opioids**
  - **This booklet describes the myths about opioids in an effort to dispel them and open the way for better opioid therapy**
  - Author = Partners Against Pain [Unbranded??]
    - *An educational service of The Purdue Frederick Company and Purdue Pharma*
- Key Terms
  - Pseudotolerance -- Pseudotolerance is the need to increase dosage that is not due to tolerance, but due to other factors such as: disease progression, new disease, increased physical activity, lack of compliance, change in medication, drug interaction, addiction, and deviant behavior. When a once-fixed opioid dose is no longer effective, the above conditions should be reviewed to exclude pseudotolerance.
  - Pseudoaddiction -- Pseudoaddiction is drug-seeking behavior that seems similar to addiction, but is due to unrelieved pain. This behavior stops once that pain is relieved, often through an increase in opioid dose. "Misunderstanding of this phenomenon may lead the clinician to inappropriately stigmatize the patient with the label 'addict.1 In the setting of unrelieved pain, the request for increases in drug dose requires careful assessment, renewed efforts to manage pain and avoidance of stigmatizing labels."
  - Note: Tolerance to or physical dependence on opioids, which may develop in a patient on extended opioid therapy, should not be confused with addiction (
- MYTHS ABOUT OPIOIDS
  - 1 -- Opioids depress respiratory function and are therefore too dangerous to be dosed safely.
    - FACT - While respiratory depression is the chief hazard of all opioid agonist preparations, clinically significant respiratory depression rarely occurs in patients in pain for whom doses of opioids are appropriately prescribed, administered, and titrated to effect.-
  - 2 -- Opioid addiction (psychological dependence) is an important clinical problem in patients with moderate to severe pain treated with opioids.
    - FACT -- Fears about psychological dependence are **exaggerated** when treating appropriate pain patients with opioids.
    - In a review of the records of 11,882 hospitalized patients treated with opioids, there were only four cases of addiction in patients with no addiction history. [**see #22 – letter to the editor; authors express regret]**
  - 3 -- Opioid tolerance develops rapidly, necessitating progressively higher doses.
  - 4 -- Adequate opioid doses cause unmanageable constipation.
  - 5 -- The majority of patients on opioids require prophylactic antiemesis.
  - 6 -- Unacceptable sedation and confusion are a frequent side effect in opioid patients.
  - 7 -- Short-acting opioids (3 to 6 hours) are the ideal analgesic for controlling moderate to severe pain.
  - 8 -- Controlled-release opioids should only be used for the management of cancer-related pain.
    - FACT -- Opioids can be used effectively in many types of non-cancer pain, such as back pain, osteoarthritis, post-operative pain, neuropathic pain, fracture/trauma, and reflex sympathetic dystrophy (RSD)
    - Either controlled-release oxycodone (**OxyContin® CII Tablets**) or controlled-release morphine sulfate (**MS Contin® CII Tablets**) have been used successfully to provide pain relief in patients with low back pain,23 osteoarthritis,23 post-herpetic neuralgia,23 chronic musculoskeletal pain," and AIDS pain
  - 9 -- Initiation of dosage and precise titration are difficult to achieve with controlled-release opioids.
  - 10 -- Severe cancer pain commonly calls far parenteral opioids.
  - 11 -- Plasma opioid concentrations correlate directly with the level of analgesia.
- **WAYBACK MACHINE – Partnersagainstpain.com**
  - Targeting elderly, dementia, hospice patients


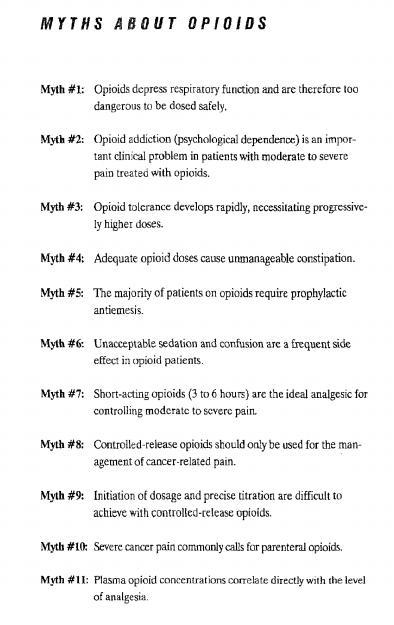


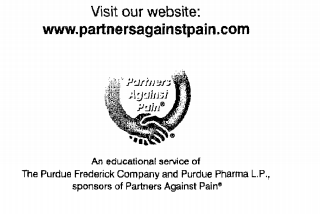


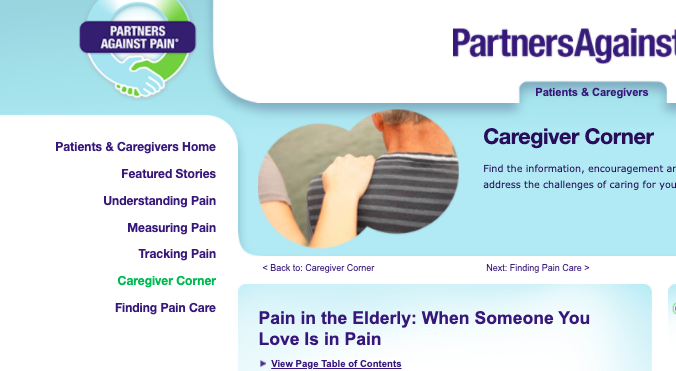


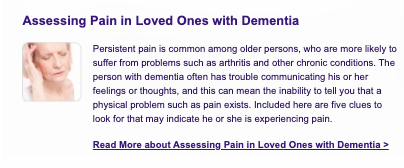


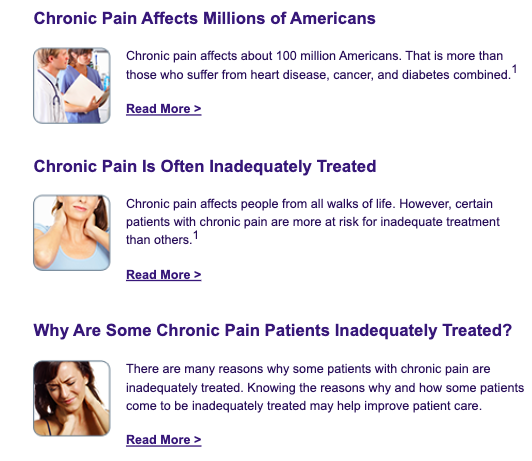


**OTHER UNBRANDED INITIATIVES**

54) Marketing Contract Review

Document Data

- **Author :** Hassler, John
- **Document Date :** 2015 June 18
- **Type :** form; letter
- **ID :** yjgg0230 ( TID : ojf71j00 )
- **ARK :** ark:/88122/yjgg0230
- **Collection :** Oklahoma Opioid Litigation Documents; Opioid Documents Collection

Document Notes

- **Bob Twillman, PhD** = Spokesperson for Pain Matters
  - Twillman = pain management psychologist
  - Pain Matters = pain management tool set up by the American Chronic Pain Association; [website](https://www.theacpa.org/acpa_maze/pain-matters/) for Pain Matters is currently blank
  - At the time of this document (June 2015), Bob Twillman was part of the American Academy of Pain Management
- Background on Pain Matters: <https://www.fiercepharma.com/marketing/updated-teva-goes-for-absurd-latest-opioid-addiction-video-series>
- “On behalf of Cephalon, Inc. d/bia Teva Pharmaceuticals ('Teva" or "Teva Pharmaceuticals"), I am pleased to confirm that you will be acting as our Spokesperson. As a Spokesperson, you will be asked to speak about your experience with chronic pain and the "Pain Matters" program at a live, Teva-sponsored event ("Spokesperson activities").”
- you agree to attend a meeting via telephone with Teva and its **public relations (PR) agency** to review the Guidelines accompanying this Agreement and to discuss, in general, **interactions with the public including the media**.
- You agree to participate in one or more of the following activities upon the request of Teva:
  - Provide quotes for use in press release announcing the Pain Matters website and key survery findings
  - Media interviews via telephone with national and trade reporters
  - Provide an article for “Pain Perspectives” to be published on PainMatters.com
- Nothing herein shall be construed to require you to purchase, order, recommend, or arrange for the purchase, order, or recommendation of any products manufactured and/or marketed by Teva. You acknowledge and agree that you will continue to make all decisions regarding treatment, prescribing, administration, or dispensing (including prescribing, administering or dispensing Teva products) solely in accordance **with your independent judgment** (including medical and clinical judgment, if applicable), and that such decisions shall not be affected by this Agreement or the relationship created hereby.
- ADVERSE EVENT REPORTING: In accordance with Teva policies and procedures, you agree that if, in connection with the Spokesperson activities, you receive information relating to adverse events (AE), product complaints (PC), and/or other Teva product-related safety information (e.g. special safety topics as communicated to Spokesperson through separate correspondence), **you will promptly notify Teva by email (**[**drag.safetv@,tevapharrn.com**](mailto:drag.safetv@,tevapharrn.com)**) within one business day**.
- You agree not to use the name of Teva and/or the Teva logo, or any Teva information that you obtain or receive while acting as a Spokesperson for Teva without the prior written permission of Teva.
- GUIDELINES
  - In every activity, you must disclose your relationship to the company. For example: "I am working with Teva Pharmaceuticals to raise awareness of the Pain Matters program.
  - **Use Teva approved talking points**
  - Describe the disease/disorder in an accurate, fair and balanced, and objective
- KEY MESSAGES FROM TEVA
  - **Pain affects more Americans than diabetes, heart disease and cancer combined.** While prescription pain medications are an important component of pain management, the reality is these products are prone to abuse and misuse.
  - A new survey conducted on behalf of Teva Pharmaceuticals in collaboration with the U.S. Pain Foundation and the American Academy of Pain Management found that healthcare professionals and people affected by pain recognize their personal responsibility in helping address prescription drug abuse, but acknowledge conversations about the issue can be uncomfortable. Nearly everyone surveyed agreed information and resources are critical.
  - **PainMatters.com**  **[NOTE: this website no longer exists]** is a new resource designed to help healthcare professionals and people affected by pain navigate the complex pain care landscape.
- Dollar amount listed as N/A
  - **NOTE: While it looks like Twillman himself does not get money directly from Purdue/TEVA, there is evidence that his group (American Academy of Pain Management, of which he is executive director) has been funded by Purdue/Teva (**<https://www.modernhealthcare.com/article/20160526/NEWS/160529930/state-medicaid-agencies-limiting-opioid-prescriptions>)
  - See document ID zrgg0230: **Teva Payments to Advocacy Groups**

81) 2013 National Advocacy Business Planning

Document Data

- Author : Johnson and Johnson; Janssen Pharmaceutical
- Document Date : 2012 June 29
- Type : proposal
- ID : zggg0230 ( TID : yhf71j00 )
- ARK : ark:/88122/zggg0230

Document Notes

- Business planning from Janssen (PAIN FRANCHISE) June 29, 2012
- Key Questions = How to leverage sales of Nucynta??
- 2013 PAIN Advocacy Strategy
  - Engage partners to embrace the IOM report-national/state implications advocate for and act collectively to actualize the recommendations
  - **Influence agencies that impact policy and quality to maintain or improve access**
- Barriers to care
  - FOCUS: **Engage with advocacy partners** at the national level for greater impact and alignment. Support models for collaboration and synergies that can then be applied at a regional level
  - Chronic pain can and should be thought of as a disease in and of itself.
  - Public health and community-based approaches are required to **address the under-treatment of chronic pain**
- Advocacy/Policy Focus
  - Provide state and federal legislators and other regulatory groups with access to objective materials to assist them in making public policy.
  - Collaborate with the **Pain Care Forum (PCF)** on policy issues and common strategies with key decision makers; such as HHS, Surgeon General's Office, CDC, state and federal legislators and regulators. •
  - Collaborate with State Pain Policy Action Coalition (SPPAC), a newly formed organization made up of pain focused organizations that will pro-actively inform and influence state policy . •
  - **Sponsor Public Awareness campaigns targeted at preventing chronic pain and misuse of prescription pain medications. •**
  - **Sponsor disease awareness to promote balanced and effective pain management. •**
  - Support collaboration between Medicine and Law Enforcement to prevent the "chilling effect".
    - [Chilling effect](https://oxfordmedicine.com/view/10.1093/med/9780190659721.001.0001/med-9780190659721-chapter-10) ([link](https://oxfordmedicine.com/view/10.1093/med/9780190659721.001.0001/med-9780190659721-chapter-10)) = An overzealous Drug Enforcement Administration is sometimes prosecuting the wrong physicians, thus creating a chilling effect in the medical community with regard to opioid prescription and making it harder for people in pain to get the help they need
  - Support effective Prescription Monitoring Programs (PMPs) that provide prescribing healthcare professionals with "real time access" and improve patient care.

104) Pain Brief Advocacy & Policy Monthly 2011

Document Data

- **Author :** Kohn, Robyn
- **Document Date :** 2011 July 07
- **Type :** email; report
- **ID :** tygg0230 ( TID : vmf71j00 )
- **ARK :** ark:/88122/tygg0230
- **Collection :** Oklahoma Opioid Litigation Documents; Opioid Documents Collection

Document Notes

- SEE # 103 – this is an example of the Policy Monthly Briefing proposed by Kohn
- Primary External Partners
  - GO TO PARTNER = **American Pain Foundation**
  - American Academy of Pain Medicine
  - American Academy of Pain Management
  - American Chronic Pain Association
- Secondary External Partners
  - American Academy of Hospice & Palliative Care Medicine
  - American Academy of Family Physicians
  - American Geriatric Society
  - American Society of Consultant Pharmacists
  - American Academy of Physician Assistants
  - American Academy of Nurse Practitioners
- **KEY ADVOCACY THEMES**
  - Marketplace
    - **Significant unmet needs in moderate-severe chronic pain market**
    - Advocacy on the local market level gaining momentum among partner org
  - Pain Tools
    - **Prescribe Responsibly**-external HCP community
    - **Smart Moves, Smart Choices**-Teen RX Abuse
    - **Let's Talk Pain**-Provider/Patient Communications
- **NEW INITIATIVES**
  - **Imagine the Possibilities – Pain Coalition**
    - Internal cross-functional members of the pain teams and external members of the pain communities
    - Goal: align and address issues in pain management with emphasis on abuse and diversion
  - **New Partnership: State Pain Policy Action Coalition (SPPAC)-AAPM, APF, ASPMN**
    - Goal: Formed out of need to respond and influence positive state-based public policies is crucial to assuring access to effective care for persons experiencing pain.
    - **National pain management organizations have attempted to influence** many of these issues, but often have done so in an ineffective piecemeal and hit-or-miss fashion, failing to take advantage of opportunities to **speak with one voice and achieve desired outcomes.** SPPAC provides the opportunity to create a synergistic voice to insure the ability of our members to provide and receive optimal care for pain.
    - While three organizations are initially organizing the coalition, other organizations will be invited to join once the structure is developed.
- IOM REPORT (6/29/11): **Reliving Pain in America**
  - What is the IOM report: The report, released on June 30, 2011 — Relieving Pain in America: A Blueprint for Transforming Prevention, Care, Education, and Research — calls for a cultural transformation of attitudes toward pain and its prevention and management.
  - Noteworthy findings in IOM
    - Chronic pain affects an estimated **116 million** American adults
    - Pain costs the nation up to **$635 billion each year** in medical treatments and lost productivity.
    - Chronic pain negatively affects socioeconomic status.
    - Federal and state drug abuse prevention laws, regulations, and enforcement practices have been considered impediments to effective pain management...." Among other barriers, they say "**Twentynine percent of primary care physicians and 16 percent of pain specialists report they prescribe opioids less often than they think appropriate because of concerns about regulatory repercussions."**
  - Partner Commentary
    - **Pain Care Task Force** is being formed to develop a communication and outreach plan for dissemination of key messaging across national/local markets. The activities can serve as an advocacy tool for our partner organizations.
    - The committee calls for government agencies, healthcare providers, and public and private funders of health care to adopt a comprehensive, strategic approach to reduce or eliminate the barriers to pain care.
- Cephalon – Dept 631: (FEBT) – External Project Costs
  - [https://www.industrydocuments.ucsf.edu/docs/mggg0230](https://www.industrydocuments.ucsf.edu/drug/docs/#id=mggg0230)
  - Author : Cephalon, Inc
  - Document Date : 2007
  - Type : table; report, financial
  - ID : mggg0230 ( TID : qhf71j00 )
  - ARK : ark:/88122/mggg0230
  - Collection : Oklahoma Opioid Litigation Documents; Opioid Documents Collection
  - Key Points:
    - External project costs table for FEBT (fentanyl effervescent buccal tablet)
    - Portenoy named several times throughout
- Commercialization Team Update
  - [https://www.industrydocuments.ucsf.edu/docs/zkgg0230](https://www.industrydocuments.ucsf.edu/drug/docs/#id=zkgg0230)
  - Author : Unknown
  - Document Date : 2006 October 25
  - Type : agenda; notes; table
  - ID : zkgg0230 ( TID : kkf71j00 )
  - ARK : ark:/88122/zkgg0230
  - Collection : Oklahoma Opioid Litigation Documents; Opioid Documents Collection
  - Key Points:
    - Strategic and Functional Area Updates for Fentora (fentanyl buccal tablet)
      - Regulatory Update
      - Clinical (Research) Update
      - Publication Update
      - Market Research (primary, secondary, dashboard metrics)
      - Marketing
      - PSMI
      - PR
      - Med-Ed (CME)
      - Product Availability
      - MCO
      - Sales Force
      - KOL
- Mapping Overview
  - [https://www.industrydocuments.ucsf.edu/docs/qzgg0230](https://www.industrydocuments.ucsf.edu/drug/docs/#id=qzgg0230)
  - Author : Cephalon; COGENIX
  - Document Date : 2004 July 07
  - Type : agenda; chart; table
  - ID : qzgg0230 ( TID : kqf71j00 )
  - ARK : ark:/88122/qzgg0230
  - Collection : Oklahoma Opioid Litigation Documents; Opioid Documents Collection
  - Key Points:
    - KOL development plan (including Lynn Webster)
- Tapentadol – NEO -Pathways, Heading in new directions in pain management
  - [https://www.industrydocuments.ucsf.edu/docs/kggg0230](https://www.industrydocuments.ucsf.edu/drug/docs/#id=kggg0230)
  - Author : Unknown
  - Document Date : Unknown
  - Type : report
  - ID : kggg0230 ( TID : ohf71j00 )
  - ARK : ark:/88122/kggg0230

182) Non Branded Round 2 – Final Report Review

Document Data

- Author : Jones, Susan T
- Document Date : 2008 April 10
- Type : email; notes
- ID : lmgg0230 ( TID : hlf71j00 )
- ARK : ark:/88122/lmgg0230
- Collection : Oklahoma Opioid Litigation Documents; Opioid Documents Collection

Document Notes

- **Pain Non-Branded Campaign Market Research – Round 2**
  - The Campaign = Non-Branded Message regarding the under treatment of pain, why so and what can lead to
  - The P3 Program = **Physicians Partnering Against Pain**
  - The Product = Tapentadol IR (NUCYNTA)
- **Market Research Objectives =** Test the non-branded campaign's ability to "impact" the market place behaviors/thinking relative to the treatment &. management of acute pain
  - To Explore physicians’ reactions to four messages presented by a sales representative
  - To understand the impact of the message regarding the undertreatment of acute pain and its implications
  - To understand the connection of educational campaign to the Tapentadol TPP (Triplicate prescription program???)
- **THE MESSAGES – RECAP (see screenshots)**
  - Message = Undertreatment of Pain
    - Behavioral Change = will attempt better assessment of acute pain
  - Message = Consequences
    - Behavioral Change = **More aggressive approach to treating (stronger dosing and meds);** desire to spread the message to other physicians
  - Message = Concerns that Hinder Effective Pain Mgmt
    - Behavioral change = Low abuse numbers
    - About half believe the addiction percentage (For the most part, the Rep was successful with refocusing them from addiction to side effect concerns)
  - Message = Multi-Pathways
    - NEW AGENTS – we have a new solution
    - Behavioral Change = **Increased opioid use (PCP), increase use of poly pharmacy until Product Y becomes available**
- **Impact of Campaign Overall =** PCPs state that they will be more aggressive in their treatment and use more opioids
- Physicians offer the following advice and suggestions on how to better present the educational campaign regarding acute pain management
  - Include thought leader presentations and peer-to-peer discussions in the campaign
- Reactions to Tapentadol IR TPP
  - Other benefits include efficacy similar to oxycodone, low withdrawal potential, **and some physicians on their own made a leap that it may result in lower addiction potential**
- Physicians report many patient types as undertreatment. These include: **the elderly**, **younger patients**, post-op and **post-trauma patients**
  - RECOMMENDATION = Include these patients in the undertreatment message
  - Key Points:
    - “Huge Opportunity for Ultram ER.
    - Tapentadol is a huge opportunity for corporation and all of us.
    - Once in a lifetime opportunity.
    - This is the time to be CRITICAL!
      - Questions that your customers may have?
      - Questions/Comments that you have?
      - What is the feedback/reaction from your customers?
    - Some of the re-alignment has to do with Tapentadol.
    - First new centrally-acting analgesic in 25 years.
    - Percocet is the competition.
    - Back pain is still the biggest issue.
    - Call on all current customers.
    - Opening is critical so don’t confuse customer. Separate Ultram ER and Tapentadol.
    - GOALS: Get customers ready for new products. When we launch the customers will only need the name to write.”
    - Market Prep Objectives
      - Heighten awareness of the under treatment of pain and it consequences.
      - Make side effects a consideration
      - 2 pathways are better than one alone
    - Market Development
      - Profile targeted MDs in preparation for launch
      - Reps gain an understanding of how physicians treat pain individually
      - Establish reps as pain experts”
- Use of Opioid Analgesics in Pain Management
  - [https://www.industrydocuments.ucsf.edu/docs/llgg0230](https://www.industrydocuments.ucsf.edu/drug/docs/#id=llgg0230)
  - Author : Candiotti, Keith; Janssen Pharmaceuticals, Inc
  - Document Date : Unknown
  - Type : article; website
  - ID : llgg0230 ( TID : rkf71j00 )
  - ARK : ark:/88122/llgg0230
  - Collection : Oklahoma Opioid Litigation Documents; Opioid Documents Collection
  - Key Points:
    - “*Expert authors received compensation from Janssen Pharmaceuticals, Inc. for their contributions to PrescribeResponsibly.com”*
    - “By the same token, patients report similar concerns about developing an addiction to opioid analgesics.^17^ While these concerns are not without some merit, it would appear that they are often overestimated. According to clinical opinion polls, true addiction occurs only in a small percentage of patients with chronic pain who receive chronic opioid analgesics analgesic therapy.^18^”
      - Auret K, Schug SA. Underutilisation of opioid analgesics of opioid analgesics in elderly patients with chronic pain: approaches to correcting the problem. *Drugs Aging.* 2005; 22(8): 641-654.
      - Fishbain DA, Cole B, Lewis J, et al. What percentage of chronic nonmalignant pain patients exposed to chronic opioid analgesics analgesic therapy develop abuse/addiction and/oraberrant drug-related behaviors? A structured evidence-based review. *Pain Medicine.* 2008; 9(4):444-459.
- Pain Action Alliance to Implement a National Survey
  - [https://www.industrydocuments.ucsf.edu/docs/qmgg0230](https://www.industrydocuments.ucsf.edu/drug/docs/#id=qmgg0230)
  - Author : Unknown
  - Document Date : Unknown
  - Type : report; report, scientific
  - ID : qmgg0230 ( TID : mlf71j00 )
  - ARK : ark:/88122/qmgg0230
  - Collection : Oklahoma Opioid Litigation Documents; Opioid Documents Collection
  - Key Points:
    - Pain Alliance to Implement a National Strategy (PAINS)
    - “Our Mission
      - To advocate for and act collectively to actualize the recommendations set forth in the Institute of Medicine’s report, Relieving Pain in America”
    - “The Undertreatment of Chronic Pain”
- Tapentadol Professional Education Message Platform
  - [https://www.industrydocuments.ucsf.edu/docs/spgg0230](https://www.industrydocuments.ucsf.edu/drug/docs/#id=spgg0230)
  - Author : Unknown
  - Document Date : Unknown
  - Type : report; presentation; slides
  - ID : spgg0230 ( TID : knf71j00 )
  - ARK : ark:/88122/spgg0230
  - Collection : Oklahoma Opioid Litigation Documents; Opioid Documents Collection
  - Key Points:
    - “Unbranded Message Platform
      - Acute moderate-to-severe pain is critically undermanaged
      - Tapentadol is a unique and novel centrally-acting analgesic with a dual mechanism of action
      - The efficacy safety and tolerability of tapentadol have been established in clinical trials
      - Two formulations of tapentadol will be available immediate0release and extended-release
      - Tapentadol as first-choice therapy for acute and chronic moderate-to-severe pain”
    - LARGE number of references
- PriCara – Non-Branded Promotion
  - [https://www.industrydocuments.ucsf.edu/docs/xhgg0230](https://www.industrydocuments.ucsf.edu/drug/docs/#id=xhgg0230)
  - Author : Ortho-McNeil, Inc
  - Document Date : Unknown
  - Type : presentation; slides
  - ID : xhgg0230 ( TID : cif71j00 )
  - ARK : ark:/88122/xhgg0230
  - Collection : Oklahoma Opioid Litigation Documents; Opioid Documents Collection
  - Key Points:
    - Entire slide deck on unbranded communication to prime market for tapentadol launch

204) Non-Branded Promotion

Document Data

- Author : Leonard, Lynn
- Document Date : 2007 June 29
- Type : diagram; email; report
- ID : fngg0230 ( TID : rlf71j00 )
- ARK : ark:/88122/fngg0230
- Collection : Oklahoma Opioid Litigation Documents; Opioid Documents Collection

Document Notes

- Email from Lynn Leonard
  - here are some slides on the unbranded efforts for the tactical presentation
  - I have included the "banana" analysis on one of the slides - which gets us to 60% of physician targets and gives us 60% of the business (targeted business).
- Preliminary Message Points
  - 1) Pain is mismanaged and under treated
  - 2) The consequences are serious and significant
  - 3) Appropriate treatment requires effective pain control
- Capturing and Maintaing Interest
  - Supported through lively stories – Illustrate patient/MD gap and highlight elderly patients

Slide Title: **PriCara Non-Branded Promotion**

- Disrupting a Complacent Marketplace
  - Web Strategy: Professional Promotional Platform + Physician-Patient Education/PR Strategy
- Disrupt the Marketplace
  - Heighten awareness of the under-treatment of pain and its consequences
  - Establish reps as pain experts
  - Profile targeted HCPs in preparation of launch
- Unbranded Message Continuum – Paving the way for tapentadol
  - 1) Pain is mismanaged and under-treated
  - 2) There are serious consequences of mismanaging & under-treating pain
  - 3) Appropriate treatment requires EFFECTIVE pain relief
  - 4) New pathways in pain management coming soon
- Pain Management: **At the Threshold of NEO Thinking**
- Gaining Endorsements for Credibility
  - Establish Instant Credibility
  - Develop Good Will
  - Alignment of Long Term Goals
  - Alleviate Regulatory Anxiety


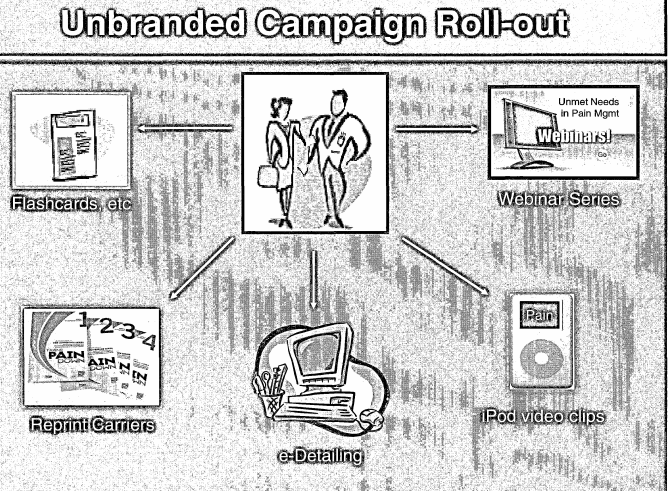


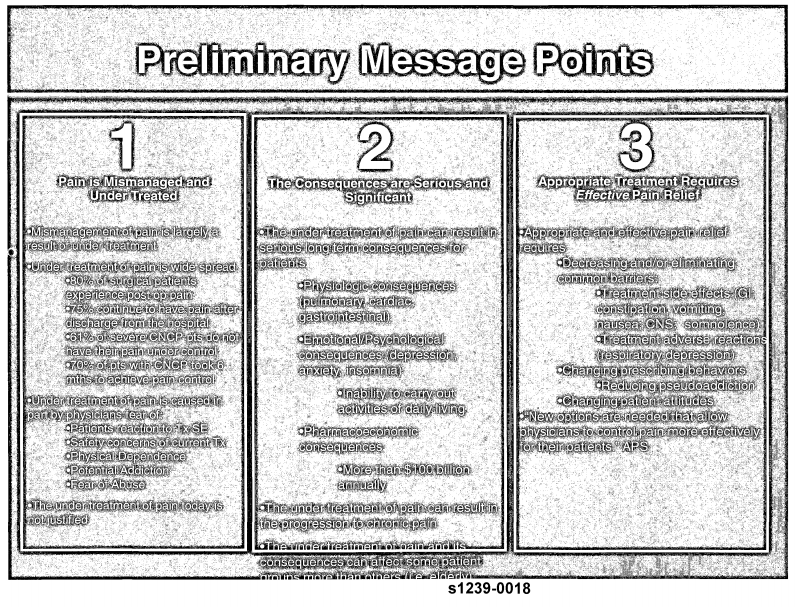


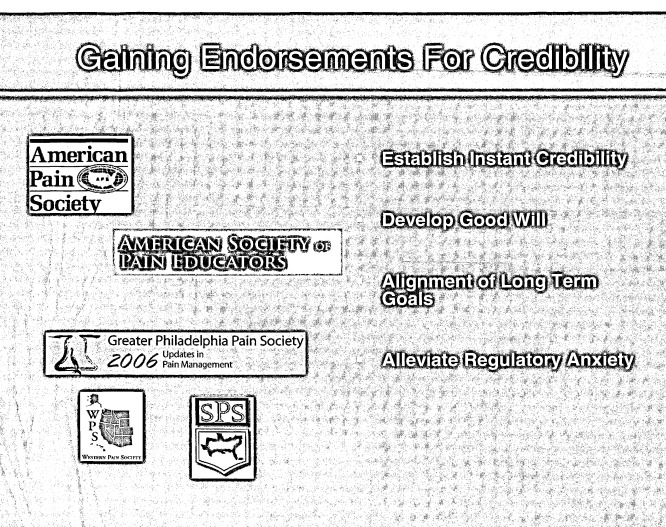


281) Analgesic Treatment for Moderate-to-Severe Acute Pain in the United States: Patients’ perspective in the Physicians Partnering against Pain (P3) Survey

Document Data

- Author : Journal of Opioid Management; Moskovitz, Bruce L; Benson, Carmela J; Patel, Aarti A; Chow, Wing; Kim, Myoung S; McCarberg, Bill H; Mody, Samir H
- Document Date : 2001 March 06
- Type : article; publication
- ID : khgg0230 ( TID : eif71j00 )
- ARK : ark:/88122/khgg0230
- Collection : Oklahoma Opioid Litigation Documents; Opioid Documents Collection

Document Notes

- **REMINDER – P3 = nonbranded initiative**
- Conclusion = In the P3 study, one of the largest outpatient surveys conducted in pain management, moderate-to-severe acute pain continued to be widely undertreated in outpatient settings in the United States, particularly among older patients. Opioids with improved tolerability profiles might help to alleviate this undertreatment of moderate-to-severe acute pain.
- **Published in 2011: Journal of Opioid Management**
- Work was funded by JANSSEN
  - The following authors are employees/paid by JANSSEN
    - **Bruce Moskovitz**
    - **Carmela Benson**
    - **Aarti Patel**
    - **Wing Chow**
    - **Samir Mody**
    - **Bill McCarberg**
    - **Myoung S Kim**


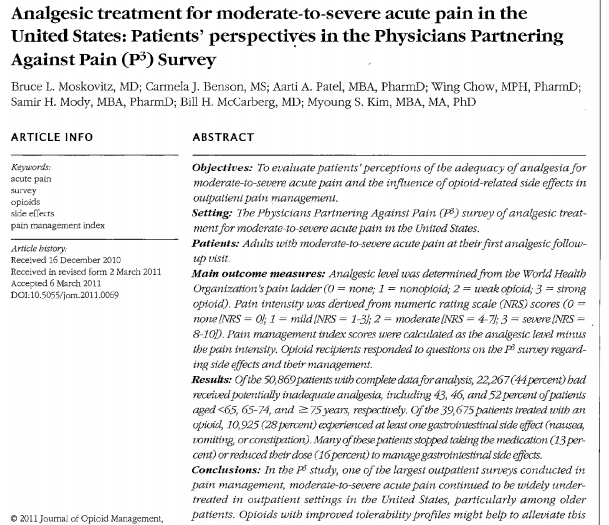


292) Myths about Opioids

Document Data

- Author : Purdue; Partners Against Pain
- Document Date : 1999
- Type : report; publication
- ID : ypgg0230 ( TID : gnf71j00 )
- ARK : ark:/88122/ypgg0230
- Collection : Oklahoma Opioid Litigation Documents; Opioid Documents Collection

Document Notes

- Booklet Title = **Dispelling the Myths about Opioids**
  - **This booklet describes the myths about opioids in an effort to dispel them and open the way for better opioid therapy**
  - Author = Partners Against Pain [Unbranded??]
    - *An educational service of The Purdue Frederick Company and Purdue Pharma*
- Key Terms
  - Pseudotolerance -- Pseudotolerance is the need to increase dosage that is not due to tolerance, but due to other factors such as: disease progression, new disease, increased physical activity, lack of compliance, change in medication, drug interaction, addiction, and deviant behavior. When a once-fixed opioid dose is no longer effective, the above conditions should be reviewed to exclude pseudotolerance.
  - Pseudoaddiction -- Pseudoaddiction is drug-seeking behavior that seems similar to addiction, but is due to unrelieved pain. This behavior stops once that pain is relieved, often through an increase in opioid dose. "Misunderstanding of this phenomenon may lead the clinician to inappropriately stigmatize the patient with the label 'addict.1 In the setting of unrelieved pain, the request for increases in drug dose requires careful assessment, renewed efforts to manage pain and avoidance of stigmatizing labels."
  - Note: Tolerance to or physical dependence on opioids, which may develop in a patient on extended opioid therapy, should not be confused with addiction (
- MYTHS ABOUT OPIOIDS
  - 1 -- Opioids depress respiratory function and are therefore too dangerous to be dosed safely.
    - FACT - While respiratory depression is the chief hazard of all opioid agonist preparations, clinically significant respiratory depression rarely occurs in patients in pain for whom doses of opioids are appropriately prescribed, administered, and titrated to effect.-
  - 2 -- Opioid addiction (psychological dependence) is an important clinical problem in patients with moderate to severe pain treated with opioids.
    - FACT -- Fears about psychological dependence are **exaggerated** when treating appropriate pain patients with opioids.
    - In a review of the records of 11,882 hospitalized patients treated with opioids, there were only four cases of addiction in patients with no addiction history. [**see #22 – letter to the editor; authors express regret]**
  - 3 -- Opioid tolerance develops rapidly, necessitating progressively higher doses.
  - 4 -- Adequate opioid doses cause unmanageable constipation.
  - 5 -- The majority of patients on opioids require prophylactic antiemesis.
  - 6 -- Unacceptable sedation and confusion are a frequent side effect in opioid patients.
  - 7 -- Short-acting opioids (3 to 6 hours) are the ideal analgesic for controlling moderate to severe pain.
  - 8 -- Controlled-release opioids should only be used for the management of cancer-related pain.
    - FACT -- Opioids can be used effectively in many types of non-cancer pain, such as back pain, osteoarthritis, post-operative pain, neuropathic pain, fracture/trauma, and reflex sympathetic dystrophy (RSD)
    - Either controlled-release oxycodone (**OxyContin® CII Tablets**) or controlled-release morphine sulfate (**MS Contin® CII Tablets**) have been used successfully to provide pain relief in patients with low back pain,23 osteoarthritis,23 post-herpetic neuralgia,23 chronic musculoskeletal pain," and AIDS pain
  - 9 -- Initiation of dosage and precise titration are difficult to achieve with controlled-release opioids.
  - 10 -- Severe cancer pain commonly calls far parenteral opioids.
  - 11 -- Plasma opioid concentrations correlate directly with the level of analgesia.
- **WAYBACK MACHINE – Partnersagainstpain.com**
  - Targeting elderly, dementia, hospice patients

**GHOSTWRITING**

10) Payment History of J&J or Janssen to Specific Individuals

- Document Data
  - **Author :** Janssen
  - **Document Date :** 2019 January 25
  - **Type :** report; legal; table
  - **ID :** rngg0230 ( TID : dmf71j00 )
  - **ARK :** ark:/88122/rngg0230
  - **Collection :** Oklahoma Opioid Litigation Documents; Opioid Documents Collection
- Document Notes
  - Dr. Charles Argoff – Psychiatry & Neurology & Chronic Pain
    - Activity = Advisor, consultant
    - Contributor to *Prescribe Responsibly*
    - Payment = $37000
  - Dr. C Keith – Anesthesiology
    - Activity = Advisor, consultant
    - Contributor to *Prescribe Responsibly*
    - Payment = $13000
  - Dr. F Pine – Anesthesiology & Chronic Pain
    - Activity = Advisor, consultant
    - Contributor to *National Pain Education Council (NPEC)*
    - Payment = $35000
  - Dr F Scott – Anesthesiology, Hospice, Palliative Medicine
    - Activity = Advisor, consultant
    - Contributor to *Let’s Talk Pain*
    - Payment = $2000
  - Dr N Katz – Anesthesia
    - Activity = Advisor, consultant
    - Payment = $5000
  - Dr B McCarberg – Pain Management
    - Activity = Advisor, consultant
    - Contributor to *Let’s Talk Pain*, patient counseling education video, NPEC, many others things for Janssen
    - Payment = $27,000

61) Greetings and Questions

Document Data

Document Notes

- Email from James Heins (senior director of corporate affairs & communication at Purdue) to Dr Charles Argoff (See #10 and #60)
- James Hein **wrote the entirety of Argoff’s “quote” and Argoff signed off on it with a simple “Excellent. Well done. I have no suggested changes”**
  - The Argoff quote (from #60): "The burden of chronic pain and the abuse of prescription medications are both pressing societal problems," said **Charles E. Argoff,** MD, Professor of Neurology at Albany Medical College and Director of the Comprehensive Pain Center at Albany Medical Center in New York. "Opioids are an essential tool in our arsenal of medical treatments options, so greater availability and use of opioid analgesics with abuse-deterrent properties has the potential to help alleviate suffering among people with chronic pain while reducing the abuse of these medications. Furthermore, this product gives treatment providers the option to use hydrocodone without acetaminophen if they are concerned that their patients may be taking too much acetaminophen on a daily basis."
- ARGOFF SEEMS TO HAVE NO REAL RELATIONSHIP WITH HYSINGLA BEFOREHAND AT ALL!!!
  - From Gina Barbarotto: *Hi Dr. Argoff. As you know, we are expecting a new product approval soon.... I want to assess your interest in talking to the media about this approval and potentially the following... Is this something that would be of interest to you?*"
  - Argoff’s Response: “I was in Argentina at IASP when you sent. Thanks for asking. I am”
    - Argoff seems to have no real relationship with the research for Hysingla, but he happily puts his name (which carries lots of weight as Professor of medicine at a top US MD school) to vouch for the med in the press release.
- Implication: Purdue press release (#60) for Hysingla used Argoff’s quote to show “expert” opinion regarding the new drug. In reality, Purdue wrote the quote for Argoff, who simply ok’d it via email, AND argoff has been getting $$$$ from Purdue for quite some time.

Document Relevance = YESSSSS

70) Email from Felice Sweeney to Patricia Cosler Regarding the Chronic Pain Management Strategies and Lessons from the Military

Document Data

- **Author :** Sweeney, Felice
- **Document Date :** 2013 May 24
- **Type :** email
- **ID :** jqgg0230 ( TID : rnf71j00 )
- **ARK :** ark:/88122/jqgg0230
- **Collection :** Oklahoma Opioid Litigation Documents; Opioid Documents Collection

Document Notes

- “Chronic Pain Management Strategies and Lessons From the Military: A Narrative Review” has been successfully submitted to the *Annals of Internal Medicine*
- Email from Felice Sweeney --> **employee at Medergy Scientific, Inc**
  - [https://www.dnb.com/business-directory/company-profiles.medergy_scientific_inc.332a3021c3bf8b1f999e7b9f01cdbfad.html﷟HYPERLINK "https://www.dnb.com/business-directory/company-profiles.medergy_scientific_inc.332a3021c3bf8b1f999e7b9f01cdbfad.html"](https://www.dnb.com/business-directory/company-profiles.medergy_scientific_inc.332a3021c3bf8b1f999e7b9f01cdbfad.html%22%EF%BF%BDHYPERLINK%20%22https://www.dnb.com/business-directory/company-profiles.medergy_scientific_inc.332a3021c3bf8b1f999e7b9f01cdbfad.html)
    - **Medergy Scientific, Inc.** is located in Yardley, PA, United States and is part of the Advertising & Marketing Services Industry. Medergy Scientific, Inc. has 40 total employees across all of its locations and generates 0 million in sales (USD).
  - <https://www.linkedin.com/company/medergy-healthgroup>
- Email sent to the Authors of the paper + “Gary Baker”
  - Can’t find who exactly Gary Baker is??
  - Authors listed on the paper: April Hazard Vallerand, Patricia Cosler, Jack E Henningfield, Pam Galassini
- Paper: “Chronic Pain Management Strategies and Lesson from the Military: A Narrative Review”
  - **ABSTRACT: The application of US military pain management guidelines has been shown to improve pain monitoring, education and relief. In addition, the US military has instituted the development of programs and guidelines to ensure proper use and discourage aberrant behaviours with regard to opioid use, because opioids are regarded as a critical part of acute and chronic pain management schemes. Inadequate pain management, particularly inadequate chronic pain management, remains a major problem for the general population in the US. Application of military strategies for pain management to the general US population may lead to more effective pain management and improved long-term patient outcomes**
  - The paper heavily repeats the major talking points from Johnson and Johnson
    - Opioids are needed in CHRONIC pain management
    - Abuse potential for opioids are limited and can be avoided
    - The REAL problem is inadequate pain management
  - **Acknowledgements**: Editorial support for manuscript preparation was provided by Megan Knagge, PhD, of MedErgy, and was funded by Janssen Scientific Affairs, LLC. The authors retained full editorial control over the content of the article. The authors acknowledge the members of the **Imagine the Possibilities Pain Coalition (Coalition started and funded by Jansenn!!!! See #82)** for their helpful discussions and critical review of this manuscript: Gary Baker, Jeff Buel, Arthur Caplan, Penney Cowen, Myra Glajchen, Phyllis Grauer, Myoung Kim, Robyn Kohn, Richard Payne, Jennifer Pluim, Frank Sapeinza, Scott Taylor, Robert Twillman, Jon Ukropec, Gary Vorsanger, Behin Yektashenas.
  - **Disclosures**: P Cosler was an employee of Janssen Scientific Affairs, LLC, at the time of the writing of the manuscript and declares no current conflicts of interest. AH Vallerand and P Galassini declare no conflicts of interest. JE Henningfield provides consulting services through Pinney Associates to pharmaceutical developers and marketers of central nervous system-acting drugs, including analgesics, to help assess the abuse liability of the drugs and develop appropriate recommendations for labelling and risk management to minimize the risks of abuse, overdose and other unintended effects.

Document Relevance = YES

92) Letter from Robyn Kohn to Tricia Haertlein enclosing Meeting 2

Document Data

- **Author :** Kohn, Robyn
- **Document Date :** 2011 November 01
- **Type :** email; presentation; slides; report
- **ID : mmgg0230**

Document Notes

- Meeting of **Imagine the Possibilities: Pain Coalition**
- 3 subteams charged with creating message
  - MEDIA OUTREACH – Targets = **YOUTH, VETERANS**, PUBLIC
  - POLICY/ADVOCACY
    - Peer-reviewed publication in health policy journal
- Messaging: **Chronic pain as public health problem, chronic pain is multi-contextual**
- Advocacy + Pain Policy Sub-Team Platform
  - **Chronic pain is the #1 public health problem**
  - **Epidemic of Pain v Epidemic of Addiction**
  - **Wide dissemination of plan**
    - Advocacy magazines – AAPM Currents, AAFP Live, AAN Neurology Today
    - General public – Op-eds, popular media, retail chains (**Starbucks pain message of the day????)**
- Education Sub-Team Platform
  - Major needs
    - Placard for HCP so they keep pain conversation front of mind (should fit in lab coat)
    - Quick Guide geared toward community-based pharmacists
    - Medical school curriculum development grant programs
- MEdia Outreach Initiatives
  - Reaching out to: **YOUTH**
    - Reach early – elementary school level – via respoected channels (coaches)
    - Delivery a practical message: **pain is your body telling you something important**
  - Reaching out to: **RETURNING VETERANS**
    - Where do they get their info about pain?
    - Change the paradaign: **the positive side of pain management**
  - Reaching out to: **MEDIA**
    - Capture venues that the media want to cover: art cretaed by people in pain
    - Emphazie the problem of **poorly managed pain is often lost to the topic of addiction fear**; even though it [poorly managed pain] is an issue of many magniutes greater concern [than addiction]
- Teams and Members
  - **MEDIA OUTREACH = “destigmatize pain”**
    - **Jack Henningfield**
    - **Patricia Cosler**
    - **Apriler Vallerand**
    - **Pam Galassini**
    - ^^^**These are all authors of the Military & Chronic Pain paper!!**
  - **PUBLIC POLICY = “change the conversation about pain”**
    - **Bob Twillman**
    - Robyn Kohn
  - **EDUCATION = “deliver the education”**
    - Art Caplan
    - Myra Glajchen
    - Gary Baker
    - Richard Payne
    - Penny Cowan
- Declaration of Russell K Portenoy, MD in State of Oklahoma v Purdue Pharma LP, et al
  - [https://www.industrydocuments.ucsf.edu/docs/nshg0230](https://www.industrydocuments.ucsf.edu/drug/docs/#id=nshg0230)
  - Author : District Court of Cleveland County, State of Oklahoma
  - Document Date : 2010 January 17
  - Type : legal; affidavit
  - ID : nshg0230 ( TID : xyf71j00 )
  - ARK : ark:/88122/nshg0230
  - Collection : Oklahoma Opioid Litigation Documents; Opioid Documents Collection
  - Key points:
    - “I have agreed to cooperate with certain plaintiffs who have entered into settlement agreements with me dismissing me as a defendant in their cases (‘Settling Plaintiffs’). Settling Plaintiffs agreed to dismiss me from their cases in exchange for my truthful cooperation. The proffer agreement with those Plaintiffs can be voided and the original lawsuits may be reinstated against me if my statements are recklessly and materially not truthful or accurate.”
    - “This declaration includes statements describing how my views about opioid therapy and its marketing by the pharmaceutical companies have changed during the period between the mid-1980s and the first decade of the 2000s.”
    - “Prior to, and then during the 1980s, opioids were disfavored for use in chronic, noncancer pain because of concerns that patients using opioids would develop tolerance and physical dependence, and be at risk for abuse, misuse, addiction, and diversion.^1^”
      - Portenoy RK, Foley KM. Chronic use of opioid analgesics in non-malignant pain: report of 38 cases. Pain. 1986;25:171-86.
    - Portenoy RK, Foley KM. Chronic use of opioid analgesics in non-malignant pain: report of 38 cases. Pain. 1986;25:171-86.
      - “This article was a retrospective case series describing anecdotal information about a highly selected group of patients. I recall that Dr. Foley and I wanted to write the paper to describe a phenomenon that we believed was under-appreciated by the medical community—the possibility of long-term pain relief from opioid therapy, without the development of tolerance leading to treatment failure and without the development of serious adverse effects, including drug abuse. We also wanted to use this description of cases as a starting point for a broad discussion of the clinical issues relevant to the appropriate use of these drugs. Our initial contribution to this discussion appeared in the Discussion section of the paper, which ended with our recommendation that opioid therapy be considered only after "all reasonable attempts at pain control have failed and persistent pain is the major impediment to improved function."^3^ Contrary to how some drug companies later used this article, it was never intended as a report of high-quality evidence, or as support for broad adoption of opioid therapy; it was a description of anecdotal information accompanied by a brief narrative review of the literature, and was intended to suggest that the role of long-term opioid therapy needed re-thinking, and more research, and that clinicians should not consider the approach to be contraindicated, but rather, worthy of consideration in the context of treatment refractory pain.”
    - “In my 1994 chapter, for example, I described a phenomenon that I then called *therapeutic dependence* and a phenomenon that had been labeled as *pseudoaddiction* in a 1989 article by Weissman and Haddox.^6^”
      - Weissman DE, Haddox JD, Opioid pseudoaddiction. Pain 1989;36(3):363-366.
    - “In all my writings, I acknowledged that the disease of addiction was a risk when opioids were used therapeutically. I stated in the 1994 chapter, as I did in many other writings during the 1980s and 1990s, that the disease of addiction would be "very unlikely" to develop when patients with chronic pain and no prior history of substance abuse were prescribed opioids and closely monitored. Although the evidence in support of this statement was scant in terms of the populations with chronic non-cancer pain, it was supported by large surveys of cancer patients receiving opioids for cancer pain. My conclusion was based on an analysis of the clinical literature at the time, which I acknowledged was very limited.”
    - “In 1996, a workgroup was formed by the American Academy of Pain Medicine and the American Pain Society for the purpose of creating a *Consensus Statement on the Use of Opioids for the Treatment of Chronic Pain*. The committee chair was Dr. J. David Haddox, who was hired by Purdue Pharma in 1999. I was not involved in the deliberations of the workgroup, but I recall that I was sent a draft of the consensus statement for my comments before it was presented to the respective organizations for approval and dissemination, I do not recall whether I made suggestions for modifications.”
    - {From p.15, a lengthy description of the nature of financial relationships with Defendants}
    - “I recall that some of these offers to write review articles, which I did not pursue, included the help of a medical writer for assistance in drafting the paper.”
    - “Based on the interactions that I have had with medical education vendors, I believe that academicians who are provided with honoraria for producing or editing material must be vigilant to avoid messages that are not well supported or prudent, and are in the interest of the drug company, without a corresponding medical benefit for the patient. Although I cannot cite specific cases, my experience suggests that some of the work ostensibly created by academicians through interaction with medical education vendors reflects the work or the influence of drug companies.”
    - “This type of controlled clinical trial provides what is considered to be highquality evidence. This evidence is needed by clinicians to confirm that the drugs they select can be efficacious when they are treating patients. However, these studies recruit patients based on strict inclusion criteria, and consequently, the results may or may not be immediately transferable to practice. In studies of opioids, the patients who are recruited are carefully selected and may not be representative of the overall population with pain; moreover, the duration of study is short compared to the patients treated in practice, and study patients are carefully monitored while they are receiving the study drugs. Clinical practice needs randomized controlled studies to provide an evidence base for patient care, but clinical guidelines are not created solely from the data acquired in these studies. It is my belief that this understanding of the role played by high-quality randomized clinical trials, i.e., necessary to establish the potential for efficacy but insufficient for clinical guidelines that must consider a patient population not represented in the study and patterns of drug use that are not specifically tested, is widely accepted by clinicians and investigators, and would be considered common knowledge in the pharmaceutical industry.”
    - “Addiction can exist while pain is experienced.”
    - “I believe that, over the years, some Defendant drug companies have used my work to promote opioids by referencing the positive statements that 1 made repeatedly without providing the background, analysis of the literature, and cautions that accompanied these positive statements.”
    - “It indicates that the risk of addiction is <1%, although this is an inaccurate interpretation of data (paragraph 40)…”
    - “Drug companies are a major source of research funding and have the ability to influence study proposals. In my opinion, it is clear that drug company research grants provided to academicians for studies of approved drugs generally fund studies that aim to identify or confirm benefits that would be helpful in marketing. Similarly, 1 believe that the drug companies distribute honoraria, fees and grants in a way that elevates specific messages, and messengers, that agree with their preferred messaging. Although I personally was never influenced to say things I did not believe, it is true that the drug companies provided me with many opportunities to express my views, and they used the positive statements that I made about opioids to portray opioid treatment as safe and effective without the accompanying discussion of risk that I included in the papers, chapters, and lectures I produced beginning in the 1980s.”
    - “My work was intended to disabuse clinicians of a bias against opioids by describing the literature as it existed then and the favorable outcomes that I and others were seeing in varied subgroups of patients, and by providing guidelines for treatment that included careful patient selection and vigilant monitoring of drug-related outcomes over time. I believe that the drag companies created material that narrowly focused on the potential for safe and effective treatment of chronic noncancer pain, some of which was attributed to my work, but failed to include an adequate and balanced discussion of the limitations in the relevant science and the risks as they were then known.”
    - “The practice of "enriched enrollment," which is a type of clinical trial design often used in pivotal trials supporting FDA approval of an opioid, exemplifies how these explanatory trials do not provide the type of information about risk that is needed in clinical practice. In these studies, there is an initial open-label phase to select patients who respond to a drug; this is then followed by a double-blind phase to determine whether these responders continue to have benefit from the drug when their effects are compared with a placebo treatment. Patients with addiction risk are typically screened out of the open-label phase, and patients who do not do well during the open-label phase are also dropped from the double-blind phase, Enriched enrollment studies are therefore designed only to see if a drug has a primary outcome, like analgesia, that exceeds placebo in a selected group of favored patients. It is not designed to assess outcomes in the heterogeneous population of patients seeking care in clinical practice, nor is it designed to assess long-term risks. Although drag companies should include documentation of evidence from explanatory trials in their labels, they should be cautious in pointing to these studies as proof of a drug's safety in real world conditions,17”
- Evaluation of Long-Term Efficacy and Safety of Transdermal Fentanyl in the Treatment of Chronic Non cancer Pain
  - [https://www.industrydocuments.ucsf.edu/docs/rsgg0230](https://www.industrydocuments.ucsf.edu/drug/docs/#id=rsgg0230)
  - Author : Milligan, Keith; Lanteri-Minet, Michael; Borchert, Klaus; Helmers, Henk; Donald, Royden
  - Document Date : Unknown
  - Type : article; graph; report, scientific
  - ID : rsgg0230 ( TID : fpf71j00 )
  - ARK : ark:/88122/rsgg0230
  - Collection : Oklahoma Opioid Litigation Documents; Opioid Documents Collection
  - Key points:
    1. International, multicenter, open-label trial
    2. Objective: “assess the efficacy and safety of up to 12 months of therapy with transdermal therapeutic system (TTS) fentanyl in patients (n=532) with chronic noncancer pain.”
    3. Main outcome measures: “pain control assessment, global treatment satisfaction, patient preference for TTS fentanyl, and quality of life.”
    4. Authors:
       1. Keith Milligan – Anaesthetic Department, South Cleveland Hospital, Middlesbrough, UK
       2. Michel Lanteri-Minet – Department of Pain Treatment, Hôpital Pasteur, Nice, France
       3. Klaus Borchert – Anästhesiologische Praxis, Greifswald, Germany
       4. Henk Helmers – Department of Anaesthesiology, Eemland Hospital, Amersfoort, The Netherlands
       5. Royden Donald – Strand Private Hospital, Cape Town, South Africa
       6. Hans-Georg Kress – Universitätsklinik für Allgemeine Anästhesie und Intensivmedizin, Abt. B, Wien, Austria
       7. Hugo Adriaensen – Universitair Ziekenhuis Antwerpen, Edegem, Belgium
       8. Dwight Moulin – Neurology Department, Victoria Hospital, London, Canada
       9. Voitto Järvimäki – Pain Clinic, Oulu University, Central Hospital, Oulu, Finland
       10. Ludo Haazen – Janssen Research Foundation, Beerse, Belgium
    5. “Supported by a grant from the Janssen Research Foundation, Belgium. K. M. has been reimbursed by Janssen-Cilag, the manufacturers of transdermal fentanyl, for attendance at a congress. L. H. was employed by the Janssen research Foundation when this study was performed.”
    6. Introduction
       1. “Evidence suggests, however, that pain is frequently undertreated even for these indications {acute pain and cancer pain}.^2,3^”
       2. “Persistent, albeit largely unfounded, fears about the risks of addiction, toxicity, physical dependence, and tolerance have led to the rejection of opioid analgesia for chronic pain resulting from noncancer disease.^4^”
       3. “Although few prospective clinical studies of opioid use in noncancer pain have been reported, evidence from surveys suggests that long-term opioid therapy can be used successfully in some patients with chronic nonmalignant pain without causing undue adverse events (AEs),^4-6^ a conclusion supported by several recent short-term, randomized controlled trials.^7-9^”
       4. “Reflecting this evidence and changing attitudes toward the therapeutic goals of treating noncancer pain, guidelines for the use of opioids in this setting have recently been proposed.^10-11^ These advocate scheduled around-the-clock drug administration and the use of long-acting preparations or sustained-release formulations to maintain therapeutic serum concentrations.”
    7. Materials and Methods
       1. “Exclusion criteria included a history of allergy or hypersensitivity to opioids; life-threatening disease; skin disease precluding the use of the transdermal system; reduced level of consciousness or inability to give informed consent; pregnancy, lactation, or possibility of pregnancy; social isolation; concomitant psychiatric disorders (depression excluded); history of substance abuse (assessed from the patient's clinical history and communication with the primary care practitioner); history of clinically relevant cardiac, nervous system, or respiratory disease or participation in any clinical trial, except the Allan study,^18^ within the preceding 30 days.”
    8. Statistical Analyses
       1. “The sample size of 500 patients was based on an estimated 2% incidence of AEs to provide a narrow and precise 95% confidence interval of 0.8% to 3.22%.”
    9. Results
       1. “A total of 532 patients were recruited, of whom 103 had participated in the study by Allan et al^18^;”
       2. “Of the 532 patients recruited, 301 (57%) patients completed the trail and 231 (43%) patients discontinued treatment prematurely.”
       3. “A total of 130 (25%) of the safety analysis population (n=530) discontinued TDF as a result of AEs.”
       4. “The most commonly used opioids immediately before study entry were morphine (48%) and fentanyl (28%, including patients from the Allan study^18^).”
       5. “A total of 375 of 421 (89%) patients stated a treatment preference (patients participating in the Allan study^18^ and those already on TTS fentanyl were not asked to express a preference).”
    10. Discussion
        1. “Stability of pain control during the 12-month period was achieved at the cost of an increase in mean TDF dosage from 48 to 90 μg/h. The largest increase occurred during the first months of treatment, as the patients titrated the dose upward themselves, and then stabilized during prolonged treatment. This increase may be due to the individual or combined effects of using conservative conversion table, underdosing at baseline, and the development of tolerance.”
        2. “Unlimited access to rescue medication, which is not always standard clinical practice, could have led to patients titrating the dose themselves to best effect, and consequently a subsequent increase in TDF dose when daily consumption of rescue morphine reached 60 to 90 mg. The average daily consumption of rescue medication was a reflection of increased pain intensity because it was probably related to an increased or altered physical function and subsequent pain at varying times.^10^ Experience with opioids has shown that tolerance to their analgesic effects is uncommon in patients with chronic pain.^10,23^ Many surveys and supportive clinical experience regarding the long-term use of opioids in patients with non-cancer pain have shown the development of tolerance not to be a clinical problem.^6,24^”
        3. “Opioid withdrawal symptoms were only reported in 3% of patients, suggesting that prolonged treatment is not associated with increased risk of withdrawal syndromes. There were no reports of addictive behavior in any of the patients during this long-term study. Because the fear of addiction is one of the reasons for the underuse of opioids in chronic noncancer pain, this study provides further evidence that these fears are unfounded. Our results also confirm that there is no basis for concern about persistent opioid side effects and long-term toxicity.^6^”
    11. Acknowledgments

“We thank all of the other investigators who participated in the trial: **J. Mayaert, L. Plaghki** (Belgium); **J. Clark,** H. Hays, **A. Mailis,** **M. Ong-Lam, D. Reid, P. Watson** (Canada); **E. Kalso** (Finland); N. Attal, F. Boreau, L. Brasseur, J. Bruxelle, P. Ginies, E. Lajous, S. Perrot, A. Serrie (France); **L. Allan**, **R. Atkinson**, G. Batchelor, **F. Campbell**, G. Carmichael, B. Collett, H. Dunckley, M. Fallon, I. Finlay, W. Fitch, **R. Gautam**, **M. Hanna**, D. Laird, D. Littlewood, W. Loan, K. Markham, L. Morrison, **W. Notcutt**, K. Rogers, P. Selby (UK); A. Beyer, W. Dinter, L. Petracic (Germany); and **G. Braak,** P. Brouwer, **G. van Oss, W. Zuurmond** (The Netherlands). We also thank the following contributors : At Janssen Research Foundation: L. Haazen was responsible for international trial supervision and coordination and was involved in the writing of the article (he will act as guarantor); **D. Peelmans**, F. Spensieri, **N. Currie, M. Jarvinen**, B. Flaisler, I. Broudic, A. Bessems, A. Schmidt-Mertens , D. Tseneklidou-Stoeter, **M. Travers**, D. Mathewson, D. Coakes, **M. Uitendaal**, W. Schipper, **P. Matthysen** (trial coordination and monitoring); and H. Swinnen, E. Everaert, I. van den Broeck, M. Nijs, **L. Bijnens**, H. Joosen (clinical data processing).”

- - - 1. No Janssen names returned results in DIDA
    1. References

1. Cleeland CS: Undertreatment of cancer pain in elderly patients. JAMA 279:1914-5, 1998
2. Zenz M, Zenz T, Tryba M, Strumpf M; Severe undertreatment of cancer pain: A 3-year survey of the German situation. J Pain Symptom Manage 10:187-91, 1995
3. Portenoy RK: Chronic opioid therapy in nonmalignant pain. J Pain Symptom Manage 5(suppl):546-62, 1990
4. France RD, Urban BJ, Keefe FJ: Long-term use of narcotic analgesics in chronic pain. Soc Sci Med 19:1379-82, 1984
   1. n=16
5. Portenoy RK: Opioid therapy for chronic nonmalignant pain: A review of the critical issues. J Pain Symptom Manage 11:203-17, 1996
6. Arkinstall W, Sandler A, Goughnour B, Babul N, Harsanyi Z, Drake A: Efficacy of controlled-release codeine in chronic non-cancer pain: A randomized, placebo-controlled trial. Pain 62:169-78, 1995
   1. FUNDED BY PURDUE
7. Jadad A, Carroll D, Glynn C, Moore R, McQuay H: Morphine responsiveness of chronic pain: Double-blind randomised crossover study with patient-controlled analgesia. Lancet 339:1367-71, 1992
   1. n=10
8. Moulin D, lezzi A, Amireh R, Sharpe W, Boyd D, Merskey H: Randomised trial of oral morphine for chronic non-cancer pain. Lancet 347:143-7, 1996
   1. FUNDED BY PURDUE
   2. n=46
9. Portenoy RK: Opioid therapy for chronic nonmalignant pain: Current status, in Fields HL, Liebeskind JC (eds): Progress in Pain Research and Management; Pharmacologic Approaches in the Treatment of Chronic Pain: New Concepts and Critical Issues. Seattle, WA, IASP Press, 1994
10. Schug S, Merry A, Acland R: Treatment principles for the use of opioids in pain of non-malignant origins. Drugs 42:228-39, 1991
    1. Behind paywall
11. Allan L, Hays H, Jensen N H, de Waroux BLP, Bolt M, Donald R, Kalso E: Randomised crossover trial of transdermal fentanyl and sustained release oral morphine for treating chronic non-cancer pain. BMJ 322:1154-1158, 2001
12. Coyle N, Adelhardt J, Foley KM, Portenoy RK: Character of terminal illness in the advanced cancer patient: Pain and other symptoms in the last 4 weeks of life. J Pain Symptom Manage 5:83-93,1990
13. Collett B-J: Opioid tolerance: The clinical perspective. Br J Anaesth 81:58-68, 1998
14. Galer BS, Coyle N, Pasternak GW, Portenoy RK: Individual variability in response to different opioids: Report of five cases. Pain 49:87-91, 1992
15. Randomised Crossover Trial of Transdermal Fentanyl and Sustained Release Oral Morphine for Treating Chronic Non-Cancer Pain
    1. [https://www.industrydocuments.ucsf.edu/docs/qsgg0230](https://www.industrydocuments.ucsf.edu/drug/docs/#id=qsgg0230)
    2. Author : Allan, Laurie; Hays, Helen; Jensen, Niels-Henrik; de Waroux, Bernard Le Polain; Bolt, Michiel; Kalso, Eija; Donald, Royden
    3. Document Date : 2001 May 12
    4. Type : publication; article
    5. ID : qsgg0230 ( TID : epf71j00 )
    6. ARK : ark:/88122/qsgg0230
    7. Collection : Oklahoma Opioid Litigation Documents; Opioid Documents Collection
    8. Key Points:
       1. Authors
          1. Laurie Allan – (director) Chronic Pain Services, Northwick Park and St. Mark’s NHS Trust, Harrow, Middlesex HA1 3UJ
          2. Helen Hays – (associate clinical professor) Department of Family Medicine, University of Alberta, Edmonton, Alberta, Canada T6G 2C8
          3. Niels-Henrik Jensen – (head of department) Multidisciplinary Pain Centre, Department of Anaesthesiology, Herlev University Hospital, DK-2730, Denmark
          4. Bernard Le Polain de Waroux – (staff anaesthesiologist) Clíniques Universitaires St-Luc, 1200 Brussels, Belgium
          5. Michiel Bolt – (anaesthesiologist) Alg Ziekenhuis Eemland De Lichtenberg, 3818 ES Amersfoort, Netherlands
          6. Royden Donald – (specialist anaesthetist) Strand Private Hospital, Cape Town 7139, South Africa
          7. Eija Kalso – (head) Helsinki University Central Hospital Pain Clinic, 00290 Helsinki, Finland
       2. “Objectives: To compare patients’ preference for transdermal fentanyl or sustained release oral morphine, their level of pain control, and their quality of life after treatment.”
       3. “Design: Randomised, multicenter, international, open label, crossover trial.”
       4. “Main outcome measures: Patients’ preference for transdermal fentanyl or sustained release oral morphine, pain control, quality of life, and safety assessments.”
       5. Introduction
          1. “Pain is often undertreated or mistreated, with patients going from doctor to doctor for relief and finally moving outside mainstream medicine in increasing numbers.^2”^
          2. “Opioids are the mainstay of management of cancer pain, providing effective pain relief.^3 4^ Opioids are the most powerful analgesics, but politics, prejudice, and continuing ignorance still impede optimum prescribing.^5^ A review of retrospective and survey data confirms the efficacy of opioids in the treatment of chronic non-cancer pain and found that fears of addiction were not justified.^6^ Randomised controlled trials of intravenous opioids in chronic non-cancer pain show benefit over placebo for morphine and fentanyl, whereas oral placebo controlled trials show efficacy for codeine, morphine, and oxycodone.^7–11^ Worldwide, the value of opioids in this role has led to the development of management guidelines, with recommendations from national organisations.^12–15^”
       6. Participants and methods
          1. “At crossover, patients received the same opioid dose as before the study.”
          2. “Patients were prescribed immediate release morphine (initially 5 mg) every four hours as needed. Patients requiring more than 60 mg of this rescue drug over two days of a three day period with fentanyl could increase their fentanyl dose. Patients receiving morphine needing more than two doses of the rescue drug per day could titrate to a higher dose of morphine.”
          3. “Details of all adverse events and presumed relation to the drugs were noted by the investigator.”
       7. Results
          1. “Sixty patients withdrew; 37 because of adverse events, five because of insufficient efficacy, and 18 for other reasons. Five patients without baseline data were excluded from the efficacy analysis. All patients were included in the safety analysis.”
          2. “The mean starting dose of transdermal fentanyl was 39.7 ìg/hr (range 25-200 ìg/hr) and of sustained release oral morphine 123.0 mg/24 hr (range 10-700 mg/24 hr). The mean dose of fentanyl at the end of the study was 57.3 ìg/hr (range 0-325 ìg/hr) and of morphine 133.1 mg/24 hrs (range 0-800 mg/24 hrs).”
          3. “The predominant reason given for preferring fentanyl was better pain relief, followed by greater convenience and fewer adverse events (table 3).” “Patients treated with transdermal fentanyl had on average lower pain intensity scores than those treated with sustained release oral morphine (mean 57.8, range 33.1-82.5 v mean 62.9, range 41.2-84.6; P < 0.001), irrespective of the order of treatment.” “Analysis of the consumption of rescue drug during the last three weeks of each treatment period showed that the mean (standard deviation) consumption was significantly higher with fentanyl (29.4 (33.0) mg) than with morphine (23.6 (32.0) mg; P < 0.001). A significant (P < 0.05) period effect was also observed: the higher consumption during fentanyl treatment was more apparent in the second trial period (mean 32.4 (SD 38.5) mg) than the first (26.3 (26.0) mg), where the consumption of the rescue drug remained essentially the same over the two treatment periods in the morphine group (23.7 (35.3) mg v 23.6 (27.3) mg).”
       8. Discussion
          1. No discussion of abuse potential or addiction throughout
       9. Acknowledgments
          1. “We thank all the investigators who participated in the trial: **J Maeyaert, L Plaghki** (Belgium); **J Clark**, A Mailis, **D Moulin**, **M Ong-Lam**, **D Reid, P Watson** (Canada); S Andersen, C Christiansen, S Clemensen, K Glahn, T Jonsson, S Larsen, F Molke Borgbjerg, A Schou Olesen, J Mølgaard (Denmark); V Järvimäki, T Heiskanen (Finland); **R Atkinson**, P Brown, **F Campbell**, **R Gautam, M Hanna**, D Hughes, C Knight, **W Notcutt** (United Kingdom); **G Braak**, J Helmers, **G Van Oss, W Zuurmond** (Netherlands); D Lines (South Africa). Contributors: LA was the principal author of the paper. H Noorduin (international supervisor of the trial), **L Bijnens** (biostatistics), **L Haazen, M Travers, D Peelmans, N Currie,** A Jepsen, **M Jarvinen, M Uitendaal, P Matthysen** (local trial coordination and monitoring). MT will act as guarantor for the paper. Funding: The study was supported by a grant from Janssen Research Foundation, Belgium. Competing interests: LA receives support from both Janssen-Cilag, the manufacturer of transdermal fentanyl (Durogesic) and Napp Laboratories, the manufacturer of sustained release morphine. EK has been reimbursed by Janssen-Cilag for participation at a meeting sponsored by Janssen-Cilag.”
       10. References
           1. (2) Ashburn MA, Staats PS. Management of chronic pain. Lancet 1999;353:1865-9.
           2. (3) Portenoy RK. Opioid and adjuvant analgesics. In: Mitchell M, ed. Pain 1999—an updated review. Seattle: IASP Press, 1999:3-18.
           3. (4) World Health Organization. Cancer pain relief, 2nd ed. Geneva: WHO, 1996
           4. (5) McQuay H. Opioids in pain management. Lancet 1999;353:2229-32.
              1. “Opioids are our most powerful analgesics, but politics, prejudice, and our continuing ignorance still impede optimum prescribing.” {Direct quote in Allan et al.}
              2. “Drug addicts are not in pain. The political message is that the medical use of opioids does not create drug addicts, and restrictions on this medical use hurt patients.”
           5. (6) Portenoy RK. Opioid therapy for chronic non-malignant pain: a review of critical issues. J Pain Symptom Manage 1996;11:203-17.
           6. (7) Rowbotham MC, Reisner-Keller LA, Fields HL. Both intravenous lidocaine and morphine reduce the pain of postherpetic neuralgia. Neurology 1991;41:1024–8.
           7. (8) Dellemijn PLI, Vanneste JAL. Randomised double-blind active-placebocontrolled crossover trial of intravenous fentanyl in neuropathic pain. Lancet 1997;349:753-8.
           8. (9) Arkinstall W, Sandler A, Goughnour B, Babul N, Harsanyi Z, Drake A. Efficacy of controlled-release codeine in chronic non-malignant pain: a randomized, placebo-controlled clinical trial. Pain 1995;62:169-78.
              1. FUNDED BY PURDUE
           9. (10) Moulin DE, Iezzi A, Amireh R, Sharpe WKJ, Boyd D, Merskey H. Randomised trial of oral morphine for chronic non-cancer pain. Lancet 1996;347:143-7.
              1. FUNDED BY PURDUE
              2. n=46
           10. (11) Watson CPN, Babul N. Efficacy of oxycodone in neuropathic pain: a randomized trial in postherpetic neuralgia. Neurology 1998;50:1837-41.
           11. (12) Schug SA, Merry AF, Acland RH. Treatment principles for the use of opioids in pain of nonmalignant origin. Drugs 1991;42:228-39.
           12. (13) Brown RL, Fleming MF, Patterson JJ. Chronic opioid analgesic therapy for chronic low back pain. J Am Board Fam Pract 1996;9:191-204.
           13. (14) Graziotti PJ, Goucke CR. The use of oral opioids in patients with chronic non-cancer pain. Management strategies. Med J Austr 1997;167:30-4.
           14. (15) Anon. The use of opioids for the treatment of chronic pain: a consensus statement from the American Academy of Pain Medicine and the American Pain Society. Pain Forum 1997;6:77–9.
16. Transdermal Fentanyl as Treatment for Chronic Low Back Pain
    1. [https://www.industrydocuments.ucsf.edu/docs/tsgg0230](https://www.industrydocuments.ucsf.edu/drug/docs/#id=tsgg0230)
    2. Author : Simpson Jr, Richard K; Edmondson, Everton A; Constant, Charles F; Collier, Connie; Journal of Pain and Symptom Management; Baylor College of Medicine; Elsevier; US Cancer Pain Relief Committe
    3. Document Date : 1997 October 04
    4. Type : article; bibliography; publication
    5. ID : tsgg0230 ( TID : hpf71j00 )
    6. ARK : ark:/88122/tsgg0230
    7. Collection : Oklahoma Opioid Litigation Documents; Opioid Documents Collection
    8. Key Points:
       1. Authors
          1. Richard K. Simpson, Jr, MD, PhD
          2. Everton A. Edmondson, MD
          3. Charles F. Constant, MPH, PhD
          4. Connie Collier, RN
          5. Department of Neurosurgery, Baylor College of Medicine, Houston, Texas
       2. Overall less aggressive in promoting opioids for chronic pain; notably, an earlier study than Allan or Milligan
       3. No discussion of addiction or abuse potential
       4. “This study was supported by a grant from the Janssen Pharmaceutica Research Foundation.”
17. Prolonged Treatment with Transdermal Fentanyl in Neuropathic Pain
    1. [https://www.industrydocuments.ucsf.edu/docs/fyfg0230](https://www.industrydocuments.ucsf.edu/drug/docs/#id=fyfg0230)
    2. Author : Dellemijn, Paul LI; Duijn, Hans van; Vanneste, Jan AL
    3. Document Date : 1998 October 04
    4. Type : graph; publication; table
    5. ID : fyfg0230 ( TID : lcf71j00 )
    6. ARK : ark:/88122/fyfg0230
    7. Collection : Oklahoma Opioid Litigation Documents; Opioid Documents Collection
    8. Key Points:
       1. Methods
          1. “Patients
             1. Subjects with noncancer neuropathic pain were recruited from our own outpatient clinic population and through telephone requests and letters to colleagues from Amsterdam and surroundings who were presumed to treat patients with neuropathic pain, such as neurologists, neurosurgeons, and anesthesiologists. All patients who completed a randomized, double-blind, active placebo-controlled trial with intravenous infusions of either FEN and diazepam or FEN and saline17 were invited to be enrolled in this second study assessing the benefits and risks of prolonged treatment with FENtd.”
             2. “Exclusion criteria were use of opioids or modified drug regimens during the 2 weeks before starting the study; contraindications to opioids, such as a history of opioid abuse; presence of multiple sites or other types of pain; intermittent neuropathic pain, such as trigeminal neuralgia; and uncertainty about the neuropathic origin of pain.”
18. Medical Affairs Analgesia Medical Science Liaison Report
    1. [https://www.industrydocuments.ucsf.edu/docs/zxgg0230](https://www.industrydocuments.ucsf.edu/drug/docs/#id=zxgg0230)
    2. Author : Unknown
    3. Document Date : Unknown
    4. Type : report; table
    5. ID : zxgg0230 ( TID : ejf71j00 )
    6. ARK : ark:/88122/zxgg0230
    7. Collection : Oklahoma Opioid Litigation Documents; Opioid Documents Collection
       - 1. Cites Portenoy multiple times

164) Tapentadol team status – 6/24/2009 -- Acute Pain Publications

Document Data

- **Author :** *Unknown*
- **Document Date :** 2009 June 24
- **Type :** report
- **ID :** jlgg0230 ( TID : pkf71j00 )
- **ARK :** ark:/88122/jlgg0230
- **Collection :** Oklahoma Opioid Litigation Documents; Opioid Documents Collection

Document Notes

- UPCOMING TAPENTADOL – **Acute Pain Publications**
  - Causes and Consequences of Under-managed Acute Pain
  - Understanding the Role of Neuroplasticity in Pain Progression
  - Pain pathways and the mechanisms of analgesia
  - Appropriate Pain Management: Matching the right drug and dose to your patient’s pain
- UPCOMING TAPENTADOL – **Chronic Pain Publications**
  - Challenge of managing mixed chronic pain
  - Long-term consequences of chronic pain
  - Opioid abandonment due to side effects: implications for chronic pain management
  - Neuronal pathways of chronic pain transmission and analgesia
  - State of the Art: multi-modal therapy for chronic pain
- UPCOMING TAPENTADOL – **Global Pain Publications**
  - The unique pharmacology of tapentadol: redefining success in pain management
  - Proceedings of expert panel: paid supplement
  - Review of side effects association with analgesic agents
- UPCOMING TAPENTADOL – **Scientific Content – CME!**
  - NeoPathways Website
  - MODULE: Chronic Pain is a Challenge to Manage due to its complex pathophysiology
  - MODULE: chronic pain has signifcant long-term consequences

**GHOST WRITING – Author: Charles Argoff;**

- Outline sent to Dr. Argoff; outline approved by Dr. Argoff; 1^st^ draft sent to author
  - Did he write it???
  - Argoff has received over $600k from pharma (see previous Argoff searches)
-
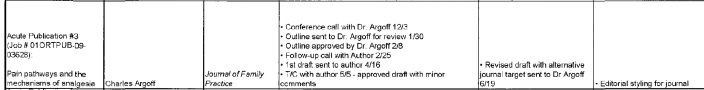


**GHOST WRITING – Author: Dr McCarb**

- Outline approved by Dr. McCarberg
- Current status = 1^st^ draft in development; Next steps = author review of first draft
-
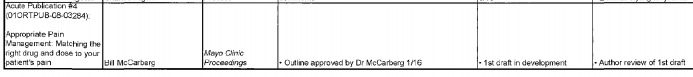


**MANY MORE EXAMPLES OF GHOST WRITING IN THIS DOCUMENT!!!**

220) Speaker Agreement

Document Data

- Author : Brookes, Lynne M; Argoff, Charles
- Document Date : 2006 April 09
- Type : agreement
- ID : gsgg0230 ( TID : uof71j00 )
- ARK : ark:/88122/gsgg0230
- Collection : Oklahoma Opioid Litigation Documents; Opioid Documents Collection

Document Notes

- Speaker agreement between CEPHALON and **CHARLES ARGOFF**
- Cephalon wishes to obtain the services of Healthcare Provider to spttak for Cephalon in areas of medical interest about Cephalon's marketed pharmaceutical products, knd Healthcare Provider wishes to provide such speaking services, all subject to the terms and conditions of this Agreement.
- As compensation for Healthcare Provider's performance of the services to he performed by Healthcare Provider under this Agreement, Cephalon **shall provide honoraria and shall reimburse Healthcare Provider** (when appropriate and in compliance with law) for out-of-pocket travel, hotel, meal and other expenses reasonably incurred in accordance with Cephalon's reimbursement guidelines described in Exhibit A

254) Duragesic Focused and Targeted Execution – 2004 Business Plan

Document Data

- Author : Janssen Pharmaceutica
- Document Date : 2003 August 06
- Type : presentation; slides
- ID : fxgg0230 ( TID : pif71j00 )
- ARK : ark:/88122/fxgg0230
- Collection : Oklahoma Opioid Litigation Documents; Opioid Documents Collection

Document Notes

- Title of Slides = **DURAGESIC; FENTANYL TRANSDERMAL SYSTEM; FOCUSED & TARGETED EXECUTION** (2004 business plan)
- Market Analysis – Growth Drivers
  - Recognized undertreatment of pain
    - Increased legislation
    - **Mandatory CE/CME**
    - Litigation for undertreatment
  - Acceptance of Opioids for non-cancer pain
  - **Consumers more demanding**
  - New & future competitive entries
- Market Analysis – Growth Inhibitors
  - **Perceived “risk” of opioids -- OPIOPHOBIA**
  - **Limited evidence-based scientific data**
  - Increased State/MCO restrictions
- Duragesic statement = **Life, Uninterrupted** and  **Work, Uninterrupted**
  - Duragesic positioning statement – Duragesic significantly improves physical and social functioning by providing the only chronic pain relief that is consistent and effective for 72 hours
- Non-cancer pain is the growth opportunity
- Differentiate DURAGESIC from competition
  - Focus on physician and patient end goal
  - Continue to leverage KOL relationships: Med affairs, NPEC, National Pain Summit
  - Enhance KOL, Pharmacist & key customer relationships within market
- Internal Assessment
  - Lessons Learned: functionality positioning aligns with physician and patients’ end goal of therapy 🡪 Brand messages have evolved to a more patient centric platform
- Internal Assessment: SWOT Analysis & Issue Identification
  - Weakness – Limited clinical data
  - Opportunities – Functionality message ownership
  - Threats – Opioid abuse publicity/ media & potential awareness
- **See “Medical Affairs; 2004 Key Projects” screenshots – potential evidence of seeding trials, ghost writing, general academic malfeasance, CME planning**


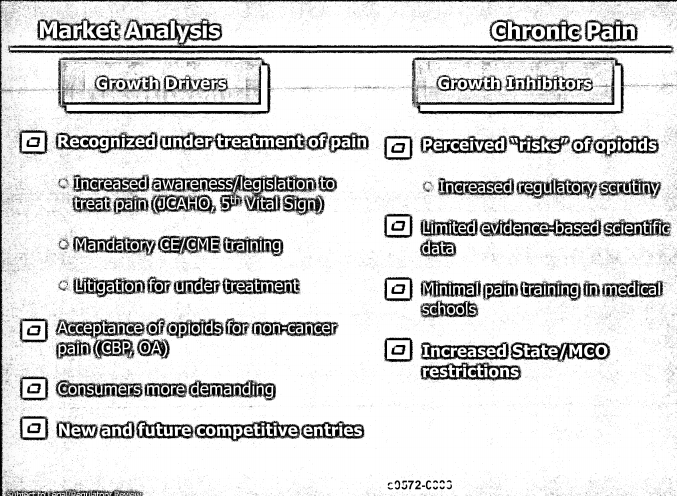


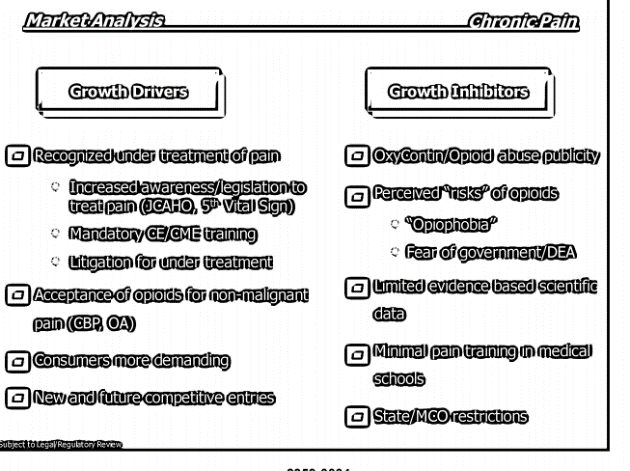


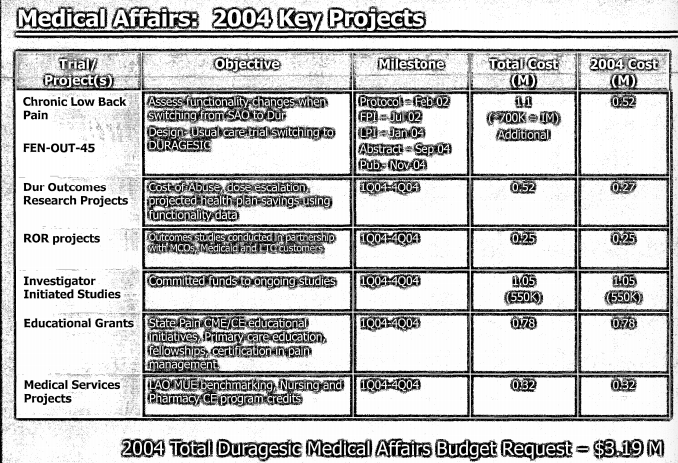


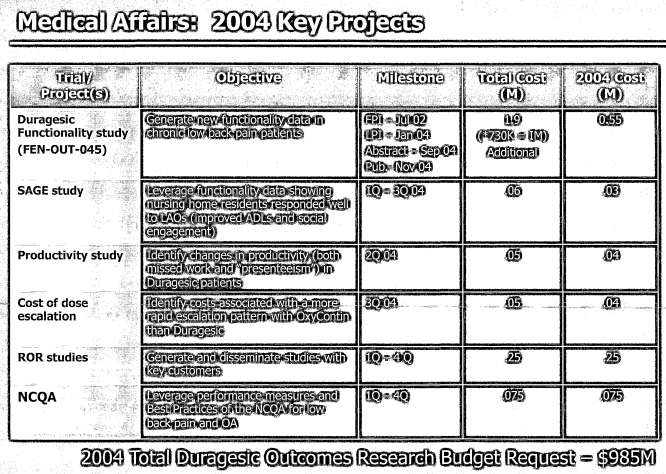


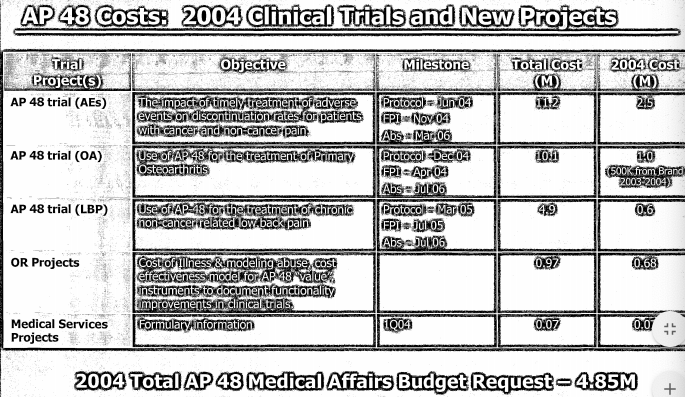


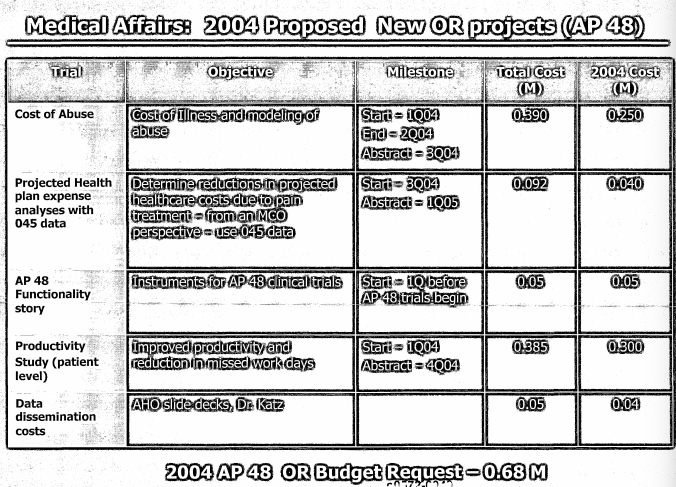


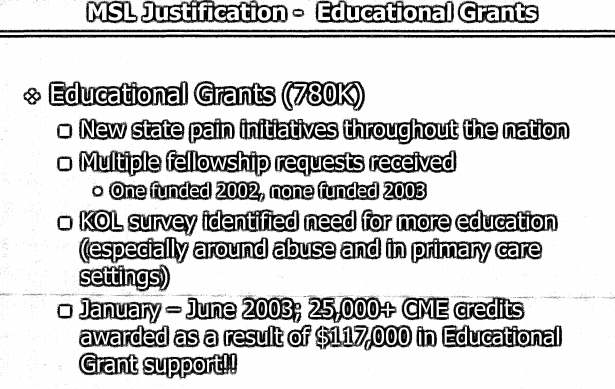


264) Duragesic Fentanyl Transdermal System

Document Data

- Author : Unknown
- Document Date : 2002 July 30
- Type : agenda; chart; graph
- ID : zqgg0230 ( TID : cof71j00 )
- ARK : ark:/88122/zqgg0230
- Collection : Oklahoma Opioid Litigation Documents; Opioid Documents Collection

Document Notes

- TITLE = **Duragesic Fentanyl Transdermal System; 2003 Business Plan** (July 30, 2002)
- Events for opportunities = BUILDING TO A 1$ BILLION BRAND
- MARKET ANALYSIS
  - Growth Drivers
    - Recognize undertreatment of pain
    - Acceptance of opioids for non-malignant pain
    - Consumers more demanding
  - Growth Inhibitors
    - Oxycontin abuse publicity
    - Perceived risk of opioids – opiophobia, fear of government/DEA
    - Limited scientific evidence
- INTERNAL ASSESSMENT
  - Lessons learned
    - **Functionality represents a primary treatment goal and is believable and ownable message**
    - DURAGESIC patients drop off earlier in therapy and to greater degree than on competitive treatments
    - **Loyalty/Retention programs must set proper expectations for patients and provide ongoing support**
- **Strategic Initiative – Enhance success and retention among DURAGESIC patients**
  - GOAL = reduce gap in patient drop-off 50% by year end
- Functionality “Visiting Professor” Preceptorship – to share learnings and educate physicians on key functionality tools and measures to be used in the treatment of chronic pain


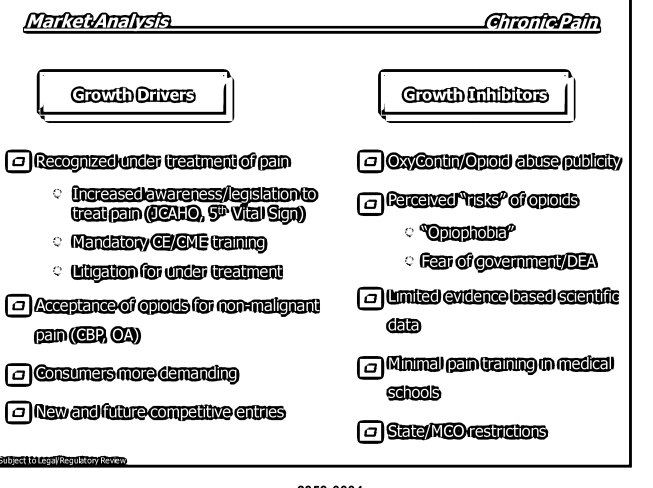


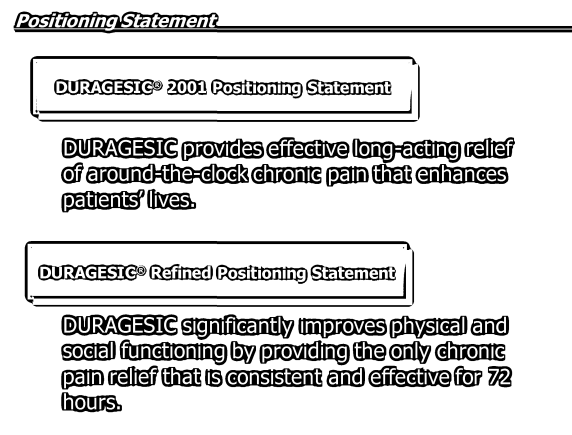


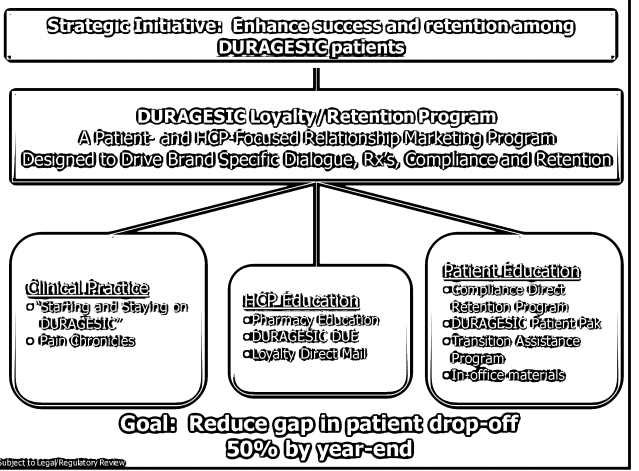


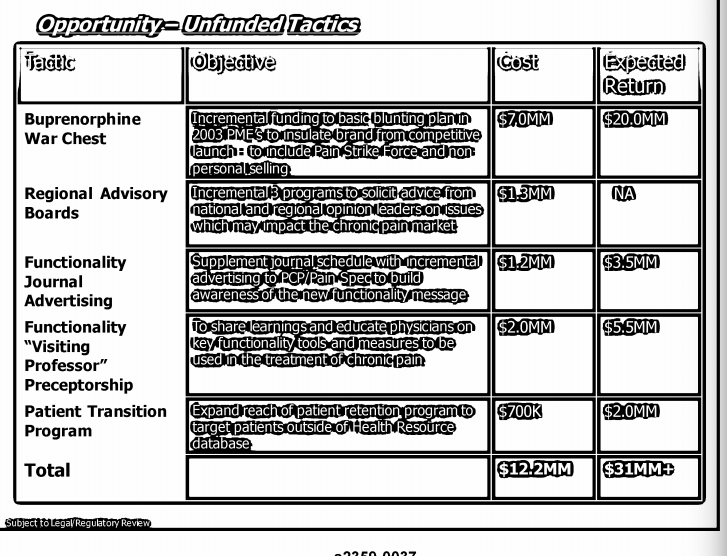


274) Duragesic Net Trade Sales 1995- 2002

Document Data

- Author : Unknown
- Document Date : 2001 October 10
- Type : report
- ID : rqgg0230 ( TID : znf71j00 )
- ARK : ark:/88122/rqgg0230
- Collection : Oklahoma Opioid Litigation Documents; Opioid Documents Collection

Document Notes

- Title = **DURAGESIC 2002 Tactical Plan (October 2001)**
- 2002 Key Strategies
  - Position DURAGESIC as optimal LA opioid choice for chornic non-malignant and malignant pain
  - Redefine “safety profile” of LA opioids to include assessment of abuse potential
  - Increase awareness and RX request from patients
- Key Strategy = **Redefine the Safety Profile of LAOs to include an Assessment of Abuse Potential**
  - Tactic = Message Refinement
    - Objective = understand and develop optimal DURAGESIC promotional and medical education message to key customer segments and assess the potential for leveraging lower abuse rates
    - Description = Multiple phase, iterative market research based assessment
  - Tactic = The Squeeze
    - Objective = Provide assistance and information in alleviating treatment obstacles which currently exist for chronic pain patients and healthcare providers
    - Description = Multiple wave direct mail campaign targeted at providers, policymakers and payors with emphasis on specific geographical issues. **Each wave will address the role of DURAGESIC and ensure that it is a safe and effective alternative**
- Key Strategy = Attain competitive Share of Voice (SOV) among highest decile targets
  - Tactic = Medical Affairs + Publication Plan
    - Objective = Increase the awareness of DURAGESIC effectiveness in treatment of **chronic non-malignant pain**
    - Description = **A comprehensive publication plan including primary and secondary publications, review articles, position papers, brief reports. Primary publications will include data generated by Janssen studies, EMRP, Outcomes research**


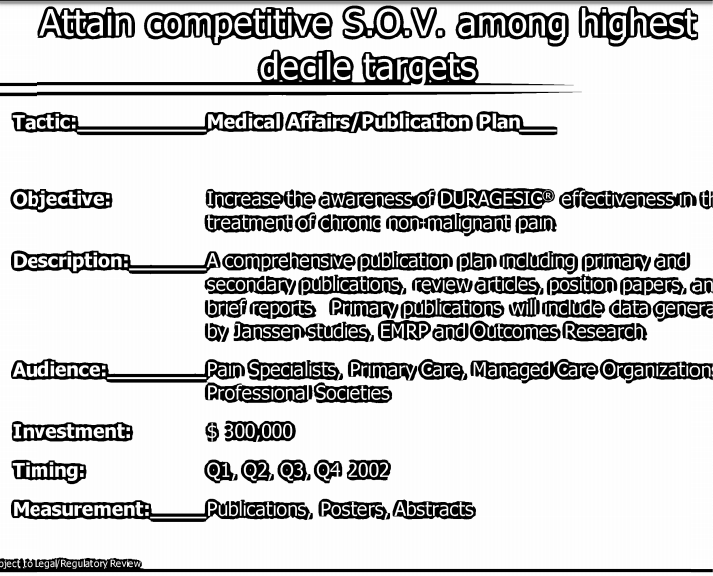


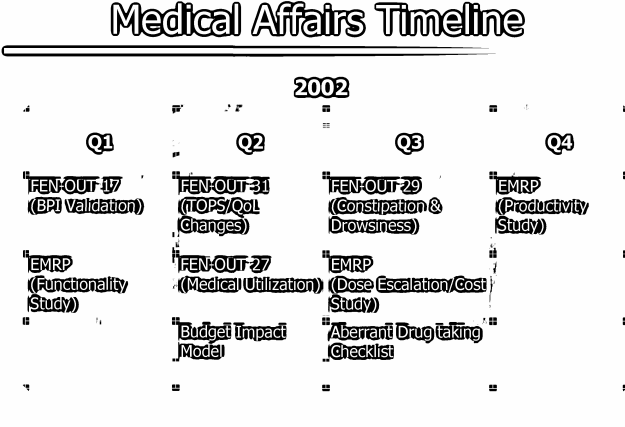


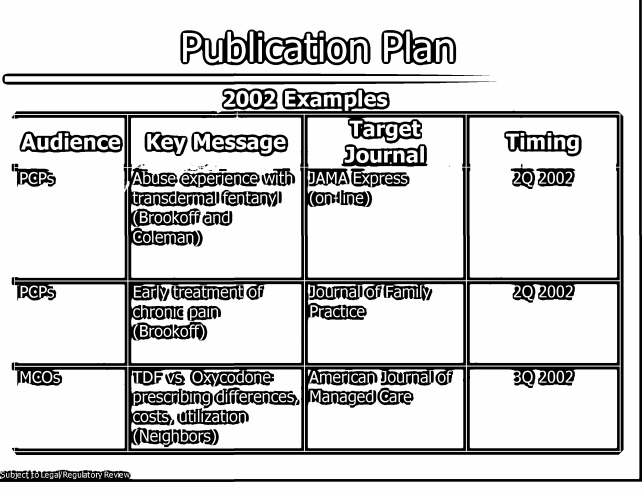


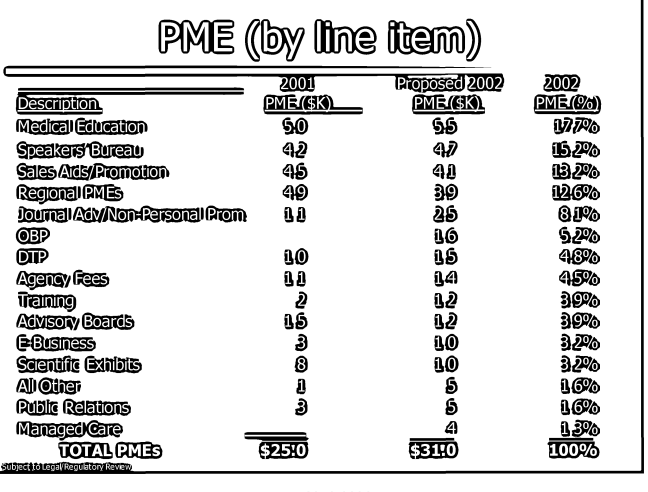


**ENRICHMENT TRIALS**

5) Benefit/Risk Assessment of Opioid Therapy for Chronic Pain Advisory Board Meeting (Nov 2011)

- Document Data
  - **Author :** Intercontinental Chicago O'Hare Hotel; Interactive Forums Inc
  - **Document Date :** 2019 March 13
  - **Type :** proposal; report, scientific
  - **ID :** ppgg0230 ( TID : hnf71j00 )
  - **ARK :** ark:/88122/ppgg0230
  - **Collection :** Oklahoma Opioid Litigation Documents; Opioid Documents Collection
- Document Notes
  - Goal of Meeting: Janssen Scientific Affairs, LLC (Janssen) is interested in evaluating the implications of the recent report of the Institute of Medicine (IOM) Committee on Advancing Pain Research, Care, and Education on Janssen's program of analgesic drug development. The primary goal of this meeting is to extend the work of the IOM Committee and to obtain expert feedback and recommendations on current and future opportunities for clinical research and educational programs aimed at **improving the benefit/risk balance associated with the use of opioid analgesics for chronic pain management**. Janssen intends to use this information to support the clinical and commercial development of Nucynta® ER [tapendatol] and its other analgesic drugs in development.
    - **Identify clinical & educational programs to maximize the benefit and minimize the risk of opioid therapy**
  - Advisors include MDs, PhDs, PharmDs from various institutes
  - Key Findings: **Current Scientific Evidence on Benefit/Risk of Opioid Therapy for Chronic Pain**
    - Cochrane Review --> probably understates opioid abuse risk (due to excluding patients w/ history of substance abuse, excluding patients from populations known to be high risk, etc)
    - Start telling patients that goal is tolerable pain, not pain-free existence
    - Create and optimize tools that physicians can use to predict/manage/minimize opioid abuse in their patients
      - However, there is no single perfect predictor in a patient’s response to opioid
      - “gut feeling” of physicians commonly wrong
    - “One advisor stated that most patients on long-term chronic opioid therapy will present with at least one episode of misuse; Patients who take opioids *periodically* do not usually have issue with misuse”
    - “Advisors were concerned that PCP would not have the time or inclination to effectivelye valuate risk for misuse and abuse”
    - Opioid contract signed by provider & patient has promise
    - “Benefit of opioid therapy for chronic pain are consider modest by most advisors”
    - Before opioids are prescribed, physician should have an exit strategy and discuss with patient
  - Key Findings: **Clinical Research Approaches to Assess Benefits/Risk of Opioid Therapies**
    - “Advisors offered the following comments regarding an effectiveness trial of Nucynta ER to evaluate superiority compared to OxyContin”
    - Cancer Pain Registry = “Cancer centers that have practitioners prescribing Nucynta ER may enroll patients and practitioners in an observational registry program”
      - **Advisors are concerned that registry will be SEEDING TRIAL – encourage use of Nucynta ER b/c Janssen is not providing free drug and patients are being compensated; perception that Nucynta ER will be prescribed in order to receive compensation**
    - Comparative studies to differentiate Nucynta ER from long-acting opioids
      - Advisors suggest designing clinical trial to be similar to previous succesful trials – FREEDOM (pregabalin) or STAR*D (Citalopram)
  - Key Finding: **Programs to Maximize the Benefit and Minimize the Risk of Opioid Therapy**
    - Brainstorming ways to minimze risk of opioid abuse
      - TV ads
      - School interventions
      - Lock boxes for patients
  - Key Finding: “Comparative studies to help differentiate Nucynta ER from other long-acting opioids.
    - Recommendation: Consider a six-month, enriched enrollment, randomized withdrawal study, similar to the Fibromyalgia Relapse Evaluation and Efficacy for Durability of Meaningful Relief (FREEDOM) trial that evaluated pregabalin for fibromyalgia pain.
    - Recommendation: Consider designing a clinical trial similar to the Sequenced Treatment Alternatives to Relieve Depression (STAR*D) study.
    - For Nucynta ER trial, consider the following methodology:
      - Step 1: Patients are initiated on duloxetine (Cymbalta®) for pain.
      - Step 2: If pain is not adequately controlled, tramadol, Nucynta or placebo would be added.
      - Step 3: If pain is not adequately controlled, patient may be changed to a different opioid.
      - Recommendation: Consider conducting a trial with a heterogeneous or enriched group of patients with depression.”
  - Key Finding: “Comparative studies help differentiate Nucynta ER from other long-acting opioids.
    - Consider a six-month, enriched enrollment, randomized, withdrawal study similar to the Fibromyalgia Relapse Evaluation and Efficacy for Durability of Meaningful Relief (FREEDOM) trial that evaluated pregabalin for fibromyalgia pain.
    - Limit enrollment to patients with chronic musculoskeletal pain such as osteoarthritis and lower back pain.
    - Consider a trial in patients with pain symptoms of diabetic neuropathy.”
    - “Consider conducting a trial with a heterogeneous or enriched group of patients with depression.”

72) Powerful chronic and acute pain management – Nucynta ER

Document Data

- **Author :** Janssen Pharmaceutical, Inc
- **Document Date :** 2013 May
- **Type :** presentation; slides; report
- **ID :** pnfg0230 ( TID : fcf71j00 )
- **ARK :** ark:/88122/pnfg0230
- **Collection :** Oklahoma Opioid Litigation Documents; Opioid Documents Collection

Document Notes

- Internal powerpoint about Nucynta from Janssen
- **ENHANCED ENROLLMENT** (in the slide its called “enriched design”)
  -
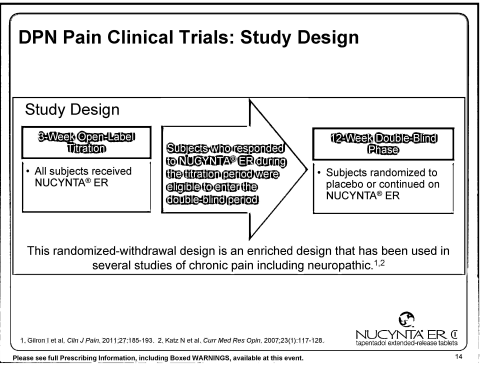

- The rest of the PowerPoint is just the PPI on slide
- Declaration of Russell K Portenoy, MD in State of Oklahoma v Purdue Pharma LP, et al
  - [https://www.industrydocuments.ucsf.edu/docs/nshg0230](https://www.industrydocuments.ucsf.edu/drug/docs/#id=nshg0230)
  - Author : District Court of Cleveland County, State of Oklahoma
  - Document Date : 2010 January 17
  - Type : legal; affidavit
  - ID : nshg0230 ( TID : xyf71j00 )
  - ARK : ark:/88122/nshg0230
  - Collection : Oklahoma Opioid Litigation Documents; Opioid Documents Collection
  - Key points:
    - “I have agreed to cooperate with certain plaintiffs who have entered into settlement agreements with me dismissing me as a defendant in their cases (‘Settling Plaintiffs’). Settling Plaintiffs agreed to dismiss me from their cases in exchange for my truthful cooperation. The proffer agreement with those Plaintiffs can be voided and the original lawsuits may be reinstated against me if my statements are recklessly and materially not truthful or accurate.”
    - “This declaration includes statements describing how my views about opioid therapy and its marketing by the pharmaceutical companies have changed during the period between the mid-1980s and the first decade of the 2000s.”
    - “Prior to, and then during the 1980s, opioids were disfavored for use in chronic, noncancer pain because of concerns that patients using opioids would develop tolerance and physical dependence, and be at risk for abuse, misuse, addiction, and diversion.^1^”
      - Portenoy RK, Foley KM. Chronic use of opioid analgesics in non-malignant pain: report of 38 cases. Pain. 1986;25:171-86.
    - Portenoy RK, Foley KM. Chronic use of opioid analgesics in non-malignant pain: report of 38 cases. Pain. 1986;25:171-86.
      - “This article was a retrospective case series describing anecdotal information about a highly selected group of patients. I recall that Dr. Foley and I wanted to write the paper to describe a phenomenon that we believed was under-appreciated by the medical community—the possibility of long-term pain relief from opioid therapy, without the development of tolerance leading to treatment failure and without the development of serious adverse effects, including drug abuse. We also wanted to use this description of cases as a starting point for a broad discussion of the clinical issues relevant to the appropriate use of these drugs. Our initial contribution to this discussion appeared in the Discussion section of the paper, which ended with our recommendation that opioid therapy be considered only after "all reasonable attempts at pain control have failed and persistent pain is the major impediment to improved function."^3^ Contrary to how some drug companies later used this article, it was never intended as a report of high-quality evidence, or as support for broad adoption of opioid therapy; it was a description of anecdotal information accompanied by a brief narrative review of the literature, and was intended to suggest that the role of long-term opioid therapy needed re-thinking, and more research, and that clinicians should not consider the approach to be contraindicated, but rather, worthy of consideration in the context of treatment refractory pain.”
    - “In my 1994 chapter, for example, I described a phenomenon that I then called *therapeutic dependence* and a phenomenon that had been labeled as *pseudoaddiction* in a 1989 article by Weissman and Haddox.^6^”
      - Weissman DE, Haddox JD, Opioid pseudoaddiction. Pain 1989;36(3):363-366.
    - “In all my writings, I acknowledged that the disease of addiction was a risk when opioids were used therapeutically. I stated in the 1994 chapter, as I did in many other writings during the 1980s and 1990s, that the disease of addiction would be "very unlikely" to develop when patients with chronic pain and no prior history of substance abuse were prescribed opioids and closely monitored. Although the evidence in support of this statement was scant in terms of the populations with chronic non-cancer pain, it was supported by large surveys of cancer patients receiving opioids for cancer pain. My conclusion was based on an analysis of the clinical literature at the time, which I acknowledged was very limited.”
    - “In 1996, a workgroup was formed by the American Academy of Pain Medicine and the American Pain Society for the purpose of creating a *Consensus Statement on the Use of Opioids for the Treatment of Chronic Pain*. The committee chair was Dr. J. David Haddox, who was hired by Purdue Pharma in 1999. I was not involved in the deliberations of the workgroup, but I recall that I was sent a draft of the consensus statement for my comments before it was presented to the respective organizations for approval and dissemination, I do not recall whether I made suggestions for modifications.”
    - {From p.15, a lengthy description of the nature of financial relationships with Defendants}
    - “I recall that some of these offers to write review articles, which I did not pursue, included the help of a medical writer for assistance in drafting the paper.”
    - “Based on the interactions that I have had with medical education vendors, I believe that academicians who are provided with honoraria for producing or editing material must be vigilant to avoid messages that are not well supported or prudent, and are in the interest of the drug company, without a corresponding medical benefit for the patient. Although I cannot cite specific cases, my experience suggests that some of the work ostensibly created by academicians through interaction with medical education vendors reflects the work or the influence of drug companies.”
    - “This type of controlled clinical trial provides what is considered to be highquality evidence. This evidence is needed by clinicians to confirm that the drugs they select can be efficacious when they are treating patients. However, these studies recruit patients based on strict inclusion criteria, and consequently, the results may or may not be immediately transferable to practice. In studies of opioids, the patients who are recruited are carefully selected and may not be representative of the overall population with pain; moreover, the duration of study is short compared to the patients treated in practice, and study patients are carefully monitored while they are receiving the study drugs. Clinical practice needs randomized controlled studies to provide an evidence base for patient care, but clinical guidelines are not created solely from the data acquired in these studies. It is my belief that this understanding of the role played by high-quality randomized clinical trials, i.e., necessary to establish the potential for efficacy but insufficient for clinical guidelines that must consider a patient population not represented in the study and patterns of drug use that are not specifically tested, is widely accepted by clinicians and investigators, and would be considered common knowledge in the pharmaceutical industry.”
    - “Addiction can exist while pain is experienced.”
    - “I believe that, over the years, some Defendant drug companies have used my work to promote opioids by referencing the positive statements that 1 made repeatedly without providing the background, analysis of the literature, and cautions that accompanied these positive statements.”
    - “It indicates that the risk of addiction is <1%, although this is an inaccurate interpretation of data (paragraph 40)…”
    - “Drug companies are a major source of research funding and have the ability to influence study proposals. In my opinion, it is clear that drug company research grants provided to academicians for studies of approved drugs generally fund studies that aim to identify or confirm benefits that would be helpful in marketing. Similarly, 1 believe that the drug companies distribute honoraria, fees and grants in a way that elevates specific messages, and messengers, that agree with their preferred messaging. Although I personally was never influenced to say things I did not believe, it is true that the drug companies provided me with many opportunities to express my views, and they used the positive statements that I made about opioids to portray opioid treatment as safe and effective without the accompanying discussion of risk that I included in the papers, chapters, and lectures I produced beginning in the 1980s.”
    - “My work was intended to disabuse clinicians of a bias against opioids by describing the literature as it existed then and the favorable outcomes that I and others were seeing in varied subgroups of patients, and by providing guidelines for treatment that included careful patient selection and vigilant monitoring of drug-related outcomes over time. I believe that the drag companies created material that narrowly focused on the potential for safe and effective treatment of chronic noncancer pain, some of which was attributed to my work, but failed to include an adequate and balanced discussion of the limitations in the relevant science and the risks as they were then known.”
    - “The practice of "enriched enrollment," which is a type of clinical trial design often used in pivotal trials supporting FDA approval of an opioid, exemplifies how these explanatory trials do not provide the type of information about risk that is needed in clinical practice. In these studies, there is an initial open-label phase to select patients who respond to a drug; this is then followed by a double-blind phase to determine whether these responders continue to have benefit from the drug when their effects are compared with a placebo treatment. Patients with addiction risk are typically screened out of the open-label phase, and patients who do not do well during the open-label phase are also dropped from the double-blind phase, Enriched enrollment studies are therefore designed only to see if a drug has a primary outcome, like analgesia, that exceeds placebo in a selected group of favored patients. It is not designed to assess outcomes in the heterogeneous population of patients seeking care in clinical practice, nor is it designed to assess long-term risks. Although drag companies should include documentation of evidence from explanatory trials in their labels, they should be cautious in pointing to these studies as proof of a drug's safety in real world conditions,17”
- Evaluation of Long-Term Efficacy and Safety of Transdermal Fentanyl in the Treatment of Chronic Non cancer Pain
  - [https://www.industrydocuments.ucsf.edu/docs/rsgg0230](https://www.industrydocuments.ucsf.edu/drug/docs/#id=rsgg0230)
  - Author : Milligan, Keith; Lanteri-Minet, Michael; Borchert, Klaus; Helmers, Henk; Donald, Royden
  - Document Date : Unknown
  - Type : article; graph; report, scientific
  - ID : rsgg0230 ( TID : fpf71j00 )
  - ARK : ark:/88122/rsgg0230
  - Collection : Oklahoma Opioid Litigation Documents; Opioid Documents Collection
  - Key points:
    1. International, multicenter, open-label trial
    2. Objective: “assess the efficacy and safety of up to 12 months of therapy with transdermal therapeutic system (TTS) fentanyl in patients (n=532) with chronic noncancer pain.”
    3. Main outcome measures: “pain control assessment, global treatment satisfaction, patient preference for TTS fentanyl, and quality of life.”
    4. Authors:
       1. Keith Milligan – Anaesthetic Department, South Cleveland Hospital, Middlesbrough, UK
       2. Michel Lanteri-Minet – Department of Pain Treatment, Hôpital Pasteur, Nice, France
       3. Klaus Borchert – Anästhesiologische Praxis, Greifswald, Germany
       4. Henk Helmers – Department of Anaesthesiology, Eemland Hospital, Amersfoort, The Netherlands
       5. Royden Donald – Strand Private Hospital, Cape Town, South Africa
       6. Hans-Georg Kress – Universitätsklinik für Allgemeine Anästhesie und Intensivmedizin, Abt. B, Wien, Austria
       7. Hugo Adriaensen – Universitair Ziekenhuis Antwerpen, Edegem, Belgium
       8. Dwight Moulin – Neurology Department, Victoria Hospital, London, Canada
       9. Voitto Järvimäki – Pain Clinic, Oulu University, Central Hospital, Oulu, Finland
       10. Ludo Haazen – Janssen Research Foundation, Beerse, Belgium
    5. “Supported by a grant from the Janssen Research Foundation, Belgium. K. M. has been reimbursed by Janssen-Cilag, the manufacturers of transdermal fentanyl, for attendance at a congress. L. H. was employed by the Janssen research Foundation when this study was performed.”
    6. Introduction
       1. “Evidence suggests, however, that pain is frequently undertreated even for these indications {acute pain and cancer pain}.^2,3^”
       2. “Persistent, albeit largely unfounded, fears about the risks of addiction, toxicity, physical dependence, and tolerance have led to the rejection of opioid analgesia for chronic pain resulting from noncancer disease.^4^”
       3. “Although few prospective clinical studies of opioid use in noncancer pain have been reported, evidence from surveys suggests that long-term opioid therapy can be used successfully in some patients with chronic nonmalignant pain without causing undue adverse events (AEs),^4-6^ a conclusion supported by several recent short-term, randomized controlled trials.^7-9^”
       4. “Reflecting this evidence and changing attitudes toward the therapeutic goals of treating noncancer pain, guidelines for the use of opioids in this setting have recently been proposed.^10-11^ These advocate scheduled around-the-clock drug administration and the use of long-acting preparations or sustained-release formulations to maintain therapeutic serum concentrations.”
    7. Materials and Methods
       1. “Exclusion criteria included a history of allergy or hypersensitivity to opioids; life-threatening disease; skin disease precluding the use of the transdermal system; reduced level of consciousness or inability to give informed consent; pregnancy, lactation, or possibility of pregnancy; social isolation; concomitant psychiatric disorders (depression excluded); history of substance abuse (assessed from the patient's clinical history and communication with the primary care practitioner); history of clinically relevant cardiac, nervous system, or respiratory disease or participation in any clinical trial, except the Allan study,^18^ within the preceding 30 days.”
    8. Statistical Analyses
       1. “The sample size of 500 patients was based on an estimated 2% incidence of AEs to provide a narrow and precise 95% confidence interval of 0.8% to 3.22%.”
    9. Results
       1. “A total of 532 patients were recruited, of whom 103 had participated in the study by Allan et al^18^;”
       2. “Of the 532 patients recruited, 301 (57%) patients completed the trail and 231 (43%) patients discontinued treatment prematurely.”
       3. “A total of 130 (25%) of the safety analysis population (n=530) discontinued TDF as a result of AEs.”
       4. “The most commonly used opioids immediately before study entry were morphine (48%) and fentanyl (28%, including patients from the Allan study^18^).”
       5. “A total of 375 of 421 (89%) patients stated a treatment preference (patients participating in the Allan study^18^ and those already on TTS fentanyl were not asked to express a preference).”
    10. Discussion
        1. “Stability of pain control during the 12-month period was achieved at the cost of an increase in mean TDF dosage from 48 to 90 μg/h. The largest increase occurred during the first months of treatment, as the patients titrated the dose upward themselves, and then stabilized during prolonged treatment. This increase may be due to the individual or combined effects of using conservative conversion table, underdosing at baseline, and the development of tolerance.”
        2. “Unlimited access to rescue medication, which is not always standard clinical practice, could have led to patients titrating the dose themselves to best effect, and consequently a subsequent increase in TDF dose when daily consumption of rescue morphine reached 60 to 90 mg. The average daily consumption of rescue medication was a reflection of increased pain intensity because it was probably related to an increased or altered physical function and subsequent pain at varying times.^10^ Experience with opioids has shown that tolerance to their analgesic effects is uncommon in patients with chronic pain.^10,23^ Many surveys and supportive clinical experience regarding the long-term use of opioids in patients with non-cancer pain have shown the development of tolerance not to be a clinical problem.^6,24^”
        3. “Opioid withdrawal symptoms were only reported in 3% of patients, suggesting that prolonged treatment is not associated with increased risk of withdrawal syndromes. There were no reports of addictive behavior in any of the patients during this long-term study. Because the fear of addiction is one of the reasons for the underuse of opioids in chronic noncancer pain, this study provides further evidence that these fears are unfounded. Our results also confirm that there is no basis for concern about persistent opioid side effects and long-term toxicity.^6^”
    11. Acknowledgments

“We thank all of the other investigators who participated in the trial: **J. Mayaert, L. Plaghki** (Belgium); **J. Clark,** H. Hays, **A. Mailis,** **M. Ong-Lam, D. Reid, P. Watson** (Canada); **E. Kalso** (Finland); N. Attal, F. Boreau, L. Brasseur, J. Bruxelle, P. Ginies, E. Lajous, S. Perrot, A. Serrie (France); **L. Allan**, **R. Atkinson**, G. Batchelor, **F. Campbell**, G. Carmichael, B. Collett, H. Dunckley, M. Fallon, I. Finlay, W. Fitch, **R. Gautam**, **M. Hanna**, D. Laird, D. Littlewood, W. Loan, K. Markham, L. Morrison, **W. Notcutt**, K. Rogers, P. Selby (UK); A. Beyer, W. Dinter, L. Petracic (Germany); and **G. Braak,** P. Brouwer, **G. van Oss, W. Zuurmond** (The Netherlands). We also thank the following contributors : At Janssen Research Foundation: L. Haazen was responsible for international trial supervision and coordination and was involved in the writing of the article (he will act as guarantor); **D. Peelmans**, F. Spensieri, **N. Currie, M. Jarvinen**, B. Flaisler, I. Broudic, A. Bessems, A. Schmidt-Mertens , D. Tseneklidou-Stoeter, **M. Travers**, D. Mathewson, D. Coakes, **M. Uitendaal**, W. Schipper, **P. Matthysen** (trial coordination and monitoring); and H. Swinnen, E. Everaert, I. van den Broeck, M. Nijs, **L. Bijnens**, H. Joosen (clinical data processing).”

- - - 1. No Janssen names returned results in DIDA
    1. References

1. Cleeland CS: Undertreatment of cancer pain in elderly patients. JAMA 279:1914-5, 1998
2. Zenz M, Zenz T, Tryba M, Strumpf M; Severe undertreatment of cancer pain: A 3-year survey of the German situation. J Pain Symptom Manage 10:187-91, 1995
3. Portenoy RK: Chronic opioid therapy in nonmalignant pain. J Pain Symptom Manage 5(suppl):546-62, 1990
4. France RD, Urban BJ, Keefe FJ: Long-term use of narcotic analgesics in chronic pain. Soc Sci Med 19:1379-82, 1984
   1. n=16
5. Portenoy RK: Opioid therapy for chronic nonmalignant pain: A review of the critical issues. J Pain Symptom Manage 11:203-17, 1996
6. Arkinstall W, Sandler A, Goughnour B, Babul N, Harsanyi Z, Drake A: Efficacy of controlled-release codeine in chronic non-cancer pain: A randomized, placebo-controlled trial. Pain 62:169-78, 1995
   1. FUNDED BY PURDUE
7. Jadad A, Carroll D, Glynn C, Moore R, McQuay H: Morphine responsiveness of chronic pain: Double-blind randomised crossover study with patient-controlled analgesia. Lancet 339:1367-71, 1992
   1. n=10
8. Moulin D, lezzi A, Amireh R, Sharpe W, Boyd D, Merskey H: Randomised trial of oral morphine for chronic non-cancer pain. Lancet 347:143-7, 1996
   1. FUNDED BY PURDUE
   2. n=46
9. Portenoy RK: Opioid therapy for chronic nonmalignant pain: Current status, in Fields HL, Liebeskind JC (eds): Progress in Pain Research and Management; Pharmacologic Approaches in the Treatment of Chronic Pain: New Concepts and Critical Issues. Seattle, WA, IASP Press, 1994
10. Schug S, Merry A, Acland R: Treatment principles for the use of opioids in pain of non-malignant origins. Drugs 42:228-39, 1991
    1. Behind paywall
11. Allan L, Hays H, Jensen N H, de Waroux BLP, Bolt M, Donald R, Kalso E: Randomised crossover trial of transdermal fentanyl and sustained release oral morphine for treating chronic non-cancer pain. BMJ 322:1154-1158, 2001
12. Coyle N, Adelhardt J, Foley KM, Portenoy RK: Character of terminal illness in the advanced cancer patient: Pain and other symptoms in the last 4 weeks of life. J Pain Symptom Manage 5:83-93,1990
13. Collett B-J: Opioid tolerance: The clinical perspective. Br J Anaesth 81:58-68, 1998
14. Galer BS, Coyle N, Pasternak GW, Portenoy RK: Individual variability in response to different opioids: Report of five cases. Pain 49:87-91, 1992
15. Randomised Crossover Trial of Transdermal Fentanyl and Sustained Release Oral Morphine for Treating Chronic Non-Cancer Pain
    1. [https://www.industrydocuments.ucsf.edu/docs/qsgg0230](https://www.industrydocuments.ucsf.edu/drug/docs/#id=qsgg0230)
    2. Author : Allan, Laurie; Hays, Helen; Jensen, Niels-Henrik; de Waroux, Bernard Le Polain; Bolt, Michiel; Kalso, Eija; Donald, Royden
    3. Document Date : 2001 May 12
    4. Type : publication; article
    5. ID : qsgg0230 ( TID : epf71j00 )
    6. ARK : ark:/88122/qsgg0230
    7. Collection : Oklahoma Opioid Litigation Documents; Opioid Documents Collection
    8. Key Points:
       1. Authors
          1. Laurie Allan – (director) Chronic Pain Services, Northwick Park and St. Mark’s NHS Trust, Harrow, Middlesex HA1 3UJ
          2. Helen Hays – (associate clinical professor) Department of Family Medicine, University of Alberta, Edmonton, Alberta, Canada T6G 2C8
          3. Niels-Henrik Jensen – (head of department) Multidisciplinary Pain Centre, Department of Anaesthesiology, Herlev University Hospital, DK-2730, Denmark
          4. Bernard Le Polain de Waroux – (staff anaesthesiologist) Clíniques Universitaires St-Luc, 1200 Brussels, Belgium
          5. Michiel Bolt – (anaesthesiologist) Alg Ziekenhuis Eemland De Lichtenberg, 3818 ES Amersfoort, Netherlands
          6. Royden Donald – (specialist anaesthetist) Strand Private Hospital, Cape Town 7139, South Africa
          7. Eija Kalso – (head) Helsinki University Central Hospital Pain Clinic, 00290 Helsinki, Finland
       2. “Objectives: To compare patients’ preference for transdermal fentanyl or sustained release oral morphine, their level of pain control, and their quality of life after treatment.”
       3. “Design: Randomised, multicenter, international, open label, crossover trial.”
       4. “Main outcome measures: Patients’ preference for transdermal fentanyl or sustained release oral morphine, pain control, quality of life, and safety assessments.”
       5. Introduction
          1. “Pain is often undertreated or mistreated, with patients going from doctor to doctor for relief and finally moving outside mainstream medicine in increasing numbers.^2”^
          2. “Opioids are the mainstay of management of cancer pain, providing effective pain relief.^3 4^ Opioids are the most powerful analgesics, but politics, prejudice, and continuing ignorance still impede optimum prescribing.^5^ A review of retrospective and survey data confirms the efficacy of opioids in the treatment of chronic non-cancer pain and found that fears of addiction were not justified.^6^ Randomised controlled trials of intravenous opioids in chronic non-cancer pain show benefit over placebo for morphine and fentanyl, whereas oral placebo controlled trials show efficacy for codeine, morphine, and oxycodone.^7–11^ Worldwide, the value of opioids in this role has led to the development of management guidelines, with recommendations from national organisations.^12–15^”
       6. Participants and methods
          1. “At crossover, patients received the same opioid dose as before the study.”
          2. “Patients were prescribed immediate release morphine (initially 5 mg) every four hours as needed. Patients requiring more than 60 mg of this rescue drug over two days of a three day period with fentanyl could increase their fentanyl dose. Patients receiving morphine needing more than two doses of the rescue drug per day could titrate to a higher dose of morphine.”
          3. “Details of all adverse events and presumed relation to the drugs were noted by the investigator.”
       7. Results
          1. “Sixty patients withdrew; 37 because of adverse events, five because of insufficient efficacy, and 18 for other reasons. Five patients without baseline data were excluded from the efficacy analysis. All patients were included in the safety analysis.”
          2. “The mean starting dose of transdermal fentanyl was 39.7 ìg/hr (range 25-200 ìg/hr) and of sustained release oral morphine 123.0 mg/24 hr (range 10-700 mg/24 hr). The mean dose of fentanyl at the end of the study was 57.3 ìg/hr (range 0-325 ìg/hr) and of morphine 133.1 mg/24 hrs (range 0-800 mg/24 hrs).”
          3. “The predominant reason given for preferring fentanyl was better pain relief, followed by greater convenience and fewer adverse events (table 3).” “Patients treated with transdermal fentanyl had on average lower pain intensity scores than those treated with sustained release oral morphine (mean 57.8, range 33.1-82.5 v mean 62.9, range 41.2-84.6; P < 0.001), irrespective of the order of treatment.” “Analysis of the consumption of rescue drug during the last three weeks of each treatment period showed that the mean (standard deviation) consumption was significantly higher with fentanyl (29.4 (33.0) mg) than with morphine (23.6 (32.0) mg; P < 0.001). A significant (P < 0.05) period effect was also observed: the higher consumption during fentanyl treatment was more apparent in the second trial period (mean 32.4 (SD 38.5) mg) than the first (26.3 (26.0) mg), where the consumption of the rescue drug remained essentially the same over the two treatment periods in the morphine group (23.7 (35.3) mg v 23.6 (27.3) mg).”
       8. Discussion
          1. No discussion of abuse potential or addiction throughout
       9. Acknowledgments
          1. “We thank all the investigators who participated in the trial: **J Maeyaert, L Plaghki** (Belgium); **J Clark**, A Mailis, **D Moulin**, **M Ong-Lam**, **D Reid, P Watson** (Canada); S Andersen, C Christiansen, S Clemensen, K Glahn, T Jonsson, S Larsen, F Molke Borgbjerg, A Schou Olesen, J Mølgaard (Denmark); V Järvimäki, T Heiskanen (Finland); **R Atkinson**, P Brown, **F Campbell**, **R Gautam, M Hanna**, D Hughes, C Knight, **W Notcutt** (United Kingdom); **G Braak**, J Helmers, **G Van Oss, W Zuurmond** (Netherlands); D Lines (South Africa). Contributors: LA was the principal author of the paper. H Noorduin (international supervisor of the trial), **L Bijnens** (biostatistics), **L Haazen, M Travers, D Peelmans, N Currie,** A Jepsen, **M Jarvinen, M Uitendaal, P Matthysen** (local trial coordination and monitoring). MT will act as guarantor for the paper. Funding: The study was supported by a grant from Janssen Research Foundation, Belgium. Competing interests: LA receives support from both Janssen-Cilag, the manufacturer of transdermal fentanyl (Durogesic) and Napp Laboratories, the manufacturer of sustained release morphine. EK has been reimbursed by Janssen-Cilag for participation at a meeting sponsored by Janssen-Cilag.”
       10. References
           1. (2) Ashburn MA, Staats PS. Management of chronic pain. Lancet 1999;353:1865-9.
           2. (3) Portenoy RK. Opioid and adjuvant analgesics. In: Mitchell M, ed. Pain 1999—an updated review. Seattle: IASP Press, 1999:3-18.
           3. (4) World Health Organization. Cancer pain relief, 2nd ed. Geneva: WHO, 1996
           4. (5) McQuay H. Opioids in pain management. Lancet 1999;353:2229-32.
              1. “Opioids are our most powerful analgesics, but politics, prejudice, and our continuing ignorance still impede optimum prescribing.” {Direct quote in Allan et al.}
              2. “Drug addicts are not in pain. The political message is that the medical use of opioids does not create drug addicts, and restrictions on this medical use hurt patients.”
           5. (6) Portenoy RK. Opioid therapy for chronic non-malignant pain: a review of critical issues. J Pain Symptom Manage 1996;11:203-17.
           6. (7) Rowbotham MC, Reisner-Keller LA, Fields HL. Both intravenous lidocaine and morphine reduce the pain of postherpetic neuralgia. Neurology 1991;41:1024–8.
           7. (8) Dellemijn PLI, Vanneste JAL. Randomised double-blind active-placebocontrolled crossover trial of intravenous fentanyl in neuropathic pain. Lancet 1997;349:753-8.
           8. (9) Arkinstall W, Sandler A, Goughnour B, Babul N, Harsanyi Z, Drake A. Efficacy of controlled-release codeine in chronic non-malignant pain: a randomized, placebo-controlled clinical trial. Pain 1995;62:169-78.
              1. FUNDED BY PURDUE
           9. (10) Moulin DE, Iezzi A, Amireh R, Sharpe WKJ, Boyd D, Merskey H. Randomised trial of oral morphine for chronic non-cancer pain. Lancet 1996;347:143-7.
              1. FUNDED BY PURDUE
              2. n=46
           10. (11) Watson CPN, Babul N. Efficacy of oxycodone in neuropathic pain: a randomized trial in postherpetic neuralgia. Neurology 1998;50:1837-41.
           11. (12) Schug SA, Merry AF, Acland RH. Treatment principles for the use of opioids in pain of nonmalignant origin. Drugs 1991;42:228-39.
           12. (13) Brown RL, Fleming MF, Patterson JJ. Chronic opioid analgesic therapy for chronic low back pain. J Am Board Fam Pract 1996;9:191-204.
           13. (14) Graziotti PJ, Goucke CR. The use of oral opioids in patients with chronic non-cancer pain. Management strategies. Med J Austr 1997;167:30-4.
           14. (15) Anon. The use of opioids for the treatment of chronic pain: a consensus statement from the American Academy of Pain Medicine and the American Pain Society. Pain Forum 1997;6:77–9.
16. Transdermal Fentanyl as Treatment for Chronic Low Back Pain
    1. [https://www.industrydocuments.ucsf.edu/docs/tsgg0230](https://www.industrydocuments.ucsf.edu/drug/docs/#id=tsgg0230)
    2. Author : Simpson Jr, Richard K; Edmondson, Everton A; Constant, Charles F; Collier, Connie; Journal of Pain and Symptom Management; Baylor College of Medicine; Elsevier; US Cancer Pain Relief Committe
    3. Document Date : 1997 October 04
    4. Type : article; bibliography; publication
    5. ID : tsgg0230 ( TID : hpf71j00 )
    6. ARK : ark:/88122/tsgg0230
    7. Collection : Oklahoma Opioid Litigation Documents; Opioid Documents Collection
    8. Key Points:
       1. Authors
          1. Richard K. Simpson, Jr, MD, PhD
          2. Everton A. Edmondson, MD
          3. Charles F. Constant, MPH, PhD
          4. Connie Collier, RN
          5. Department of Neurosurgery, Baylor College of Medicine, Houston, Texas
       2. Overall less aggressive in promoting opioids for chronic pain; notably, an earlier study than Allan or Milligan
       3. No discussion of addiction or abuse potential
       4. “This study was supported by a grant from the Janssen Pharmaceutica Research Foundation.”
17. Prolonged Treatment with Transdermal Fentanyl in Neuropathic Pain
    - [https://www.industrydocuments.ucsf.edu/docs/fyfg0230](https://www.industrydocuments.ucsf.edu/drug/docs/#id=fyfg0230)
    - Author : Dellemijn, Paul LI; Duijn, Hans van; Vanneste, Jan AL
    - Document Date : 1998 October 04
    - Type : graph; publication; table
    - ID : fyfg0230 ( TID : lcf71j00 )
    - ARK : ark:/88122/fyfg0230
    - Collection : Oklahoma Opioid Litigation Documents; Opioid Documents Collection
    - Key Points:
      1. Methods
         1. “Patients
            1. Subjects with noncancer neuropathic pain were recruited from our own outpatient clinic population and through telephone requests and letters to colleagues from Amsterdam and surroundings who were presumed to treat patients with neuropathic pain, such as neurologists, neurosurgeons, and anesthesiologists. All patients who completed a randomized, double-blind, active placebo-controlled trial with intravenous infusions of either FEN and diazepam or FEN and saline17 were invited to be enrolled in this second study assessing the benefits and risks of prolonged treatment with FENtd.”
            2. “Exclusion criteria were use of opioids or modified drug regimens during the 2 weeks before starting the study; contraindications to opioids, such as a history of opioid abuse; presence of multiple sites or other types of pain; intermittent neuropathic pain, such as trigeminal neuralgia; and uncertainty about the neuropathic origin of pain.”
         2. Cites Portenoy multiple times
18. ~~Actiq Approved~~
    - [~~https://www.industrydocuments.ucsf.edu/docs/ztgg0230~~](https://www.industrydocuments.ucsf.edu/drug/docs/#id=ztgg0230)
    - ~~Author : Unknown~~
    - ~~Document Date : Unknown~~
    - ~~Type : table~~
    - ~~ID : ztgg0230 ( TID : ypf71j00 )~~
    - ~~ARK : ark:/88122/ztgg0230~~
    - ~~Collection : Oklahoma Opioid Litigation Documents; Opioid Documents Collection~~
    - ~~Key Points:~~
      1. ~~Actiq speaker list~~
19. Article Summary Evaluation of Long-Term Efficacy and Safety of Transdermal Fentanyl in the Treatment of Chronic Noncancer Pain
    - [https://www.industrydocuments.ucsf.edu/docs/ssgg0230](https://www.industrydocuments.ucsf.edu/drug/docs/#id=ssgg0230)
    - Author : Janssen Pharmaceutical, Inc
    - Document Date : 2001 September 21
    - Type : memo
    - ID : ssgg0230 ( TID : gpf71j00 )
    - ARK : ark:/88122/ssgg0230
    - Collection : Oklahoma Opioid Litigation Documents; Opioid Documents Collection
    - Key Points:
      1. PNU on Milligan study
      2. *“Although there is the potential for bias with a open-label study, this design is more like a real world situation.”*
      3. “The majority of patients (95%) were receiving concomitant medications, such as long-term steroidal or nonsteroidal anti-inflammatory agents, antiepileptics, laxatives, antidepressants, and other psychtropic drugs.” “*Since this study population included patients with concomitant comorbidities and medications, it is a good representation of a real world setting.”*
      4. *“The authors stated that TDF provided stable, sustained, long-term pain control. Approximately 1/3 of the study population did not ‘respond’ to TDF. This coincides with Perry Fine’s comments (see editorial) that a process of trial and error is often needed to achieve adequate pain management.”*
      5. *“A possible explanation for the low rate of global efficacy is that, unlike pain control assessment, the results for the global efficacy measurement did not include a ‘moderate’ rating.”*
      6. *“The patients that withdrew during the first months of the trial did so as a result of an AE or insufficient response. Patients rarely withdrew from the study die to an AE or insufficient response after 6 months into the trial. This result may indicate that within the first months of the trial, most of the withdrawals secondary to insufficient response may be related to improper titration. Withdrawals secondary to AEs may be due to lack of tolerability to the transient side effects of TDF.*

*The authors stated that the incidence of Aes and the rate of withdrawal from the trial are relatively high but neith unusual nor unexpected considering the baseline clinical status of the study population. There were multiple comorbidities, as evidenced by their concomitant diagnoses and concomitant medications. In addition, these patients had low QoL scores at baseline.”*

**RESEARCH LAUNDERING**

11) Duragesic Fentanyl Transdermal System

- Document Data
  - **Author :** *Unknown*
  - **Document Date :** 2019 January 24
  - **Type :** report, marketing
  - **ID :** fkgg0230 ( TID : vjf71j00 )
  - **ARK :** ark:/88122/fkgg0230
  - **Collection :** Oklahoma Opioid Litigation Documents; Opioid Documents Collection
- Document Notes
  - What is this document? **Labeled as “Exhibit 4, Portenoy” -- seems to be a memo regarding the marketing of Duragesic (FENTANYL)**
  - Your commitment to sell DURAGESIC by being **ethically aggressive [????]** will continue to position the brand for double digit sales growth throughout 2002 and beyond
  - Market Update
    - Goal = take market share from OxyContin
  - Strategic Focus
    - PHYSICIAN TARGETING – increase frequency of calls to “high-decline” physicians to twice a month; goal is to increase DURAGESIC share at expense of OxyContin at targeted physicians
    - PATIENT TARGETING – **expand duragesic use in chronic, non-malignant pain (ex = lower back pain) = considerable growth oppo**, convince physicians that DURAGESIC is effective & safe.
    - CORE MESSAGE = LIFE UNINTERRUPTED – goal is to enhance current promotional message to show emphasis on patient functionality as primary goal of treatment
  - Sales Materials/Programs
    - Personal Selling
      - Revised visual aids – focus on Life uninterrupted + functionality benefit
      - **Milligan Study**—reprint this study; supports long-term treatmen tof chronic pain is effective and well-tolerated & improvement in functionality
        - **Alan & Simpson & Milligan Study** -- see Brian’s notes; also used in Oklahoma!; likely ghostwritten
        - Funded by Janssen; cites studies that are also wonky
    - Non-Personal Selling
      - Newsletter for pharmacists & CP patients – promotional message
      - *New Reality of Pain Management* (Direct mail program for clinicians, nurses, pharmacists) -- goal is to provide high-decile MD & PharmD with key DURAGESIC communication points
    - Medical Education
      - Invitation to Clinicians + pharmacists to **NEPC** – the NEPC is funded by an educational grant from Janssen. Invitation to participate in program for medical professionals about opiod therapy for chronic pain
      - Dr. DeLeon Cacner Pain Management TeleTopics
      - Dr. Passik Substance Abuse Issues TeleTopics -- focuses on identifying substance abuse problems and minimizing potential abuse problems

22) Addiction Rare in Patients Treated with Narcotics

Document Data

- **Author :** Boston University Medical Center; Porter, Jane; Jick, Hershel
- **Document Date :** 2018 November 30
- **Type :** article; publication
- **ID :** qkgg0230 ( TID : gkf71j00 )
- **ARK :** ark:/88122/qkgg0230
- **Collection :** Oklahoma Opioid Litigation Documents; Opioid Documents Collection

Document Notes

- Authors = Jane Porter, Hershel Hick, Boston University Medical Center
- NOTE: This paper was published in NEJM in 1980
  - **Letter to Editor – only 1 page long**
- “To the Editor; Recently, we examined our current files to determine the incidence of narcotic addiction in 39,946 hospitalized medical patients' who were monitored consecutively, Although there were 11,882 patients who received at least one narcotic preparation, there were only four cases of reasonably well documented addiction in patients who had no history of addiction. The addiction was considered major in only one instance, The drugs implicated were meperidine in two patients,' Percodan in one, and hydromorphone in one. We conclude that despite widespread use of narcotic drugs in hospitals, the development of addiction is rare inmedical patients with no history of addiction.”
- Exhibit 24; Portenoy

37) Doctor Who Wrote 1980 Letter on Painkillers Regrets that It Fed The Opioid Crisis

Document Data

- **Author :** *Unknown*
- **Document Date :** 2017 June 16
- **Type :** photograph; publication; website
- **ID :** qlgg0230 ( TID : wkf71j00 )
- **ARK :** ark:/88122/qlgg0230
- **Collection :** Oklahoma Opioid Litigation Documents; Opioid Documents Collection

Document Notes

- “A 1980 letter published in the NEJM was later widely cited as evidence that long-term use of opioid painkillers such as oxycodone was safe, even though the letter did not back up that claim”
  - **REFER TO #34: The Oklahoma Commission on Opioid Abuse Final Report**
  - **Letter to the editor:** <https://www.nejm.org/doi/pdf/10.1056/NEJM198001103020221>
  -
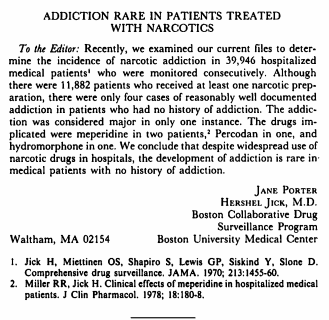

- Inaccurate representations of that 1980 letter led to a dramatic increase in the prescribing of opioids for chronic pain, according to an article (<http://www.nejm.org/doi/full/10.1056/NEJMcl700150>) published this month in the same medical journal by Dr. David Juurlink (<http://ihpme.utoronto.ca/faculty/davidjuurlink/>) of the University of Toronto, who researches drug safety. He and his co-authors found **more than 600 citations of the letter,** a majority of which failed to note that the patients whom Jick and Porter described were in hospitals for brief stays when prescribed opioids. Some of the citations "grossly misrepresented the conclusions of the letter," they found.
- "We believe that this citation pattern contributed to the North American opioid crisis by helping to shape a narrative that allayed prescribers' concerns about the risk of addiction associated with long-term opioid therapy," they write, pointing out that citations soared after the introduction of OxyContin in the mid1990s.
- "Only years and years later, that letter was used to advertise by new companies that were pushing out new pain drugs," he says. "I was sort of amazed. None of the companies came to me to talk to me about the letter, or the use as an ad."
- **He says the drug companies used his letter to conclude that their new opioids were not addictive**. "But that's not in any shape or form what we suggested in our letter." Asked whether he regrets having written the letter, Jick says, "The answer is, fundamentally, sure. The letter wasn't of value to health and medicine in and of itself. So if I could take it back — if I knew then what I know now, I would never have published it. It wasn't worth it."
- JICK: “Well, to be honest, I'm mortified because we have published roughly 400 papers on drug safety. But never before have we had one that got into such a bizarre and unhealthy situation.”

86) Pain Franchise 2012 PR Program

Document Data

- **Author :** *Unknown*
- **Document Date :** 2012 February 10
- **Type :** notes; report; report, scientific
- **ID :** nrgg0230 ( TID : lof71j00 )
- **ARK :** ark:/88122/nrgg0230
- **Collection :** Oklahoma Opioid Litigation Documents; Opioid Documents Collection

Document Notes

- Long Term PR Goal: **Build out narrative and deliver data points that establish NUCYNTA as the right choice for patients and a potential solution to a broader societal problem**
  - Establish NUCYNTA as new standard in moderate-severe pain
  - Demonstrate industry leadership in advocacy for HCP & patient access
- IOM Study: Relieving Pain in America ([LINK](https://healthland.time.com/2011/06/29/report-chronic-undertreated-pain-affects-116-million-americans/))
  - **New England Journal of Medicine editorial (Jan. 2012) from Dean of Stanford Med School** quotes IOM study More than 116 million Americans have chronic pain Financial costs ranging from $560 to $635 billion per year
- Seizing Media Opportunities
  - Two **RADAR** posters
    - Non-med use of Tapentadol
    - Non-med use of opioids/tapentadol among college students
  - Focus on **MILITARY** -- "The long road home"-lraq/Afghanistan troops the next gen of chronic pain patients
    - News Hook: Major troop withdrawals, return to civilian life with chronic pain
    - News Hook: Those who have served, need to be served
- Educate/Influence to Maintain Physician & Patient Access – Public affairs/policy support
  - Laser focus on states where access is threatened
- “Tell the true story of unmet medical need in chronic pain patients”

97) Senate Hearing on the IOM Report

Document Data

- **Author :** Brooks-Coley, Keysha; American Cancer Society Action Network
- **Document Date :** 2011 October 04
- **Type :** email
- **ID :** rggg0230 ( TID : vhf71j00 )
- **ARK :** ark:/88122/rggg0230
- **Collection :** Oklahoma Opioid Litigation Documents; Opioid Documents Collection

Document Notes

- Email from Will Rowe – CEO of American Pain Foundation
  - NOTE: **Will Rowe is listed as co-chair of the Pain Care Foundation IOM Task Force** (PCF = corporate group that works to advocate for pain medicine!!!!)
- Email sent to **presidents or every major pain advocacy group in America**
- Goal = “Help push this [IOM Report] over the line” in the Senate
  - IOM Report = Relieving Pain in America [[LINK](https://www.nap.edu/catalog/13172/relieving-pain-in-america-a-blueprint-for-transforming-prevention-care)]
- Excerpts from the email
  - Please review and consider signing onto the attached letter.
  - Please respond to Bonnie Weissfeld, bweissfeld(S)painfoundation.org , with your agreement to sign your organization onto this letter. **The more organizations the better.**
  - This hearing will be a great opportunity to educate members of Congress about the problem of pain and the information in the IOM report and enlist their **support in urging implementation of the recommendations**
  - This is a "Seize the Day" opportunity to leverage the IOM Report for **greater attention and investment** in improving pain care and research
- Response from Keysha Brooks-Coley = Director of American Cancer Society Cancer Action Network (ACS CAN): “*Will - Please add ACS CAN.”*

104) Pain Brief Advocacy & Policy Monthly 2011

Document Data

- **Author :** Kohn, Robyn
- **Document Date :** 2011 July 07
- **Type :** email; report
- **ID :** tygg0230 ( TID : vmf71j00 )
- **ARK :** ark:/88122/tygg0230
- **Collection :** Oklahoma Opioid Litigation Documents; Opioid Documents Collection

Document Notes

- SEE # 103 – this is an example of the Policy Monthly Briefing proposed by Kohn
- Primary External Partners
  - GO TO PARTNER = **American Pain Foundation**
  - American Academy of Pain Medicine
  - American Academy of Pain Management
  - American Chronic Pain Association
- Secondary External Partners
  - American Academy of Hospice & Palliative Care Medicine
  - American Academy of Family Physicians
  - American Geriatric Society
  - American Society of Consultant Pharmacists
  - American Academy of Physician Assistants
  - American Academy of Nurse Practitioners
- **KEY ADVOCACY THEMES**
  - Marketplace
    - **Significant unmet needs in moderate-severe chronic pain market**
    - Advocacy on the local market level gaining momentum among partner org
  - Pain Tools
    - **Prescribe Responsibly**-external HCP community
    - **Smart Moves, Smart Choices**-Teen RX Abuse
    - **Let's Talk Pain**-Provider/Patient Communications
- **NEW INITIATIVES**
  - **Imagine the Possibilities – Pain Coalition**
    - Internal cross-functional members of the pain teams and external members of the pain communities
    - Goal: align and address issues in pain management with emphasis on abuse and diversion
  - **New Partnership: State Pain Policy Action Coalition (SPPAC)-AAPM, APF, ASPMN**
    - Goal: Formed out of need to respond and influence positive state-based public policies is crucial to assuring access to effective care for persons experiencing pain.
    - **National pain management organizations have attempted to influence** many of these issues, but often have done so in an ineffective piecemeal and hit-or-miss fashion, failing to take advantage of opportunities to **speak with one voice and achieve desired outcomes.** SPPAC provides the opportunity to create a synergistic voice to insure the ability of our members to provide and receive optimal care for pain.
    - While three organizations are initially organizing the coalition, other organizations will be invited to join once the structure is developed.
- IOM REPORT (6/29/11): **Reliving Pain in America**
  - What is the IOM report: The report, released on June 30, 2011 — Relieving Pain in America: A Blueprint for Transforming Prevention, Care, Education, and Research — calls for a cultural transformation of attitudes toward pain and its prevention and management.
  - Noteworthy findings in IOM
    - Chronic pain affects an estimated **116 million** American adults
    - Pain costs the nation up to **$635 billion each year** in medical treatments and lost productivity.
    - Chronic pain negatively affects socioeconomic status.
    - Federal and state drug abuse prevention laws, regulations, and enforcement practices have been considered impediments to effective pain management...." Among other barriers, they say "**Twentynine percent of primary care physicians and 16 percent of pain specialists report they prescribe opioids less often than they think appropriate because of concerns about regulatory repercussions."**
  - Partner Commentary
    - **Pain Care Task Force** is being formed to develop a communication and outreach plan for dissemination of key messaging across national/local markets. The activities can serve as an advocacy tool for our partner organizations.
    - The committee calls for government agencies, healthcare providers, and public and private funders of health care to adopt a comprehensive, strategic approach to reduce or eliminate the barriers to pain care.

105) Prescriptive Authority Slide

Document Data

- **Author :** Janssen; Johnson and Johnson; Kohn, Robyn; Health Care Systems, Inc
- **Document Date :** 2011 July 05
- **Type :** email; report
- **ID :** gpgg0230 ( TID : ymf71j00 )
- **ARK :** ark:/88122/gpgg0230
- **Collection :** Oklahoma Opioid Litigation Documents; Opioid Documents Collection

Document Notes

- Email from Robyn Kohn (National Advocacy Director) -- **POWERPOINT: “2011-2012 Advocacy Launch Plan”**
- Critical Launch Success Factors
  - Continue to drive national and regional access
  - Understand & influence policy and legislative events to ensure appropriate patient access
  - **Collaborate with key patient advocacy organizations to advance awareness of under -treatment and under-management of pain**
- National Advocacy: Key Launch Components
  - Pain Patient Foundations: **APF, ACPA**
  - Provider Organizations: **APS, AAPM (medicine), AAPM (management)**
- National Advocacy: Local Advocacy
  - Patient Foundation: **APF Action Network, ASPI**
  - Provider Org: **APS, AAPM**
  - Individal KAL: **COEs, Medical/Professional Pain & Family Practice organizations**
- Recognizing pain as own disease state
  - Stigma of Pain
  - Validate, diagnosis, treatment, management – **UNDER TREATMENT OF PAIN**
- **Big problem = LIMITED ACCESS TO PRESCRIPTIVE AUTHORITY**
- Advocacy Support: National & Local needed for balanced pain policy position statements
  - Goals for Advocacy Support
    - **Remove barriers to access for pain treatment**
    - Educate partners and stakeholders on appropriate opioid use
  - **“We support unrestricted access to scheduled pain medications that are being used appropriately for the treatment of moderate to severe pain”**
  - **“We support intervention by the appropriate authorities to address the misuse, abuse, and diversion of scheduled pain medications”**
- Tier 1 Pain Organization: **American Pain Society, American Academy of Pain Medicine, American Pain Foundation/Action Network, COE**
- Goal = Coordinate/Collaborated network prepared to mobilize when issues on the state level need Advocacy Support from our:
  - **Pain Organizations**
  - **Professional Organizations**
  - **Patient Organizations**
  - **Medical & Health Care Organization**
- Pain state issues
  - States are moving quickly to address RX drug abuse – emphasis on opiates
  - Growing importance of role of national state organizations
  - Concerns about punitive policy that discourages appropriate pain management
- **CASE STUDIES – Examples of Successes**
  - PhRMA supports/actively lobbies PMP for first time (3/11) in Georgia
    - Law enacted w/ many of our desired provisions
    - **Defeated 60 unit per RX limited**
    - **Defeated wholesaler reporting requirement**
    - **Includes balanced pain management language**
  - FL Pill Mill Enacted with PMP
    - Secured inclusion of PMP
    - Favorable amendment added: wholesaler 5000 monthly pill limit removed
  - Defeated ND Worker’s Comp Restrictive Opiate Protoco
    - Bill would have made it extremely difficult to prescribe opiates for pain
    - Close collaboration between GA&P (Government affairs & policy) and SCG/Advocacy resulted in success
- **Landmark Advocacy reports Released by Obama Admin**
  - **NIH/IOM Report: State of pain – *validation of unmet need***
- **UNBRANDED INITIATIVES** -- a term I am seeing a lot in the documents
  - Unbranded Initiatives commonly mentioned in Jansen internal memos ([link](https://www.multivu.com/assets/51908/documents/51164-Janssen-Responsibility-Fact-Sheet-original.pdf))
    - **Let’s Talk Pain** -- TOOL FOR PATIENTS
      - Let’s Talk Pain is a coalition that encourages people with pain and their healthcare professionals to talk more about pain, to listen actively, and to act in ways that improve care for people with acute or chronic pain. The Let’s Talk Pain Coalition is a collaboration between the American Pain Foundation, the American Academy of Pain Management, the American Society for Pain Management Nursing, and Janssen Pharmaceuticals, Inc., which also sponsors the Coalition. LetsTalkPain.org features resources and interactive tools to help encourage an open dialogue about pain. These include tips to help patients talk with their healthcare professional, tips to help healthcare professionals talk to their patients, and several videos providing patient and healthcare professional perspectives, including two online video series, the “Let’s Talk Pain Show” and a medication safety series.
    - **Prescriber Responsibly** – TOOL FOR PHYSICIANS
      - Prescribe Responsibly is a website for healthcare professionals about the appropriate and responsible prescribing of opioid pain therapies for patients with acute and chronic pain. Physicians cite a number of concerns when prescribing opioid analgesics to people with pain, including risks of diversion and misuse.1 These concerns can present barriers to appropriate pain management, and may lead to the under-treatment of pain.2 Prescribe Responsibly addresses concerns physicians may have when prescribing opioid analgesics to patients with pain. PrescribeResposibly.com covers a range of topics related to the appropriate prescribing of opioid analgesics and offers case studies to help healthcare professionals assess various scenarios they may encounter when caring for patients with pain. Prescribe Responsibly also provides links to valuable tools that can help healthcare professionals assess pain levels as well as the risks for aberrant drug-related behavior
    - **Smart Moves, Smart Choices** – TOOL FOR TEENS
      - The National Association of School Nurses and Janssen Pharmaceuticals teamed up to create Smart Moves, Smart Choices, a national awareness program that informs parents, teens and educators about teen prescription drug abuse and its serious risks. SmartMovesSmartChoices.org features a wealth of resources, including myth-busting facts, tips for parents, an interactive quiz for teens, lesson plans and a school assembly tool kit for educators, and videos featuring addiction medicine specialist and TV host Dr. Drew Pinsky in conversations with teens, parents and educators.
  - “UI” appears in every email by Kohn (with the same 3 programs listed)
  - **More info on** [**UI**](https://www.pm360online.com/the-boldness-of-an-unbranded-campaign/)
    - when an unbranded campaign vigorously hones in on a specific unmet need that can ultimately direct HCPs to your brand—it is essential. It is worth its weight in gold, and it will indelibly illuminate the reasons to prescribe your product.
    - How can we be sure that an unbranded campaign will ultimately translate into greater prescription volume when we never even mention the product’s name?
    - Which is why an unbranded campaign can deliver to HCPs the type of information that will challenge them to overcome clinical inertia, reconsider their accepted protocols, and deviate from the status quo
    - Above all, an unbranded campaign must highlight a crisply defined unmet need that your product will in some way address
    - Goals of unbranded campaign
      - 1) Drive diagnosis through Disease Awareness
      - 2) Highlight the Pathophysiology & Burden of a Specific Disease
      - 3) Enhance Corporate Reputation


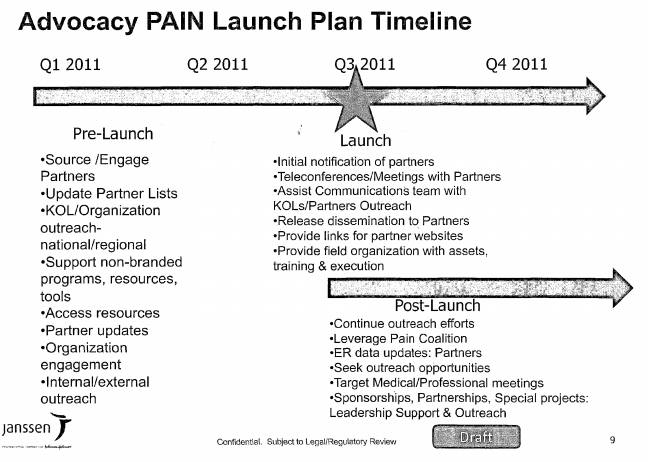


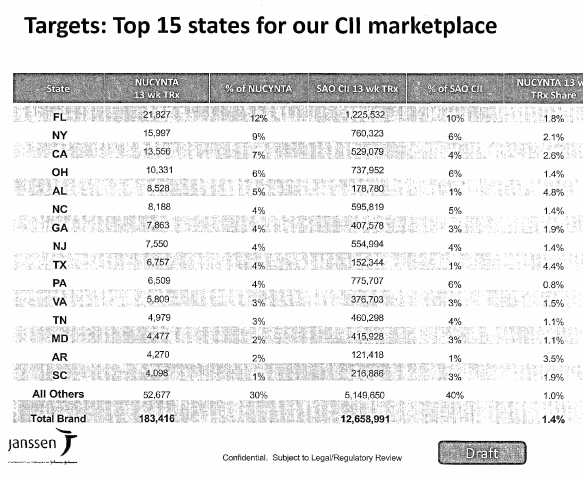


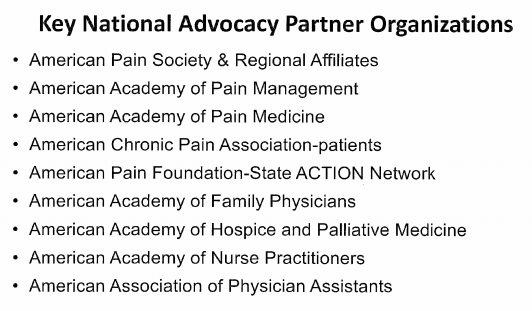


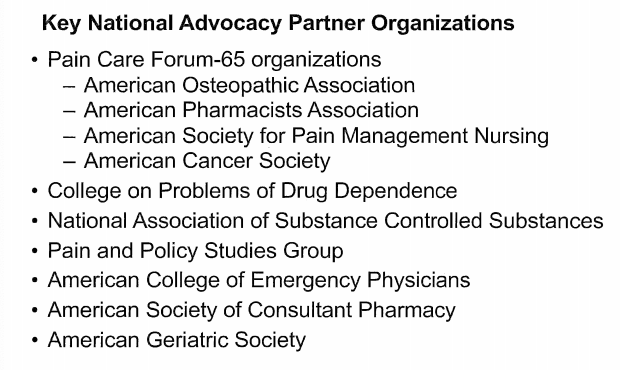


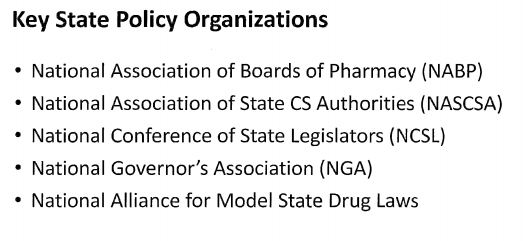


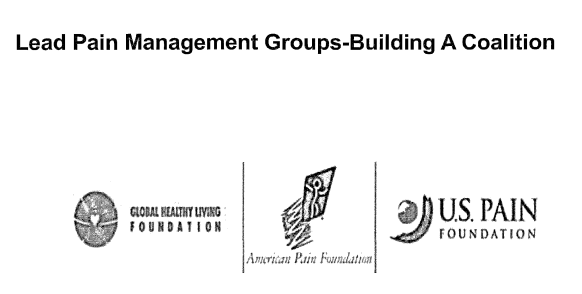


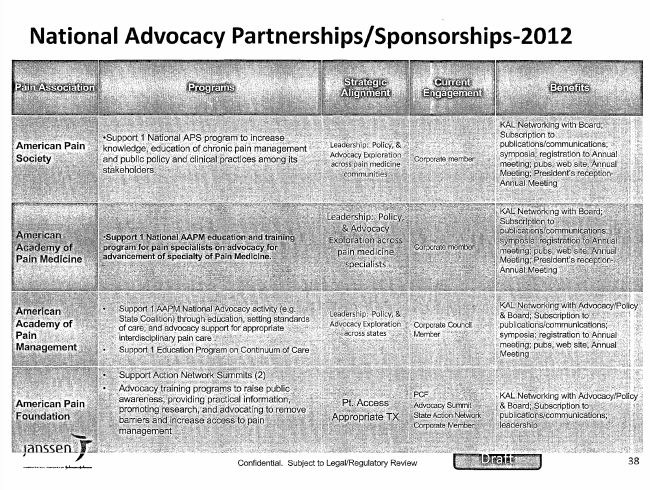


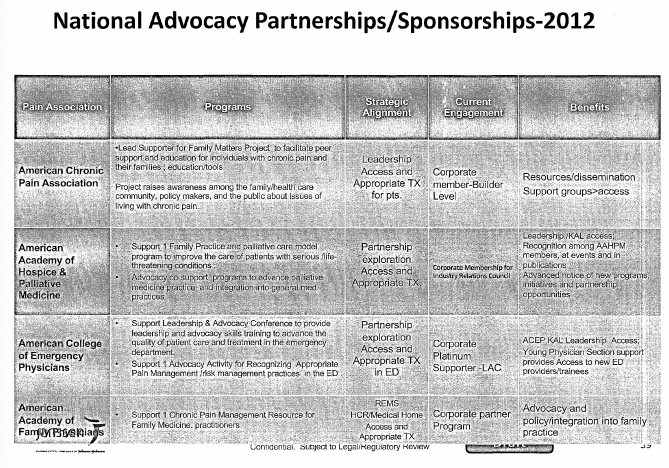


- Declaration of Russell K Portenoy, MD in State of Oklahoma v Purdue Pharma LP, et al
  - [https://www.industrydocuments.ucsf.edu/docs/nshg0230](https://www.industrydocuments.ucsf.edu/drug/docs/#id=nshg0230)
  - Author : District Court of Cleveland County, State of Oklahoma
  - Document Date : 2010 January 17
  - Type : legal; affidavit
  - ID : nshg0230 ( TID : xyf71j00 )
  - ARK : ark:/88122/nshg0230
  - Collection : Oklahoma Opioid Litigation Documents; Opioid Documents Collection
  - Key points:
    - “I have agreed to cooperate with certain plaintiffs who have entered into settlement agreements with me dismissing me as a defendant in their cases (‘Settling Plaintiffs’). Settling Plaintiffs agreed to dismiss me from their cases in exchange for my truthful cooperation. The proffer agreement with those Plaintiffs can be voided and the original lawsuits may be reinstated against me if my statements are recklessly and materially not truthful or accurate.”
    - “This declaration includes statements describing how my views about opioid therapy and its marketing by the pharmaceutical companies have changed during the period between the mid-1980s and the first decade of the 2000s.”
    - “Prior to, and then during the 1980s, opioids were disfavored for use in chronic, noncancer pain because of concerns that patients using opioids would develop tolerance and physical dependence, and be at risk for abuse, misuse, addiction, and diversion.^1^”
      - Portenoy RK, Foley KM. Chronic use of opioid analgesics in non-malignant pain: report of 38 cases. Pain. 1986;25:171-86.
    - Portenoy RK, Foley KM. Chronic use of opioid analgesics in non-malignant pain: report of 38 cases. Pain. 1986;25:171-86.
      - “This article was a retrospective case series describing anecdotal information about a highly selected group of patients. I recall that Dr. Foley and I wanted to write the paper to describe a phenomenon that we believed was under-appreciated by the medical community—the possibility of long-term pain relief from opioid therapy, without the development of tolerance leading to treatment failure and without the development of serious adverse effects, including drug abuse. We also wanted to use this description of cases as a starting point for a broad discussion of the clinical issues relevant to the appropriate use of these drugs. Our initial contribution to this discussion appeared in the Discussion section of the paper, which ended with our recommendation that opioid therapy be considered only after "all reasonable attempts at pain control have failed and persistent pain is the major impediment to improved function."^3^ Contrary to how some drug companies later used this article, it was never intended as a report of high-quality evidence, or as support for broad adoption of opioid therapy; it was a description of anecdotal information accompanied by a brief narrative review of the literature, and was intended to suggest that the role of long-term opioid therapy needed re-thinking, and more research, and that clinicians should not consider the approach to be contraindicated, but rather, worthy of consideration in the context of treatment refractory pain.”
    - “In my 1994 chapter, for example, I described a phenomenon that I then called *therapeutic dependence* and a phenomenon that had been labeled as *pseudoaddiction* in a 1989 article by Weissman and Haddox.^6^”
      - Weissman DE, Haddox JD, Opioid pseudoaddiction. Pain 1989;36(3):363-366.
    - “In all my writings, I acknowledged that the disease of addiction was a risk when opioids were used therapeutically. I stated in the 1994 chapter, as I did in many other writings during the 1980s and 1990s, that the disease of addiction would be "very unlikely" to develop when patients with chronic pain and no prior history of substance abuse were prescribed opioids and closely monitored. Although the evidence in support of this statement was scant in terms of the populations with chronic non-cancer pain, it was supported by large surveys of cancer patients receiving opioids for cancer pain. My conclusion was based on an analysis of the clinical literature at the time, which I acknowledged was very limited.”
    - “In 1996, a workgroup was formed by the American Academy of Pain Medicine and the American Pain Society for the purpose of creating a *Consensus Statement on the Use of Opioids for the Treatment of Chronic Pain*. The committee chair was Dr. J. David Haddox, who was hired by Purdue Pharma in 1999. I was not involved in the deliberations of the workgroup, but I recall that I was sent a draft of the consensus statement for my comments before it was presented to the respective organizations for approval and dissemination, I do not recall whether I made suggestions for modifications.”
    - {From p.15, a lengthy description of the nature of financial relationships with Defendants}
    - “I recall that some of these offers to write review articles, which I did not pursue, included the help of a medical writer for assistance in drafting the paper.”
    - “Based on the interactions that I have had with medical education vendors, I believe that academicians who are provided with honoraria for producing or editing material must be vigilant to avoid messages that are not well supported or prudent, and are in the interest of the drug company, without a corresponding medical benefit for the patient. Although I cannot cite specific cases, my experience suggests that some of the work ostensibly created by academicians through interaction with medical education vendors reflects the work or the influence of drug companies.”
    - “This type of controlled clinical trial provides what is considered to be highquality evidence. This evidence is needed by clinicians to confirm that the drugs they select can be efficacious when they are treating patients. However, these studies recruit patients based on strict inclusion criteria, and consequently, the results may or may not be immediately transferable to practice. In studies of opioids, the patients who are recruited are carefully selected and may not be representative of the overall population with pain; moreover, the duration of study is short compared to the patients treated in practice, and study patients are carefully monitored while they are receiving the study drugs. Clinical practice needs randomized controlled studies to provide an evidence base for patient care, but clinical guidelines are not created solely from the data acquired in these studies. It is my belief that this understanding of the role played by high-quality randomized clinical trials, i.e., necessary to establish the potential for efficacy but insufficient for clinical guidelines that must consider a patient population not represented in the study and patterns of drug use that are not specifically tested, is widely accepted by clinicians and investigators, and would be considered common knowledge in the pharmaceutical industry.”
    - “Addiction can exist while pain is experienced.”
    - “I believe that, over the years, some Defendant drug companies have used my work to promote opioids by referencing the positive statements that 1 made repeatedly without providing the background, analysis of the literature, and cautions that accompanied these positive statements.”
    - “It indicates that the risk of addiction is <1%, although this is an inaccurate interpretation of data (paragraph 40)…”
    - “Drug companies are a major source of research funding and have the ability to influence study proposals. In my opinion, it is clear that drug company research grants provided to academicians for studies of approved drugs generally fund studies that aim to identify or confirm benefits that would be helpful in marketing. Similarly, 1 believe that the drug companies distribute honoraria, fees and grants in a way that elevates specific messages, and messengers, that agree with their preferred messaging. Although I personally was never influenced to say things I did not believe, it is true that the drug companies provided me with many opportunities to express my views, and they used the positive statements that I made about opioids to portray opioid treatment as safe and effective without the accompanying discussion of risk that I included in the papers, chapters, and lectures I produced beginning in the 1980s.”
    - “My work was intended to disabuse clinicians of a bias against opioids by describing the literature as it existed then and the favorable outcomes that I and others were seeing in varied subgroups of patients, and by providing guidelines for treatment that included careful patient selection and vigilant monitoring of drug-related outcomes over time. I believe that the drag companies created material that narrowly focused on the potential for safe and effective treatment of chronic noncancer pain, some of which was attributed to my work, but failed to include an adequate and balanced discussion of the limitations in the relevant science and the risks as they were then known.”
    - “The practice of "enriched enrollment," which is a type of clinical trial design often used in pivotal trials supporting FDA approval of an opioid, exemplifies how these explanatory trials do not provide the type of information about risk that is needed in clinical practice. In these studies, there is an initial open-label phase to select patients who respond to a drug; this is then followed by a double-blind phase to determine whether these responders continue to have benefit from the drug when their effects are compared with a placebo treatment. Patients with addiction risk are typically screened out of the open-label phase, and patients who do not do well during the open-label phase are also dropped from the double-blind phase, Enriched enrollment studies are therefore designed only to see if a drug has a primary outcome, like analgesia, that exceeds placebo in a selected group of favored patients. It is not designed to assess outcomes in the heterogeneous population of patients seeking care in clinical practice, nor is it designed to assess long-term risks. Although drag companies should include documentation of evidence from explanatory trials in their labels, they should be cautious in pointing to these studies as proof of a drug's safety in real world conditions,17”
- Evaluation of Long-Term Efficacy and Safety of Transdermal Fentanyl in the Treatment of Chronic Non cancer Pain
  - [https://www.industrydocuments.ucsf.edu/docs/rsgg0230](https://www.industrydocuments.ucsf.edu/drug/docs/#id=rsgg0230)
  - Author : Milligan, Keith; Lanteri-Minet, Michael; Borchert, Klaus; Helmers, Henk; Donald, Royden
  - Document Date : Unknown
  - Type : article; graph; report, scientific
  - ID : rsgg0230 ( TID : fpf71j00 )
  - ARK : ark:/88122/rsgg0230
  - Collection : Oklahoma Opioid Litigation Documents; Opioid Documents Collection
  - Key points:
    1. International, multicenter, open-label trial
    2. Objective: “assess the efficacy and safety of up to 12 months of therapy with transdermal therapeutic system (TTS) fentanyl in patients (n=532) with chronic noncancer pain.”
    3. Main outcome measures: “pain control assessment, global treatment satisfaction, patient preference for TTS fentanyl, and quality of life.”
    4. Authors:
       1. Keith Milligan – Anaesthetic Department, South Cleveland Hospital, Middlesbrough, UK
       2. Michel Lanteri-Minet – Department of Pain Treatment, Hôpital Pasteur, Nice, France
       3. Klaus Borchert – Anästhesiologische Praxis, Greifswald, Germany
       4. Henk Helmers – Department of Anaesthesiology, Eemland Hospital, Amersfoort, The Netherlands
       5. Royden Donald – Strand Private Hospital, Cape Town, South Africa
       6. Hans-Georg Kress – Universitätsklinik für Allgemeine Anästhesie und Intensivmedizin, Abt. B, Wien, Austria
       7. Hugo Adriaensen – Universitair Ziekenhuis Antwerpen, Edegem, Belgium
       8. Dwight Moulin – Neurology Department, Victoria Hospital, London, Canada
       9. Voitto Järvimäki – Pain Clinic, Oulu University, Central Hospital, Oulu, Finland
       10. Ludo Haazen – Janssen Research Foundation, Beerse, Belgium
    5. “Supported by a grant from the Janssen Research Foundation, Belgium. K. M. has been reimbursed by Janssen-Cilag, the manufacturers of transdermal fentanyl, for attendance at a congress. L. H. was employed by the Janssen research Foundation when this study was performed.”
    6. Introduction
       1. “Evidence suggests, however, that pain is frequently undertreated even for these indications {acute pain and cancer pain}.^2,3^”
       2. “Persistent, albeit largely unfounded, fears about the risks of addiction, toxicity, physical dependence, and tolerance have led to the rejection of opioid analgesia for chronic pain resulting from noncancer disease.^4^”
       3. “Although few prospective clinical studies of opioid use in noncancer pain have been reported, evidence from surveys suggests that long-term opioid therapy can be used successfully in some patients with chronic nonmalignant pain without causing undue adverse events (AEs),^4-6^ a conclusion supported by several recent short-term, randomized controlled trials.^7-9^”
       4. “Reflecting this evidence and changing attitudes toward the therapeutic goals of treating noncancer pain, guidelines for the use of opioids in this setting have recently been proposed.^10-11^ These advocate scheduled around-the-clock drug administration and the use of long-acting preparations or sustained-release formulations to maintain therapeutic serum concentrations.”
    7. Materials and Methods
       1. “Exclusion criteria included a history of allergy or hypersensitivity to opioids; life-threatening disease; skin disease precluding the use of the transdermal system; reduced level of consciousness or inability to give informed consent; pregnancy, lactation, or possibility of pregnancy; social isolation; concomitant psychiatric disorders (depression excluded); history of substance abuse (assessed from the patient's clinical history and communication with the primary care practitioner); history of clinically relevant cardiac, nervous system, or respiratory disease or participation in any clinical trial, except the Allan study,^18^ within the preceding 30 days.”
    8. Statistical Analyses
       1. “The sample size of 500 patients was based on an estimated 2% incidence of AEs to provide a narrow and precise 95% confidence interval of 0.8% to 3.22%.”
    9. Results
       1. “A total of 532 patients were recruited, of whom 103 had participated in the study by Allan et al^18^;”
       2. “Of the 532 patients recruited, 301 (57%) patients completed the trail and 231 (43%) patients discontinued treatment prematurely.”
       3. “A total of 130 (25%) of the safety analysis population (n=530) discontinued TDF as a result of AEs.”
       4. “The most commonly used opioids immediately before study entry were morphine (48%) and fentanyl (28%, including patients from the Allan study^18^).”
       5. “A total of 375 of 421 (89%) patients stated a treatment preference (patients participating in the Allan study^18^ and those already on TTS fentanyl were not asked to express a preference).”
    10. Discussion
        1. “Stability of pain control during the 12-month period was achieved at the cost of an increase in mean TDF dosage from 48 to 90 μg/h. The largest increase occurred during the first months of treatment, as the patients titrated the dose upward themselves, and then stabilized during prolonged treatment. This increase may be due to the individual or combined effects of using conservative conversion table, underdosing at baseline, and the development of tolerance.”
        2. “Unlimited access to rescue medication, which is not always standard clinical practice, could have led to patients titrating the dose themselves to best effect, and consequently a subsequent increase in TDF dose when daily consumption of rescue morphine reached 60 to 90 mg. The average daily consumption of rescue medication was a reflection of increased pain intensity because it was probably related to an increased or altered physical function and subsequent pain at varying times.^10^ Experience with opioids has shown that tolerance to their analgesic effects is uncommon in patients with chronic pain.^10,23^ Many surveys and supportive clinical experience regarding the long-term use of opioids in patients with non-cancer pain have shown the development of tolerance not to be a clinical problem.^6,24^”
        3. “Opioid withdrawal symptoms were only reported in 3% of patients, suggesting that prolonged treatment is not associated with increased risk of withdrawal syndromes. There were no reports of addictive behavior in any of the patients during this long-term study. Because the fear of addiction is one of the reasons for the underuse of opioids in chronic noncancer pain, this study provides further evidence that these fears are unfounded. Our results also confirm that there is no basis for concern about persistent opioid side effects and long-term toxicity.^6^”
    11. Acknowledgments

“We thank all of the other investigators who participated in the trial: **J. Mayaert, L. Plaghki** (Belgium); **J. Clark,** H. Hays, **A. Mailis,** **M. Ong-Lam, D. Reid, P. Watson** (Canada); **E. Kalso** (Finland); N. Attal, F. Boreau, L. Brasseur, J. Bruxelle, P. Ginies, E. Lajous, S. Perrot, A. Serrie (France); **L. Allan**, **R. Atkinson**, G. Batchelor, **F. Campbell**, G. Carmichael, B. Collett, H. Dunckley, M. Fallon, I. Finlay, W. Fitch, **R. Gautam**, **M. Hanna**, D. Laird, D. Littlewood, W. Loan, K. Markham, L. Morrison, **W. Notcutt**, K. Rogers, P. Selby (UK); A. Beyer, W. Dinter, L. Petracic (Germany); and **G. Braak,** P. Brouwer, **G. van Oss, W. Zuurmond** (The Netherlands). We also thank the following contributors : At Janssen Research Foundation: L. Haazen was responsible for international trial supervision and coordination and was involved in the writing of the article (he will act as guarantor); **D. Peelmans**, F. Spensieri, **N. Currie, M. Jarvinen**, B. Flaisler, I. Broudic, A. Bessems, A. Schmidt-Mertens , D. Tseneklidou-Stoeter, **M. Travers**, D. Mathewson, D. Coakes, **M. Uitendaal**, W. Schipper, **P. Matthysen** (trial coordination and monitoring); and H. Swinnen, E. Everaert, I. van den Broeck, M. Nijs, **L. Bijnens**, H. Joosen (clinical data processing).”

- - - 1. No Janssen names returned results in DIDA
    1. References

1. Cleeland CS: Undertreatment of cancer pain in elderly patients. JAMA 279:1914-5, 1998
2. Zenz M, Zenz T, Tryba M, Strumpf M; Severe undertreatment of cancer pain: A 3-year survey of the German situation. J Pain Symptom Manage 10:187-91, 1995
3. Portenoy RK: Chronic opioid therapy in nonmalignant pain. J Pain Symptom Manage 5(suppl):546-62, 1990
4. France RD, Urban BJ, Keefe FJ: Long-term use of narcotic analgesics in chronic pain. Soc Sci Med 19:1379-82, 1984
   1. n=16
5. Portenoy RK: Opioid therapy for chronic nonmalignant pain: A review of the critical issues. J Pain Symptom Manage 11:203-17, 1996
6. Arkinstall W, Sandler A, Goughnour B, Babul N, Harsanyi Z, Drake A: Efficacy of controlled-release codeine in chronic non-cancer pain: A randomized, placebo-controlled trial. Pain 62:169-78, 1995
   1. FUNDED BY PURDUE
7. Jadad A, Carroll D, Glynn C, Moore R, McQuay H: Morphine responsiveness of chronic pain: Double-blind randomised crossover study with patient-controlled analgesia. Lancet 339:1367-71, 1992
   1. n=10
8. Moulin D, lezzi A, Amireh R, Sharpe W, Boyd D, Merskey H: Randomised trial of oral morphine for chronic non-cancer pain. Lancet 347:143-7, 1996
   1. FUNDED BY PURDUE
   2. n=46
9. Portenoy RK: Opioid therapy for chronic nonmalignant pain: Current status, in Fields HL, Liebeskind JC (eds): Progress in Pain Research and Management; Pharmacologic Approaches in the Treatment of Chronic Pain: New Concepts and Critical Issues. Seattle, WA, IASP Press, 1994
10. Schug S, Merry A, Acland R: Treatment principles for the use of opioids in pain of non-malignant origins. Drugs 42:228-39, 1991
    1. Behind paywall
11. Allan L, Hays H, Jensen N H, de Waroux BLP, Bolt M, Donald R, Kalso E: Randomised crossover trial of transdermal fentanyl and sustained release oral morphine for treating chronic non-cancer pain. BMJ 322:1154-1158, 2001
12. Coyle N, Adelhardt J, Foley KM, Portenoy RK: Character of terminal illness in the advanced cancer patient: Pain and other symptoms in the last 4 weeks of life. J Pain Symptom Manage 5:83-93,1990
13. Collett B-J: Opioid tolerance: The clinical perspective. Br J Anaesth 81:58-68, 1998
14. Galer BS, Coyle N, Pasternak GW, Portenoy RK: Individual variability in response to different opioids: Report of five cases. Pain 49:87-91, 1992
15. Randomised Crossover Trial of Transdermal Fentanyl and Sustained Release Oral Morphine for Treating Chronic Non-Cancer Pain
    1. [https://www.industrydocuments.ucsf.edu/docs/qsgg0230](https://www.industrydocuments.ucsf.edu/drug/docs/#id=qsgg0230)
    2. Author : Allan, Laurie; Hays, Helen; Jensen, Niels-Henrik; de Waroux, Bernard Le Polain; Bolt, Michiel; Kalso, Eija; Donald, Royden
    3. Document Date : 2001 May 12
    4. Type : publication; article
    5. ID : qsgg0230 ( TID : epf71j00 )
    6. ARK : ark:/88122/qsgg0230
    7. Collection : Oklahoma Opioid Litigation Documents; Opioid Documents Collection
    8. Key Points:
       1. Authors
          1. Laurie Allan – (director) Chronic Pain Services, Northwick Park and St. Mark’s NHS Trust, Harrow, Middlesex HA1 3UJ
          2. Helen Hays – (associate clinical professor) Department of Family Medicine, University of Alberta, Edmonton, Alberta, Canada T6G 2C8
          3. Niels-Henrik Jensen – (head of department) Multidisciplinary Pain Centre, Department of Anaesthesiology, Herlev University Hospital, DK-2730, Denmark
          4. Bernard Le Polain de Waroux – (staff anaesthesiologist) Clíniques Universitaires St-Luc, 1200 Brussels, Belgium
          5. Michiel Bolt – (anaesthesiologist) Alg Ziekenhuis Eemland De Lichtenberg, 3818 ES Amersfoort, Netherlands
          6. Royden Donald – (specialist anaesthetist) Strand Private Hospital, Cape Town 7139, South Africa
          7. Eija Kalso – (head) Helsinki University Central Hospital Pain Clinic, 00290 Helsinki, Finland
       2. “Objectives: To compare patients’ preference for transdermal fentanyl or sustained release oral morphine, their level of pain control, and their quality of life after treatment.”
       3. “Design: Randomised, multicenter, international, open label, crossover trial.”
       4. “Main outcome measures: Patients’ preference for transdermal fentanyl or sustained release oral morphine, pain control, quality of life, and safety assessments.”
       5. Introduction
          1. “Pain is often undertreated or mistreated, with patients going from doctor to doctor for relief and finally moving outside mainstream medicine in increasing numbers.^2”^
          2. “Opioids are the mainstay of management of cancer pain, providing effective pain relief.^3 4^ Opioids are the most powerful analgesics, but politics, prejudice, and continuing ignorance still impede optimum prescribing.^5^ A review of retrospective and survey data confirms the efficacy of opioids in the treatment of chronic non-cancer pain and found that fears of addiction were not justified.^6^ Randomised controlled trials of intravenous opioids in chronic non-cancer pain show benefit over placebo for morphine and fentanyl, whereas oral placebo controlled trials show efficacy for codeine, morphine, and oxycodone.^7–11^ Worldwide, the value of opioids in this role has led to the development of management guidelines, with recommendations from national organisations.^12–15^”
       6. Participants and methods
          1. “At crossover, patients received the same opioid dose as before the study.”
          2. “Patients were prescribed immediate release morphine (initially 5 mg) every four hours as needed. Patients requiring more than 60 mg of this rescue drug over two days of a three day period with fentanyl could increase their fentanyl dose. Patients receiving morphine needing more than two doses of the rescue drug per day could titrate to a higher dose of morphine.”
          3. “Details of all adverse events and presumed relation to the drugs were noted by the investigator.”
       7. Results
          1. “Sixty patients withdrew; 37 because of adverse events, five because of insufficient efficacy, and 18 for other reasons. Five patients without baseline data were excluded from the efficacy analysis. All patients were included in the safety analysis.”
          2. “The mean starting dose of transdermal fentanyl was 39.7 ìg/hr (range 25-200 ìg/hr) and of sustained release oral morphine 123.0 mg/24 hr (range 10-700 mg/24 hr). The mean dose of fentanyl at the end of the study was 57.3 ìg/hr (range 0-325 ìg/hr) and of morphine 133.1 mg/24 hrs (range 0-800 mg/24 hrs).”
          3. “The predominant reason given for preferring fentanyl was better pain relief, followed by greater convenience and fewer adverse events (table 3).” “Patients treated with transdermal fentanyl had on average lower pain intensity scores than those treated with sustained release oral morphine (mean 57.8, range 33.1-82.5 v mean 62.9, range 41.2-84.6; P < 0.001), irrespective of the order of treatment.” “Analysis of the consumption of rescue drug during the last three weeks of each treatment period showed that the mean (standard deviation) consumption was significantly higher with fentanyl (29.4 (33.0) mg) than with morphine (23.6 (32.0) mg; P < 0.001). A significant (P < 0.05) period effect was also observed: the higher consumption during fentanyl treatment was more apparent in the second trial period (mean 32.4 (SD 38.5) mg) than the first (26.3 (26.0) mg), where the consumption of the rescue drug remained essentially the same over the two treatment periods in the morphine group (23.7 (35.3) mg v 23.6 (27.3) mg).”
       8. Discussion
          1. No discussion of abuse potential or addiction throughout
       9. Acknowledgments
          1. “We thank all the investigators who participated in the trial: **J Maeyaert, L Plaghki** (Belgium); **J Clark**, A Mailis, **D Moulin**, **M Ong-Lam**, **D Reid, P Watson** (Canada); S Andersen, C Christiansen, S Clemensen, K Glahn, T Jonsson, S Larsen, F Molke Borgbjerg, A Schou Olesen, J Mølgaard (Denmark); V Järvimäki, T Heiskanen (Finland); **R Atkinson**, P Brown, **F Campbell**, **R Gautam, M Hanna**, D Hughes, C Knight, **W Notcutt** (United Kingdom); **G Braak**, J Helmers, **G Van Oss, W Zuurmond** (Netherlands); D Lines (South Africa). Contributors: LA was the principal author of the paper. H Noorduin (international supervisor of the trial), **L Bijnens** (biostatistics), **L Haazen, M Travers, D Peelmans, N Currie,** A Jepsen, **M Jarvinen, M Uitendaal, P Matthysen** (local trial coordination and monitoring). MT will act as guarantor for the paper. Funding: The study was supported by a grant from Janssen Research Foundation, Belgium. Competing interests: LA receives support from both Janssen-Cilag, the manufacturer of transdermal fentanyl (Durogesic) and Napp Laboratories, the manufacturer of sustained release morphine. EK has been reimbursed by Janssen-Cilag for participation at a meeting sponsored by Janssen-Cilag.”
       10. References
           1. (2) Ashburn MA, Staats PS. Management of chronic pain. Lancet 1999;353:1865-9.
           2. (3) Portenoy RK. Opioid and adjuvant analgesics. In: Mitchell M, ed. Pain 1999—an updated review. Seattle: IASP Press, 1999:3-18.
           3. (4) World Health Organization. Cancer pain relief, 2nd ed. Geneva: WHO, 1996
           4. (5) McQuay H. Opioids in pain management. Lancet 1999;353:2229-32.
              1. “Opioids are our most powerful analgesics, but politics, prejudice, and our continuing ignorance still impede optimum prescribing.” {Direct quote in Allan et al.}
              2. “Drug addicts are not in pain. The political message is that the medical use of opioids does not create drug addicts, and restrictions on this medical use hurt patients.”
           5. (6) Portenoy RK. Opioid therapy for chronic non-malignant pain: a review of critical issues. J Pain Symptom Manage 1996;11:203-17.
           6. (7) Rowbotham MC, Reisner-Keller LA, Fields HL. Both intravenous lidocaine and morphine reduce the pain of postherpetic neuralgia. Neurology 1991;41:1024–8.
           7. (8) Dellemijn PLI, Vanneste JAL. Randomised double-blind active-placebocontrolled crossover trial of intravenous fentanyl in neuropathic pain. Lancet 1997;349:753-8.
           8. (9) Arkinstall W, Sandler A, Goughnour B, Babul N, Harsanyi Z, Drake A. Efficacy of controlled-release codeine in chronic non-malignant pain: a randomized, placebo-controlled clinical trial. Pain 1995;62:169-78.
              1. FUNDED BY PURDUE
           9. (10) Moulin DE, Iezzi A, Amireh R, Sharpe WKJ, Boyd D, Merskey H. Randomised trial of oral morphine for chronic non-cancer pain. Lancet 1996;347:143-7.
              1. FUNDED BY PURDUE
              2. n=46
           10. (11) Watson CPN, Babul N. Efficacy of oxycodone in neuropathic pain: a randomized trial in postherpetic neuralgia. Neurology 1998;50:1837-41.
           11. (12) Schug SA, Merry AF, Acland RH. Treatment principles for the use of opioids in pain of nonmalignant origin. Drugs 1991;42:228-39.
           12. (13) Brown RL, Fleming MF, Patterson JJ. Chronic opioid analgesic therapy for chronic low back pain. J Am Board Fam Pract 1996;9:191-204.
           13. (14) Graziotti PJ, Goucke CR. The use of oral opioids in patients with chronic non-cancer pain. Management strategies. Med J Austr 1997;167:30-4.
           14. (15) Anon. The use of opioids for the treatment of chronic pain: a consensus statement from the American Academy of Pain Medicine and the American Pain Society. Pain Forum 1997;6:77–9.
16. Transdermal Fentanyl as Treatment for Chronic Low Back Pain
    1. [https://www.industrydocuments.ucsf.edu/docs/tsgg0230](https://www.industrydocuments.ucsf.edu/drug/docs/#id=tsgg0230)
    2. Author : Simpson Jr, Richard K; Edmondson, Everton A; Constant, Charles F; Collier, Connie; Journal of Pain and Symptom Management; Baylor College of Medicine; Elsevier; US Cancer Pain Relief Committe
    3. Document Date : 1997 October 04
    4. Type : article; bibliography; publication
    5. ID : tsgg0230 ( TID : hpf71j00 )
    6. ARK : ark:/88122/tsgg0230
    7. Collection : Oklahoma Opioid Litigation Documents; Opioid Documents Collection
    8. Key Points:
       1. Authors
          1. Richard K. Simpson, Jr, MD, PhD
          2. Everton A. Edmondson, MD
          3. Charles F. Constant, MPH, PhD
          4. Connie Collier, RN
          5. Department of Neurosurgery, Baylor College of Medicine, Houston, Texas
       2. Overall less aggressive in promoting opioids for chronic pain; notably, an earlier study than Allan or Milligan
       3. No discussion of addiction or abuse potential
       4. “This study was supported by a grant from the Janssen Pharmaceutica Research Foundation.”
17. Chronic Therapy for Nonmalignant Pain
    1. [https://www.industrydocuments.ucsf.edu/docs/xtgg0230](https://www.industrydocuments.ucsf.edu/drug/docs/#id=xtgg0230)
    2. Author : Portenoy, Russell K
    3. Document Date : Unknown
    4. Type : report; report, scientific
    5. ID : xtgg0230 ( TID : mpf71j00 )
    6. ARK : ark:/88122/xtgg0230
    7. Collection : Oklahoma Opioid Litigation Documents; Opioid Documents Collection
18. Chronic Therapy for Nonmalignant Pain
    1. [https://www.industrydocuments.ucsf.edu/docs/xtgg0230](https://www.industrydocuments.ucsf.edu/drug/docs/#id=xtgg0230)
    2. Author : Portenoy, Russell K
    3. Document Date : Unknown
    4. Type : report; report, scientific
    5. ID : xtgg0230 ( TID : mpf71j00 )
    6. ARK : ark:/88122/xtgg0230
    7. Collection : Oklahoma Opioid Litigation Documents; Opioid Documents Collection
19. Cochrane Review: Long-Term Opioid Management for Chronic Noncancer Pain
    1. [https://www.industrydocuments.ucsf.edu/docs/yrhg0230](https://www.industrydocuments.ucsf.edu/drug/docs/#id=yrhg0230)
    2. Author : Treadwell Jr, Noble M; Tregear, SJ; Coates, VH; Wiffen, PJ; Akafomo, C; Schoelles, KM; Chou, R
    3. Document Date : 2010
    4. Type : report, clinical study; publication
    5. ID : yrhg0230 ( TID : iyf71j00 )
    6. ARK : ark:/88122/yrhg0230
    7. Collection : Oklahoma Opioid Litigation Documents; Opioid Documents Collection
    8. Key Points:
       1. Defense exhibit
       2. Systematic review “To assess safety, efficacy, and effectiveness of opioids taken long-term for CNCP.”
       3. “PLAIN LANGUAGE SUMMARY

Opioids for long-term treatment of noncancer pain

The findings of this systematic review suggest that proper management of a type of strong painkiller (opioids) in well-selected patients with no history of substance addiction or abuse can lead to long-term pain relief for some patients with a very small (though not zero) risk of developing addition, abuse, or other serious side effect. However, the evidence supporting these conclusions is weak, and longer-term studies are needed to identify the patients who are most likely to benefit from treatment.”

1. What Percentage of Chronic Nonmalignant Pain Patients Exposed to Chronic Opioid Analgesic Therapy Develop Abuse/Addiction and/or Aberrant Drug-Related Behaviors? A Structured Evidence-Based Review
   - [https://www.industrydocuments.ucsf.edu/docs/hlfg0230](https://www.industrydocuments.ucsf.edu/drug/docs/#id=hlfg0230)
   - [https://www.industrydocuments.ucsf.edu/docs/mnfg0230](https://www.industrydocuments.ucsf.edu/drug/docs/#id=mnfg0230)
   - Author : Fishbain, David A; Cole, Brandly; Lewis, John; Rosomoff, Hubert L; Rosomoff, R Steele
   - Document Date : 2018 December 12
   - Type : publication; article
   - ID : hlfg0230 ( TID : raf71j00 ); mnfg0230 ( TID : ccf71j00 )
   - ARK : ark:/88122/hlfg0230; ark:/88122/mnfg0230
   - Collection : Oklahoma Opioid Litigation Documents; Opioid Documents Collection
   - Key Points:
     1. Systematic review submitted by defendants
2. Pseudoaddiction Revisited: a Commentary on Clinical and Historical Considerations
   - [https://www.industrydocuments.ucsf.edu/docs/fnfg0230](https://www.industrydocuments.ucsf.edu/drug/docs/#id=fnfg0230)
   - Author : Passik, Steven D; Kirsh, Kenneth L; Webster, Lynn
   - Document Date : 2011
   - Type : publication; article
   - ID : fnfg0230 ( TID : vbf71j00 )
   - ARK : ark:/88122/fnfg0230
   - Collection : Oklahoma Opioid Litigation Documents; Opioid Documents Collection
3. Prolonged Treatment with Transdermal Fentanyl in Neuropathic Pain
   - [https://www.industrydocuments.ucsf.edu/docs/fyfg0230](https://www.industrydocuments.ucsf.edu/drug/docs/#id=fyfg0230)
   - Author : Dellemijn, Paul LI; Duijn, Hans van; Vanneste, Jan AL
   - Document Date : 1998 October 04
   - Type : graph; publication; table
   - ID : fyfg0230 ( TID : lcf71j00 )
   - ARK : ark:/88122/fyfg0230
   - Collection : Oklahoma Opioid Litigation Documents; Opioid Documents Collection
   - Key Points:
     1. Methods
        1. “Patients
           1. Subjects with noncancer neuropathic pain were recruited from our own outpatient clinic population and through telephone requests and letters to colleagues from Amsterdam and surroundings who were presumed to treat patients with neuropathic pain, such as neurologists, neurosurgeons, and anesthesiologists. All patients who completed a randomized, double-blind, active placebo-controlled trial with intravenous infusions of either FEN and diazepam or FEN and saline17 were invited to be enrolled in this second study assessing the benefits and risks of prolonged treatment with FENtd.”
           2. “Exclusion criteria were use of opioids or modified drug regimens during the 2 weeks before starting the study; contraindications to opioids, such as a history of opioid abuse; presence of multiple sites or other types of pain; intermittent neuropathic pain, such as trigeminal neuralgia; and uncertainty about the neuropathic origin of pain.”
        2. Cites Portenoy multiple times
4. The Epidemic of Pain in America
   - [https://www.industrydocuments.ucsf.edu/docs/yqgg0230](https://www.industrydocuments.ucsf.edu/drug/docs/#id=yqgg0230)
   - Author : American Pain Foundation, The; Rogers, Mike
   - Document Date : 2006 June 13
   - Type : report
   - ID : yqgg0230 ( TID : wnf71j00 )
   - ARK : ark:/88122/yqgg0230
   - Collection : Oklahoma Opioid Litigation Documents; Opioid Documents Collection
   - Key Points:
     1. Extensive report (133 pages) supporting the concept of undertreatment of pain
5. [Agenda for the Chronic Pain Scientific Advisory Board]
   - [https://www.industrydocuments.ucsf.edu/docs/fggg0230](https://www.industrydocuments.ucsf.edu/drug/docs/#id=fggg0230)
   - Author : Chronic Pain Scientific Advisory Board
   - Document Date : 2001 November 30
   - Type : agenda
   - ID : fggg0230 ( TID : jhf71j00 )
   - ARK : ark:/88122/fggg0230
   - Collection : Oklahoma Opioid Litigation Documents; Opioid Documents Collection
   - Key Points:
     1. Confidential
     2. “Objective: KOLs to visit Janssen, understand J&J and Janssen, introduce Janssen’s EMRP research program with MSLs as key communication links, find common interests for research alliances, obtain feedback on the EMRP research agenda, and discuss issues related to abuse and diversion of opioids.”
     3. “Audience: 17 KOLs (List attached)”
     4. “EMRP definition:
        1. Studies focused on Duragesic in non-malignant pain states (Schein)
        2. ‘Nimble’ trials, tightly designed, small-scale, clearly defined with limited duration
        3. Pilot studies
        4. Anticipated output: abstracts presented at Association meetings (e.g., AAPM, APS)
        5. Funding has ranged from $15K - $200K and is dependent on:
           1. # of subjects
           2. complexity of the protocol, e.g., retrospective study ~$15K-25K, larger, multisite study ~$200K, average ~$100K
           3. complexity of assessments
           4. # researchers involved
           5. # required auxiliary personnel (e.g., statisticians)”
     5. “The goals for EMRP studies should be explicitly stated: Janssen wants to obtain certain data and seed studies that, after completion, may be expanded by funding from other sources.”
     6. “Review of the Allan and Milligan Data

Cheryl Pavia, PharmD

Cheryl's review of the new Allan and Milligan data, which came in between the EMRP sessions summarized above, was well received and elicited significant discussion. Many participamnts seemed unfamiliar with the studies. Many questions arose, which Cheryl fielded expertly.

- - - 1. Why was Allan's reported "better pain relief" with Duragesic accompanied by higher use of rescue medications.
      2. Cheryl responded that, because Duragesic has 4 patch strengths, many in-between doses may have been required.
      3. A "major stumbling block" in using Duragesic is the difficulty of converting from SAOs or other LAOs to Duragesic, which requires many iterations. The PI is to conservative, we need a more realistic conversion chart.
      4. Cheryl responded that the PI puts the patient in the ballpark for pain control and the physician should titrate up from there.
      5. That's the problem, Dr. Benjamin responded, PCPs do not know how to titrate.
      6. Cheryl indicated that this was an area for education and that Janssen had a major commitment to an education initiative for PCPs.
      7. Cheryl queried the physicians on what conversions they used.
      8. Some referenced the Breitbart paper on a dosing algorithm for transdermal fentanyl in cancer pain.
      9. Others said the 25 mcg patch ≠ 100 mg of morphine. It is more like 100 mg morphine = 200 meg patch.
      10. Allan and Milligan do not give their conversion calculations, this is a weakness.
      11. They do not state how long initial side effects lasted and this is also important information to know.”
    1. “What does drug abuse mean to you?
       1. Compulsive use; prescription drug abuse or diversion; drug seeking for a high; continued use of the drug despite harm to self or others; medicine not used for its intended purpose.
       2. Needed” o nosology of the negative outcomes from drug abuse. Is drug abuse a clinical outcome or a sociological phenomenon? (Katz)
       3. The DSM4 defines drug abuse as: craving, compulsive use, causing harm, so we don’t get to define the medical illness, but we have to define its cause and manage its sequellae. (King)
       4. Drug abuse is not in the realm of medication domain but refers to using narcotic drugs for uses other than pain relief, e.g., selling or recreational use. (Kerns)
       5. We need a new terminology, currently we use words derived from the field of drug and alcohol abuse. The use of these terms is not scientifically valid, these are not RX medication behaviors. (Gallagher)”
    2. “Should the abuse potential of Duragesic be discussed?
       1. "NO" - resounding and unanimous. It is bad for the LAO class and bad for patients and prescribers.
       2. Drug abusers will figure out how to abuse Duragesic once it is more available. Currently, it may be less abused and there is a dangerous narrow margin between a recreational dose to get high and a lethal dose. As market share goes up, so will abuse. Overpromising on the lack of abuseability is what got OxyContin in trouble. Duragesic should not repeat the same mistake.
       3. Dissenting opinion: comparing the patch to Percocet, the patch is less abuseable, less prone to self-administration behavior. For high risk patients, I give them the patch.
       4. It requires a lot of education for physicians and patients, you can't just say the patch is less abuseable.
       5. The relapse to drug abuse is a complex issue, involving many social and psychological factors. It would be better to define, measure and validate the risk factors for abuse.”
    3. Discussion of different messaging: “Conclusion: Do not include the abuse message. Do not sell opioids on the abuse issue.”
    4. “In their research, Drs. Passik and Portenoy have developed an assessment instrument, currently in validation trials, to monitor outcomes in chronic opioid therapy.”
    5. “Once Passik and Portenoy's assessment tool identifies that a patient is exhibiting aberrant behaviors, the clinician must determine why the behavior is occurring. The reason determines the clinical course of action. Aberrant behaviors may indicate:
       1. Addiction
       2. Pseudo-addiction
       3. Other psychiatric illness
       4. Family dysfunction
       5. Criminal intent”

1. The Prescription Opioid and Heroin Crisis
   - [https://www.industrydocuments.ucsf.edu/docs/jtgg0230](https://www.industrydocuments.ucsf.edu/drug/docs/#id=jtgg0230)
   - Author : Kolodny, Andrew; Courtwright, David T; Hwang, Catherine S; Kreiner, Peter; Eadie, John L; Clark, Thomas W; Alexander, G Caleb
   - Document Date : Unknown
   - Type : article; report
   - ID : jtgg0230 ( TID : npf71j00 )
   - ARK : ark:/88122/jtgg0230
   - Collection : Oklahoma Opioid Litigation Documents; Opioid Documents Collection
2. Medical Affairs Analgesia Medical Science Liaison Report
   - [https://www.industrydocuments.ucsf.edu/docs/zxgg0230](https://www.industrydocuments.ucsf.edu/drug/docs/#id=zxgg0230)
   - Author : Unknown
   - Document Date : Unknown
   - Type : report; table
   - ID : zxgg0230 ( TID : ejf71j00 )
   - ARK : ark:/88122/zxgg0230
   - Collection : Oklahoma Opioid Litigation Documents; Opioid Documents Collection
3. Risk Factors for Drug Dependence Among Out-Patients On Opioid Therapy In A Large US Health-Care System
   - [https://www.industrydocuments.ucsf.edu/docs/nhgg0230](https://www.industrydocuments.ucsf.edu/drug/docs/#id=nhgg0230)
   - Author : Boscarino, Joseph A; Rukstalis, Margaret; Hoffman, Stuart N; Hans, John J; Erlich, Porat M; Gerhard, Glenn S; Stewart, Walter F; Mount Sinai School of Medicine; TempleUniversity School of Medicine; Jo
   - Document Date : 2018 December 12
   - Type : report
   - ID : nhgg0230 ( TID : hif71j00 )
   - ARK : ark:/88122/nhgg0230
   - Collection : Oklahoma Opioid Litigation Documents; Opioid Documents Collection
   - Key Points:
     1. “Aims: Our study sought to assess the prevalence of and risk factors for opioid drug dependence among out-patients on long-term opioid therapy in a large health-care system.”
4. Addiction Rare in Patients Treated With Narcotics
   - [https://www.industrydocuments.ucsf.edu/docs/qkgg0230](https://www.industrydocuments.ucsf.edu/drug/docs/#id=qkgg0230)
   - Author : Boston University Medical Center; Porter, Jane; Jick, Hershel
   - Document Date : 2018 November 30
   - Type : article; publication
   - ID : qkgg0230 ( TID : gkf71j00 )
   - ARK : ark:/88122/qkgg0230
   - Collection : Oklahoma Opioid Litigation Documents; Opioid Documents Collection
   - Key Points:
     1. NEJM Letter to the Editor
5. Development of Dependence Following Treatment with Opioid Analgesics for Pain Relief a System Review
   - [https://www.industrydocuments.ucsf.edu/docs/nnfg0230](https://www.industrydocuments.ucsf.edu/drug/docs/#id=nnfg0230)
   - Author : Minozzi, Silver; Amato, Laura; Davoli, Marina
   - Document Date : 2012
   - Type : publication; article
   - ID : nnfg0230 ( TID : dcf71j00 )
   - ARK : ark:/88122/nnfg0230
   - Collection : Oklahoma Opioid Litigation Documents; Opioid Documents Collection
   - Key Points:
     1. “Aims: To assess the incidence or prevalence of opioid dependence syndrome in adults (with and without previous history of substance abuse) following treatment with opioid analgesics for pain relief.”
6. Johns Hopkins Anesthesiology Handbook
   - [https://www.industrydocuments.ucsf.edu/docs/tkfg0230](https://www.industrydocuments.ucsf.edu/drug/docs/#id=tkfg0230)
   - Author : Heitmiller, Eugene S; Schwengel, Deborah A; Mosby, Inc; Carinci, Adam J; Crooks, Matthew; Lenox, Brandon; Mazloomdoost, Danesh; Christo, Paul J
   - Document Date : 2010
   - Type : publication
   - ID : tkfg0230 ( TID : naf71j00 )
   - ARK : ark:/88122/tkfg0230
   - Collection : Oklahoma Opioid Litigation Documents; Opioid Documents Collection
   - Key Points:
     1. Clinical pain guideline
7. Opioid Use Behaviors, Mental Health and Pain-Development of a Typology of Chronic Pain Patients
   - [https://www.industrydocuments.ucsf.edu/docs/glfg0230](https://www.industrydocuments.ucsf.edu/drug/docs/#id=glfg0230)
   - Author : Banta-Green, Caleb J; Merrill, Joseph O; Doyle, Suzanne R; Boudreau, Denis M; Calsyn, Donald
   - Document Date : 2009 May 25
   - Type : publication; article
   - ID : glfg0230 ( TID : qaf71j00 )
   - ARK : ark:/88122/glfg0230
   - Collection : Oklahoma Opioid Litigation Documents; Opioid Documents Collection
   - Key Points:
     1. “Background: The intersection of pain, addiction and mental health has not been adequately described. We describe the roles of these three conditions in a chronic pain patient population using opioid analgesics. Aims were to improve our understanding of this population as well as to explore ways of identifying different types of patients.”
8. Substance Use Disorder in a Primary Care Sample Receiving Daily Opioid Therapy
   - [https://www.industrydocuments.ucsf.edu/docs/flfg0230](https://www.industrydocuments.ucsf.edu/drug/docs/#id=flfg0230)
   - Author : Fleming, Michael F; Balousek, Stacey L; Klessig, Cynthia L; Mundt, Marlon P; Brown, David D; University of Wisconsin; University of British Columbia; Elsevier; Journal of Pain, The
   - Document Date : 2007 July
   - Type : publication; article
   - ID : flfg0230 ( TID : paf71j00 )
   - ARK : ark:/88122/flfg0230
   - Collection : Oklahoma Opioid Litigation Documents; Opioid Documents Collection
   - Key Points:
     1. “Perspective: This study found that the frequency of opioid use disorders was 4 times higher in patients receiving opioid therapy compared with general population samples (3.8% vs 0.9%). The study also provides quantitative data linking aberrant drug behaviors to opioid use disorders.”
9. Directors Meeting ACTIQ
   - [https://www.industrydocuments.ucsf.edu/docs/yhgg0230](https://www.industrydocuments.ucsf.edu/drug/docs/#id=yhgg0230)
   - Author : Unknown
   - Document Date : 2004 December 02
   - Type : agenda; chart; graph; photograph; website
   - ID : yhgg0230 ( TID : iif71j00 )
   - ARK : ark:/88122/yhgg0230
   - Collection : Oklahoma Opioid Litigation Documents; Opioid Documents Collection
   - Key Points:

     2. “2004 External Challenges
        1. Increased media attention – Q2 2004
        2. Increased scrutiny from law enforcement and regulatory agencies
           1. Meetings with states AG and FDA
        3. Difficult process for promotional materials development given FDA review & comment
           1. Pulled ALL promo materials in August
        4. Growing ‘opiophobia’
           1. Concerns of abuse/addiction/diversion
           2. Concerns with increased prescriber scrutiny
        5. Increasing reimbursement barriers”


     8. “Emerging Solutions in Pain (ESP) is an ongoing initiative that is being developed by physicians for physicians, pharmacists and other healthcare professionals, to address some of the most critical issues in pain management today. These issues involve balancing the fundamental rights of patients and clinicians with the challenge of identifying patients who are at greater or lesser risk for opioid misuse and addiction, and with the challenges associated with the complex regulations involved in prescribing controlled substances. Through the expertise of a cadre of leading pain and addiction medicine experts, the ESP program will provide clinicians with guidance in the implementation of good practice management techniques, emphasizing favorable interaction with regulatory and law enforcement agencies, as well as, effective assessment, monitoring and documentation strategies, which will contribute to the overall goal of optimizing outcomes for their pain patients. ESP is a branded educational initiative supported by Cephalon, Inc and the Pain Franchise through an unrestricted educational grant.”
     9. “ESP Objectives

We've taught physicians the benefits of opioids and how to prescribe them, but neglected to adequately teach them about the risks. - Russell K. Portenoy

- - - 1. Broad-based
      2. Branded
      3. Awareness
      4. Education
      5. Improve Practice Management for Practitioners
      6. Minimize the risks of prescribing and dispensing opioids for physicians, pharmacists ana patients
      7. Ensure that pain is identified and treated appropriately
      8. Enhance Cephalon image as a Leader in Pain Management”
    1. “Project Overview
       1. Ongoing Initiative
       2. Previously named "Reduce the Risk"
          1. Originally focused on minimizing diversion and abuse
          2. Tool Kit to help assess and manage risk
       3. Emerging Solutions in Pain (ESP)
          1. Proposed as a broad-based "educational" initiative
          2. Supported through an unrestricted educational grant by Cephalon & the Pain Franchise
          3. Risk Minimization is an integral and core component of ESP”

1. Duragesic: Information on Opioid Dependence, Tolerance and Addiction
   - [https://www.industrydocuments.ucsf.edu/docs/ljgg0230](https://www.industrydocuments.ucsf.edu/drug/docs/#id=ljgg0230)
   - Author : Unknown
   - Document Date : 2003 June 16
   - Type : report
   - ID : ljgg0230 ( TID : ljf71j00 )
   - ARK : ark:/88122/ljgg0230
   - Collection : Oklahoma Opioid Litigation Documents; Opioid Documents Collection
   - Key Points:
     1. Definitions of terminology around addiction
     2. “This document is part of the DURAGESIC® (fentanyl transdermal system) press kit.”

150) Durogesic Slide Kid

Document Data

- **Author :** Donnelly, Julia
- **Document Date :** 2009 December 02
- **Type :** email
- **ID :** fqgg0230 ( TID : nnf71j00 )
- **ARK :** ark:/88122/fqgg0230
- **Collection :** Oklahoma Opioid Litigation Documents; Opioid Documents Collection

Document Notes

- Response by Bruce Moskovitsz, MD (Head of Analgesia, Jansenn) in response to Julia Donnelly’s Durogesic slides
  - Slide 3: Indicate potency is relative to morphine. Slide 40/41: Is this in line with recommendations to use Durogesic only in opioid-tolerant patients? It implies clinical use in na'ive patients. Slide 93: Postherpetic is misspelled. **Slide 117: We NEVER use the DAWN data to conclude low addiction potential. The data can be interpreted in other ways. Fentanyl is a highly addictive opioid; there should be no data presented to suggest otherwise.** Slide 145: Is "Opioid-naive patients should NOT be started on doses greater than 25 mcg/h" correct ex-US? Opioid-naïve is a contraindication in the US and I thought this recommendation was deleted outside the US. General: do you need to indicate for certain slides (e.g., neuropathic pain) that TDF Durogesic is not indicated for neuropathic pain?
- Could NOT find the slides that were referenced in this email; tried using “More Like This” Tool, searching by author, searching by Bruce Moskovitsz
- Increase in Unintentional Medication Overdose Deaths
  - [https://www.industrydocuments.ucsf.edu/docs/lpgg0230](https://www.industrydocuments.ucsf.edu/drug/docs/#id=lpgg0230)
  - Author : Piercefield, Emily; Archer, Pam; Kemp, Philip; Mallonee, Sue
  - Document Date : Unknown
  - Type : article; graph; report; report, scientific; table
  - ID : lpgg0230 ( TID : dnf71j00 )
  - ARK : ark:/88122/lpgg0230
  - Collection : Oklahoma Opioid Litigation Documents; Opioid Documents Collection
  - Key Points:
    - Funded by the Oklahoma State Department of Health and the Oklahoma Office of the Chief Medical Examiner
- Responsible Opioid Prescribing
  - [https://www.industrydocuments.ucsf.edu/docs/lrgg0230](https://www.industrydocuments.ucsf.edu/drug/docs/#id=lrgg0230)
  - Author : Colameco, Stephen
  - Document Date : Unknown
  - Type : presentation; slides; report
  - ID : lrgg0230 ( TID : jof71j00 )
  - ARK : ark:/88122/lrgg0230
  - Collection : Oklahoma Opioid Litigation Documents; Opioid Documents Collection
  - Key Points:
    - CME
    - Funded by:
      - Horizon Foundation of New Jersey
      - King Pharmaceuticals
      - PriCara (Johnson & Johnson)
      - Purdue Pharma
    - “Learning Objectives
      - Define addiction, pseudo-addiction, and dependence
      - Articulate common myths about opioid therapy that can interfere with one’s ability to manage pain effectively
      - Implement the function-based approach to pain management
      - Identify and address psychosocial issues associated with chronic pain and its management
      - Explain the basic components of an effective management plan for chronic pain:
        - Patient evaluation
        - Informed consent and agreement
        - Periodic review of treatment plan
        - Appropriate referrals and other components of care management
        - Documentation
        - Compliance with federal and state regulations
      - Understand key strategies to minimize the risk of drug misuse, abuse, and diversion
      - Reference the most recent consensus-based and evidence-based treatment recommendations: *The Clinical Guidelines for the Use of Chronic Opioid Therapy (COT) in Chronic Noncancer Pain American Pain Society/American Academy of Pain Medicine (2009)*
    - “Opioids: Myth vs. Fact
      - Myth: Opioid medications are always addicting

Fact: Many studies show that opioids are *rarely* addicting when used properly for the management of chronic pain.

- - - - “Myth: Opioids doses inevitably rise over time because of physical tolerance

Fact: Unless the underlying cause of pain gets worse (e.g. increasing spinal stenosis) patients are likely to remain on the same dose or only need small increases over time.”

- - - - “Myth: Opioids make it harder to function normally

Fact: When used correctly for appropriate conditions, opioids may make it *easier* for people to live normally

Neuropathic and non-neuropathic pain conditions appear in general to respond similarly COT”

- - - - “Myth: Patients with CNCP should all receive a long-acting opioid as part of COT

Fact: There is insufficient evidence to recommend short-acting versus long-acting opioids, or as-needed versus around-the-clock dosing of opioids.”

- - - - “Recommendation: Clinicians may consider COT for patients with CNCP and history of drug abuse, psychiatric issues, or serious aberrant drug-related behaviors only if they are able to implement more frequent and stringent monitoring parameters. In such situations, clinicians should strongly consider consultation with a mental health or addiction specialist.”
- Amended Expected Expert Opinion Testimony of Laurentius Marais
  - [https://www.industrydocuments.ucsf.edu/docs/nkfg0230](https://www.industrydocuments.ucsf.edu/drug/docs/#id=nkfg0230)
  - Author : Marais, M Laurentius
  - Document Date : Unknown
  - Type : notes; resume
  - ID : nkfg0230 ( TID : haf71j00 )
  - ARK : ark:/88122/nkfg0230
  - Collection : Oklahoma Opioid Litigation Documents; Opioid Documents Collection
  - Key Points:
    - “Dr. Marais expects to rebut evidence and/or opinions proffered by the State’s experts where such evidence and/or opinions are unfounded, erroneous, or unreliable, whether due to a failure to identify and employ relevant and reliable data sources, failure to employ reliable methodology, or for other reasons.”
    - “Dr. Marais may also opine from the perspective of his areas of expertise regarding the methods employed by the States experts where their testimony is based on or otherwise implicates statistics or applied mathematics.”
    - “Dr. Marais will assess from the perspective of his areas of expertise the methods employed by the State’s experts Jason Beaman and James L. Gibson for quantifying allegedly medically unnecessary opioid prescriptions, and will opine regarding the validity of these estimates.”
    - “Dr. Marais is expected to testify that the prescription sata relied upon by the State’s experts Jason Beaman and James L. Gibson, and the medical claims data provided by the State of Oklahoma, show that 0.7% of patients who filled opioid prescriptions solely for Duragesic were diagnosed with an opioid use disorder at any time after their first Duragesic prescription. For comparison, 4.4% of patients who never received a Janssen opioid, but did receive another manufacturer’s opioid, were diagnosed with an opioid use disorder at some time after their first opioid prescription.”
    - “Dr. Marais is expected to testify that the prescription data relied upon by the State’s experts Jason Beaman and James L. Gibson, and the medical claims data provided by the State of Oklahoma, shows that 97.0% of patients receiving Duragesic were *not* diagnosed with an opioid use disorder within 12 months of their Duragesic prescriptions. The same holds for 96.3% of patients receiving any pertinent Janssen opioid.”
    - 33 previous expert testimonies in the previous 4 years
- Tapentadol Professional Education Message Platform
  - [https://www.industrydocuments.ucsf.edu/docs/spgg0230](https://www.industrydocuments.ucsf.edu/drug/docs/#id=spgg0230)
  - Author : Unknown
  - Document Date : Unknown
  - Type : report; presentation; slides
  - ID : spgg0230 ( TID : knf71j00 )
  - ARK : ark:/88122/spgg0230
  - Collection : Oklahoma Opioid Litigation Documents; Opioid Documents Collection
  - Key Points:
    - “Unbranded Message Platform
      - Acute moderate-to-severe pain is critically undermanaged
      - Tapentadol is a unique and novel centrally-acting analgesic with a dual mechanism of action
      - The efficacy safety and tolerability of tapentadol have been established in clinical trials
      - Two formulations of tapentadol will be available immediate0release and extended-release
      - Tapentadol as first-choice therapy for acute and chronic moderate-to-severe pain”
    - LARGE number of references
- Long-Lasting Efficacy
  - [https://www.industrydocuments.ucsf.edu/docs/ysgg0230](https://www.industrydocuments.ucsf.edu/drug/docs/#id=ysgg0230)
  - Author : Janssen
  - Document Date : Unknown {2004}
  - Type : graph; photograph; publication; table
  - ID : ysgg0230 ( TID : cpf71j00 )
  - ARK : ark:/88122/ysgg0230
  - Collection : Oklahoma Opioid Litigation Documents; Opioid Documents Collection
  - Key Points:
    - Duragesic flyer “Information for the Pharmacist”
    - “Uninterrupted” brochure

190) March 2008 Agenda

Document Data

- Author : Pharmacy Board; Lepore, Christopher; Johnson and Johnson; Moskovitz, Bruce L; Ortho-McNeil Janssen Scientific Affairs
- Document Date : 2008 February 20
- Type : email
- ID : mhgg0230 ( TID : gif71j00 )
- ARK : ark:/88122/mhgg0230
- Collection : Oklahoma Opioid Litigation Documents; Opioid Documents Collection

Document Notes

- Email from Chris Lepore, Director of State Gov Affairs @ J&J
  - I just received the NV Board of Pharmacy's Agenda for their meeting March 5 & 6. They plan to discuss the scheduling of Tramadol and may take action. This is the first time the issue has been brought up in Nevada
- Response from Bruce Moskovitz, Therapeutic Area Head @ Janssen
  - Is there a "swat" team that we can put together with Ted Cicero and whatever resource at RADARS (perhaps under a retainer system) that would allow them to mobilize as soon as a threat is detected, with minimal oversight on our part? It seems to me this is how we routinely respond anyway, except that we always start from ground zero.
  - **More evidence that the authors behind RADAR used their system to create data that was favorable to the marketing of J&J drugs**

191) NEBRASKA – TRAMADOL LEGISLATION

Document Data

- Author : Gary, Vorsanger J; Ortho-McNeil Janssen Scientific Affairs, LLC; Margaret, Quinn
- Document Date : 2008 February 18
- Type : email
- ID : qggg0230 ( TID : uhf71j00 )
- ARK : ark:/88122/qggg0230
- Collection : Oklahoma Opioid Litigation Documents; Opioid Documents Collection

Document Notes

- Email from Gary Vorsanger, PHd, MD, Senior Director of Clinical Development @ Janssen
  - Yes, RADARS is a network that provides information on abuse and diversion of prescription pain medications on a subscription basis to participating pharmaceutical companies about their own products. We purchase data from RADARS for Duragesic and our tramadol containing products. We would not, for example, be able to provide data on branded prescription pain medications such as OxyContin.
- Hospital Sales Force Cycle III Presentation
  - [https://www.industrydocuments.ucsf.edu/docs/gtgg0230](https://www.industrydocuments.ucsf.edu/drug/docs/#id=gtgg0230)
  - Author : Unknown
  - Document Date : 2002 September 17
  - Type : chart; graph; photograph; publication
  - ID : gtgg0230 ( TID : kpf71j00 )
  - ARK : ark:/88122/gtgg0230
  - Collection : Oklahoma Opioid Litigation Documents; Opioid Documents Collection
- Predicting Aberrant Drug Behavior
  - [https://www.industrydocuments.ucsf.edu/docs/kxgg0230](https://www.industrydocuments.ucsf.edu/drug/docs/#id=kxgg0230)
  - Author : Gilson, Aaron M; Ryan, Karen M; Joranson, David E; Dahl, June L; University of Wisconsin-Madison Comprehensive Cancer Center; Journal of Pain and Symptom Management
  - Document Date : 2004 August 02
  - Type : publication; article
  - ID : kxgg0230 ( TID : uif71j00 )
  - ARK : ark:/88122/kxgg0230
  - Collection : Oklahoma Opioid Litigation Documents; Opioid Documents Collection
- Barriers to Optimal Pain Management
  - [https://www.industrydocuments.ucsf.edu/docs/rxgg0230](https://www.industrydocuments.ucsf.edu/drug/docs/#id=rxgg0230)
  - Author : Joranson and Gilson; American Pain Society
  - Document Date : 2008 March 11
  - Type : article; diagram; graph; publication
  - ID : rxgg0230 ( TID : bjf71j00 )
  - ARK : ark:/88122/rxgg0230
  - Collection : Oklahoma Opioid Litigation Documents; Opioid Documents Collection
- Demonstrated Effectiveness in Chronic Back Pain with Additional Patient Benefits
  - [https://www.industrydocuments.ucsf.edu/docs/zsgg0230](https://www.industrydocuments.ucsf.edu/drug/docs/#id=zsgg0230)
  - Author : Unknown
  - Document Date : Unknown
  - Type : article; graph; report
  - ID : zsgg0230 ( TID : ipf71j00 )
  - ARK : ark:/88122/zsgg0230
  - Collection : Oklahoma Opioid Litigation Documents; Opioid Documents Collection
  - Key Points:
    - Marketing handout for Duragesic
    - “1,360 loaves…and counting”

266) Trends in Medical Use and Opioid Analgesics

Document Data

- Author : Joranson, David E; Ryan, Karen M; Gilson, Aaron M; Dahl, June L; JAMA; American Medical Association; Brittany Kellogg
- Document Date : 2002 April 05
- Type : publication; article
- ID : jxgg0230 ( TID : tif71j00 )
- ARK : ark:/88122/jxgg0230
- Collection : Oklahoma Opioid Litigation Documents; Opioid Documents Collection

Document Notes

- PAPER: **Trends in Medical Use and Abuse of Opioid Analgesics (JAMA, 2002)**
- CONCLUSION: The trend of increasing medical use of opioid analgesics to treat pain does not appear to contribute to increases in the health consequences of opioid analgesic abuse.
- AUTHORS
  - David Joranson, MSSW
  - Karen Ryan, MA
  - Aaron Gilson, PhD
  - June Dahl, PhD
- Financial contributions
  - JORANSON –Purdue Pharma, Janssen Pharma
  - DAHL – Purdue Pharma, Knoll Pharma
- More information on Joranson and Dahl
  - **Joranson and Dahl are part of University of Wisconsin’s Pain Group**
  - UW Pain Group has received $1.6 million from Purdue from 1999-2010 ([LINK](http://www.salem-news.com/articles/april222011/pain-studies-ms.php))
  - Not only has the UW Pain Group hauled in pharmaceutical industry money, but on more than a dozen occasions over about 10 years Joranson and Gilson were paid by drugmakers or organizations connected with them to give talks, author papers, or to work in other capacities. ([abcnews](https://abcnews.go.com/Health/academics-profit-making-case-opioid-painkillers/story?id=13284493))
- **PAPER USES DAWN DATA**
  - SEE #272 -- Internal email @ Janssen =
    - **As I mentioned this morning at Dura Team Mtg. I have grave concerns about the acceptability of the DAWN data**
    - The denominator in the DAWN abuse statistics
    - The docs saw DAWN as representative of an entirely different and unrelated population—street dmg users as opposed to the patients - a very different group, they contend
    - They felt that, if DURAGESIC were as widely distributed (as available) as OxyContin, it would have the same uptake in the abuse community and would lead Janssen down the path to problems followed by Purdue,

271) Duragesic Sales Force Update

Document Data

- Author : Unknown
- Document Date : 2002
- Type : report; report, marketing
- ID : qhgg0230 ( TID : kif71j00 )
- ARK : ark:/88122/qhgg0230
- Collection : Oklahoma Opioid Litigation Documents; Opioid Documents Collection

Document Notes

- Coming off a record-breaking year of $543 million in 2001, the bar has been raised for DURAGESIC® in 2002 to $692 million in sales, a 28% increase!
  - You are our primary sales force that drives nearly 75% of the business through **Pain Specialist and Primary Care physicians**
- Your commitment to sell DURAGESIC by being **ethically aggressive** will continue to position the brand for double digit sales growth throughout 2002 and beyond.
- Patient Target: **Expand DURAGESIC use in non-malignant pain**
  - Chronic, non-malignant pain states, such as lower back pain, represent considerable growth opportunities for the brand; it is imperative that we accelerate our growth in these areas.
  - Our objective is to convince physicians that DURAGESIC is effective and safe to use in moderate to severe chronic pain such as **back pain and degenerative joint disease** like osteoarthritis.
- Core Message: Life, Uninterrupted
  - Research identified the opportunity to place even greater emphasis on patient functionality as the primary goal of treatment, supported by a strong message on pain relief efficacy. Specifically, research shows: 1) **functionality is a key driver of brand selection**, 2) functionality is the end-benefit of physician treatment goals and 3) no brand currently owns functionality.
- Sales Material
  - Milligan Reprint Carrier
    - Reprint carrier for chronic non-malignant pain discussing long-term observation of DURAGESIC patients. Study supports that long-term treatment of chronic pain is effective and well tolerated. A significant improvement was shown in SF36 functionality assessment scores and patients' demonstrated preference for DURAGESIC over previous opioid medication.
  - National Pain Education Council NPEC Invitation
    - National Pain Education Council is funded by an educational grant from Janssen. Invitation to participate in a multimedia CME program for physicians and other medical professionals on the appropriate opioid pharmacotherapy for chronic pain management
  - **Dr Passik Substance Abuse Issue Teletopics**
    - CME resource on the subject of Substance Abuse Issues in Chronic Pain presented by Dr. Steven Passik. This interactive teleconference workshop identifies aberrant behaviors that may be indicative of substance abuse problems and issues with opioids in the treatment of chronic pain and impact of drug choice and use on minimizing potential abuse problems
    - CONTROL+F “PASSIK” -- lots of money from pharma!!

272) Email from Christine DeVries Regarding the Message Refinement

Document Data

- Author : DeVries, Christine
- Document Date : 2001 December 03
- Type : email
- ID : qpgg0230 ( TID : inf71j00 )
- ARK : ark:/88122/qpgg0230
- Collection : Oklahoma Opioid Litigation Documents; Opioid Documents Collection

Document Notes

- As I mentioned this morning at Dura Team Mtg. I have grave concerns about tlie acceptability of the DAWN data
  - The denominator in the DAWN abuse statistics
  - The docs saw DAWN as representative of an entirely different and unrelated population—street dmg users as opposed to the patients - a very different group, they contend
  - They felt that, if DURAGESIC were as widely distributed (as available) as OxyContin, it would have the same uptake in the abuse community and would lead Janssen down the path to problems followed by Purdue,
- **NEED TO LOOK INTO DAWN!!!!!**

286) Review of Trends in Medical Use and Abuse of Opioid Analgesics

Document Data

Author : Janssen; Update Product News

Document Date : 2000 June 12

Type : memo

ID : nxgg0230 ( TID : xif71j00 )

ARK : ark:/88122/nxgg0230

Collection : Oklahoma Opioid Litigation Documents; Opioid Documents Collection

Document Notes

- Email from Janssen Sales Training to Field Sales Force, Eldercare Saresforce
- Attached for your review is an article published in the April 5th issue of the Journal of American Medical Association (JAMA) which discusses the use and abuse of opioid analgesics from .1990-1996. **The authors found that the present trend of increasing medical use of opioid analgesics to treat pain does not appear to contribute to opioid analgesic abuse.**
  - This article should be helpful in discussing these issues with your physicians and should enhance your understanding of the potential for abuse or misuse in the context of pain management.
  - The authors write that the present trend of increasing medical use of opioid analgesics to treat pain does not appear to be contributing to increases in the health consequences of opioid analgesic abuse.

Study limitations = The DAWN system may underestimate the extent of the drug abuse problem, because it measures only those episodes of drug abuse that result in an admission to an Emergency Department

1. The Economic Impact of Opioid Use in the Management of Chronic Nonmalignant Pain
   - [https://www.industrydocuments.ucsf.edu/docs/gnfg0230](https://www.industrydocuments.ucsf.edu/drug/docs/#id=gnfg0230)
   - Author : Lipman, Arthur; Webster, Lynn
   - Document Date : 2015 October
   - Type : publication; article
   - ID : gnfg0230 ( TID : wbf71j00 )
   - ARK : ark:/88122/gnfg0230
   - Collection : Oklahoma Opioid Litigation Documents; Opioid Documents Collection
   - Key Points:
     1. “Webster is a consultant to AstraZeneca, Cara Therapeutics, CVS Caremark, Mallinckrodt Pharmaceuticals, Marathon Pharmaceuticals, Merck, and Zogenix. He is a participant in advisory boards for Charleston Labs, Collegium Pharmaceuticals, Egalet, Inspirion Pharmaceuticals, Kaleo, Orexo, Pfizer, Signature Therapeutics, and Trevena. His is a participant in advisory boards and a consultant to Insys Therapeutics and Proove Biosciences.”
2. Risk Management for Tapentadol
   - [https://www.industrydocuments.ucsf.edu/docs/jrgg0230](https://www.industrydocuments.ucsf.edu/drug/docs/#id=jrgg0230)
   - Author : Unknown
   - Document Date : Unknown
   - Type : report; report, scientific
   - ID : jrgg0230 ( TID : hof71j00 )
   - ARK : ark:/88122/jrgg0230
   - Collection : Oklahoma Opioid Litigation Documents; Opioid Documents Collection
   - Key Points:
     1. “For internal training purposes only”
     2. “Learning Objectives

Upon completion of this chapter you will be able to:

- - - 1. Identify the potential risks associated with opioid therapy.
      2. Explain why respiratory depression is one of the most serious risks of opioid therapy.
      3. Differentiate between misuse, abuse, diversion, tolerance, and dependence.
      4. Discuss the rationale behind the FDA’s class-wide Risk Evaluation and Mitigation Strategies (REMS) for opioid medications.
      5. Discuss why the potential for drug-seeking behavior should be assessed prior to and during opioid therapy.
      6. Describe assessment tools that are available for opioid risk assessment.
      7. Describe the components of FDA-approved REMS for OxyContin®, Embeda®, and Exalgo®.
      8. Discuss the importance or proper patient and dose selection for opioid medications.”

1. Cumulative Review of Iatrogenic Addiction Associated with the Use of Transdermal Duragesic (fentanyl) Patch
   - Author : Sanderson-Bongiovanni, Dawn
   - Document Date : 2006 September 06
   - Type : report; report, scientific
   - ID : xlfg0230 ( TID : saf71j00 )
   - ARK : ark:/88122/xlfg0230
   - Collection : Oklahoma Opioid Litigation Documents; Opioid Documents Collection
   - Key Points:
     1. “Conclusion

A review of 103 cases that reported drug dependence associated with chronic use of the transdermal fentanyl patch indicates that the risk of iatrogenic addiction is very rare. The CCDS adequately communicates the risk associated with this particular product.”

- - 1. Cites Webster paper
    2. “The information in this document contains trade secrets and commercial information that are privileged or confidential and may not be disclosed unless such disclosure is required by applicable law or regulations. In any event, the persons to whom the information is disclosed must be informed that the information is privileged or confidential and may not be further disclosed by them. These restrictions on disclosure will apply equally to all future information supplied to you, which is indicated as privileged or confidential.”
    3. Johnson & Johnson Pharmaceutical Research & Development, L.L.C. Benefit Risk Management

1. Actiq Approved
   - [https://www.industrydocuments.ucsf.edu/docs/ztgg0230](https://www.industrydocuments.ucsf.edu/drug/docs/#id=ztgg0230)
   - Author : Unknown
   - Document Date : Unknown
   - Type : table
   - ID : ztgg0230 ( TID : ypf71j00 )
   - ARK : ark:/88122/ztgg0230
   - Collection : Oklahoma Opioid Litigation Documents; Opioid Documents Collection
   - Key Points:
     1. Actiq speaker list
2. Article Summary – Randomised crossover trial of transdermal fentanyl and sustained release oral morphine for treating chronic non-cancer pain
   - [https://www.industrydocuments.ucsf.edu/docs/psgg0230](https://www.industrydocuments.ucsf.edu/drug/docs/#id=psgg0230)
   - Author : Medical Services, Sales Training Duragesic Brand Team
   - Document Date : 2001 July 19
   - Type : memo; report
   - ID : psgg0230 ( TID : dpf71j00 )
   - ARK : ark:/88122/psgg0230
   - Collection : Oklahoma Opioid Litigation Documents; Opioid Documents Collection
   - Key Points:
     1. Email to sales force regarding Allan study
     2. “This PNU provides an overview of the study and comments on key points *(in italics)* whenever possible.”
     3. “*There were no U.S. sites in this trial. It is important to note that the European perspective on the treatment of nonmalignant pain with opioids is very different than in the US. While there is no consensus in the U.S., physicians here are more likely to prescribe opioids for nonmalignant pain than physicians in Europe.”*
     4. Responses to letters to the editor critical of study design including from McQuay
3. Comment on Draft Duragesic Slide Deck
   - [https://www.industrydocuments.ucsf.edu/docs/msgg0230](https://www.industrydocuments.ucsf.edu/drug/docs/#id=msgg0230)
   - Author : Brett, Vince
   - Document Date : 2007 November 15
   - Type : publication; report, scientific; table; website
   - ID : msgg0230 ( TID : apf71j00 )
   - ARK : ark:/88122/msgg0230
   - Collection : Oklahoma Opioid Litigation Documents; Opioid Documents Collection
   - Key Points:
     1. “Consider deleting the Milligan, Ahmedzai, Agarwal, and Sellers papers from this slide deck. As published, these ‘studies’ are flawed, convoluted, and/or incomplete. There are numerous errors, inconsistencies, gaps, omissions, misrepresentations, and conclusions that are not supported by statistical significance.”
     2. “The slide deck failed to present the primary endpoint results for some of these studies.”
     3. Milligan study
        1. “Overall criticism of paper: This was the worst of the studies. Milligan et al is loaded with inconsistencies, errors, and omissions of data, which calls into question the integrity of the results. Consider deleting this study from slide deck.”
4. Duragesic Warning Letter Response
   - [https://www.industrydocuments.ucsf.edu/docs/tpfg0230](https://www.industrydocuments.ucsf.edu/drug/docs/#id=tpfg0230)
   - Author : Burrus, James K; Johnson and Johnson Pharmaceutical Research and Development
   - Document Date : 2004 September 17
   - Type : bibliography; letter; memo
   - ID : tpfg0230 ( TID : pdf71j00 )
   - ARK : ark:/88122/tpfg0230
   - Collection : Oklahoma Opioid Litigation Documents; Opioid Documents Collection
   - Key Points:
     1. Fax to Thomas W. Abrams, RPh, MBA, Director of Division of Drug Marketing, Advertising and Communications (DDMAC)
     2. “Janssen and J&JPRD respectfully disagree with DDMAC’s position that the professional file card (DR-850) for Duragesic makes false of misleading statements about the abuse potential, effectiveness and other risks of the drug. Janssen and J&J PRD also respectfully disagree with DDMAC that by suggesting that Duragesic has a low potential for abuse compared to other opioid products the file card encourages the unsafe use of the drug in such a way that could potentially result in serious of life-threatening hypoventilation.”
     3. Low Abuse Potential
        1. Zacny et al (2003)
        2. Mironer et al (2000)
        3. Joransen et al (2000)
        4. Coleman et al (Manuscript)
5. Warning Letter Regarding NDA #19-813 Duragesic (fentanyl transdermal system)
   - [https://www.industrydocuments.ucsf.edu/docs/hggg0230](https://www.industrydocuments.ucsf.edu/drug/docs/#id=hggg0230)
   - Author : Food and Drug Administration; Department of Health and Human Services; Abrams, Thomas W
   - Document Date : 2004 September 02
   - Type : letter
   - ID : hggg0230 ( TID : lhf71j00 )
   - ARK : ark:/88122/hggg0230
   - Collection : Oklahoma Opioid Litigation Documents; Opioid Documents Collection
   - Key Points:
     1. “The Division of Drug Marketing, Advertising, and Communications (DDMAC) has reviewed a professional file card (DR-850) for Duragesic® (fentanyl transdermal system) submitted by Janssen Pharmaceutica, Inc. (Janssen) under cover of Form FDA 2253. The file card makes false or misleading claims about the abuse potential and other risks of the drug, and includes unsubstantiated effectiveness claims for Duragesic. The file card thus misbrands the drug under Section 502(a) of the Federal Food, Drug, and Cosmetic Act (Act) 21 U.S.C. 352(a). By suggesting that Duragesic has a lower potential for abuse compared to other opioid products, the file card could encourage the unsafe use of the drug, potentially resulting in serious or life-threatening hypoventilation.”
     2. “This is false or misleading for two reasons. First, we are not aware of substantial evidence or substantial clinical experience to support this comparative claim. The DAWN data cannot provide the basis for a valid comparison among these products. As you know, DAWN is not a clinical trial database. Instead, it is a national public health surveillance system that monitors drug-related emergency department visits and deaths. If you have other data demonstrating that Duragesic is less abused, please submit them.

Second, Duragesic is not as widely prescribed as other opioid products. As a result, the relatively lower number of mentions could be attributed to the lower frequency of use, and not to a lower incidence of abuse. The file card fails to disclose this information.”

- - 1. “The file card states, on page four, "Demonstrated effectiveness in chronic back pain with additional patient benefits." The referenced study,' conducted by Simpson et al., is inadequate to support this claim, because it was an open-label, single-arm trial with no control group. We are not aware of substantial evidence or substantial clinical experience to support this claim.”
    2. “On pages 6 and 7, the file card includes the claims, "Long-term effects: 12-month open-label study," "Significant improvement in physical functioning summary score," and "Significant improvement in social functioning," along with figures illustrating these claims. To support these claims, the file card cites a study2 conducted by Milligan et al. This open-label, uncontrolled study is not adequate in design to show an analgesic effect. The data from this study are not substantial evidence or substantial clinical experience to support such outcomes claims. We are not aware of substantial evidence or substantial clinical experience to support these claims.”
    3. “On pages 8 and 9, the file card includes the claims, "Improved patient outcomes: Open-label, crossover comparison study," "Significant improvement in physical functioning summary score," and "Significant improvement in social functioning," along with figures comparing data for Duragesic and sustained release oral morphine. To support these claims, the file card cites the study3 conducted by Allan et al.. An open-label study cannot minimize bias in the reporting of subjective response in the SF-36, a general healthcare questionnaire. It is therefore not sufficient to support the cited claims. We are not aware of substantial evidence or substantial clinical experience to support these claims.”
    4. “DDMAC requests that Janssen immediately cease the dissemination of promotional materials for Duragesic the same as or similar to those described above.”

1. Article Summary Evaluation of Long-Term Efficacy and Safety of Transdermal Fentanyl in the Treatment of Chronic Noncancer Pain
   - [https://www.industrydocuments.ucsf.edu/docs/ssgg0230](https://www.industrydocuments.ucsf.edu/drug/docs/#id=ssgg0230)
   - Author : Janssen Pharmaceutical, Inc
   - Document Date : 2001 September 21
   - Type : memo
   - ID : ssgg0230 ( TID : gpf71j00 )
   - ARK : ark:/88122/ssgg0230
   - Collection : Oklahoma Opioid Litigation Documents; Opioid Documents Collection
   - Key Points:
     1. PNU on Milligan study
     2. *“Although there is the potential for bias with a open-label study, this design is more like a real world situation.”*
     3. “The majority of patients (95%) were receiving concomitant medications, such as long-term steroidal or nonsteroidal anti-inflammatory agents, antiepileptics, laxatives, antidepressants, and other psychtropic drugs.” “*Since this study population included patients with concomitant comorbidities and medications, it is a good representation of a real world setting.”*
     4. *“The authors stated that TDF provided stable, sustained, long-term pain control. Approximately 1/3 of the study population did not ‘respond’ to TDF. This coincides with Perry Fine’s comments (see editorial) that a process of trial and error is often needed to achieve adequate pain management.”*
     5. *“A possible explanation for the low rate of global efficacy is that, unlike pain control assessment, the results for the global efficacy measurement did not include a ‘moderate’ rating.”*
     6. *“The patients that withdrew during the first months of the trial did so as a result of an AE or insufficient response. Patients rarely withdrew from the study die to an AE or insufficient response after 6 months into the trial. This result may indicate that within the first months of the trial, most of the withdrawals secondary to insufficient response may be related to improper titration. Withdrawals secondary to AEs may be due to lack of tolerability to the transient side effects of TDF.*

*The authors stated that the incidence of Aes and the rate of withdrawal from the trial are relatively high but neith unusual nor unexpected considering the baseline clinical status of the study population. There were multiple comorbidities, as evidenced by their concomitant diagnoses and concomitant medications. In addition, these patients had low QoL scores at baseline.”*

**GENERAL ACADEMIC MALFEASANCE**

10) Payment History of J&J or Janssen to Specific Individuals

- Document Data
  - **Author :** Janssen
  - **Document Date :** 2019 January 25
  - **Type :** report; legal; table
  - **ID :** rngg0230 ( TID : dmf71j00 )
  - **ARK :** ark:/88122/rngg0230
  - **Collection :** Oklahoma Opioid Litigation Documents; Opioid Documents Collection
- Document Notes
  - Dr. Charles Argoff – Psychiatry & Neurology & Chronic Pain
    - Activity = Advisor, consultant
    - Contributor to *Prescribe Responsibly*
    - Payment = $37000
  - Dr. C Keith – Anesthesiology
    - Activity = Advisor, consultant
    - Contributor to *Prescribe Responsibly*
    - Payment = $13000
  - Dr. F Pine – Anesthesiology & Chronic Pain
    - Activity = Advisor, consultant
    - Contributor to *National Pain Education Council (NPEC)*
    - Payment = $35000
  - Dr F Scott – Anesthesiology, Hospice, Palliative Medicine
    - Activity = Advisor, consultant
    - Contributor to *Let’s Talk Pain*
    - Payment = $2000
  - Dr N Katz – Anesthesia
    - Activity = Advisor, consultant
    - Payment = $5000
  - Dr B McCarberg – Pain Management
    - Activity = Advisor, consultant
    - Contributor to *Let’s Talk Pain*, patient counseling education video, NPEC, many others things for Janssen
    - Payment = $27,000

25) External Project Cost

Document Data

**Author :** Cephalon, Inc

**Document Date :** 2018 November 07

**Type :** report, financial

**ID : l**ggg0230 ( TID : phf71j00 )

**ARK :** ark:/88122/lggg0230

**Collection :** Oklahoma Opioid Litigation Documents; Opioid Documents Collection

Document Notes

- Tables that document exactly how much $$$ was given to various organizations and why the money was given from Cephalon, Inc!! Lots of juicy stuff
  - NOTE: Cephalon is a subsidiary of Teva Pharmaceuticals
- Sample of some of the more interesting payments:
  - Booth space – american academy of pain, american pain society, AMCP, american society of health system pharmacists, ASCO, oncology nursing society
  - Contribution – American Pain Foundation, American Academy of Pain Mngmt, American Pain Society, American Chronic Pain Assoc, DAMMADD, National Coalation for Cancer, National Pain Foundation
  - Journal Ad – ELsevier Science, **Intellisphere – MEDICAL PUBLISHER!**

81) 2013 National Advocacy Business Planning

Document Data

- Author : Johnson and Johnson; Janssen Pharmaceutical
- Document Date : 2012 June 29
- Type : proposal
- ID : zggg0230 ( TID : yhf71j00 )
- ARK : ark:/88122/zggg0230

Document Notes

- Business planning from Janssen (PAIN FRANCHISE) June 29, 2012
- Key Questions = How to leverage sales of Nucynta??
- 2013 PAIN Advocacy Strategy
  - Engage partners to embrace the IOM report-national/state implications advocate for and act collectively to actualize the recommendations
  - **Influence agencies that impact policy and quality to maintain or improve access**
- Barriers to care
  - FOCUS: **Engage with advocacy partners** at the national level for greater impact and alignment. Support models for collaboration and synergies that can then be applied at a regional level
  - Chronic pain can and should be thought of as a disease in and of itself.
  - Public health and community-based approaches are required to **address the under-treatment of chronic pain**
- Advocacy/Policy Focus
  - Provide state and federal legislators and other regulatory groups with access to objective materials to assist them in making public policy.
  - Collaborate with the **Pain Care Forum (PCF)** on policy issues and common strategies with key decision makers; such as HHS, Surgeon General's Office, CDC, state and federal legislators and regulators. •
  - Collaborate with State Pain Policy Action Coalition (SPPAC), a newly formed organization made up of pain focused organizations that will pro-actively inform and influence state policy . •
  - **Sponsor Public Awareness campaigns targeted at preventing chronic pain and misuse of prescription pain medications. •**
  - **Sponsor disease awareness to promote balanced and effective pain management. •**
  - Support collaboration between Medicine and Law Enforcement to prevent the "chilling effect".
    - [Chilling effect](https://oxfordmedicine.com/view/10.1093/med/9780190659721.001.0001/med-9780190659721-chapter-10) ([link](https://oxfordmedicine.com/view/10.1093/med/9780190659721.001.0001/med-9780190659721-chapter-10)) = An overzealous Drug Enforcement Administration is sometimes prosecuting the wrong physicians, thus creating a chilling effect in the medical community with regard to opioid prescription and making it harder for people in pain to get the help they need
  - Support effective Prescription Monitoring Programs (PMPs) that provide prescribing healthcare professionals with "real time access" and improve patient care.

85) RE: Conference Call Availability FW

Document Data

- **Author :** Dart, Richard; Vorsanger, Gary J; Janssen Scientific Affairs, LLC; Cecero, Theodore; Washington University
- **Document Date :** 2012 February 11
- **Type :** email
- **ID :** xhhg0230 ( TID : yrf71j00 )
- **ARK :** ark:/88122/xhhg0230
- **Collection :** Oklahoma Opioid Litigation Documents; Opioid Documents Collection

Document Notes:

NOTE: To understand this document, must first read about doc ffgg0230

- **What is RADARS**: About RADARS® System ([LINK](https://www.prnewswire.com/news-releases/opioid-abuse-on-the-decline-according-to-radars-system-300020983.html))
  The RADARS® System is a non-profit public health prescription drug abuse, misuse, and diversion surveillance system that collects timely product and geographically specific data. The RADARS® System has grown since its inception in 2001 and is made up of seven primary programs each designed to provide different but complementary perspectives on prescription drug abuse in the United States.
- Principal Investigators
  - **Hilary Surratt**
  - **Theodore Cicero**
  - **Richard Dart**
  - **Andrew Rosenblum**
- Target Opioid = Tapentadol
- Objectives:
  - a) To identify abuse, misuse and diversion rates for oxycodone, hydrocodone, morphine, hydromorphone, fentanyl, buprenorphine, methadone, tapentadol and tramadol. Rates will be calculated by population (per 100,000 population) and by Unique Recipients of Dispensed Drugs (per 1,000 URDD)
  - b) To identify "signal sites" for Nucynta as well as the comparator drugs. A "signal site" is defined as any 3-digit ZIP code which meets or exceeds its signal threshold for any given drug in any given quarter. Signal thresholds are defined as the following:
    - Drug Diversion Program: 5 cases per 100,000 population
    - Poison Center, Survey of Key Informants' Patients and Opioid Treatment Programs: 2 cases per 100,000 population
- Nucynta Trends in Rates per 100,000 Population
  - Summary = not being abused
- Nucynta Trends in rates per 1,000 URDD (Unique recipients of dispended drug)
  - Summary = not being abused

NOTES on document xhhg0230

- Email from Cicero on first draft of RADAR
  - **1) First, when URDDs are used tapentadol is abused at rates that are equivalent to other schedule II and III drugs. Is that really the message we want to impart?** It is an artifact of low sales such that 2 or 3 cases of abuse become huge. Until it is better established as a product I think it is misleading to publish rates using URDDs. The explanation dealing with the discrepancy is weak and unconvincing
  - 3) Third, I understand why the timing for this article, but I predict what will be seen here is exactly what that brilliant scientist Cicero (*he’s talking about himself in third person???*) showed with tramadol. An initial period of experimentation and then a gradual decline to very low steady state levels. **Thus, there was a simple explanation for early results suggesting a lot of abuse.** I would at least mention that in this paper.
  - 4) Fourth, I worry about the duel mechanism explanation for dependence as I have for years about its role in tramadol misuse. Specifically you have to argue that tramadol /tapentadol produce euphoria whereas the norepi reuptake acts as an anti-euphoric agent. I don't know of any evidence to support that, but **I can** **live with the people at J&J and Edgar Adams believing this to be true**. At least the paper does say it is a hypothesis, but it is weak in my view.
  - 5) Fifth, the first sentence of the discussion is at best misleading. **It reads "Results from the 4 RADARS System show...tapentadol IR has low rates of abuse and diversion compared with oxycodone and hydrocodone". Not true if you use the URDDs**. Rather tapentadol has rates similar to oxycodone and hydrocodone if URDDs are used. The remainder of the paragraph tries very unconvincingly to explain this away
- Email from Richard Dart in response to critism from Cicero
  - 1) Answer: Ted, your message portrays the results as an aberration caused by low volume of cases. I don't think that is true. **The URDD are much higher than you allude**. Perhaps you are referring to a single 3DZ, but we are using national data. It looks to me that tapentadol is exactly where we think it should be....I realize this varies by the program, but the results are evident in the figures. In SKIP and OTP, **tapentadol is lower than oxycodone, about the same as hydrocodone and higher that tramadol. Thus, among abusers, there is some attractiveness for tapentadol**
    - Based on the population denominator alone, once would disregard tapentadol as a drug of abuse. When both denominators, however, o**ne can see that it is indeed abused** albeit not to the degree of oxycodone and hydrocodone.
  - 3) Interestingly, we are writing a separate paper using College Student data and **there was more apparent abuse** at the beginning, **but that doesn't help us here**
  - 4) **I don't completely get it either. I can live with it,** but I didn't want to expand on the concept because this paper is epidemiological and doesn't really address that question well. The statement is clearly hypothetical and I would suggest we leave as is and see how reviewers react.

NOTES FROM DOCUMENT NRGG0230 – Internal Document about NUCYNTA PR

- **RADAR data is clearly being used to show Tapentadol is a better opioid Option**
- “Pitch mention of RADARS data on Tapentadol abuse, nonuse among college students”
- “In addition, two RADARS System surveillance data also shared at ] AAPM showed that NUCYNTA® has low rates of abuse, misuse \ and diversion. In fact, one study showed that among college \ students, the rate is low and decreasing over time.”

**OVERALL = Manipulating data to make Tapentadol seem to have less abuse-potential than it really has?**

110) Speaker Agreement

Document Data

- **Author :** Cephalon, Inc; Repella, Robert
- **Document Date :** 2011 May 06
- **Type :** agreement
- **ID :** lfhg0230 ( TID : vqf71j00 )
- **ARK :** ark:/88122/lfhg0230
- **Collection :** Oklahoma Opioid Litigation Documents; Opioid Documents Collection

Document Notes

- APRIL VALLERAND = **Contracted speaker with Cephalon**
  - Also one of the authors of Chronic Pain Management Strategies and Lessons from the Military
- Contract between April Vallerand and Cephalon
  - Cephalon = FENTORA (fentanyl)
- Services
  - a. Cephalon may engage Speaker to conduct presentations at Cephalon CSP (Cephalon Speaker Programs)
- Compensation
  - Cephalon shall pay Speaker a fee in accordance with honoraria guidelines established by Cephalon and described in Exhibit C (Compensation and Training).
  - The parties agree that the compensation provided hereunder has been established pursuant to aims length negotiations between the parties and is consistent with the fair market value of the services provided by Speaker under this Agreement
- Standards
  - Speaker will have access to a Cephalon-approved slide kit. Speaker must present this slide kit. Speaker must never use their own slides or other presentation materials for any purpose during the course of a CSP
  - Product-Specific Questions
    - In response to an "on-Iabel" question in the Q&A session. Speaker may answer the question verbally and/or use the Cephalon approved promotional slides
    - In response to an "off-label" question in the Q&A session, Speaker must not display or distribute any slides or other materials, but rather, if Speaker chooses, may provide a verbal answer to the question
    - Speaker's answer to questions must present the information in an accurate, fair and balanced, and objective manner, and must disclose the basis for answering the question (e.g. personal clinical experience, or data from a clinical study).
- Confidentiality. Speaker agrees to maintain in confidence, beyond the termination of this Agreement, and to not disclose to any third partly the terms of this Agreement, any information provided to Speaker by Cephalon under 'litis Agreement, as well as any password(s) that can be used to access any Cephalon Speaker Bureau web site. Speaker also agrees not to provide copies of the Cephalon slide kits to any third party, including any other pharmaceutical ( company.

124) Current Concepts in Pain Management

Document Data

- **Author :** PriCara; Ortho-McNeil-Janssen Pharmaceuticals
- **Document Date :** 2011 January 27
- **Type :** report; publication
- **ID :** pqgg0230 ( TID : xnf71j00 )
- **ARK :** ark:/88122/pqgg0230
- **Collection :** Oklahoma Opioid Litigation Documents; Opioid Documents Collection

Document Notes

- **Project = Pain Advocacy Partners Resource Kit; Nurse’s Newsletter; NUCYNTA**
  - Advertising and Promotion???
- Editor (and author of multiple articles) = **PAUL ARNSTEIN, RN, PHD**
  - Paid consultant for PriCara (which is now Jansenn); has been compensated for serving as an author and editor on this publication
- Author of article = **SUSAN PANDERGRASS**
  - Paid consultant for PriCara; has been compensated for serving as an author on this publication
- Editor & Author = **YVONNE D’ARCY, MS, CRNP**
  - Paid consultant for PriCara; has been compensated for serving as an author and editor on this publication
[truncated: 448,593 more chars]
