# Supplementary material for: Pharmaceutical industry use of key opinion leaders to market prescription opioids: A review of internal industry documents
Source: Explor Res Clin Soc Pharm. 2024 Nov 19;16:100543. doi: 10.1016/j.rcsop.2024.100543 (PMC11647219; doi:10.1016/j.rcsop.2024.100543)
Supplement: Supplementary file 2 — Supplementary material 2 [file mmc2.docx]

KOL Paper Supplementary Table

**Topics**

- **Legal filings [S1.0]**
- **Vulnerable populations**
  - **Children**
  - **Military**
  - **Elderly**
- **Academic Malfeasance**
  - **Ghostwriting**
  - **Enriched Enrollment**
  - **Research Laundering**
  - **Other**
- **Other relevant topics**
  - **Advocacy Groups**
  - **Key Opinion Leaders**
  - **Continuing Education**

1. Directors Meeting ACTIQ
   - [https://www.industrydocuments.ucsf.edu/docs/yhgg0230](https://www.industrydocuments.ucsf.edu/drug/docs/#id=yhgg0230)
   - Author : Unknown
   - Document Date : 2004 December 02
   - Type : agenda; chart; graph; photograph; website
   - ID : yhgg0230 ( TID : iif71j00 )
   - ARK : ark:/88122/yhgg0230
   - Collection : Oklahoma Opioid Litigation Documents; Opioid Documents Collection
   - Key Points:
     1.
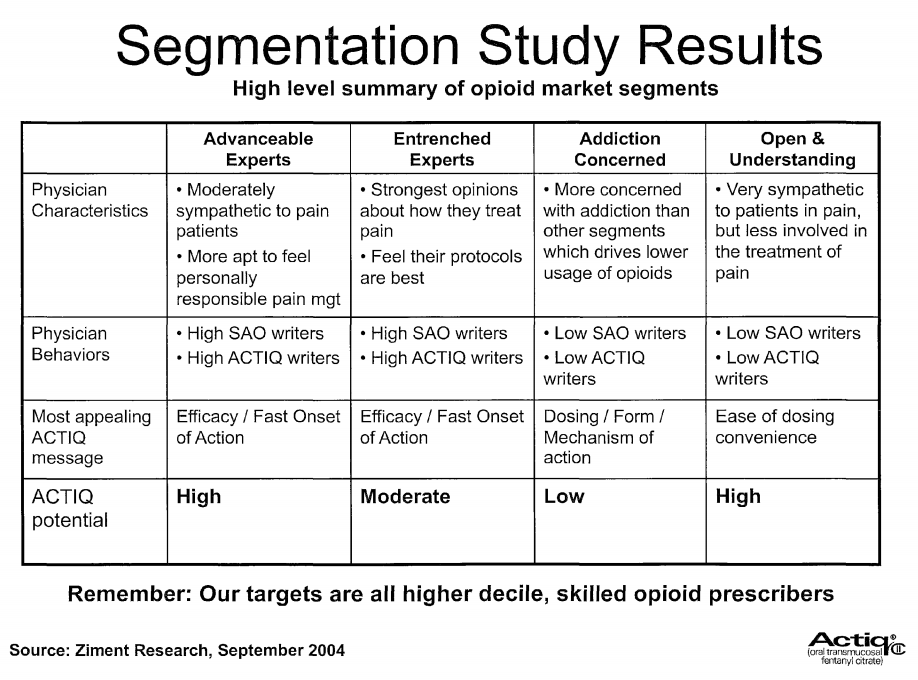

     2. “2004 External Challenges
        1. Increased media attention – Q2 2004
        2. Increased scrutiny from law enforcement and regulatory agencies
           1. Meetings with states AG and FDA
        3. Difficult process for promotional materials development given FDA review & comment
           1. Pulled ALL promo materials in August
        4. Growing ‘opiophobia’
           1. Concerns of abuse/addiction/diversion
           2. Concerns with increased prescriber scrutiny
        5. Increasing reimbursement barriers”
     3.
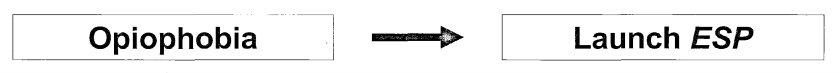

     4.
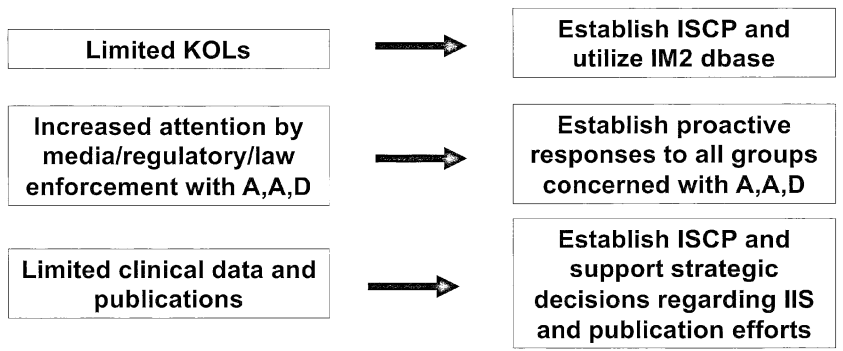

     5.
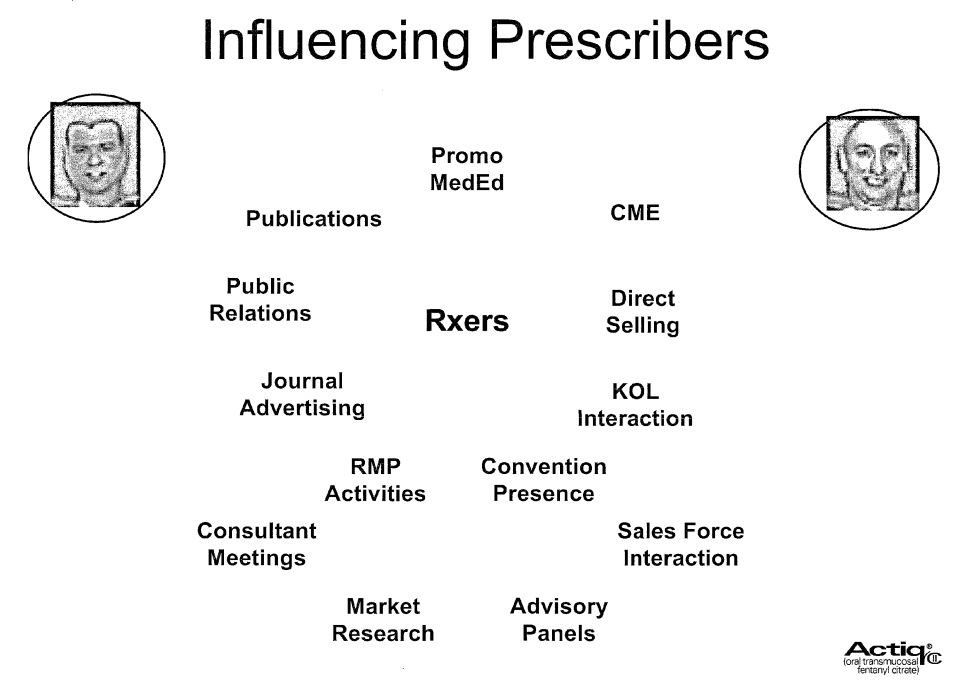

     6.
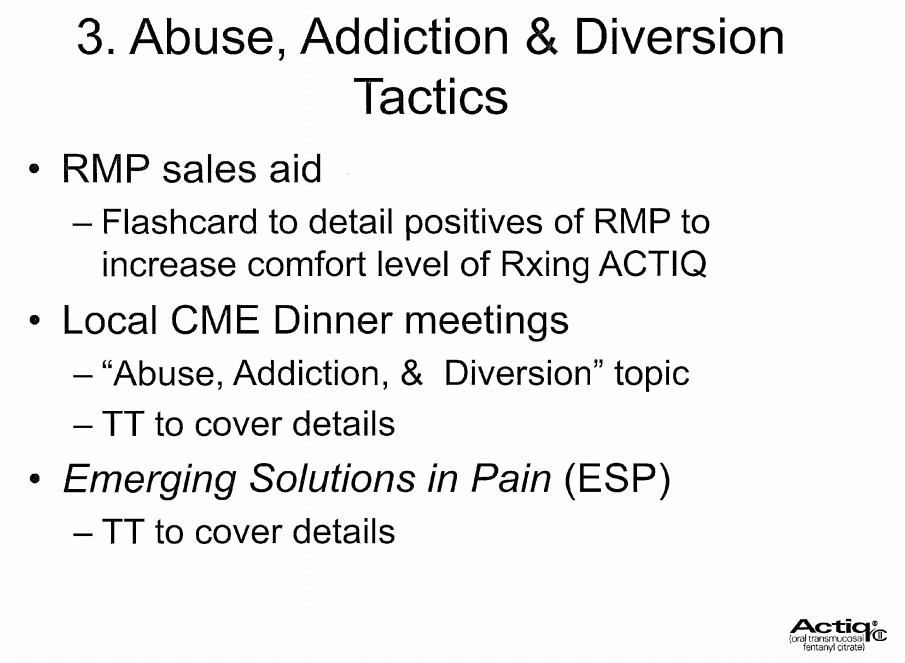

     7.
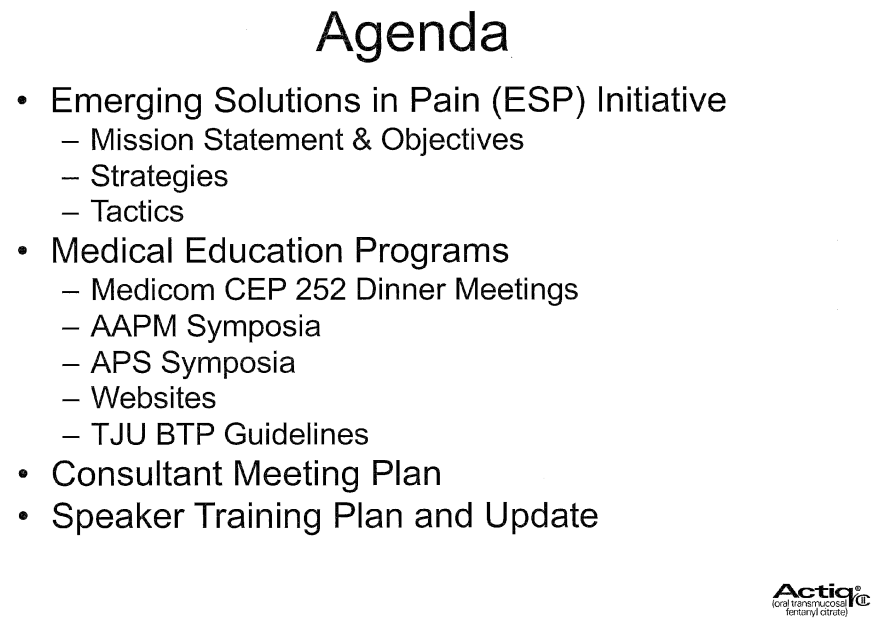

     8. “Emerging Solutions in Pain (ESP) is an ongoing initiative that is being developed by physicians for physicians, pharmacists and other healthcare professionals, to address some of the most critical issues in pain management today. These issues involve balancing the fundamental rights of patients and clinicians with the challenge of identifying patients who are at greater or lesser risk for opioid misuse and addiction, and with the challenges associated with the complex regulations involved in prescribing controlled substances. Through the expertise of a cadre of leading pain and addiction medicine experts, the ESP program will provide clinicians with guidance in the implementation of good practice management techniques, emphasizing favorable interaction with regulatory and law enforcement agencies, as well as, effective assessment, monitoring and documentation strategies, which will contribute to the overall goal of optimizing outcomes for their pain patients. ESP is a branded educational initiative supported by Cephalon, Inc and the Pain Franchise through an unrestricted educational grant.”
     9. “ESP Objectives

We've taught physicians the benefits of opioids and how to prescribe them, but neglected to adequately teach them about the risks. - Russell K. Portenoy

- - - 1. Broad-based
      2. Branded
      3. Awareness
      4. Education
      5. Improve Practice Management for Practitioners
      6. Minimize the risks of prescribing and dispensing opioids for physicians, pharmacists ana patients
      7. Ensure that pain is identified and treated appropriately
      8. Enhance Cephalon image as a Leader in Pain Management”
    1. “Project Overview
       1. Ongoing Initiative
       2. Previously named "Reduce the Risk"
          1. Originally focused on minimizing diversion and abuse
          2. Tool Kit to help assess and manage risk
       3. Emerging Solutions in Pain (ESP)
          1. Proposed as a broad-based "educational" initiative
          2. Supported through an unrestricted educational grant by Cephalon & the Pain Franchise
          3. Risk Minimization is an integral and core component of ESP”

1. Speaker Agreement

Document Data

- Author : Brookes, Lynne M; Argoff, Charles
- Document Date : 2006 April 09
- Type : agreement
- ID : gsgg0230 ( TID : uof71j00 )
- ARK : ark:/88122/gsgg0230
- Collection : Oklahoma Opioid Litigation Documents; Opioid Documents Collection

Document Notes

- Speaker agreement between CEPHALON and **CHARLES ARGOFF**
- Cephalon wishes to obtain the services of Healthcare Provider to spttak for Cephalon in areas of medical interest about Cephalon's marketed pharmaceutical products, knd Healthcare Provider wishes to provide such speaking services, all subject to the terms and conditions of this Agreement.
- As compensation for Healthcare Provider's performance of the services to he performed by Healthcare Provider under this Agreement, Cephalon **shall provide honoraria and shall reimburse Healthcare Provider** (when appropriate and in compliance with law) for out-of-pocket travel, hotel, meal and other expenses reasonably incurred in accordance with Cephalon's reimbursement guidelines described in Exhibit A

1. Use of Opioid Analgesics in Pain Management
   - [https://www.industrydocuments.ucsf.edu/docs/llgg0230](https://www.industrydocuments.ucsf.edu/drug/docs/#id=llgg0230)
   - Author : Candiotti, Keith; Janssen Pharmaceuticals, Inc
   - Document Date : Unknown
   - Type : article; website
   - ID : llgg0230 ( TID : rkf71j00 )
   - ARK : ark:/88122/llgg0230
   - Collection : Oklahoma Opioid Litigation Documents; Opioid Documents Collection
   - Key Points:
     1. “*Expert authors received compensation from Janssen Pharmaceuticals, Inc. for their contributions to PrescribeResponsibly.com”*
     2. “By the same token, patients report similar concerns about developing an addiction to opioid analgesics.^17^ While these concerns are not without some merit, it would appear that they are often overestimated. According to clinical opinion polls, true addiction occurs only in a small percentage of patients with chronic pain who receive chronic opioid analgesics analgesic therapy.^18^”
        1. Auret K, Schug SA. Underutilisation of opioid analgesics of opioid analgesics in elderly patients with chronic pain: approaches to correcting the problem. *Drugs Aging.* 2005; 22(8): 641-654.
        2. Fishbain DA, Cole B, Lewis J, et al. What percentage of chronic nonmalignant pain patients exposed to chronic opioid analgesics analgesic therapy develop abuse/addiction and/oraberrant drug-related behaviors? A structured evidence-based review. *Pain Medicine.* 2008; 9(4):444-459.
2. [Agenda for the Chronic Pain Scientific Advisory Board]
   - [https://www.industrydocuments.ucsf.edu/docs/fggg0230](https://www.industrydocuments.ucsf.edu/drug/docs/#id=fggg0230)
   - Author : Chronic Pain Scientific Advisory Board
   - Document Date : 2001 November 30
   - Type : agenda
   - ID : fggg0230 ( TID : jhf71j00 )
   - ARK : ark:/88122/fggg0230
   - Collection : Oklahoma Opioid Litigation Documents; Opioid Documents Collection
   - Key Points:
     1. Confidential
     2. “Objective: KOLs to visit Janssen, understand J&J and Janssen, introduce Janssen’s EMRP research program with MSLs as key communication links, find common interests for research alliances, obtain feedback on the EMRP research agenda, and discuss issues related to abuse and diversion of opioids.”
     3. “Audience: 17 KOLs (List attached)”
     4. “EMRP definition:
        1. Studies focused on Duragesic in non-malignant pain states (Schein)
        2. ‘Nimble’ trials, tightly designed, small-scale, clearly defined with limited duration
        3. Pilot studies
        4. Anticipated output: abstracts presented at Association meetings (e.g., AAPM, APS)
        5. Funding has ranged from $15K - $200K and is dependent on:
           1. # of subjects
           2. complexity of the protocol, e.g., retrospective study ~$15K-25K, larger, multisite study ~$200K, average ~$100K
           3. complexity of assessments
           4. # researchers involved
           5. # required auxiliary personnel (e.g., statisticians)”
     5. “The goals for EMRP studies should be explicitly stated: Janssen wants to obtain certain data and seed studies that, after completion, may be expanded by funding from other sources.”
     6. “Review of the Allan and Milligan Data

Cheryl Pavia, PharmD

Cheryl's review of the new Allan and Milligan data, which came in between the EMRP sessions summarized above, was well received and elicited significant discussion. Many participamnts seemed unfamiliar with the studies. Many questions arose, which Cheryl fielded expertly.

- - - 1. Why was Allan's reported "better pain relief" with Duragesic accompanied by higher use of rescue medications.
      2. Cheryl responded that, because Duragesic has 4 patch strengths, many in-between doses may have been required.
      3. A "major stumbling block" in using Duragesic is the difficulty of converting from SAOs or other LAOs to Duragesic, which requires many iterations. The PI is to conservative, we need a more realistic conversion chart.
      4. Cheryl responded that the PI puts the patient in the ballpark for pain control and the physician should titrate up from there.
      5. That's the problem, Dr. Benjamin responded, PCPs do not know how to titrate.
      6. Cheryl indicated that this was an area for education and that Janssen had a major commitment to an education initiative for PCPs.
      7. Cheryl queried the physicians on what conversions they used.
      8. Some referenced the Breitbart paper on a dosing algorithm for transdermal fentanyl in cancer pain.
      9. Others said the 25 mcg patch ≠ 100 mg of morphine. It is more like 100 mg morphine = 200 meg patch.
      10. Allan and Milligan do not give their conversion calculations, this is a weakness.
      11. They do not state how long initial side effects lasted and this is also important information to know.”
    1. “What does drug abuse mean to you?
       1. Compulsive use; prescription drug abuse or diversion; drug seeking for a high; continued use of the drug despite harm to self or others; medicine not used for its intended purpose.
       2. Needed” o nosology of the negative outcomes from drug abuse. Is drug abuse a clinical outcome or a sociological phenomenon? (Katz)
       3. The DSM4 defines drug abuse as: craving, compulsive use, causing harm, so we don’t get to define the medical illness, but we have to define its cause and manage its sequellae. (King)
       4. Drug abuse is not in the realm of medication domain but refers to using narcotic drugs for uses other than pain relief, e.g., selling or recreational use. (Kerns)
       5. We need a new terminology, currently we use words derived from the field of drug and alcohol abuse. The use of these terms is not scientifically valid, these are not RX medication behaviors. (Gallagher)”
    2. “Should the abuse potential of Duragesic be discussed?
       1. "NO" - resounding and unanimous. It is bad for the LAO class and bad for patients and prescribers.
       2. Drug abusers will figure out how to abuse Duragesic once it is more available. Currently, it may be less abused and there is a dangerous narrow margin between a recreational dose to get high and a lethal dose. As market share goes up, so will abuse. Overpromising on the lack of abuseability is what got OxyContin in trouble. Duragesic should not repeat the same mistake.
       3. Dissenting opinion: comparing the patch to Percocet, the patch is less abuseable, less prone to self-administration behavior. For high risk patients, I give them the patch.
       4. It requires a lot of education for physicians and patients, you can't just say the patch is less abuseable.
       5. The relapse to drug abuse is a complex issue, involving many social and psychological factors. It would be better to define, measure and validate the risk factors for abuse.”
    3. Discussion of different messaging: “Conclusion: Do not include the abuse message. Do not sell opioids on the abuse issue.”
    4. “In their research, Drs. Passik and Portenoy have developed an assessment instrument, currently in validation trials, to monitor outcomes in chronic opioid therapy.”
    5. “Once Passik and Portenoy's assessment tool identifies that a patient is exhibiting aberrant behaviors, the clinician must determine why the behavior is occurring. The reason determines the clinical course of action. Aberrant behaviors may indicate:
       1. Addiction
       2. Pseudo-addiction
       3. Other psychiatric illness
       4. Family dysfunction
       5. Criminal intent”

1. [Email from Stephen Cornwell to Dennis Fitzgerald Regarding the Master KOL Listing]
   - [https://www.industrydocuments.ucsf.edu/docs/gygg0230](https://www.industrydocuments.ucsf.edu/drug/docs/#id=gygg0230)
   - Author : Cornwell, Stephen
   - Document Date : 2005 February 01
   - Type : email
   - ID : gygg0230 ( TID : imf71j00 )
   - ARK : ark:/88122/gygg0230
   - Collection : Oklahoma Opioid Litigation Documents; Opioid Documents Collection
   - Key Points:
     1. “Business Objective
        1. In order to target the most influential physicians in Pain management, and to maximize DURAGESIC® prescribing that is produced by physicians who are influenced by thought leaders Janssen needs to identify Key Opinion Leaders in Chronic Pain market at
           1. National Level
           2. Regional Level

and to understand the overall pattern and relative influence of KOLs on physician prescribing”

- - 1. “Methodology
       1. A Questionnaire based survey was conducted amongst 1,000 physician
          1. 200 physicians recruited in each of five Green SF regions
          2. All doctors recruited from top six deciles of Duragesic prescribing
       2. The respondents were asked to allocate points to factors that influenced their overall prescribing in order of importance on a scale of 100
          1. Opinion leadership
          2. Journal articles
          3. CME or other ‘events’
          4. Sales rep detailing
          5. Etc.
       3. This question was used to gauge the overall importance of KOLs in this sample without regard to specific individual relationship
       4. Each respondent was asked to list 3 national and 3 regional KOLs
          1. ‘Regional’ KOLs were defined as being ‘located within 250 miles of your practice’
       5. They were also asked to provide statistics on some of the influencing factors such as:
          1. Number of events attended where that specific KOL spoke
          2. Number of articles read that were written by that specific KOL
          3. Influence Rating of that KOL on the respondent’s prescribing, using a scale of 1 to 7”
    2. “Points Allocation Summary of the Parameters
       1. Of the nine Parameters, three had the highest mean. Of those three, ‘Peer reviewed Journal articles’ and ‘Medical Education’ were rated as 100 by about 30% of the respondents.”
    3. “Points Allocation Summary by Specialty Groups”
       1. “All specialties rate ‘Sales Representative Messages’ as relatively unimportant, although statistical modeling of promotion response will generally show it to have more effect than a doctor realizes.”
    4. “National Key Opinion Leaders”
       1. “The distribution of KOLs by number of ‘mentions/ (number of respondents identifying them as KOL) was very spread out.
          1. A relatively small number of doctors with multiple mentions (true ‘National’ KOLs)
          2. A moderate (but not large) number of doctors with a few mentions
          3. A large number of doctors identified by only one respondent”
       2.
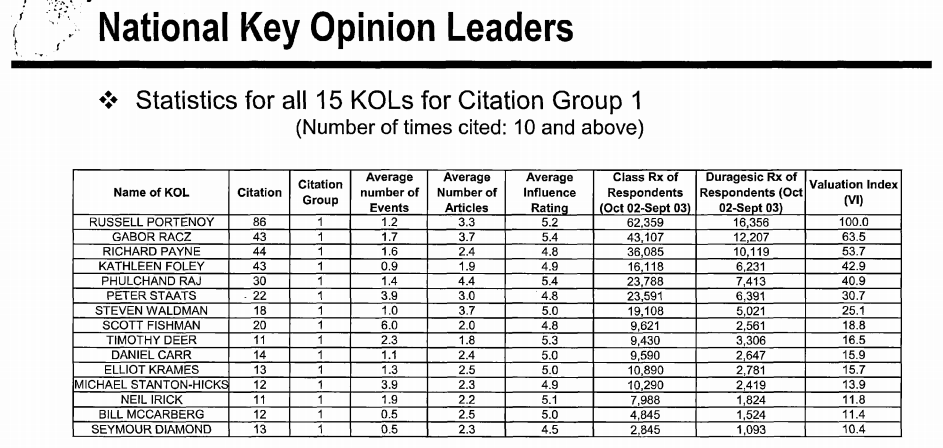

    5. “Correlations of events and articles with Influence scores
       1. Overall, correlations seem to be very weak between events and articles with influence rating for KOL
       2. For respondents that ranked ‘Influence of Opinion Leaders’ as High (influence Group =3), a high degree of influence by their specifically-named KOLs seems to be associated more with the number of events attended as compared to Journal articles read.”
       3. “The positive correlation between events and articles primarily indicates that respondents tend to read (or remember reading) articles by the sane authors that they remember hearing as speakers”

1. Payment History by JandJ or Janssen to specified individuals – based upon deposition of Kimberly Deem-Eshelman
   - [https://www.industrydocuments.ucsf.edu/docs/rngg0230](https://www.industrydocuments.ucsf.edu/drug/docs/#id=rngg0230)
   - Author : Janssen
   - Document Date : 2019 January 25
   - Type : report; legal; table
   - ID : rngg0230 ( TID : dmf71j00 )
   - ARK : ark:/88122/rngg0230
   - Collection : Oklahoma Opioid Litigation Documents; Opioid Documents Collection
   - Key Points:
     1. “Prepared based on a reasonable investigation conducted as of the deposition of Kimberly Deem-Eshleman on January 25, 2019. Janssen reserves the right to amend or supplement this information as appropriate.”
2. Duragesic Focused and Targeted Execution – 2004 Business Plan

Document Data

- Author : Janssen Pharmaceutica
- Document Date : 2003 August 06
- Type : presentation; slides
- ID : fxgg0230 ( TID : pif71j00 )
- ARK : ark:/88122/fxgg0230
- Collection : Oklahoma Opioid Litigation Documents; Opioid Documents Collection

Document Notes

- Title of Slides = **DURAGESIC; FENTANYL TRANSDERMAL SYSTEM; FOCUSED & TARGETED EXECUTION** (2004 business plan)
- Market Analysis – Growth Drivers
  - Recognized undertreatment of pain
    - Increased legislation
    - **Mandatory CE/CME**
    - Litigation for undertreatment
  - Acceptance of Opioids for non-cancer pain
  - **Consumers more demanding**
  - New & future competitive entries
- Market Analysis – Growth Inhibitors
  - **Perceived “risk” of opioids -- OPIOPHOBIA**
  - **Limited evidence-based scientific data**
  - Increased State/MCO restrictions
- Duragesic statement = **Life, Uninterrupted** and  **Work, Uninterrupted**
  - Duragesic positioning statement – Duragesic significantly improves physical and social functioning by providing the only chronic pain relief that is consistent and effective for 72 hours
- Non-cancer pain is the growth opportunity
- Differentiate DURAGESIC from competition
  - Focus on physician and patient end goal
  - Continue to leverage KOL relationships: Med affairs, NPEC, National Pain Summit
  - Enhance KOL, Pharmacist & key customer relationships within market
- Internal Assessment
  - Lessons Learned: functionality positioning aligns with physician and patients’ end goal of therapy 🡪 Brand messages have evolved to a more patient centric platform
- Internal Assessment: SWOT Analysis & Issue Identification
  - Weakness – Limited clinical data
  - Opportunities – Functionality message ownership
  - Threats – Opioid abuse publicity/ media & potential awareness
- **See “Medical Affairs; 2004 Key Projects” screenshots – potential evidence of seeding trials, ghost writing, general academic malfeasance, CME planning**


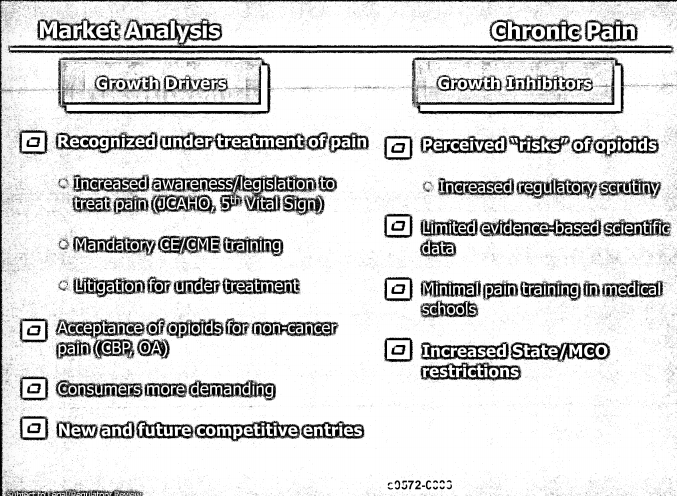


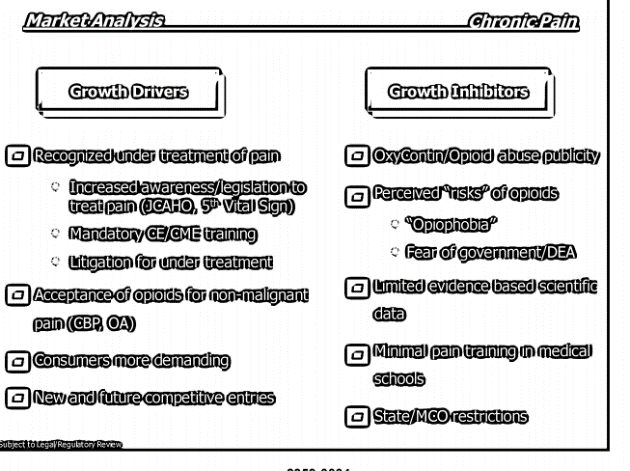


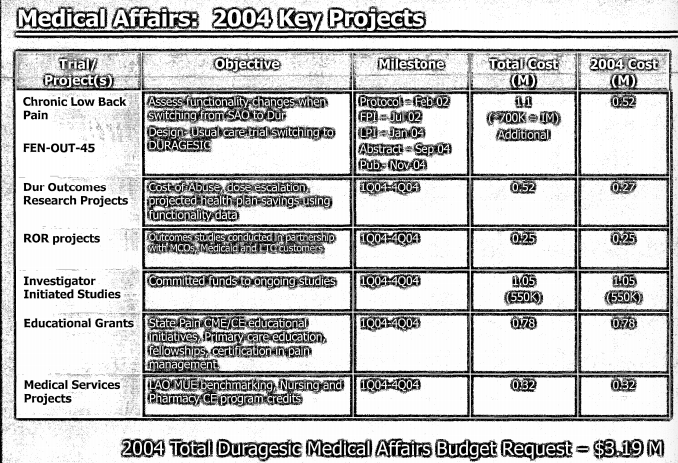


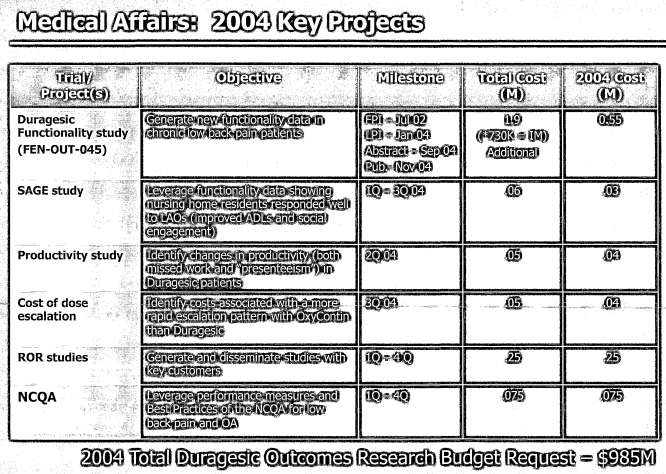


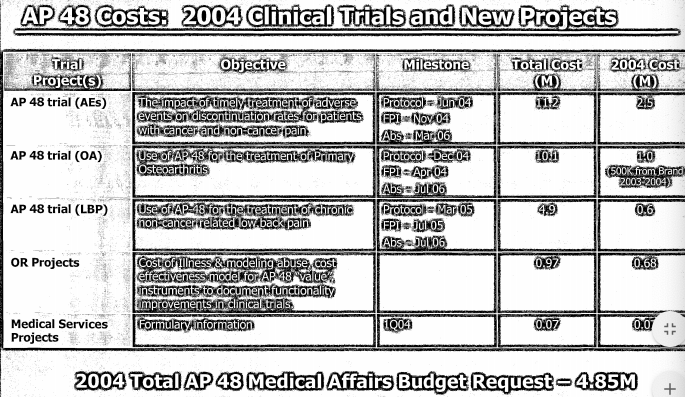


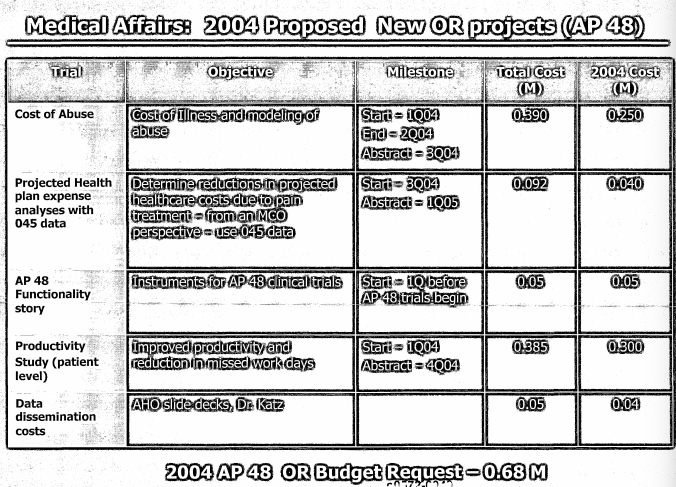


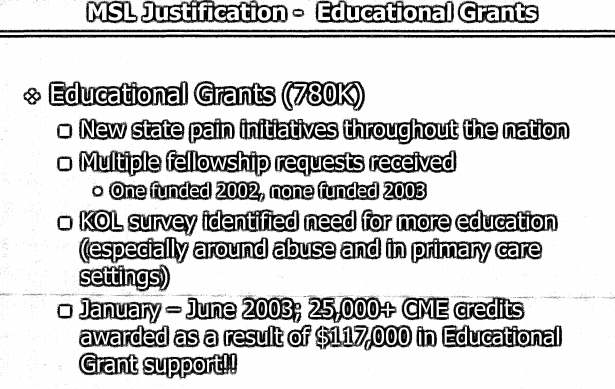


1. Key Opinion Leader (KOL) Development Plan for Cephalon Pain Franchise
   - [https://www.industrydocuments.ucsf.edu/docs/hygg0230](https://www.industrydocuments.ucsf.edu/drug/docs/#id=hygg0230)
   - Author : Neumann, Chris; Toscani, Michael; Cephalon
   - Document Date : 2005 February 04
   - Type : presentation; report
   - ID : hygg0230 ( TID : jmf71j00 )
   - ARK : ark:/88122/hygg0230
   - Collection : Oklahoma Opioid Litigation Documents; Opioid Documents Collection
   - Key Points:
     1. “Value of KOLs (cont.)
        1. Surveys for >25 years have shown that the #1 reason a MD changes prescribing behavior is due to ***peers***
        2. Healthcare professionals learn through an apprenticeship model…..throughout their careers
        3. In spite of their importance…most pharma companies do not currently have an effective system in place to identify, manage or develop OLs”
     2. “Importance of KOLs
        1. Critical to the success of new product launches
        2. OLs help to shape:
           1. Clinical drug development
           2. Product positioning
           3. Brand development/life cycle management
           4. Prescribing practices
           5. ….. i.e. $$$”
     3.
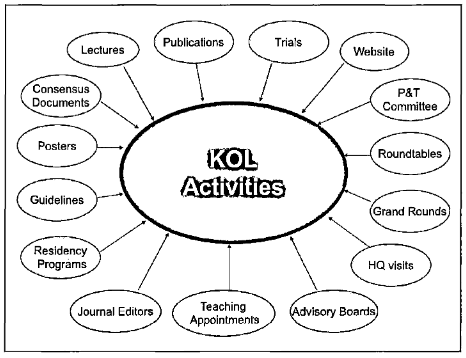

     4. “Diminishing Effectiveness of Reps
        1. Only 20% speak with a MD…
           1. And then only for <2 minutes
           2. Only 8% are remembered
           3. 65% drop samples w/o seeing MD
        2. Reps often poorly informed
        3. Discontented, ineffective district managers
        4. MDs aren’t getting the info they need
           1. Patient perspective, cost, compliance, formulary status, managed care co-pay
           2. Literature, off-label use, OL POV, safety, QoL
        5. MDs feel besieged – pharma consolidation”
     5. “Keys to Building OL Relationships
        1. Cultivate OL relationships throughout the product lifecycle
        2. Build a strong support infrastructure
           1. Minimize administrative tasks
        3. Align MSL activities with overall corporate strategy and market positioning
        4. Establish a distinct MSL recruitment process
           1. Balanced scientific and business experience
        5. CQI process”
     6. “Negotiating with KOLs
        1. Payment
           1. Local vs. regional vs. national lectures
           2. Advisory boards
           3. Clinical trials
        2. MSL involvement
        3. Managing difficult KOLs
        4. … Consistency is the key”
     7. How to integrate KOLs into trials
        1. Advisory Capacity
           1. Protocol design
           2. Evaluation of literature
           3. Medical community POV
           4. Competitive Intelligence
        2. Trial enrollment
        3. Strategic Communications Plan
           1. Publications – abstracts, posters, original articles, reviews, guidelines, ‘white papers’, enduring materials. etc
           2. Lectures – national society, satellite symposia, state society meetings, grand rounds, local dinner meetings, etc
           3. Press Releases”
     8. “Trial Recruitment Tips
        1. Flyers
        2. Newspaper / radio ads
        3. Laminated cards
        4. Protocol Education
           1. Resident lunch
           2. In-service
        5. Slide development
        6. Identify Issues
        7. Communication ‘monitoring’ form”
     9. “KOL Validation
        1. Define variables
           1. Publications, lectures, trials, editorial boards, society offices, guidelines, FDA advisory committee, training program director, advisory boards, books, committees, academic appointments, etc
        2. Establish weighted importance
        3. Compare and Contrast KOLs”
     10.
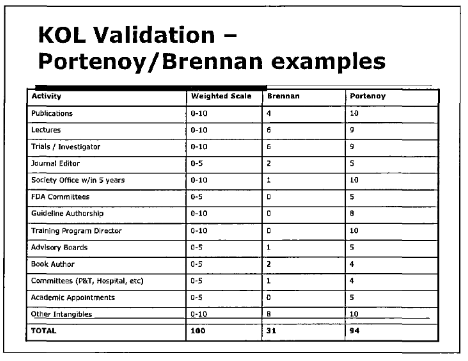

     11. “Objectives
         1. Identify top-tier KOLs at national, regional and local levels
         2. Establish budget for KOL development
         3. Understand the prescribing behaviors & motivations of a very diverse target audience to effectively deliver optimal messages
         4. Identify current supporters and critics”
     12. “Critical Issues
         1. Small number of KOLs (<100) influence hundreds of prescribers
         2. A small number of prescribers (<500) generate large dollar volume
         3. All pain pharma companies are actively seeking the support of the same, few KOLs
         4. Cephalon does not have a field-based medical group (e.g. MSLs)
         5. Cephalon does not have pain-specialty reps”
     13. “OVF – Critical Success Factors
         1. Differentiate OVF vs. ACTIQ and other SAOs in clinical trials
            1. Conduct studies & publish/present results consistent with commercial need
         2. Build KOL/Society loyalty and relationships
         3. Raise recognition of proper assessment and Tx of BTP in CA & non-CA
            1. Establish clinical, QoL & economic benefits
         4. Establish ‘RAO’ term and link to BTP as appropriate Tx
         5. Build and disseminate value proposition story for OVF for managed care/payers, patients and healthcare providers
         6. Establish Cephalon as valued partner to pain community
         7. KOLs and pain societies endorsement of OVF”
     14. “Action Plan – Broad
         1. Analyze, segment and validate KOLs
            1. Validate ‘IM2’ database
         2. Identify ‘Centers of Excellence’
         3. Meet with executives of key professional societies (AAPM, APS, ONS, AAPM&R, ASRA, ASCO, ASA, NCQA, JCAHO, AMA)
         4. Gain approval for KOL Development budget
         5. Build relationships with patient advocacy groups”
     15.
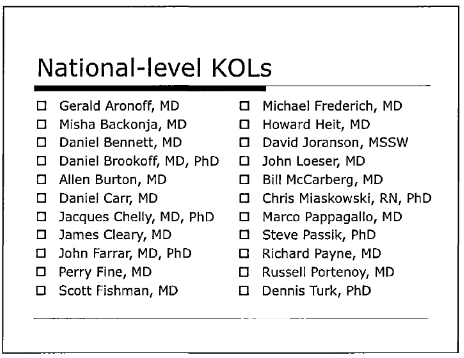

     16. “Action Plan – National KOLs
         1. Establish national Pain Medicine Executive Advisory Board and meet at least 2x/year (e.g. April & Nov.)
         2. Conduct advisory boards for managed care, pharmacy, nursing, risk management
         3. Attend and conduct activities at key pain society meetings (educational symposia, posters, hospitality suites, investigator meetings, exhibit booths, ‘reporter’ interviews/highlights, Cephalon pain award, one-on-one meetings)
         4. ESP Booth
            1. Meet-the-professor sessions”
     17. “Action Plan – National KOLs (cont.)
         1. Maximize BTP Guidelines – follow-up projects
            1. Gain society endorsement
            2. Publish ‘how to’ / ways to implement
            3. Publish case studies
            4. Publish effectiveness of guideline implementation
         2. Involve national KOLs in Pub Plan
         3. CME/ACPE/ANCC programs
         4. IIS/Phase 4/Patient Registry
         5. SageMed Extranet – push/pull marketing to registered KOLs
         6. Fellowship Training Program
         7. Data Mining
         8. PR Activities”
     18.
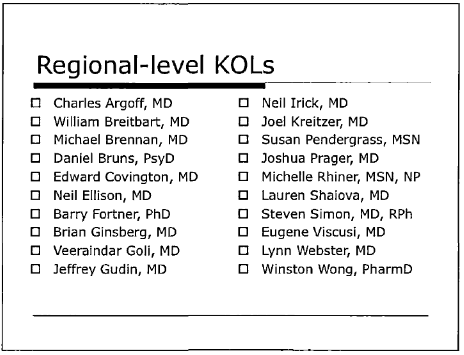

2. NEO Agenda
   - [https://www.industrydocuments.ucsf.edu/docs/zngg0230](https://www.industrydocuments.ucsf.edu/drug/docs/#id=zngg0230)
   - Author : Ortho McNell Janssen Pharmaceuticals Inc
   - Document Date : 2008 July 25
   - Type : agenda; outline; proposal; publication; report; table; website
   - ID : zngg0230 ( TID : gmf71j00 )
   - ARK : ark:/88122/zngg0230
   - Collection : Oklahoma Opioid Litigation Documents; Opioid Documents Collection
   - Key Points:
     1. “Objectives
        1. Launch NEO Module 3 to further disrupt and development the acute pain market (2 hours)
        2. Communicate and train on key tactics for delivering NEO message to targeted pharmacies”
     2. “Progress Check
        1. How do you know if you achieved Module 1: Undertreatment Goals?
           1. Establish that moderate to severe acute pain continues to be undertreated
           2. Familiarize physicians with the Pain Management Index (PMI), a useful tool that supports the appropriate use of analgesics”
     3. “Progress Check
        1. How do you know if you achieved Module 2: Consequences of Unresolved Acute Pain?
           1. Goals

Underscore the urgency of achieving effective relief of moderate to severe pain as soon as possible

Help HCPs make the connection between the undertreatment of acute pain and the risk of developing chronic pain

Support the choice of appropriate analgesics for moderate to severe pain”

- - 1. “NEO Pathways Module 3: Barriers to Optimal Pain Management
       1. Goals
          1. Raise prescribers’ awareness of how important tolerability is to their patients who are candidates for an opioid analgesic
          2. Address concerns about regulatory scrutiny and risk for disciplinary action against physicians who are prescribing opioids appropriately in the treatment of moderate to severe pain
          3. Raise awareness of the importance of physician-patient communication in identifying and overcoming barriers to optimal pain management”
    2. “Module 3: Key messages
       1. For many patients, the desire to avoid opiod {sic} side effects can be more important than pain control
       2. Although many physicians are reluctant to prescribe controlled substances, the risks (for both patient addiction/misuse and physician disciplinary action) are much smaller than commonly believed
       3. Better communication between patients and HCPs can help to change the course of moderate to severe pain”
    3. “Execution tip #1:

Avoid the Addiction Ditch

- - - 1. Use Portenoy’s study to create dialogue about Opiophobia as a barrier
         1. Many HCP’s will find the 2.6% incidence of addiction to be extremely low
         2. It’s not about the %, but about the barrier contributing to the undertreatment of pain”
    1. “Execution tip #2: Know the Difference
       1. HCP’s often do not distinguish between addiction, dependence, and tolerance.
          1. **Addiction** is a disease characterized by behaviors that include one or more of the following: compulsive drug use, craving, impaired control over drug use, and continued drug use in spite of harm.
          2. **Physical Dependence** is a state of adaptation that is manifested by a drug class specific withdrawal syndrome that can be produced by abrupt cessation, rapid dose reduction, decreasing blood level of the drug, or administration of an antagonist
          3. **Tolerance** is a physiological adaptation to analgesic effect and occurs when the analgesic effect at a constant dose is diminished over time. An increased dosage is required to achieve the analgesic effect.”
    2. “NEO Pharmacy Initiative Objectives
       1. Time Frame: November to Launch
          1. Identify key pharmacies that stock and dispense CII drugs
          2. Develop relationships with all pharmacy staff
          3. Create pharmacy profile and document in ViewPoint
          4. Identify opportunities for and schedule NEO speaker programs for licensed pharmacists”
    3. “Address patient concern about efficacy, addiction, or withdrawal”
    4. “#1 Identify Key Pharmacies
       1. Review current list of called on pharmacies
          1. Are they aligned to your NEO targets?
          2. Do they stock and dispense CII drugs?
       2. Identify other key pharmacies in your territory that stock and dispense CII drugs
          1. Use new pharmacy data
          2. Ask your NEO targets which pharmacies their patients use most often
       3. Create pharmacy routing for consistent call frequency

1. The Use of Opioids for the Treatment of Chronic Pain
   - [https://www.industrydocuments.ucsf.edu/docs/pkgg0230](https://www.industrydocuments.ucsf.edu/drug/docs/#id=pkgg0230)
   - Author : American Pain Society; American Academy of Pain Medicine
   - Document Date : 1997
   - Type : publication; article
   - ID : pkgg0230 ( TID : fkf71j00 )
   - ARK : ark:/88122/pkgg0230
   - Collection : Oklahoma Opioid Litigation Documents; Opioid Documents Collection
   - Key Points:
     1. Consensus statement from the American Academy of Pain Medicine and the American Pain Society
2. TEVA Advocacy Mapping: Identifying Advocacy Partners to Enhance Patient Care

Document Data

- **Author :** Teva
- **Document Date :** 2013 March 28
- **Type :** report; agenda
- **ID :** lngg0230 ( TID : xlf71j00 )
- **ARK :** ark:/88122/lngg0230
- **Collection :** Oklahoma Opioid Litigation Documents; Opioid Documents Collection

Document Notes


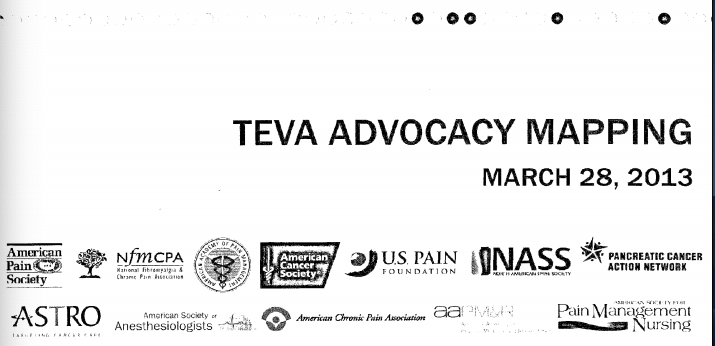


- Teva is relatively new to the pain community - specifically to those who manage chronic pain. It's critical to engage patient and professional advocacy groups to help establish positive relationships with both patients and HCPs
- GolinHarris analyzed the pain/oncology advocacy landscape to help identify and prioritize those groups with which Teva is most aligned. This proprietary GH program is designed to help brands better understand **potential allies and detractors** as an important early step in developing strategies to **engage and/or minimize them.**
- Scoring Criteria for Advocacy Groups
  - Organization Resources: Annual revenue, expenses, # of employees
  - Membership: # of members, **prescribing habits**
  - Visibility: Publications, congresses, social media, traditional media presence
  - Partnerships: Corporate opportunities and sponsorship; past history with pharma
  - Influence: Active on the Hill, medical guidelines, achievements
  - **Focus on Pain Management: Particularly relevant for oncology groups**


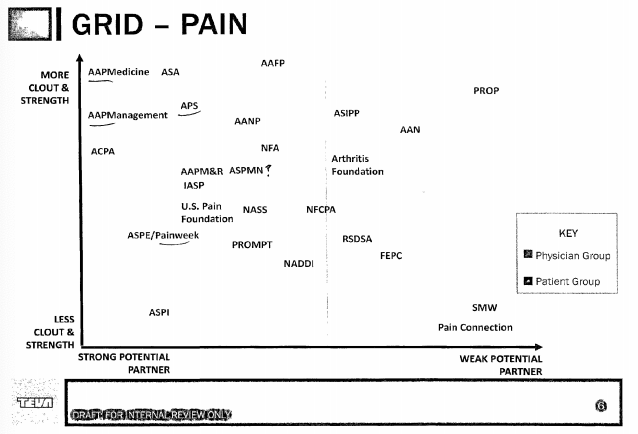


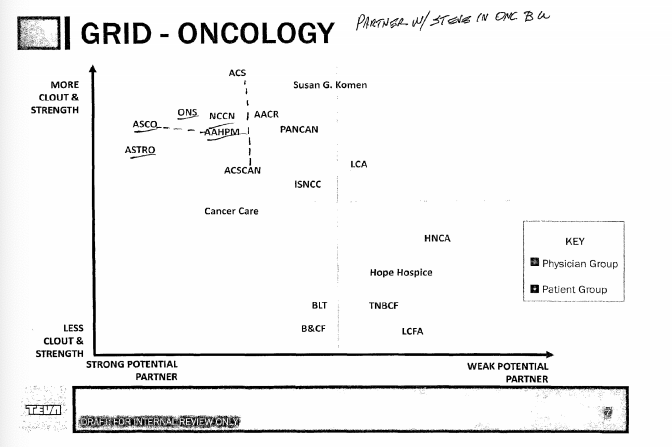


- Engagement Strategies: **For every advocacy group, TEVA has outlined how to engage the group and who are the main influencers (see example below with US Pain Foundation)**
-
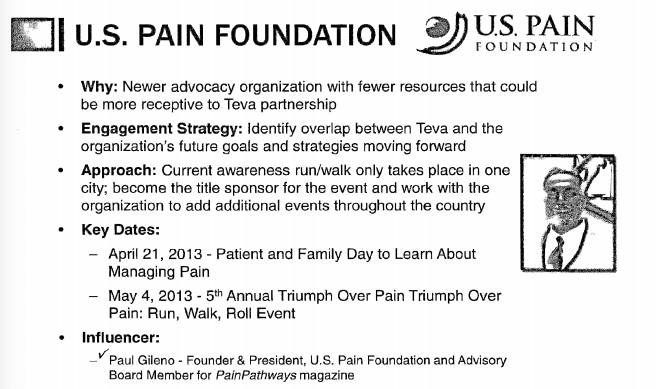

- INFLUENCERS – ORGANIZATIONAL
  - Keysha Brooks Coley
  - Scott Fishman
    - See #6
    - Author of “Responsible Opioid Prescribing: A Physician’s Guide”
    - HIs book was funded by Purdue (need to look back at #6, could have been funding by TEVA too)
  - Jeffrey Fudin
  - Bob Twillman
    - See #54
    - Twillman is a contracted spokesman for TEVA
  - Amy Abernethy
- INFLUENCERS – COE
  - John Farrar
  - David Craig
  - John Loeser
  - Steve Feinberg

Document relevance = **YES VERY IMPORTANT DOCUMENT!!! At the end, lists history of money accepted by each organization!**

1. KOL Categorization

Document Data

- Author : Thomas, Heather
- Document Date : 2002 February 18
- Type : memo
- ID : lhgg0230 ( TID : fif71j00 )
- ARK : ark:/88122/lhgg0230
- Collection : Oklahoma Opioid Litigation Documents; Opioid Documents Collection

Document News

- Email from Thomas Heather @ Jansen
  - Another consideration: remember the KOL I mentioned during my presentation at the MA meeting in Philly? **Told me he hated my company, would never take money for education from me, blah, blah?** By these criteria, he wasn't an opponent. I don't think he should have been considered one, either - he just likes a good argument. Are you guys OK with the concept that just because a KOL is obnoxious, difficult or a pain it doesn't mean he or she is an opponent? Conversely, that a sweetie who just isn't putting out for you shouldn't qualify as an advocate?
- **KOL OPPONENTS** – Not only are they not helping, they are actively hurting the cause. Can qualify by:
  - Refusing to meet with Jannssen MSL
  - Blocking access to the department by our sales rep
  - Giving talks that denigrate opioids or DURAGESIC
  - Teaching resides and fellows any of the above positions
  - Get hysterical, as opposed to intelligently concerned, about the abuse and diversion of opioids
  - Spread URBAN MYTHS about duragesic (Chicklets, shooting up the gel)
- **KOL NEUTRALS**: probably consider themselves good friends of Janssen but
  - Never mention opioids or DURAGESIC in their talks
  - Inadvertently pigeon-holes DURAGESIC for NPO, bowel-obstructed patients
- **KOL ADVOCATES:** Can present well-balanced completely fair material, but also
  - Alert MSL to upcoming publication of interests
  - Position DURAGESIC enthusiastically in chronic pain
  - Dispel myths about opioids and DURAGESIC in their teaching/talks
  - Create, review drafts of algorithms and guidelines with an eye to suitable mention of DURAGESIC
  - Says “Use non-invasive measure first like patches or oral” when giving pain lecture
  - Introduce MSL to other practitioners
